# A novel role for *trithorax* in the gene regulatory network for a rapidly evolving fruit fly pigmentation trait

**DOI:** 10.1371/journal.pgen.1010653

**Published:** 2023-02-16

**Authors:** Michael L. Weinstein, Chad M. Jaenke, Hasiba Asma, Matthew Spangler, Katherine A. Kohnen, Claire C. Konys, Melissa E. Williams, Ashley V. Williams, Mark Rebeiz, Marc S. Halfon, Thomas M. Williams

**Affiliations:** 1 Department of Biology, University of Dayton, 300 College Park, Dayton, Ohio, United States of America; 2 Program in Genetics, Genomics, and Bioinformatics, University at Buffalo-State University of New York, Buffalo, New York, United States of America; 3 West Carrollton High School, 5833 Student St., Dayton, Ohio, United States of America; 4 Department of Biological Sciences, University of Pittsburgh, Pittsburgh, Pennsylvania, United States of America; 5 Department of Biochemistry, University at Buffalo-State University of New York, Buffalo, New York, United States of America; 6 The Integrative Science and Engineering Center, University of Dayton, 300 College Park, Dayton, Ohio, United States of America; University of California Davis, UNITED STATES

## Abstract

Animal traits develop through the expression and action of numerous regulatory and realizator genes that comprise a gene regulatory network (GRN). For each GRN, its underlying patterns of gene expression are controlled by *cis*-regulatory elements (CREs) that bind activating and repressing transcription factors. These interactions drive cell-type and developmental stage-specific transcriptional activation or repression. Most GRNs remain incompletely mapped, and a major barrier to this daunting task is CRE identification. Here, we used an *in silico* method to identify predicted CREs (pCREs) that comprise the GRN which governs sex-specific pigmentation of *Drosophila melanogaster*. Through *in vivo* assays, we demonstrate that many pCREs activate expression in the correct cell-type and developmental stage. We employed genome editing to demonstrate that two CREs control the pupal abdomen expression of *trithorax*, whose function is required for the dimorphic phenotype. Surprisingly, *trithorax* had no detectable effect on this GRN’s key *trans*-regulators, but shapes the sex-specific expression of two realizator genes. Comparison of sequences orthologous to these CREs supports an evolutionary scenario where these *trithorax* CREs predated the origin of the dimorphic trait. Collectively, this study demonstrates how *in silico* approaches can shed novel insights on the GRN basis for a trait’s development and evolution.

## Author summary

The traits decorating animal bodies form by patterns of gene expression that are controlled by *cis*-regulatory element sequences. These sequences behave like switches to turn genes on or off during development. The conservation and evolution of such switches are key aspects for many traits’ origin. Unfortunately, they remain difficult to find, and their regulated genes and activities remain unknown. Here, the authors focused on a derived pattern of fruit fly pigmentation. They used a small set of known switches to find hundreds of predicted similarly-functioning switches through a computer algorithm that finds patterns in DNA sequences. The authors focused on two predicted switches, and showed how they control the expression of an evolutionarily-conserved regulator of gene expression. In an interesting twist, this regulator does not repeat its embryonic function in the development of this adult trait. Rather it targets two genes that encode pigmentation enzymes. It was found that the expression and switch functions for this regulator pre-date the derived trait, indicating that the gain of these enzyme targets were key events in this trait’s origin. This study is noteworthy, as it shows how computer- and animal-based approaches can deepen our understanding of genetic switches in development and evolution.

## Introduction

The reigning genetic model for trait development involves the orchestrated expression of regulatory genes (such as transcription factors, coactivators, corepressors, and chromatin modifiers) that interact with the *cis*-regulatory elements (CREs) of their direct target genes. These interactions culminate in the expression of the realizator genes whose encoded RNAs and proteins make the characteristic phenotype [1–3]. These genes and their expression-controlling CREs embody a gene regulatory network (GRN). A central goal of evolutionary-developmental research is to elucidate the GRNs for traits and to understand how such GRNs were originally constructed, and subsequently diversified [4,5]. While there has been success in identifying some genes and their CREs for a variety of animal traits [6,7], it remains difficult to identify the breadth of CREs and regulated genes for any single trait, let alone to reconstruct the origins of GRNs in evolutionary history. This is largely due to the difficulty in identifying CREs with a specific regulatory activity within a vast genome, and subsequently connecting these to their target gene of regulation.

For various animal traits, some of their key genes and CREs have been elucidated, and in the best of cases one or a few of the interacting transcription factors are known. In order to find additional CREs, *in silico* approaches have been devised that can predict other similarly functioning CREs based upon the presence of a similar profile of transcription factor binding sites within a CRE-sized DNA sequence [8]. Unfortunately, though, for many CREs few if any of their interacting transcription factors are known, nullifying the utility of motif-based approaches. As an alternative, motif-blind approaches have been developed that rely solely on the sequences of a set of known similarly-functioning CREs for predicting new CREs [8,9]. Such *in silico* approaches offer an opportunity to better resolve the CRE breadth of partially-characterized GRNs, and create a wealth of opportunities to use *in vivo* methods to reveal the target genes for these new CREs, and to study their evolutionary histories.

**Fig 1:**
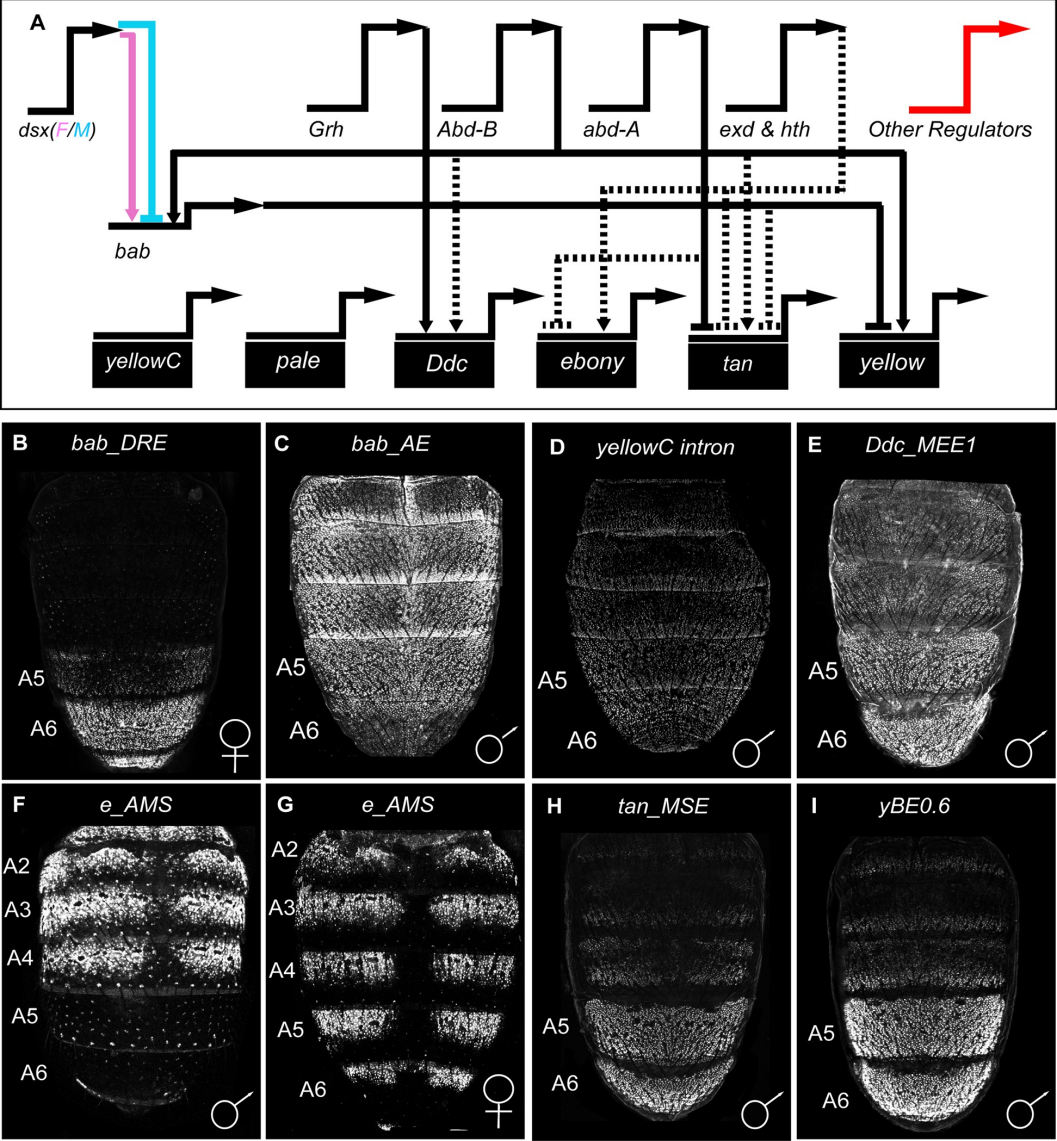
The known regulatory architecture and CREs for the *D*. *melanogaster* tergite pigmentation GRN. (A) Several *trans*-regulators are known that govern the expression of subordinate transcription factor genes, such as *bab1* and *bab2* (collectively referred to as *bab*) and the realizator genes that encode proteins involved in pigment metabolism (gene name on black colored rectangles). It is likely that other regulators remain unknown that shape spatial, temporal, sex-specific, and/or levels of gene expression. Direct interactions between a gene’s encoded transcription factor and a target gene’s CRE are indicated by solid line connections, whereas indirect interactions or interactions for which a CRE binding site remains unknown are shown as dashed line connections. Positive regulation is represented as an arrow head and repression as a nail head shape. (B-I) EGFP reporter expressions driven by CREs that regulate a pigmentation GRN gene *in vivo*. All EGFP expressions shown were captured at around 80–95 hours after puparium formation (Stages P13 to P14-15i), except for F and G which are shown at ~2 hours after eclosion. (B) The *bab dimorphic element* (or *DRE*) drives female-specific expression. (C-E) CREs that operate similarly in males and females but for which the male image is shown here for convenience. (F and G) EGFP expression driven by an *ebony* locus enhancer and silencer elements. (F) Notably, a silencer element represses reporter expression in the A5 and A6 segments of males. (H and I) The CREs for *tan* and *yellow* drive robust expression in the male A5 and A6 abdominal segments. These 8 CREs were used as a training set to find DNA sequences with similar motifs and that may be novel similar-functioning CREs.

The GRN responsible for the male-specific pigmentation on the posterior abdominal segments of *Drosophila melanogaster* presents an ideal test case for combining *in silico* and *in vivo* approaches to GRN analysis. The *Drosophila* abdomen is protected by hardened but flexible tergites that have segment-specific pigmentation phenotypes. In *D*. *melanogaster* and its close relatives, a fairly well-studied GRN controls dark coloration of the posterior-most segments (A5 and A6) in males but not females. This dimorphic GRN and its evolution have received considerable attention [10], and CREs have been identified for the *bric-à-brac* (*bab*) *trans*-regulators [11] and several of its realizator genes [12–15] (Fig 1). Moreover, varied data generally supports an evolutionary scenario in which this dimorphic phenotype is derived, originating in the lineage of *D*. *melanogaster* and closely-related species, after it diverged from lineages of species that possess the ancestral monomorphic phenotype, such as *D*. *willistoni* [16–18].

In this study, we used the motif-blind SCRMshaw tool [19–21] to predict hundreds of CREs based upon similarities to an original set of eight known CREs (Fig 1B–1I). The accuracy of these predictions was tested by reporter transgene assays. Successful predictions were included in a second round of SCRMshaw discovery that seemingly resulted in more accurate CRE prediction. We followed up on the function and evolution of two of the pCREs, revealing that they regulate the expression of the *trithorax* (*trx*) gene. *trx* encodes a protein known to function as a coactivator for a wealth of *D*. *melanogaster* genes, including the *Hox* gene *Abdominal-B* (*Abd-B*) [22]. Further, we unexpectedly show that Trx does not appear to regulate Abd-B or Bab function within this tergite pigmentation GRN, but rather regulates the expression of two realizator genes that are expressed in sex-specific patterns. The pigmentation GRN activity for these *trx* CREs appears to pre-date the origin of this dimorphic trait. Collectively, this work shows how *in silico* and *in vivo* approaches can be used in combination to further the evo-devo understanding of partially characterized GRNs.

## Results

### Identification of novel CREs by a motif-blind search for sequences similar to eight known *D*. *melanogaster* CREs

It is reasonable to suspect that the seven known and one suspected (*yellow C*) CREs that control the expression of genes within *D*. *melanogaster* tergite pigmentation GRN are an important but small subset of this GRN’s CREs. We sought additional CREs that shape the trait’s development. The DNA sequences for the eight known CREs (S1 Document) were used as “training set” input to search the *D*. *melanogaster* genome using the SCRMshaw (Supervised *c**is*-Regulatory Module) CRE discovery algorithm [19–21]. SCRMshaw uses a machine learning framework to compare the short DNA subsequence composition of a training set of known CREs with related function to that of a “background” set of non-CRE sequences; the trained model is then used to search the genome and predict additional related CREs. This analysis predicted 232 sequences to possess a similar CRE activity (referred to as pCREs for “predicted CREs”) and which are distributed in intronic or intergenic regions on the X, 2^nd^, and 3^rd^ *D*. *melanogaster* chromosomes (S1 Table).

**Fig 2:**
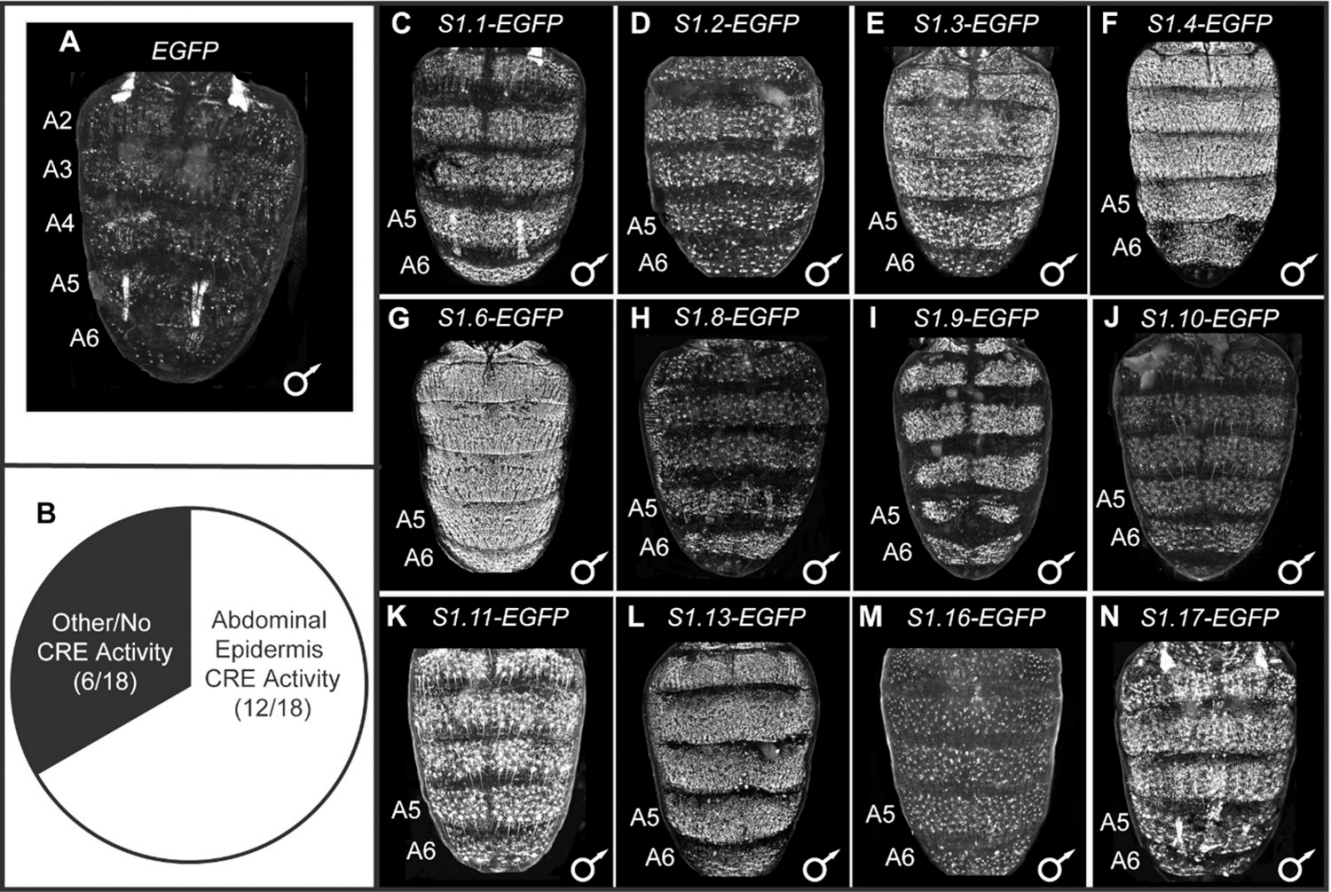
Reporter transgene test of 18 predicted CREs from the first dorsal pupal abdomen CRE training set. (A and C-N) *EGFP* reporter transgene expressions in the dorsal abdomens of male *D*. *melanogaster* pupa at ~80–88 hours after puparium formation (hAPF) or the P13-P14(i) developmental stage. Expressions in the female abdomens were similar and therefore not reported here. (A) In the absence of a CRE, EGFP expression was seen in some abdominal muscles, but little to no expression was seen in the abdominal epidermis. (B) Of the 18 pCREs tested by reporter transgene assays, twelve showed reproducible dorsal abdominal epidermis expression that exceeds what is observed in the (A) no CRE control. (C-N) Among the pCREs with abdominal expression regulatory activity, EGFP expression was generally seen throughout abdominal segments A2-A6. All reporter transgenes were inserted into the 51D *att*P site on the 2^nd^ chromosome, and therefore experience the same genomic and chromatin environments. (J) Brightness was increased in Adobe Photoshop by 25% compared to the other figure panels with EGFP expressions.

In order to assess the quality of the 232 pCREs, we selected 18 to test in reporter transgene assays (Table 1). These 18 were selected because of their proximity to genes encoding proteins involved in gene expression regulation or cuticle development. Each of the 18 sequences was cloned into the S3aG reporter transgene vector upstream of the *hsp70* promoter and the *Enhanced Green Fluorescent Protein* (*EGFP*) reporter gene [23]. Each reporter, as well as a negative control transgene that lacked a CRE, was individually integrated into the same 51D *att*P site on *D*. *melanogaster* chromosome 2R [24]. In the absence of a CRE, EGFP expression was observed in some abdominal muscles, likely due to trapping the regulatory activity of an endogenous CRE or CREs that reside nearby the *att*P insertion site (Fig 2A). Importantly though, little to no expression was seen in the abdominal epidermis where genes of the tergite pigmentation GRN are expressed during pupal or early adult stages. For the 18 evaluated pCREs (S1 Fig), 12 (~67%) showed notable and reproducible EGFP expression in the dorsal abdominal epidermis (Fig 2C–2N and S2 Fig).

**Table 1:** Pertinent information for the 18 SCRMshaw predicted CREs that were evaluated in *Drosophila melanogaster* reporter transgene assays.

predicted CRE (pCRE) Name	Gene Neighborhood	Coordinates	Method	~Score
***S1*.*1***	*HDAC4* intron 1	X:13284500–13285000	imm	13.4
***S1*.*2***	*e(y)3* intron 4	X:19631250–19631750	pac	0.41
***S1*.*3***	*Muc4B - CG43134* intergenic	X:4246250–4246750	hexmcd,imm	18.6
***S1*.*4***	*CG9650* intron 2	X:7213500–7214000	hexmcd,imm	19.3
*S1*.*5*	*mgl* intron 2	X:9389500–9390000	pac	0.42
***S1*.*6***	*Sp1—CG1354* Intergenic	X:9774000–9774750	imm	13.3
*S1*.*7*	*yellow C* intron 1	2L:15035500–15036500	hexmcd,imm	34.6
***S1*.*8***	Upstream of *Kr-h1*	2L:6082000–6082500	pac	0.43
***S1*.*9***	*twist* intron 1	2R:23005250–23005750	imm	10.1
***S1*.*10***	Upstream of *klu*	3L:11010000–11010750	hexmcd	21.2
***S1*.*11***	*Eip74EF* intron 2	3L:17601000–17601500	hexmcd	17.7
*S1*.*12*	*Pdp1* intron 2	3L:7833500–7834000	pac	0.41
***S1*.*13***	*GATAe–pnr* Intergenic	3R:16020250–16020750	pac	0.41
*S1*.*14*	*Abd-B* intron 1	3R:16949500–16950000	pac	0.42
*S1*.*15*	*Eip93F* intron 6	3R:22001250–22001750	hexmcd,imm	24.1
***S1*.*16***	*hh–unkempt* Intergenic	3R:23145000–23145500	pac	0.42
***S1*.*17***	*hdc* intron 2	3R:30337750–30338250	hexmcd,imm	17.9
*S1*.*18*	Downstream of *yellow-h*	4:231750–232500	imm	12.9

Note: pCREs with bolded font were those found to drive robust EGFP reporter expression in the developing pupal abdomen.

**Fig 3:**
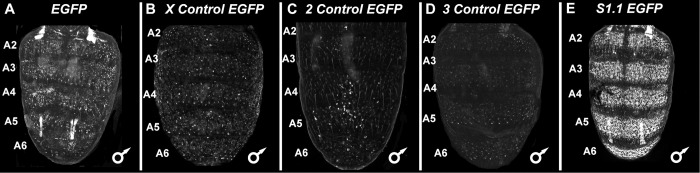
Non-coding sequences selected solely for the possession of similar nucleotide compositions to a specific training set CRE failed to drive noteworthy pupal abdominal epidermis expression. (A-E) *EGFP* reporter transgene expressions in the dorsal abdomens of male *D*. *melanogaster* pupa at ~80–88 hours after puparium formation (hAPF) or the P13-P14(i) developmental stage. Expressions in the female abdomens were similar and therefore not reported here. (A) EGFP expression pattern when the reporter transgene does not possess a CRE. (B) Expression driven by an X-chromosome non-coding sequence (X:7749947–7751734) with nucleotide composition equivalent to the *Dopa decarboxylase* Male Epidermis Element 1 or “*Ddc_MEE1*” CRE. (C) Expression driven by a 2^nd^ chromosome non-coding sequence (2L:11922488–11924519) with nucleotide composition equivalent to the *bric à brac* (*bab*) “*anterior element*” (ACEsub3 region) CRE. (D) Expression driven by a 3^rd^ chromosome non-coding sequence (3L:27532906–27534457) with nucleotide composition equivalent to the *ebony* activation element CRE. (E) EGFP expression driven by the *S1*.*1* CRE is shown to illustrate the activity of a sequence with the genuine pupal abdomen regulatory activity. 3A and 3E are redundant images to those used in Fig 2A and 2C respectively.

As a control, we randomly selected genomic sequences similar in length and nucleotide composition to each of the *Ddc_MEE1*, *ebony* activation element, and *bab Anterior Element* CREs (S2 Table), and tested them in the identical reporter gene assay. These sequences all lacked enhancer activity in the pupal abdominal epidermis (Fig 3B–3D). We further tested a set of 14 randomly-selected (no *a priori* criteria used in the selections from the large collection of GAL4 lines) reporter gene lines from the FlyLight collection [25] (S3 Table) for their ability to function as pupal abdominal epidermis CREs. None of the 14 displayed noteworthy expression (S3 Fig). Collectively, these control experiments support the conclusion that SCRMshaw was specifically able to leverage information encoded in just eight CREs to identify with reasonable accuracy DNA sequences with similar regulatory activity.

### An enlarged training set of CREs improved the breadth and efficacy of pupal abdomen CRE identification by SCRMshaw

Six of the tested pCREs lacked compelling abdomen expression (S1 and S2 Figs). It is possible that some of these sequences function as silencers, and therefore their activity could not be observed in the reporter transgene assays. However, we note that the SCRMshaw training set we used, consisting of only eight CREs, is significantly smaller than the recommended training set size of 20–30 sequences [9]. These six pCREs may therefore simply represent false-positive predictions, putting the false-positive rate somewhere around 33%. We therefore generated a revised training set by removing two of the original eight sequences—the *ebony* silencer and the *yellow-c* intron (which has not been shown to participate in the tergite pigmentation GRN) and adding 10 of the newly-identified CREs with abdominal epidermis activity (Fig 2). We used this updated training set, now consisting of 16 sequences, (S2 Document) to again screen the *D*. *melanogaster* genome by SCRMshaw for pCREs. We identified over 700 pCREs (S4 Table). Importantly, these pCREs included ten of the twelve pCREs from the original SCRMshaw round that were successfully validated, and only one of the six (*S1*.*15*) that failed to drive reporter gene expression.

**Fig 4:**
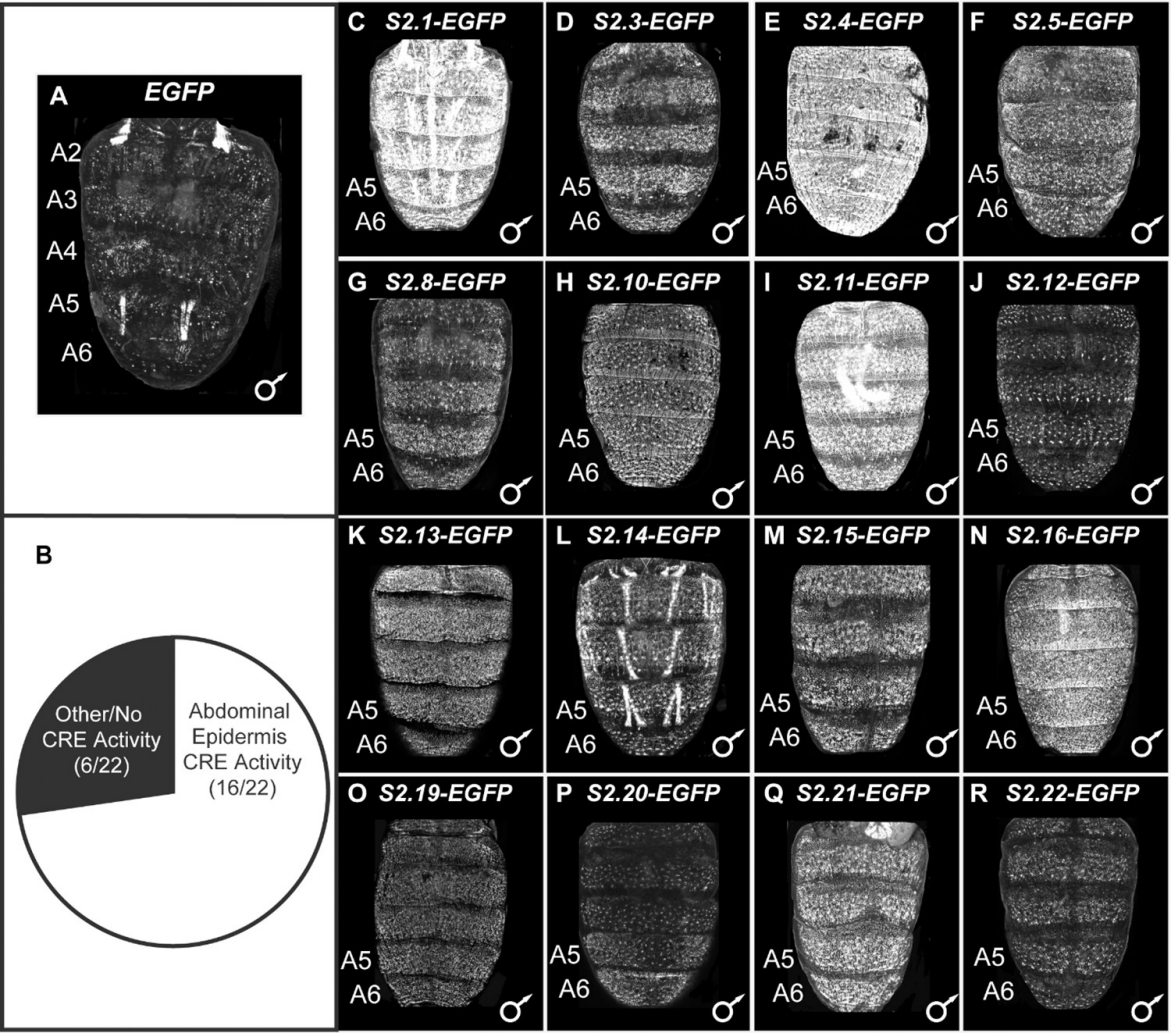
Reporter transgene tests of 22 predicted *cis*-regulatory elements from the second CRE training set. (A and C-R) *EGFP* reporter transgene expressions in the dorsal abdomens of male *D*. *melanogaster* pupa at ~80–88 hours after puparium formation (hAPF) or the P13-P14(i) developmental stage. Expressions in the female abdomens were similar and therefore not reported here. (A) In the absence of a CRE, EGFP expression was seen in some abdominal muscles, but little to no expression was seen in the abdominal epidermis. (B) Of the 22 pCREs tested by reporter transgene assays, 16 showed reproducible abdominal epidermis expression that exceeds what is observed in the no CRE control (A). (C-R) Among the pCREs with abdominal expression regulatory activity, EGFP expression was generally seen throughout abdominal segments A2-A6. All reporter transgenes were inserted into the 51D *att*P site on the 2^nd^ chromosome, and experience the same genomic and chromatin environments. 4A is a redundant image to those used in Fig 2A and 3A.

In order to estimate the quality of the predictions from this second SCRMshaw round, we tested 22 pCREs (Table 2) for relevant enhancer activity in reporter transgene assays (Figs 4 and S4). These pCREs were selected for their proximity to genes encoding proteins involved in gene expression regulation, and genes that are in close proximity to multiple pCREs, like *Hr4*, *sbb*, and *trithorax* (*trx*). Of these 22 pCREs, 16 (73%) drove robust expression of the *EGFP* reporter transgene in the dorsal pupal abdomen at time points when the known pigmentation CREs are active (Fig 4B). Among the ~700 pCREs (S4 Table), we noticed several situations where multiple pCREs resided within or near genes that have been previously implicated to be involved in the *D*. *melanogaster* pigmentation GRN. Two such examples are *Hr4* and *sbb*, whose functions were shown to be necessary for the formation of melanic tergites [26]. Of the five pCREs that reside in an *Hr4* intron, four had robust abdominal enhancer activity (Fig 4C–4F). Of the three pCREs that reside in the 1^st^ intron of *sbb*, each drove reporter expression in the pupal abdominal epidermis (Fig 4J–4L). These results suggest that SCRMshaw can identify cases of redundant (“shadow”) CREs. Based on these results, we estimate that roughly three-quarters of the pCREs have enhancer activity in the pupal abdominal epidermis, a substantial improvement from our first round of predictions using the smaller training set (S5 Table). Since we did not evaluate a random sample of 22 pCREs, this estimation may be on the high end. Some, or perhaps many, of these may be CREs that regulate genes involved in the GRN responsible for the *D*. *melanogaster* abdomen pigmentation pattern.

**Table 2:** Pertinent information for the 22 SCRMshaw predicted CREs that were evaluated in *Drosophila melanogaster* reporter transgene assays.

predicted CRE (pCRE) Name	Gene Neighborhood	Coordinates	Method	~Score
***S2*.*1***	*Hr4* intron 3	X:1947150–1947800	imm	19.9
*S2*.*2*	*Hr4* intron 3	X:1965990–1966710	imm	30.8
***S2*.*3***	*Hr4* intron 4	X:1975750–1976540	imm	30.9
***S2*.*4***	*Hr4* intron 6	X:1989990–1990810	imm	29.9
***S2*.*5***	*Hr4* intron 6	X:1991560–1992580	imm	32.8
*S2*.*6*	*mgl* intron 2	X:9428350–9429020	imm	19.7
*S2*.*7*	*mgl* intron 2	X:9467850–9468580	imm	25.5
***S2*.*8***	*mgl* intron 1	X:9495830–9496730	imm	33.8
*S2*.*9*	*mmy* intron 1	2L:6473220–6473810	imm	17.9
***S2*.*10***	*tou* intron 1	2R:11596760–11597560	imm	26.1
***S2*.*11***	*tou* intron 1	2R:11604520–11605020	imm	16.6
***S2*.*12***	*sbb* intron 1	2R:18306580–18307470	imm	24.6
***S2*.*13***	*sbb* intron 1	2R:18310870–18311370	imm	15.8
***S2*.*14***	*sbb* intron 1	2R:18333120–18333620	imm	16.1
***S2*.*15***	*pdm3* upstream region	2R:8326770–8327720	imm	30.5
***S2*.*16***	*ect* intron 1	3L:10196740–10197660	imm	27.8
*S2*.*17*	*vvl–CR45115* intergenic region	3L:6832930–6833560	imm	21.0
*S2*.*18*	*Gug* intron 1	3L:8450420–8450970	imm	17.9
***S2*.*19***	*trx* intron 1	3R:14278540–14279370	imm	30.6
***S2*.*20***	*trx* intron 1	3R:14284920–14285770	imm	25.4
***S2*.*21***	*corto* upstream region	3R:5086870–5087550	imm	19.4
***S2*.*22***	*corto* upstream region	3R:5087760–5088450	imm	27.4

Note: pCREs with bolded font were those found to drive robust EGFP reporter expression in the developing pupal abdomen.

### Two pCREs additively regulate the activity of the *trithorax* gene and are required for *D*. *melanogaster* tergite pigmentation patterning

**Fig 5:**
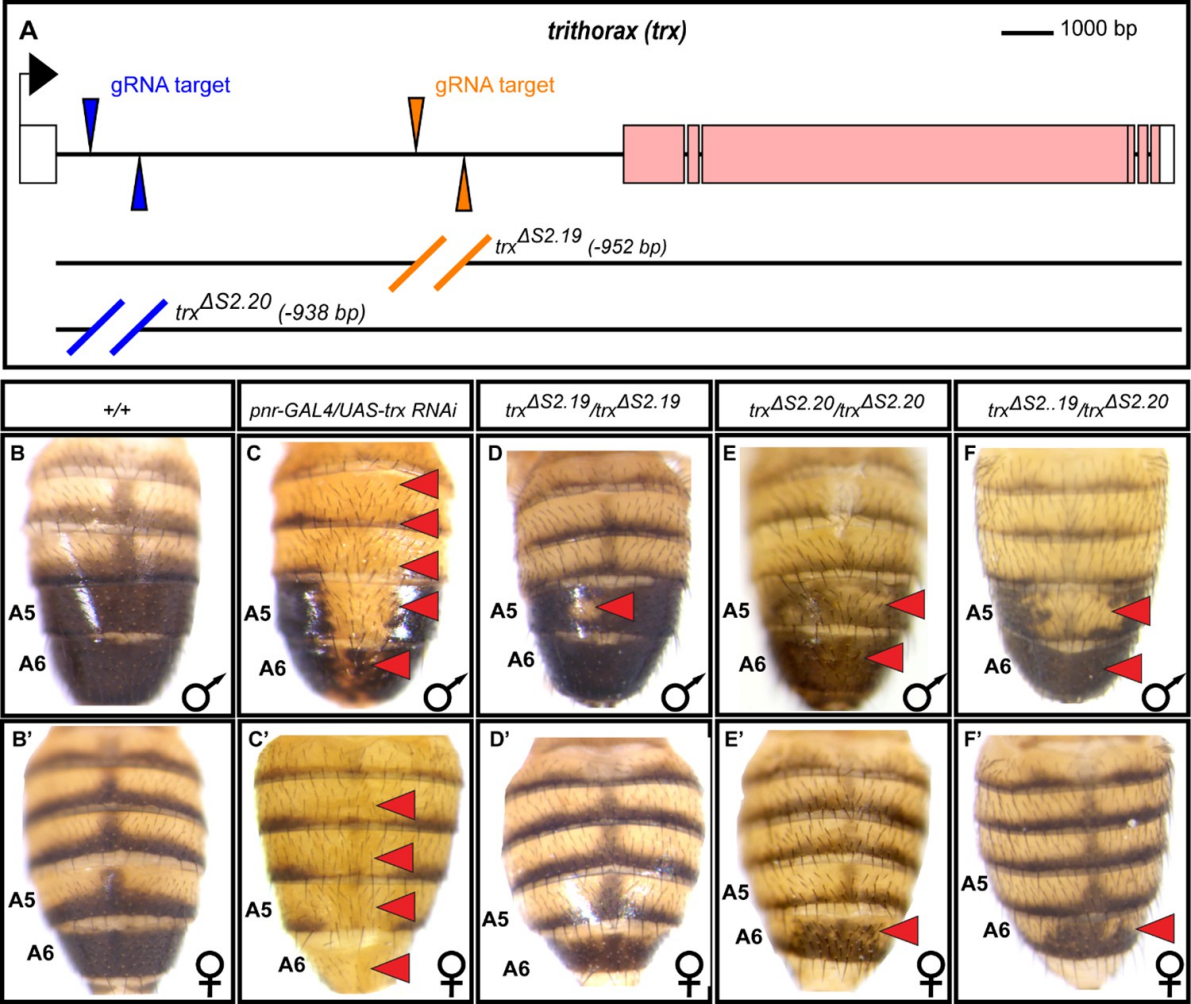
*trithorax* function and its intronic CREs are necessary for the *D*. *melanogaster* pigmentation pattern. (A) To-scale annotated representation of the *trithorax* locus. Black arrow indicates the site and direction of transcription initiation. Exons are the large rectangles, and those coding for the Trx protein are colored pink. The position of the *S2*.*19* and *S2*.*20* pCREs each lie between a pair of guide RNA (gRNA) target sites that were used to create the CRE deletion alleles by CRISPR/Cas9-mediated genome editing. The wild type pattern of (B) male and (B’) female tergite pigmentation from the *w*^*1118*^ stock. Suppression of *trx* expression in the dorsal midline region by RNA interference (RNAi) resulted in lost tergite pigmentation in (C) males and (C’) females. When the *S2*.*19* sequence was deleted (D) tergite pigmentation was reduced in the male A5 segment, whereas (D’) female pigmentation appeared wild type. When the *S2*.*20* sequence was deleted, (E) tergite pigmentation was reduced on the male A5 segment tergite and to a lesser extent in the A6 segment, while in females (E’) A6 tergite pigmentation was subtly reduced. In *trx trans*-heterozygous (F) males and (F’) females, tergite pigmentation is further reduced in the male A5 tergite, and subtly in the male and female A6 tergites. Red arrowheads point to tergites that have reduced melanic pigmentation compared to the wild type (*+/+*) control.

In order to properly situate some of the newly discovered CREs within the pigmentation GRN, we chose to investigate two CREs that reside in the large first intron of the *trithorax* (*trx*) gene (Fig 5A). These were two of the 22 CREs analyzed in *D*. *melanogaster* reporter transgene assays, and shown to drive EGFP expression in the pupal abdominal epidermis (Fig 4O and 4P). *trx* is a well-known regulator of *Hox* genes [22], including *Abd-B*, at early stages of *D*. *melanogaster* development, and encodes a protein which is an evolutionarily conserved chromatin remodeler [27]. We expressed a UAS-regulated RNA-interference (RNAi) transgene in the dorsal midline region by the expression of GAL4 in the endogenous domain of *pannier* (*pnr*) expression [28]. The expressed hairpin targets *trx* and thereby reduces *trx* expression. We observed a dramatic loss of melanic tergite pigmentation (compare Fig 5C and 5C’ to the wild type control in 5B and 5B’). This outcome indicates that *trx* acts within the tergite pigmentation GRN to promote formation of the melanic phenotype.

We were curious whether the newly identified *S2*.*19* and *S2*.*20* CREs are required for endogenous *trx* expression and for the development of the normal tergite pigmentation pattern. In order to test this, we used pairs of guide RNAs targeting sequences flanking the *S2*.*19* and separately the *S2*.*20* CRE sequences (Fig 5A and S6 Table). These were used to introduce double stranded breaks by Cas9, allowing for homology dependent repair to remove the CRE and replace it with a *loxP* site flanked cassette possessing the *EGFP* gene under the regulation of the 3XP3 eye enhancer. This cassette was secondarily deleted by the activity of Cre recombinase. In contrast to the wild type dimorphic pattern of tergite pigmentation in the control strain (Fig 5B and 5B’), when the *S2*.*19* CRE was removed (*trx*^*ΔS2*.*19*^/*trx*^*ΔS2*.*19*^), melanic pigmentation was subtly reduced in the male A5 tergite (Fig 5D). When the *S2*.*20* CRE was removed (*trx*^*ΔS2*.*20*^/*trx*^*ΔS2*.*20*^), pigmentation was notably reduced in the male A5 tergite, and to a lesser extent in the A6 tergite (Fig 5E). In females, the absence of the *S2*.*20* CRE occasionally resulted in a subtle decrease in A6 tergite pigmentation (Fig 5E’, red arrowhead). Tergite pigmentation was further reduced in *trans*-heterozygous (*trx*^*ΔS2*.*19*^/*trx*^*ΔS2*.*20*^) males and females (Fig 5F and 5F’). These male and female CRE deletion phenotypes were consistent across replicate specimens of the same genotype (S5 Fig). Though milder, these CRE deletion phenotypes resemble that seen from RNAi, indicating that the *S2*.*19* and *S2*.*20* CREs both regulate *trx* expression in the abdominal epidermis.

### The pigmentation GRN function of Abd-B and Bab appears to be independent of Trx

**Fig 6:**
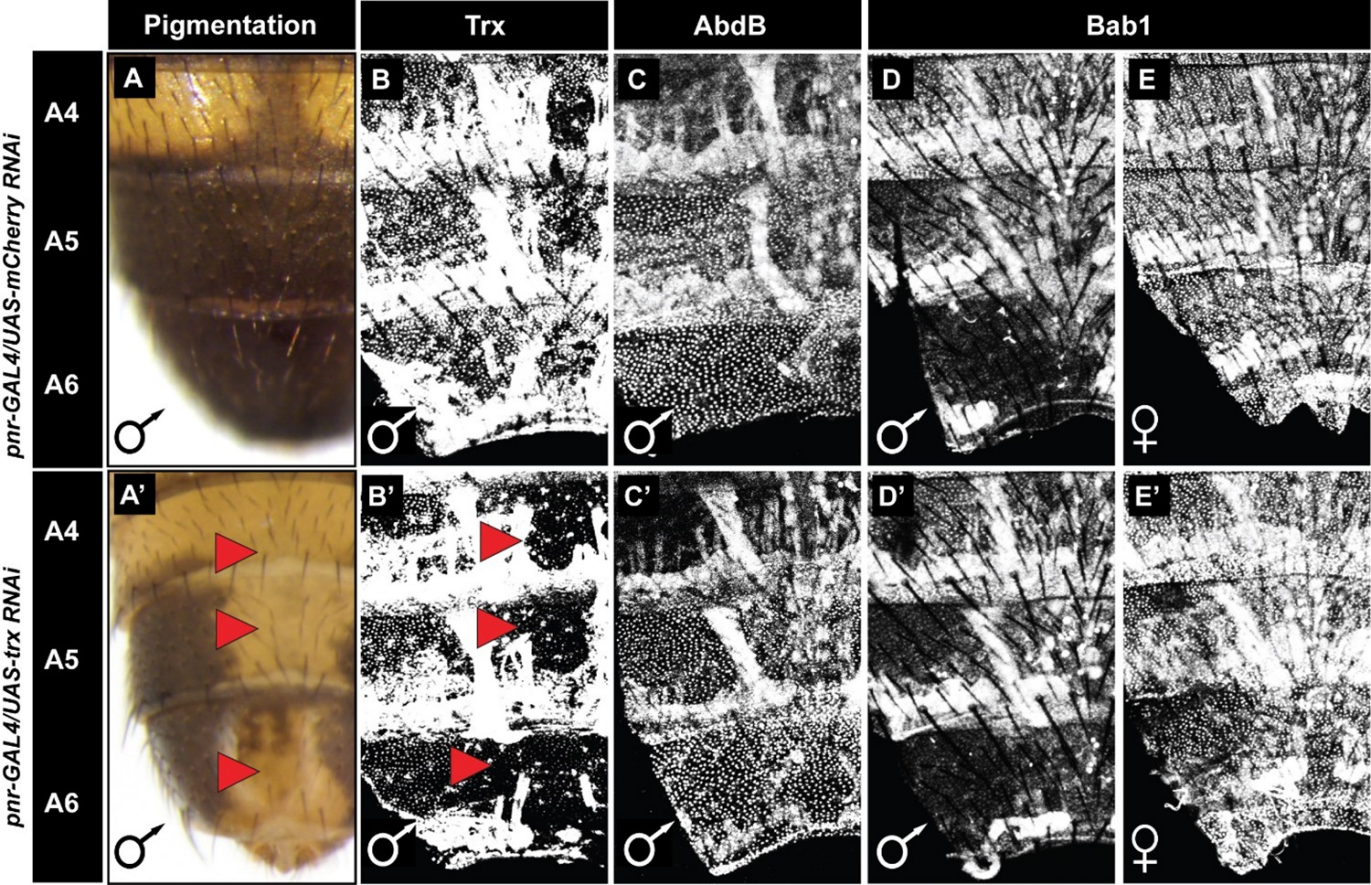
Reduced Trithorax expression has little to no effect on pupal abdominal expression of Abd-B and Bab1. (A-E) Specimens for which RNAi targeted the negative control *mCherry* gene in the dorsal abdomen midline region. (A’-E’) Specimens for which RNAi targeted the *trithorax* gene in the dorsal abdomen midline region. (A) While *mCherry* RNAi had no effect on adult male tergite pigmentation, (A’) a conspicuous loss of pigmentation occurred following RNAi for *trx*. (B) While *mCherry* RNAi had no effect on Trx protein expression in the abdominal epidermis, (B’) a conspicuous loss of Trx expression was seen in the midline region in response to *trx* RNAi. Neither (C) *mCherry* RNAi nor (C’) *trx* RNAi had a noticeable effect on the A5 and A6 segment expression of Abd-B. In addition to the expected Abd-B nuclear expression, in these specimens some non-specific expression was observed in muscle tissue. Neither (D and E) *mCherry* RNAi nor (D’ and E’) *trx* RNAi caused a noticeable difference in the expression of Bab1. Red arrowheads point to abdomen regions where pigmentation or protein expression was altered compared to the control.

Abd-B and Bab are two of the most prominent *trans*-regulators within the *D*. *melanogaster* tergite pigmentation GRN (Fig 1A). The male A5 and A6 tergite pigmentation of *Abd-B* null mutants resembles the non-melanic appearance of con-specific females [17]. Thus, we thought Trx might function as a positive regulator of Abd-B A5 and A6 segment expression during the pupal time frame of pigmentation patterning (Fig 6). However, surprisingly, while RNAi for *trx* in the dorsal midline regions resulted in losses in male tergite pigmentation and Trx protein expression (Fig 6A’ and 6B’), little to no change was observed for Abd-B expression (Figs 6C’ and S6). Thus, it seems unlikely that Trx impacts this GRN at the level of Abd-B regulation.

**Fig 7:**
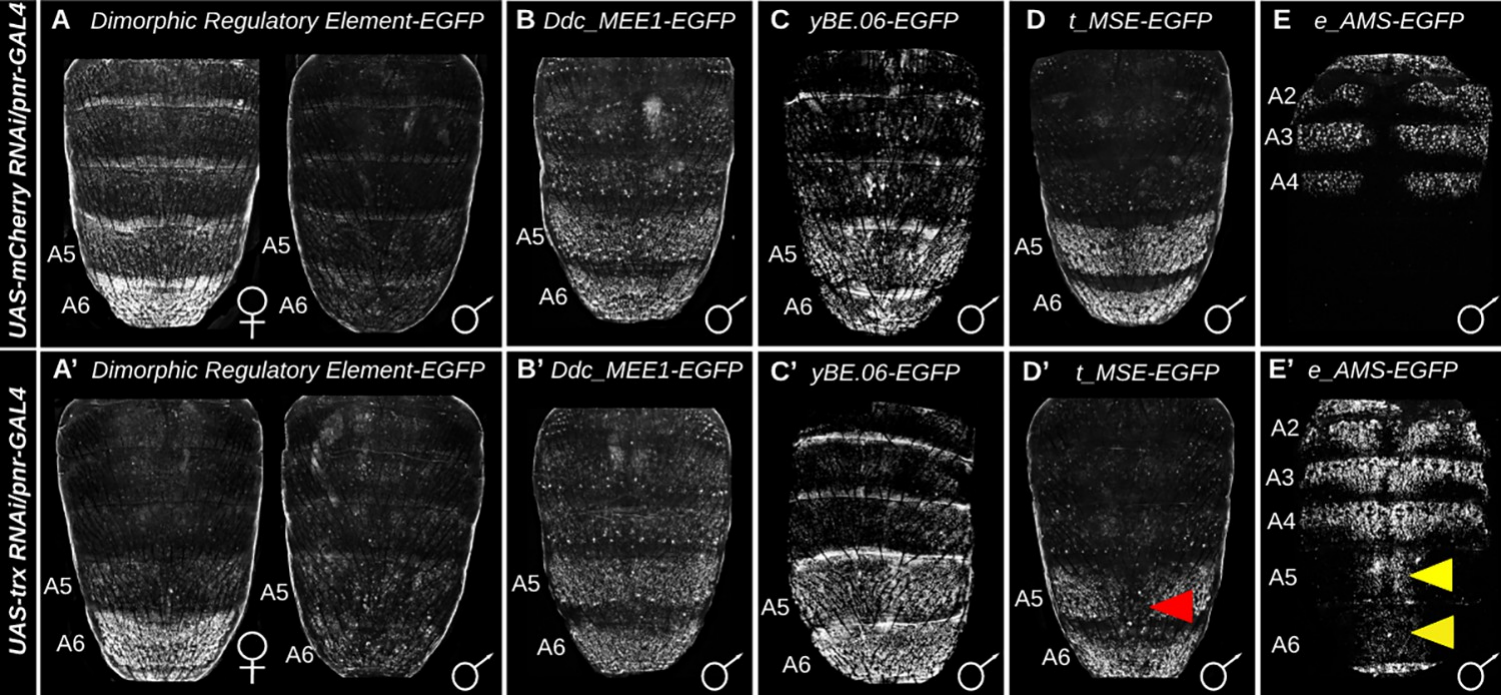
Tests for genetic interactions show that *trithorax* specifically regulates the expression of some pigmentation pathway genes. EGFP reporter transgene expression driven by CREs in genetic backgrounds where RNAi was performed in the midline region (A–E) for the negative control *mCherry* gene and (A’-E’) for *trithorax*. EGFP expression driven by the (A and A’) *bab dimorphic element*, (B and B’) *Ddc_MEE1*, and (C and C’) *yBE0*.*6* were taken at the P13-14(i) developmental stage. EGFP expression driven by the (D and D’) *t_MSE* was recorded at the P14-15(i) stage, and (E and E’) *e_AMS* CRE’s activity were recorded at 2 hours after eclosion. All specimens are hemizygous for the *EGFP* reporter transgene. Yellow arrowheads indicate segments where the genetic background alterations resulted in conspicuous ectopic EGFP reporter expression. Red arrowheads indicate segments where the genetic background alterations resulted in a reduced EGFP reporter expression.

It is also known that the development of melanic male A5 and A6 tergites requires Bab expression to be greatly down-regulated in these segments compared to females [17,29]. Moreover, ectopic Bab expression in males is sufficient to suppress melanic pigmentation of the A5 and A6 tergites [29]. Thus, it seemed plausible that Trx might function in some manner to keep Bab expression off in the male abdomen during pigmentation patterning. However, we saw no noteworthy increase in abdominal epidermis expression of Bab1 in males when *trx* expression was down-regulated by RNAi in the dorsal midline region (Figs 6D’ and S7). In fact, expression was no different than when RNAi targeted *mCherry*, a gene that does not exist in the *D*. *melanogaster* genome (Figs 6D and S7). We similarly analyzed the activity of the *bab* dimorphic element CRE in a reporter transgene assay in pupae with RNAi-suppressed *trx* expression in the midline region. Consistent with the Bab1 protein expression (Fig 6D’), here we did not observe ectopic EGFP reporter expression in the male abdomen nor reduced expression in the female abdomen (compare Figs 7A’ to the *mCherry* RNAi control in 7A, and S8). Thus, whatever important role Trx plays in this species’ pigmentation patterning, it does not noticeably impact the GRN’s most well-known *trans*-regulators.

### Trithorax regulates the expression of two pigmentation genes whose expression is sexually dimorphic

To find potential targets of Trx regulation, we tested the activity of pigmentation GRN CREs in the genetic background where *trx* expression was suppressed in the dorsal midline by RNAi (Fig 7). RNAi-reduced *trx* expression had no apparent effect on the activity of the *Ddc_MEE1* (compare Figs 7B’ to the control 7B, and replicates in S9) or yBE0.6 CREs (compare Figs 7C’ to 7C, and replicates in S10), indicating that Trx regulation has no influence on these CREs that respectively control expression of the pigmentation enzyme genes *Dopa Decarboxylase* and *yellow*. However, reduced *trx* expression did result in a reduction of the male-specific regulatory activities for the t_MSE CRE that activates the expression of *tan* in the male A5 and A6 segments (compare Figs 7D’ to the control 7D, and replicates in S11). However, no noteworthy change in t_MSE activity was observed in females when *trx* expression was reduced (S12 Fig). *trx* was previously shown to regulate female pigmentation, and that at low temperatures in young adult females *trx* is required for high levels of *tan* expression and the deposition of H3K4me3 at the *tan* promoter region [30]. This discrepancy is likely due to the specimens in this study being reared at temperatures at which there is little to no *tan* expression and melanic pigmentation in females.

Interestingly, reduced *trx* expression also resulted in a gain of EGFP expression in the male A5 and A6 segments when under the combined regulatory control of the *ebony* activation and silencer CREs (referred to as the e_AMS) [15] (compare Figs 7E’ to the control 7E and replicates in S13). A similar increase in expression was observed in the female abdomen (S14 Fig). *ebony* expression in these segments is normally repressed by the activity of a CRE that functions as a male-specific silencer and CRE that acts as silencer in the posterior region of male and female abdomen segments [15]. These results suggest that Trx normally facilitates the activity of either or both of the male-specific silencer and the posterior stripe silencer element. Furthermore, we show that while *trx* is not expressed in a sex-specific manner, it is a necessary factor in the expression regulation of two of this GRN’s realizator genes that normally exhibit sex-specific regulation; *tan*, and *ebony*.

### The evolutionary history of the *trithorax cis*-regulatory elements

**Fig 8:**
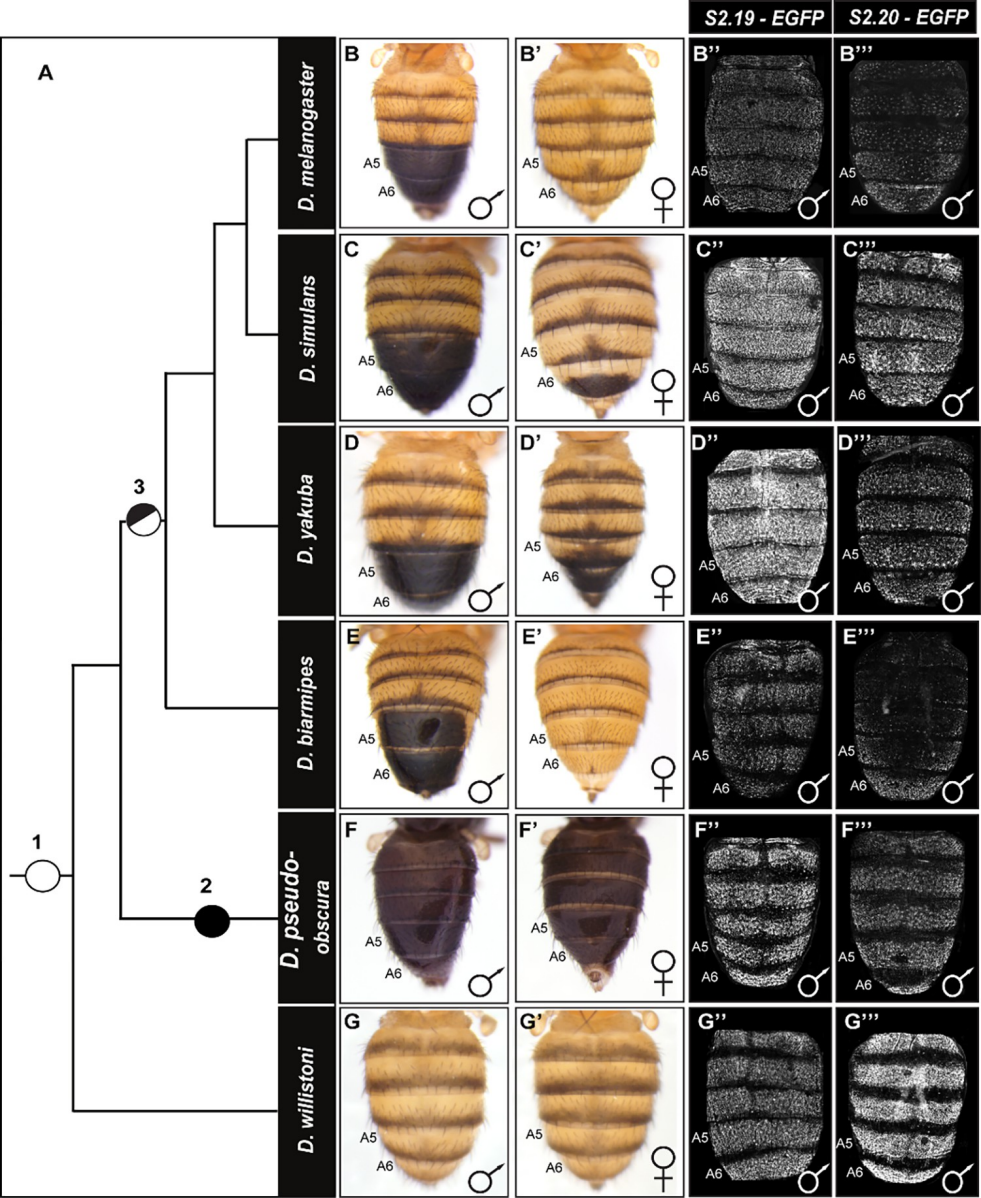
The evolution of tergite pigmentation and *trx* CRE activity in the *D*. *melanogaster* lineage. (A) Phylogeny showing the relationship of various *Sophophora* species with nodes indicating the origin of male-specific tergite pigmentation, and the placement of ancestral pigmentation phenotypes. It is generally accepted that the ancestor at nodes 1 and 2 had monomorphic tergite pigmentation patterns, with the node 2 ancestor being broadly melanic in color. Node 3 indicates the origin of sexually dimorphic pigmentation, where male tergites were fully melanic in color. Adult tergite pigmentation phenotypes for representative (B-G) males and (B’-G’) females. EGFP reporter expression patterns in transgenic *D*. *melanogaster* that were driven by sequences orthologous to the (B”-G”) *S2*.*19* and (B”‘-G”‘) *S2*.*20* CREs of *D*. *melanogaster*. The EGFP expression patterns demonstrate the conservation of ancestral pupal abdomen epidermis regulatory activities. However, some differences are seen among the orthologous sequences in the pattern and intensity of EGFP reporter expressions.

Male-specific tergite pigmentation is a derived phenotype that evolved in the lineage of *D*. *melanogaster* and closely-related species after they diverged from the monomorphic lineages of *D*. *willistoni* and *D*. *pseudoobscura* (Fig 8). We were curious whether the *trx S2*.*19* and *S2*.*20* CREs that drive non-sex-specific abdominal expression were available at the time when the sexually dimorphic GRN evolved. Thus, we cloned the orthologous sequences from dimorphic species closely related to *D*. *melanogaster* (*D*. *simulans*, *D*. *yakuba*, and *D*. *biarmipes*) and monomorphic species *D*. *pseudoobscura* and *D*. *willistoni* that hail from ancestrally monomorphic lineages (S15A Fig). For *S2*.*19*, the orthologous sequences from the dimorphic and monomorphic pigmentated species alike each possessed regulatory activity in the abdominal epidermis (Fig 8B”–8G”).

For the orthologous *S2*.*20* sequences, we generally observed a similar conserved regulatory activity in the abdominal epidermis (Fig 8B”‘–8G”‘). The conserved abdomen activities for these two CREs suggest that pupal abdomen expression of Trx pre-dated the origin of this dimorphic trait. Consistent with this interpretation, we observed similar abdomen patterns of Trx expression in the dorsal pupal abdomen epidermis of *D*. *melanogaster* and *D*. *willistoni* (S16 Fig).

To better understand how the regulatory activity is partitioned among the *S2*.*20* region of *D*. *melanogaster*, we tested the regulatory activity of truncated versions in reporter transgene assays (S15B Fig). Somewhat surprisingly, we were unable to further narrow the region harboring the abdominal epidermis activity below its 938 base pair size without the concomitant occurrence of ectopic reporter activity (S15C–S15K Fig). Future studies are need to determine the location of key binding sites and the identities of their interacting transcription factors.

## Discussion

**Fig 9:**
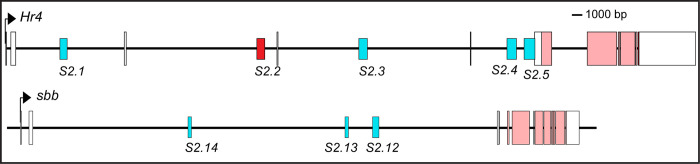
This study identified several *trans*-regulatory genes that harbor multiple pCREs. Like *trx*, SCRMshaw identified multiple pCREs within the *Hr4* and *sbb* loci. The short red rectangle indicates a pCRE found to lack noteworthy abdomen epidermis activity, whereas blue short rectangles indicate pCREs with demonstrated abdomen epidermis regulatory activity.

Here, we show how iterative application of the SCRMshaw computational tool, coupled with *in vivo* validation, was able to leverage an initial small set of CREs for the abdomen pigmentation GRN of *D*. *melanogaster* to enable high-accuracy CRE prediction, resulting in the identification of hundreds of predicted CREs (pCREs). A subset (biased towards those residing near compelling genes) of these were tested *in vivo* for enhancer activity characteristic of the GRN, with roughly three-quarters activating reporter gene expression in the abdomen epidermis during the timeframe when tergite cuticle pigmentation is patterned. We investigated two predicted CREs that reside in the large first intron of the *trithorax* (*trx*) gene, and show that these CREs act semi-redundantly in promoting the melanic phenotype characteristic of males. Although *trx* is a known positive regulator of the *Hox* gene *Abdominal-B* (*Abd-B*) during embryonic and larval development, this relationship does not appear to be a part of this later-acting abdominal pigmentation GRN. *trx* instead directly or indirectly regulates the sexually dimorphic expression of the *ebony* and *tan* realizator genes, whose encoded proteins work antagonistically in the formation of pigment molecules. These *trx* CREs were found to predate the origin of the dimorphic pigmentation in the lineage of *D*. *melanogaster*. Collectively, these results show how known CRE sequences can be used to find additional CREs *in silico*, and how doing so may illuminate novel aspects of a GRN. The findings suggest that much of the CRE landscape for the fruit fly pupal abdomen remains to be characterized, and for several genes, like *trx*, their *in vivo* pattern of expression may be under the control of an assembly of separate modular CREs (Fig 9).

### Insights on the genomic landscape of gene expression regulation

While the genetic composition and CRE-connectivity has been well-resolved for a few morphology-governing GRNs [3,31,32], most GRNs remain incompletely characterized to some extent. Thus, an appreciation for the gene and CRE rosters, their regulatory connections, and evolutionary history remain an important goal for the field of evolutionary-developmental biology. The male-specific posterior abdomen pigmentation GRN, though well studied, is certainly not comprehensively mapped [10]. However, beyond a small number of case studies lies the daunting task to uncover the rest of this GRN and tease out how it originated.

In this study, we sought to see whether there are some shared encodings (perhaps binding sites for the same transcription factors) within a small set of known CREs that could be extracted by the SCRMshaw tool and used to predict additional CREs that might connect additional genes into this GRN. An initial small set of 8 CREs proved to be sufficient for SCRMshaw to identify hundreds of pCREs, albeit with a moderately high false-positive prediction rate. We used the results of *in vivo* validation experiments from this first round of predictions to construct an improved training set with a substantially higher true-positive rate (S5 Table). Thus, even when only a limited number of known CREs are available for training, *in vivo* validation coupled with an iterative searching strategy enables SCRMshaw to be used for high-accuracy CRE prediction. It is important to reiterate that our tests of prediction accuracy were not based on tests of random selections of pCREs. Thus, it is possible that the true-positive estimates may be somewhat inflated, and significantly more *in vivo* testing would be needed to get a stronger handle on the false-positive rates.

We estimate that as many as three-quarters of our predictions—over 550—may act as enhancers in the pupal abdomen epidermis of transgenic *D*. *melanogaster*. It remains to be determined what percentage of these pCREs act not just in the abdomen but in the tergite pigmentation GRN. However, even if that fraction is as low as 10%, we still will have identified dozens or more genes and their expression-controlling CREs that now must be placed within the GRN and have their ancestral states resolved. Doing so will be integral to a comprehensive understanding of GRN assembly and diversification that this system has been so influential in establishing.

Among the wealth of pCREs, several examples were found where multiple pCREs reside in the sequence surrounding the same gene. This includes two for *trx* that were characterized in this study. Other notable examples include *Hr4* and *sbb* (Fig 9). These two genes encode transcription factors, and a previous study showed that each is necessary for the development of the melanic A5 and A6 tergite phenotype of *D*. *melanogaster* males [26]. Multiple pCREs were identified in the *hth* locus, which encodes a Hox co-factor that is a known direct regulator of the t_MSE CRE [14]. Likewise, several pCREs reside with the *bab* locus, suggesting the temporal, sex-specific, and quantitative levels of Bab1 and Bab2 expression are under control of more CREs than currently appreciated. These examples raise the possibility that several key *trans*-regulators of this GRN have their expression controlled by a landscape of CREs similar to what has been observed in the regulation of tetrapod *Hox* genes [33,34], the butterfly *WntA* gene [35], and *svb* of fruit flies [36,37]. In addition to these known regulatory genes of the pigmentation GRN having multiple pCREs, the roster of pCREs (S4 Table) is flush with occurrences of multiple pCREs per gene, including 18 for the *mamo* locus. Looking forward, this model system might be ripe with opportunities to explore how complex regulatory landscapes originate.

### A novel function and origin for *trithorax* in a late developing morphological trait

Of all the genes close to a pCRE, *trx* caught our attention for several reasons. The Trithorax protein has been found in various functional complexes that regulate gene expression [38,39]. *trx* functions to sustain the expression of several *Hox* genes, including *Abd-B* in the posterior embryonic abdomen [22], and is the founding member of the Trithorax Group (TrxG) of proteins, which function as transcriptional activators and counteract the repressive effects on chromatin structure of the Polycomb Group proteins [27]. *Abd-B* regulates multiple downstream genes critical to pigmentation, *tan* and *yellow* realizator genes [14,18], and the “dimorphic element” CRE that activates Bab1 and Bab2 expression in the female abdomen [11] which then repress the expression of *yellow* and *tan* [13,14]. This prior knowledge suggested that Trx would be a key factor driving the expression of Abd-B in the epidermis of the A5 and A6 pupal segments. To our surprise, reduced Trx expression had no noticeable impact on Abd-B expression during pigmentation patterning, suggesting that these key proteins play independent roles in the tergite pigmentation GRN, or that the order of regulation has been reversed.

Our data also indicate that Bab regulation in the pupal abdomen is independent of *trithorax*, as reduced *trx* expression has no noticeable effect on Bab1 expression or on the activity of its dimorphic element CRE. While reduced *trx* expression did not seem to alter the activity of the CREs controlling *Ddc* and *yellow* expression in the A5 and A6 segments, we observed reduced activity of the CREs controlling *tan* expression, and ectopic expression from the activator and silencer CREs that control the dimorphic pattern of *ebony* expression. These results indicate that *trx* does not play a generic positive role on gene expression in the pupal abdomen, but rather has a narrowly-focused function in the pigmentation GRN to shape the expression of two terminal-acting realizators with reciprocal patterns of sex-specific expression.

These results make evident the need for future studies to address how Trx facilitates repression of *ebony* in the A5 and A6 segments and the activation of *tan* in these same male segments. This might prove to be a daunting challenge, as TrxG proteins are known to regulate gene expression through biochemically diverse mechanisms [38,39]. Here, we investigated the influence that reduced *trx* has upon CREs for five different genes, each that shared the same heterologous promoter regions. The limitation of effects to the transgenes with *ebony* and *tan* CREs, suggests that regulation is not occurring through the promoter region, but rather targeted to a CRE sequence, perhaps through Trx interacting with CRE-bound transcription factors and its recruitment of the powerful CBP coactivator to the *ebony* and *tan* CREs. However, it is also possible that *ebony* and/or *tan* regulation may be indirect, through Trx regulating the expression of a key activator (*tan*) or repressor (*ebony*) for these realizator genes. Further studies in this evo-devo model present an opportunity to resolve how Trx acquired two new realizator target genes during the origin of a novel pigmentation trait.

## Materials and methods

### Fly stocks and genetic crosses

Fly stocks were maintained at 25°C on a sugar food medium that was previously described [29]. CRE sequences were obtained from the *D*. *melanogaster* (14021–0231.04), and *D*. *willistoni* (14030–0811.24) species stocks from the San Diego (currently located at Cornell University) Drosophila Stock Center. Genomic DNA for *D*. *simulans*, *D*. *yakuba*, *D*. *biarmipes*, and *D*. *pseudoobscura* were isolated from species stocks obtained from Dr. Sean B. Carroll (Howard Hughes Medical Institute and the University of Maryland).

Tests for effects of *trx* on CRE activities were done using the *yBE0*.*6-EGFP* transgene and the *bab dimorphic element-EGFP* transgene that were separately inserted into the *attP*40 site on chromosome 2 [14,40,41], and *Ddc_MEE1*, t_MSE, and *ebony* activator and male-specific silencer element (*e_AMS*) transgenes that were inserted into the 51D *attP* site on chromosome 2 [12,15,24]. These reporter transgene stocks each possess the *pnr-GAL4* allele on the third chromosome [28] which results in GAL4 expression along the dorsal abdomen midline throughout pupal development in the endogenous pattern of the *pannier* gene [28]. These stocks were crossed to the *UAS-trx* RNAi line (Bloomington Drosophila Stock Center, or BDSC, #31092) that expresses a double stranded RNA upon GAL4 activation which then targets endogenous *trx* mRNAs by the RNAi pathway. As a negative control, the combined *pnr-GAL4* and reporter transgene stocks were separately crossed to the *UAS-mCherry* RNAi line (BDSC #35785). This RNAi line targets the *mCherry* gene that does not exist in the *D*. *melanogaster* genome.

Male flies possessing the *UAS-trx* RNAi transgene were crossed to virgin *pnr-GAL4/TM6B* females (BDSC #58788) to obtain progeny for which Trx expression was knocked down along the dorsal midline of pupa. Similar crosses were performed with the *mCherry* RNAi stock to obtain negative control progeny. These specimens were dissected for use in immunohistochemistry experiments. Trx expression was also studied for the *D*. *melanogaster w1118* (BDSC #3605) and *D*. *willistoni* (14030–0811.24) stocks.

### *cis*-regulatory element prediction

The first SCRMshaw iteration was performed using a set of eight CREs (S1 Document) as the training data. These were the well-characterized dimorphic element and anterior element that together drive a dimorphic pattern of *bric à brac* (*bab*) expression [11], the t_MSE2 and yBE0.6 CREs that activate male-specific expression of *tan* and *yellow* respectively [14], the *ebony* activation element and male repressor element which generate a dimorphic pattern of *ebony* expression [15], and the *Ddc_MEE1* which activates posterior abdomen expression of *Dopa decarboxylase* [12]. This set also included the *yellow-C* intron element that we found to drive reporter gene expression in the posterior abdomen epidermis of *D*. *melanogaster* in a reporter transgene assay. Orthologous and background sequences were selected as previously described [42]. SCRMshaw was run using all three scoring methods (IMM, HexMCD, and PACrc; see [20,21], thitw = 5000, and otherwise default parameters [42]. The top 100 predictions from each scoring method were selected using “Generate_top_N_SCRMhits.pl” and merged to consolidate duplicate and/or overlapping sequences using BEDTools “merge” [43], as described in the SCRMshaw protocol [42].

The second round of SCRMshaw discovery was performed with an expanded training set of 16 sequences, based on the validation results from the first round (S2 Document). The *ebony* male repression element and the *yellow-C* intron element were removed from this set, and replaced by 10 pCREs that were shown to have pupal abdominal epidermis activity in the first iteration of SCRMshaw. SCRMshaw was run with the same parameters as in round 1 except that the “HD” method of windowing the genome and selecting high-scoring predictions was used [19], and only results from the IMM scoring method were included in the final set of predictions (similar results were obtained using standard (non “HD”) SCRMshaw and merging the top 500 hits from all scoring methods).

### Novel EGFP reporter transgenes

EGFP reporter transgenes were used to test whether DNA sequences function as CREs that activate patterns of gene expression. Reporter transgenes were assembled by cloning pCREs or control sequences into the *Asc*I and *Sbf*I restriction enzyme sites for the S3aG vector [23], except for *S2*.*8* that was cloned into the vector’s *Eco*RI and *Sbf*I sites. These cloned DNA sequences reside just 5’ of a minimal *hsp70* promoter and the coding sequence for the EGFP-NLS reporter protein [44]. The resultant *reporter* transgenes analyzed were integrated into the 51D *att*P landing site [24] using ϕC integrase methods (Best Gene Inc.) [45]. The primer pairs used to PCR-amplify *D*. *melanogaster* pCREs are presented in the S7 Table and S8 Table. The primer pairs used to PCR-amplify orthologous CREs to those found in the first intron of *D*. *melanogaster trx* are presented in S9 Table. The primer pairs used to PCR-amplify truncated forms of the *D*. *melanogaster S2*.*20* CRE are presented in S10 Table. The primer pairs used to amplify the randomly-selected control sequences are presented in S11 Table. The orthologous *S2*.*20* sequence of *D*. *willistoni* and the *S2*.*19* sequence of *D*. *yakuba* did not amplify by PCR. Therefore, they were synthesized (GenScript Biotech Corporation) to match the sequence in their genome (S3 Document) [46,47], and then subcloned into the *Asc*I and *Sbf*I sites of S3aG vector. The orthologous sequence to the *trithorax* intron region of *D*. *melanogaster* were obtained as BLAST matches in FlyBase to the *melanogaster* sequence [48]. These sequences were aligned by the MUSCLE program [49]. The aligned sequences are provided in the S4 Document, which includes the annotated positions and sequences for the primers used to amplify the orthologous *S2*.*19* and *S2*.*20* CREs, and the sequences that were synthesized for *D*. *yakuba* and *D*. *willistoni*.

### CRE Removal by CRISPR/Cas9 Gene Editing

Deletion of pCRE sequences were done by GenetiVision Corporation. Acceptable target site sequences for guide RNAs (gRNAs) were identified that flank the *S2*.*19* and *S2*.*20* pCREs (Fig 5A, S3 Table) in the first intron of the *trx* locus. These gRNA sequences were cloned into an expression vector. A donor construct vector containing a *loxP* site-flanked 3xP3-GFP+ selectable marker was prepared for each CRE that has an ~1 kb homology arm to the *trx* intron on each side of the cassette. For each pCRE, a pair of gRNA vectors and the donor vector with the correct homology arms were co-injected into a *w1118* stock that includes a *nos-Cas9* transgene. G0 crosses were set up with *w1118* mates, and F1 progeny were screened for GFP+ flies. GFP+ flies were balanced and genomic DNA PCR and DNA sequencing was done to confirm that donor cassette was inserted in the location of the target pCRE. GFP+ cassettes were removed by crossing confirmed GFP+ lines to mates of a stock containing a transgene on the TM6B balancer chromosome that drives Cre recombinase expression in embryos (BDSC #1501). Resulting GFP-negative flies were crossed to make each CRE deletion allele homozygous and to remove the Cre recombinase transgene. These *trx*^*ΔS2*.*19*^ and *trx*^*ΔS2*.*20*^ stocks were crossed to generate *trans*-heterozygous adults.

### Immunohistochemistry

Dorsal abdomen specimens were dissected from pupae between the P14 and P15(i) developmental stages [12,16,50]. Male and female specimens were combined, and females were distinguished by the removal of their wings. The ensuing steps were done with male and female specimens together in the same tubes or plate in order to expose to identical conditions. Specimens were fixed for 35 min in PBST solution (phosphate buffered saline with 0.3% Triton X-100) with 4% paraformaldehyde (Electron Microscopy Services). After fixation, specimens were washed twice with PBST and then blocked for 1 hour at room temperature in a blocking solution (PBST with 1% Bovine Serum Albumin). Specimens were then incubated overnight at 4°C with a primary antibody in PBST. These were either mouse monoclonal anti-Abd-B (Developmental Studies Hybridoma Bank, 1A2E9) at a dilution of 1:200 of a concentrated stock, rabbit anti-Bab1 primary antibody [11] at a 1:200 dilution, and rabbit anti-Trx [39] at a 1:200 dilution.

After four washes with PBST and then 1 hour in blocking solution, specimens were incubated with either goat anti-mouse Alexa Fluor 647 (Invitrogen, #A21236) secondary antibody, or goat anti-rabbit Alexa Fluor 646 (Invitrogen, #A21244) secondary antibody. These secondary antibodies were used at a 1:500 dilution in PBST, and incubated for 2 hours at room temperature. Following four washes with PBST, the specimens were equilibrated for ten minutes at room temperature in Glycerol Mount:PBST (50% glycerol, 50% PBST) solution. Specimens were then transferred to the glycerol mount (80% glycerol) before being situated between a glass cover slip and slide for imaging with a confocal microscope. Cover slip and slides were separated by one piece of double-sided sticky tape, for which a hole was cut out in the center by a razor blade. Specimens are situated in the hole with their cuticle towards the cover slip side.

### Imaging of *Drosophila* abdomens

The pigmentation patterns on the dorsal abdomens of *Drosophila* fruit flies were imaged using an Olympus SZX16 Zoom Stereoscope running the Olympus cellSens Standard 2.2 software package, and with a mounted DP72 digital camera. All samples were imaged at either 50X or 63X magnification. Prior to imaging, the legs and wings were removed from the adult specimens that were between two to four-days old, and that were mounted on double sided sticky tape that was adhered to a glass slide

Projection images for EGFP-NLS reporter transgene expression were generated with an Olympus Fluoview FV 1000 confocal microscope and software. The regulatory activity of most CREs were documented as EGFP expression patterns at ~80–88 hours after puparium formation (hAPF), a time point during pupal development when most of the training set CREs activate their characterized pattern of reporter expression and that corresponds with the developmental stage when the endogenous genes expressions occur. The exception to this developmental stage were the experiments looking at EGFP reporter expression driven by the *ebony* CREs. For this experiment, specimens were collected as newly eclosed adults and aged for two hours prior to confocal imaging. Robust *ebony* expression has been shown to occur post-eclosion. The standard settings for visualizing EGFP expression patterns were: 30% laser power (488 laser), HV between 650 and 700, offset equal to 1, gain equal to 1, aperture set at 180 microns, Z-series step size of 10 microns, and Kalman line averaging set to 2.

Images were taken of the immunohistochemistry specimens with confocal microscope settings of: 30% laser power (640 laser), HV between 650 and 700, offset equal to 1, gain equal to 1, aperture set at 180 microns, Z-series step size of 2 microns, and Kalman line averaging set to 2.

### Figure development

Sequence visualization for the *trithorax*, *Hr4*, and *sbb* loci of *D*. *melanogaster* (Figs 5 and 9) were made using the GenePalette tool [51]. Sequence conservation of the *trx* intron (S8 Fig) was done using mVISTA tool [52,53] with orthologous *Drosophila* sequences that were obtained by using BLAST at FlyBase [46].

In each figure that compares EGFP expression patterns, a representative image was selected from replicate specimens (n≥3), which were then processed through the same modifications using Photoshop CS3 (Adobe). Confocal projection images and stereoscope images were exported in TIFF, and underwent the same processing steps in Adobe Photoshop CS3. Figures were assembled in Adobe Illustrator CS3.

## Supporting information

S1 Fig Representative EGFP reporter expression driven by each of the 18 tested predicted CREs from the first dorsal pupal abdomen CRE training set. (*EGFP* reporter transgene expressions in the dorsal abdomens of male *D*. *melanogaster* pupa at ~80–88 hours after puparium formation (hAPF) or the P13-P14(i) developmental stage. Expressions in the female abdomens were similar and therefore not reported here. pCREs that drove no noteworthy expression in the dorsal abdominal epidermis have their names in red font color.
(TIF)

S2 Fig Closeup of EGFP reporter expression by three pCREs. (*EGFP* reporter transgene expressions in the dorsal abdomens of male *D*. *melanogaster* pupa at ~80–88 hours after puparium formation (hAPF) or the P13-P14(i) developmental stage. Red dashed boxes indicate regions that are shown at higher magnification. *S1*.*14* and *S1*.*15* pCREs drive conspicuous expression in bristle associated cells, whereas the *S1*.*16* pCRE drove reporter expression in bristle associated cells and more broadly throughout the epidermis. Expressions in the female abdomens were similar and therefore not reported here. pCREs that drove no noteworthy expression in the dorsal abdominal epidermis have their names in red font color.
(TIF)

S3 Fig 14 randomly-selected non-coding sequences fail to drive noteworthy pupal abdominal epidermis reporter expression. (A-P) EGFP expression in male pupae at ~80–88 hAPF developmental (Stages P13 to P14-15i) and with the generic genotype *UAS-GFP*.*nls* (BDSC #4775)/+; *Non-coding sequence-GAL4* (BDSC ID# listed on figure panel)/+. Transgene information provided in S3 Table. (O) Negative control where the *GAL4* gene lacks an associated non-coding DNA sequence—genotype *UAS-GFP*.*nls* (BDSC #4775)/+; *GAL4* (BDSC ID#68384)/+. (P) Positive control showing EGFP expression in the A5 and A6 segments of a female pupa of genotype *UAS-GFP*.*nls* (BDSC #4775)/+; *DRE D*. *fuyami-GAL4/+*. All GAL4 transgenes are located in the *att*P2 site.
(TIF)

S4 Fig Representative EGFP reporter expression driven by each of the 22 tested predicted CREs from the second dorsal pupal abdomen CRE training set. (*EGFP* reporter transgene expressions in the dorsal abdomens of male *D*. *melanogaster* pupa at ~80–88 hours after puparium formation (hAPF) or the P13-P14(i) developmental stage. Expressions in the female abdomens were similar and therefore not reported here. pCREs that drove no noteworthy expression in the dorsal abdominal epidermis have their names in red font color.
(TIF)

S5 Fig Replicate *trithorax* CRE deletion specimens demonstrate consistency in the tergite pigmentation aberrations. Adult abdomen tergite pigmentation phenotypes for five independent male and female specimens with the same *trx* locus genotypes. Similar pigmentation phenotypes are observed for individuals of the same sex and genotype.
(TIF)

S6 Fig Replicate specimens support the conclusion that reduced Trithorax expression has little to no effect on pupal-stage expression of Abd-B in the abdomen epidermis. (A-A”“) Specimens for which RNAi targeted the negative control *mCherry* gene in the dorsal abdomen midline region. (B-B”“) Specimens for which RNAi targeted the *trithorax* gene in the dorsal abdomen midline region. (A) While *mCherry* RNAi had no effect on adult male tergite pigmentation, (B) a noticeable loss of pigmentation occurred following RNAi for *trx*. Neither (A’-A”“) *mCherry* RNAi nor (B’-B”“) *trx* RNAi had a noticeable effect on the A5 and A6 segment expression of Abd-B. Besides the expected Abd-B nuclear expression, these specimens show some non-specific expression in muscle tissue. The images in A and B are those used in Fig 6.
(TIF)

S7 Fig Replicate specimens support the conclusion that reduced Trithorax expression has little to no effect on pupal-stage expression of Bab1 in the abdomen epidermis. (A-A” and C-C”) Male and (B-B” and D-D”) female specimens. (A-A” and B-B”) Specimens for which RNAi targeted the negative control *mCherry* gene in the dorsal abdomen midline region. (C-C” and D-D”) Specimens for which RNAi targeted the *trx* gene in the dorsal abdomen midline region. Neither *mCherry* RNAi nor *trx* RNAi altered the dimorphic (little to no expression in males, and robust epidermal expression in females) pattern of Bab1 expression in the pupal abdominal epidermis tissue. Red and yellow dashed boxes respectively surround a representative region of the male and female midline region where RNAi occurred by *pnr-GAL4* expression.
(TIF)

S8 Fig Midline reduction in *trx* expression by RNAi had little to no effect on the regulatory activity of the *bab* dimorphic element. EGFP reporter transgene expression driven by the *bab* gene locus dimorphic element (DRE) CRE in genetic backgrounds where RNAi was performed in the midline region for the negative control *mCherry* gene and for *trithorax*. EGFP expression was recorded at the P13-14(i) stage. Neither RNAi for the negative control *mCherry* or *trx* caused a noticeable change in reporter expression in males or females.
(TIF)

S9 Fig Midline reduction in *trx* expression by RNAi had little to no effect on the regulatory activity of the *Ddc_MEE1* CRE. EGFP reporter transgene expression driven by the *Ddc_MEE1* CRE in genetic backgrounds where RNAi was performed in the midline region for the negative control *mCherry* gene and for *trithorax*. EGFP expression was recorded at the P13-14(i) stage. Neither RNAi for the negative control *mCherry* or *trx* caused a noticeable change in reporter expression in males or females.
(TIF)

S10 Fig Midline reduction in *trx* expression by RNAi had little to no effect on the regulatory activity of the *yellow* gene’s yBE0.6 CRE. EGFP reporter transgene expression driven by the *yBE0*.*6* CRE in genetic backgrounds where RNAi was performed in the midline region for the negative control *mCherry* gene and for *trithorax*. EGFP expression was recorded at the P13-14(i) stage. Neither RNAi for the negative control *mCherry* or *trx* caused a noticeable change in reporter expression in males or females.
(TIF)

S11 Fig Midline reduction in *trx* expression by RNAi results in a repeatable reduction of t_MSE CRE activity in the male A5 segment. EGFP reporter transgene expression driven by CREs in genetic backgrounds where RNAi was performed in the midline region for the negative control *mCherry* gene and for *trithorax*. In all specimens, EGFP expression was driven by the *t_MSE* CRE and imaged at the P14-15(i) stage. Red arrowheads indicate segments where the genetic background alterations resulted in a reduced EGFP reporter expression.
(TIF)

S12 Fig Midline reduction in *trx* expression by RNAi did not result in a noteworthy change in t_MSE CRE activity in the female abdomen. EGFP reporter transgene expression driven by CREs in genetic backgrounds where RNAi was performed in the midline region for the (top row) negative control *mCherry* gene and (bottom row) for *trithorax*. In all specimens, EGFP expression was driven by the *t_MSE* CRE and imaged within the P14-15(i) stage. Age matters for the onset and strength of t_MSE activity; younger specimens express EGFP at a low to modest level in the A6 epidermis, whereas slightly more advanced specimens show more robust EGFP expression in the A6 segment. Though we only analyzed a small number of specimens, the results lacked a compelling difference in the midline level of EGFP expression between control and *trx* RNAi specimens.
(TIF)

S13 Fig Midline reduction in *trx* expression by RNAi results in a repeatable gain of *e_AMS* CRE activity in the male A5 and A6 segments. EGFP reporter transgene expression driven by CREs in genetic backgrounds where RNAi was performed in the midline region for the negative control *mCherry* gene and for *trithorax*. In all specimens, EGFP expression was driven by the *e_AMS* CREs and imaged at 2 hours after eclosion. Yellow arrowheads indicate segments where the genetic background alterations resulted in conspicuous ectopic EGFP reporter expression.
(TIF)

S14 Fig Midline reduction in *trx* expression by RNAi results in a repeatable gain of *e_AMS* CRE activity in the female A5 and A6 segments. EGFP reporter transgene expression driven by CREs in genetic backgrounds where RNAi was performed in the midline region for the negative control *mCherry* gene and for *trithorax*. In all specimens, EGFP expression was driven by the *e_AMS* CREs and imaged at 2 hours after eclosion. Yellow arrowheads indicate the A5 and A6 segments where the genetic background alterations resulted in conspicuous ectopic EGFP reporter expression.
(TIF)

S15 Fig Sequence conservation of *trx* CREs among *Sophophora* subgenus fruit fly species, and the distribution of functional sequences in the *S2*.*20* CRE as revealed by reporter transgene assays with truncated versions of *S2*.*20*. The *trx* first intron sequences were aligned with mVISTA to visualize conservation among species with ancestral or derived tergite pigmentation phenotypes. The node marked 1 indicates the last common ancestor (LCA) between *D*. *willistoni* and the other species, an ancestor suspected to have monomorphic non-melanic tergite pigmentation. The node marked 2 represents the LCA of the *obscura* species group for which a melanic monomorphic tergite pigmentation is the norm. The node marked 3 indicates the LCA of species that descend from an ancestor with the derived dimorphic tergite pigmentation. (B) The *S2*.*20* sequence of *D*. *melanogaster* was truncated into 8 smaller fragments that were tested for enhancer activity on the *EGFP* reporter transgene in *D*. *melanogaster* pupae. (C-K) EGFP expression patterns in the abdomens of male pupae at ~80–88 hours after puparium formation (hAPF) or the P13-P14(i) developmental stage. Though expression is shown for males, expression in females was not noticeably different.
(TIF)

S16 Fig Pupal abdominal epidermis expression of Trithorax is conserved between *D*. *melanogaster* and *D*. *willistoni*. Trx protein expression in the abdominal epidermis of (A) male and (B) female *D*. *melanogaster* and (C) male and (D) female *D*. *willistoni* at the P14-15(i) developmental stage. Dashed red boxes in A-D indicate the zoomed in regions shown in A’-D’.
(TIF)

S1 Document The DNA sequences for the eight known CREs that were used as “training set” input to search the *D*. *melanogaster* genome using the SCRMshaw CRE discovery algorithm. (DOCX)

S2 Document Updated training set with16 sequences used to again screen the *D*. *melanogaster* genome by SCRMshaw for predicted CREs. (DOCX)

S3 Document Synthesized and subcloned sequences orthologous to either the *D*. *melanogaster S2*.*19* or *S2*.*20 trithorax* CRE. (DOCX)

S4 Document Alignment of orthologous DNA sequences to the *D*. *melanogaster trithorax* intron region in which resides the *S2*.*19* and S2.20 CREs. (DOCX)

S1 Table 232 predicted abdominal pigmentation CREs in the *D*. *melanogaster* genome from the SCRMshaw analysis with the first training set of pigmentation GRN CREs. (DOCX)

S2 Table Non-coding sequence controls with similar nucleotide contents to a *D*. *melanogaster* pigmentation GRN CRE. (DOCX)

S3 Table Arbitrarily-selected transgenic lines that each possess a non-coding sequence-GAL4 transgene in the *att*P2 site. (DOCX)

S4 Table >700 predicted abdominal pigmentation CREs in the *D*. *melanogaster* genome from the SCRMshaw analysis with the second training set of CREs. (DOCX)

S5 Table Iterative SCRMshaw improvement. (DOCX)

S6 Table Sequences targeted by gRNAs for CRISPR/Cas9 deletion of *trithorax* intron 1 CREs. (DOCX)

S7 Table Primer pairs used to create CRE reporter transgenes to test 18 predicted CREs from the first dorsal pupal abdomen CRE training set. (DOCX)

S8 Table Primer pairs used to create CRE reporter transgenes to test 22 predicted CREs from the second dorsal pupal abdomen CRE training set. (DOCX)

S9 Table Primer pairs used to create CRE reporter transgenes with sequences orthologous to the *D*. *melanogaster S2*.*19* and *S2*.*20 trithorax* intron CREs. (DOCX)

S10 Table Primer pairs used to create CRE reporter transgenes with truncated versions of the *D*. *melanogaster S2*.*20 trithorax* CRE. (DOCX)

S11 Table Primer pairs used to create CRE reporter transgenes to test non-coding sequences selected solely for the possession of similar nucleotide compositions to a specific training set CRE. (DOCX)
