# Supplementary figures and images for: A novel role for trithorax in the gene regulatory network for a rapidly evolving fruit fly pigmentation trait
Source: PLoS Genet. 2023 Feb 16;19(2):e1010653. doi: 10.1371/journal.pgen.1010653 (PMC9977049; doi:10.1371/journal.pgen.1010653)

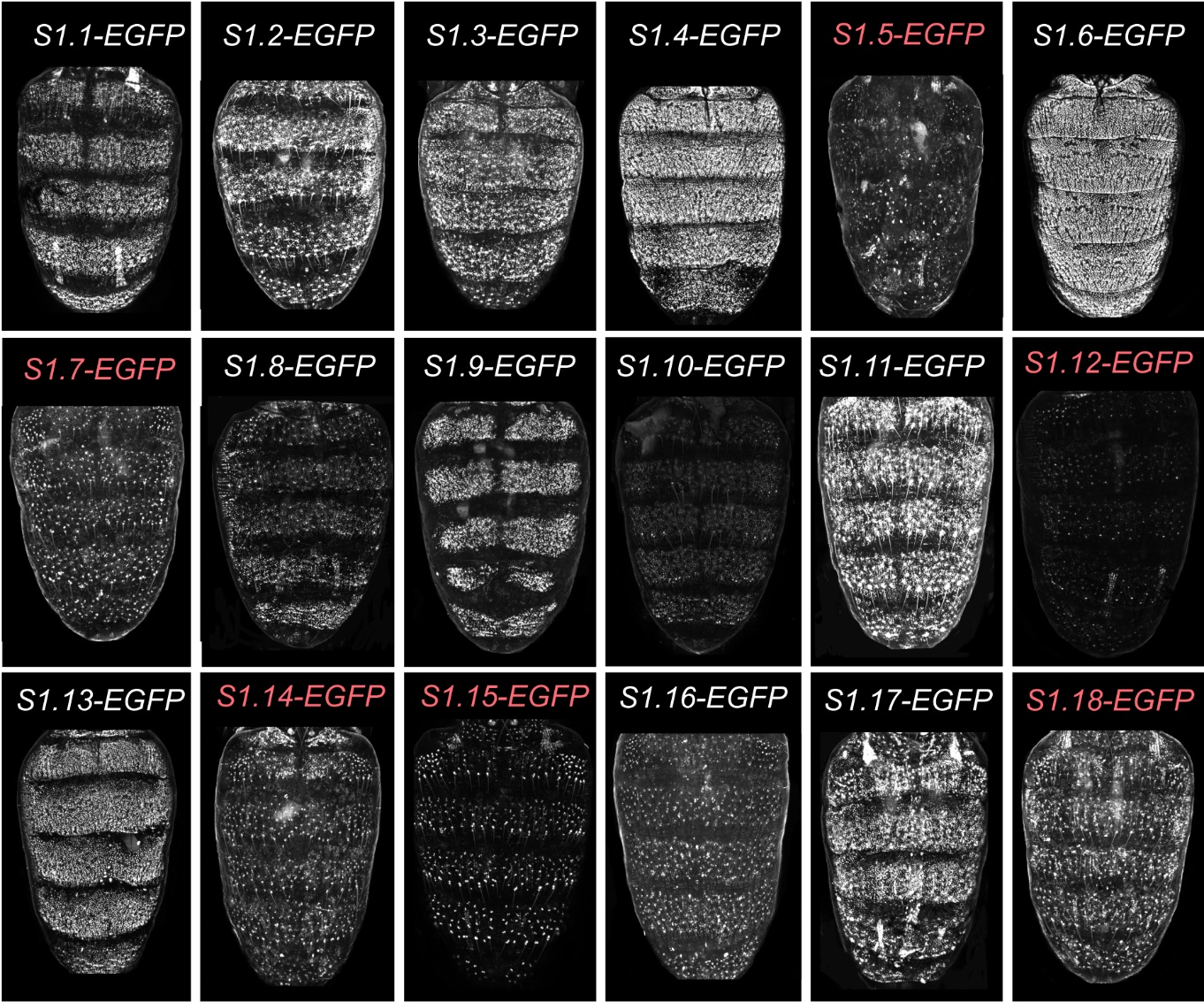

Supplement: S1 Fig — (EGFP reporter transgene expressions in the dorsal abdomens of male D. melanogaster pupa at ~80–88 hours after puparium formation (hAPF) or the P13-P14(i) developmental stage. Expressions in the female abdomens were similar and therefore not reported here. pCREs that drove no noteworthy expression in the dorsal abdominal epidermis have their names in red font color. (TIF) [file pgen.1010653.s001.tif]

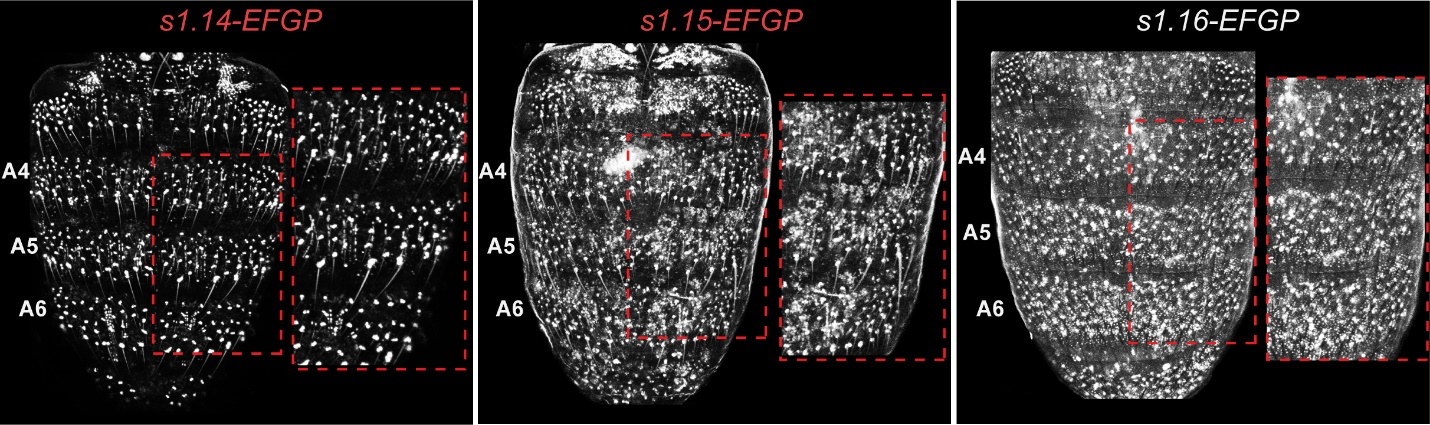

Supplement: S2 Fig — (EGFP reporter transgene expressions in the dorsal abdomens of male D. melanogaster pupa at ~80–88 hours after puparium formation (hAPF) or the P13-P14(i) developmental stage. Red dashed boxes indicate regions that are shown at higher magnification. S1.14 and S1.15 pCREs drive conspicuous expression in bristle associated cells, whereas the S1.16 pCRE drove reporter expression in bristle associated cells and more broadly throughout the epidermis. Expressions in the female abdomens were similar and therefore not reported here. pCREs that drove no noteworthy expression in the dorsal abdominal epidermis have their names in red font color. (TIF) [file pgen.1010653.s002.tif]

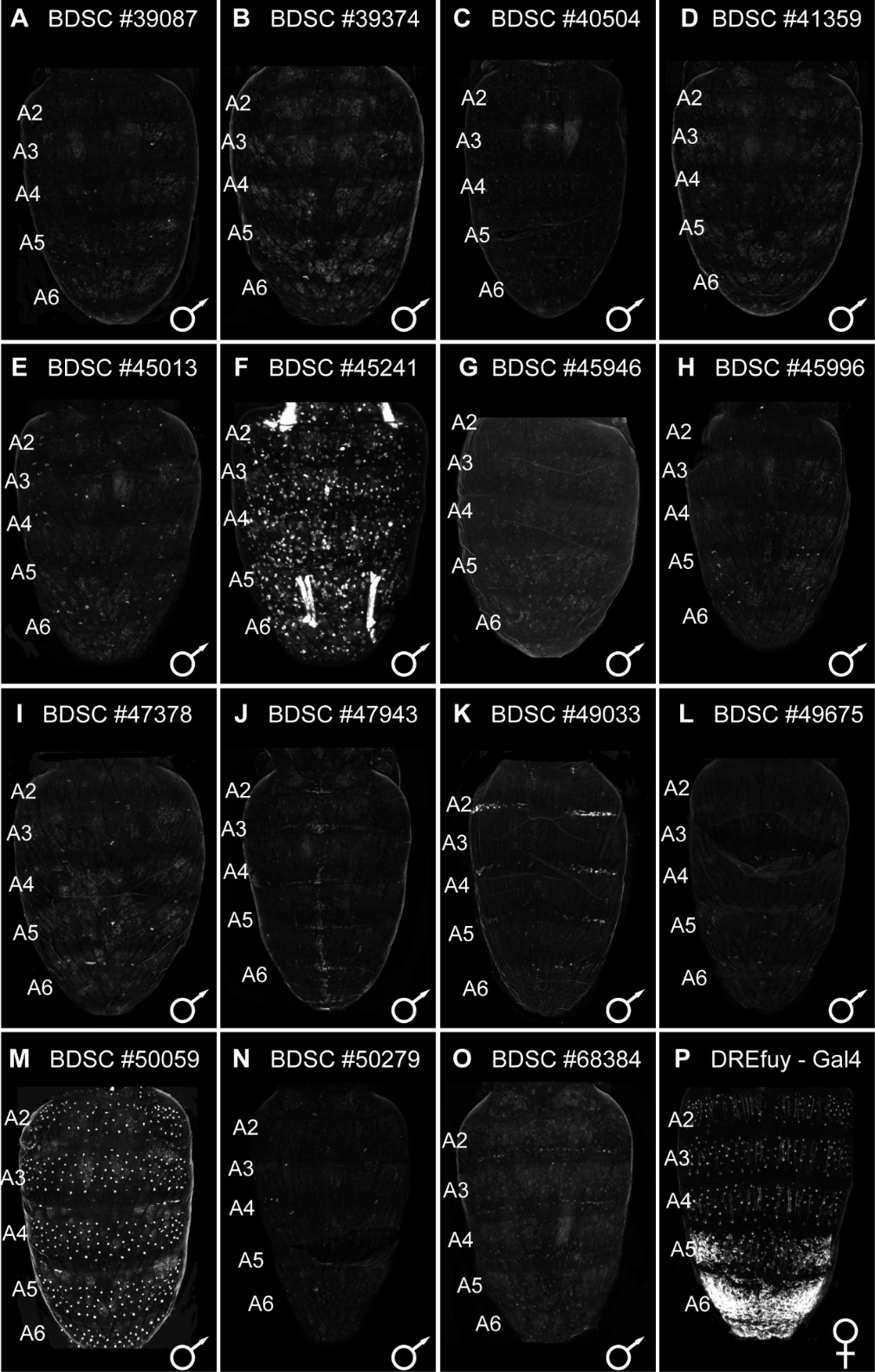

Supplement: S3 Fig — (A-P) EGFP expression in male pupae at ~80–88 hAPF developmental (Stages P13 to P14-15i) and with the generic genotype UAS-GFP.nls (BDSC #4775)/+; Non-coding sequence-GAL4 (BDSC ID# listed on figure panel)/+. Transgene information provided in S3 Table. (O) Negative control where the GAL4 gene lacks an associated non-coding DNA sequence—genotype UAS-GFP.nls (BDSC #4775)/+; GAL4 (BDSC ID#68384)/+. (P) Positive control showing EGFP expression in the A5 and A6 segments of a female pupa of genotype UAS-GFP.nls (BDSC #4775)/+; DRE D. fuyami-GAL4/+. All GAL4 transgenes are located in the attP2 site. (TIF) [file pgen.1010653.s003.tif]

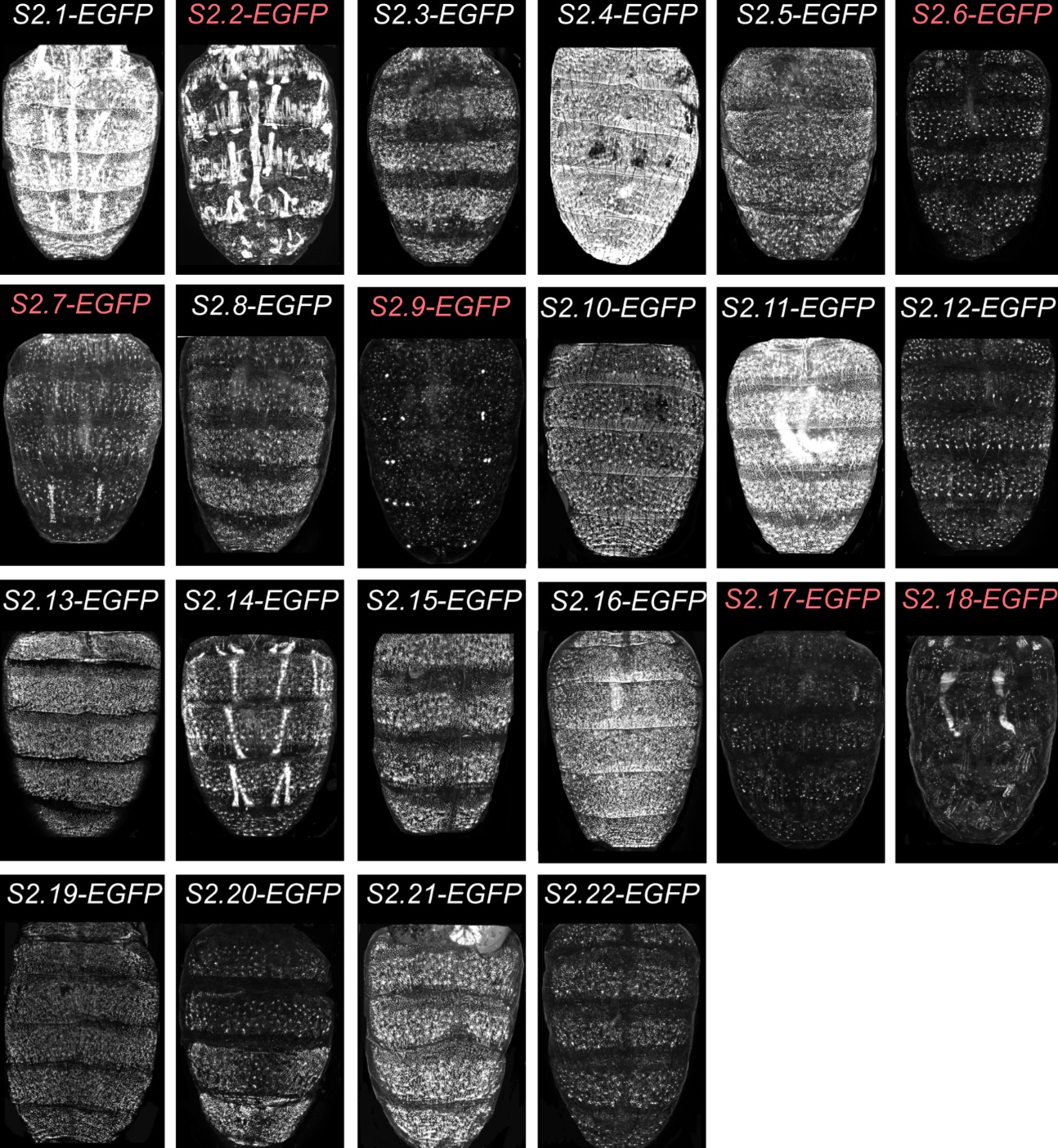

Supplement: S4 Fig — (EGFP reporter transgene expressions in the dorsal abdomens of male D. melanogaster pupa at ~80–88 hours after puparium formation (hAPF) or the P13-P14(i) developmental stage. Expressions in the female abdomens were similar and therefore not reported here. pCREs that drove no noteworthy expression in the dorsal abdominal epidermis have their names in red font color. (TIF) [file pgen.1010653.s004.tif]

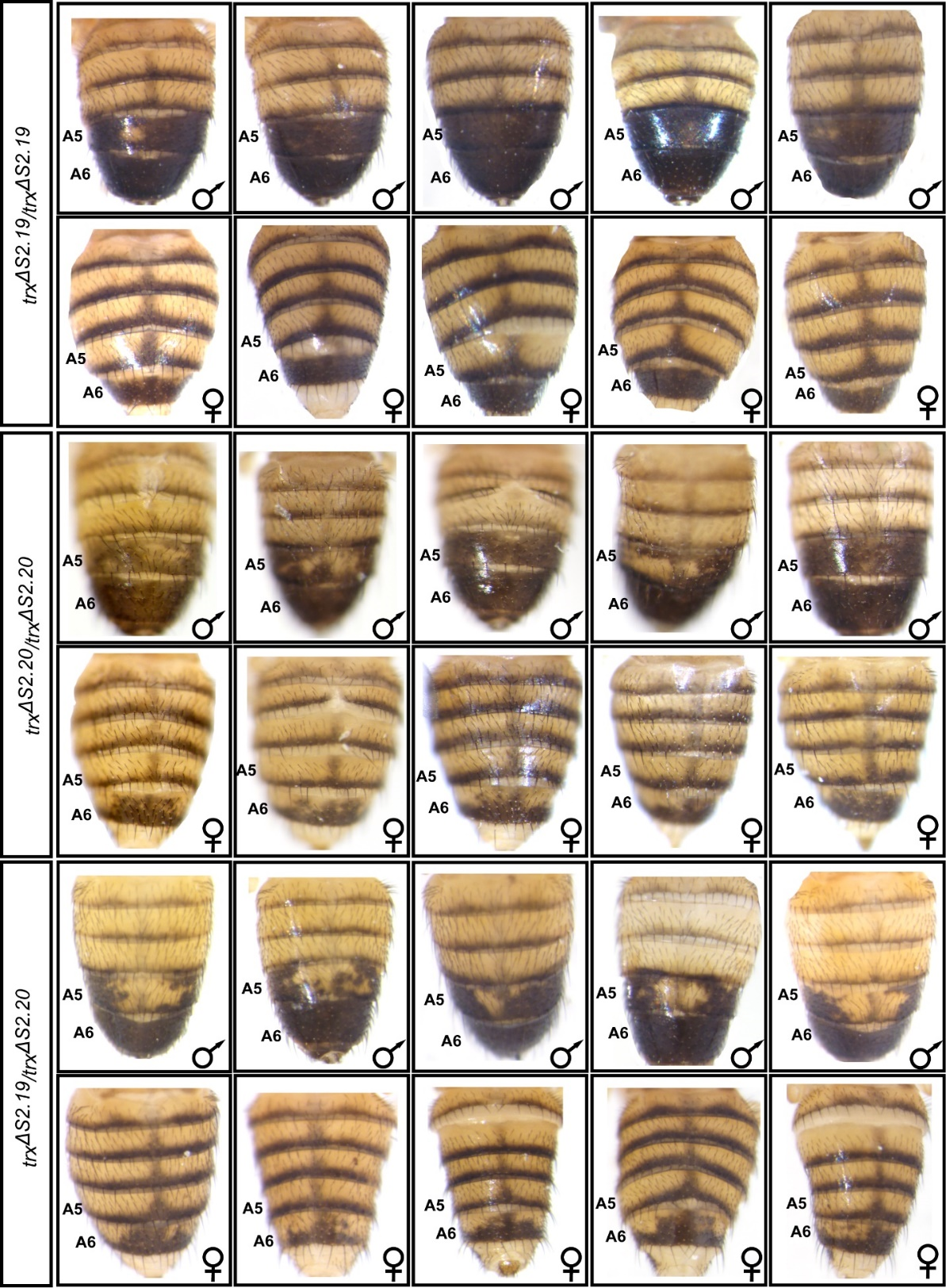

Supplement: S5 Fig — Adult abdomen tergite pigmentation phenotypes for five independent male and female specimens with the same trx locus genotypes. Similar pigmentation phenotypes are observed for individuals of the same sex and genotype. (TIF) [file pgen.1010653.s005.tif]

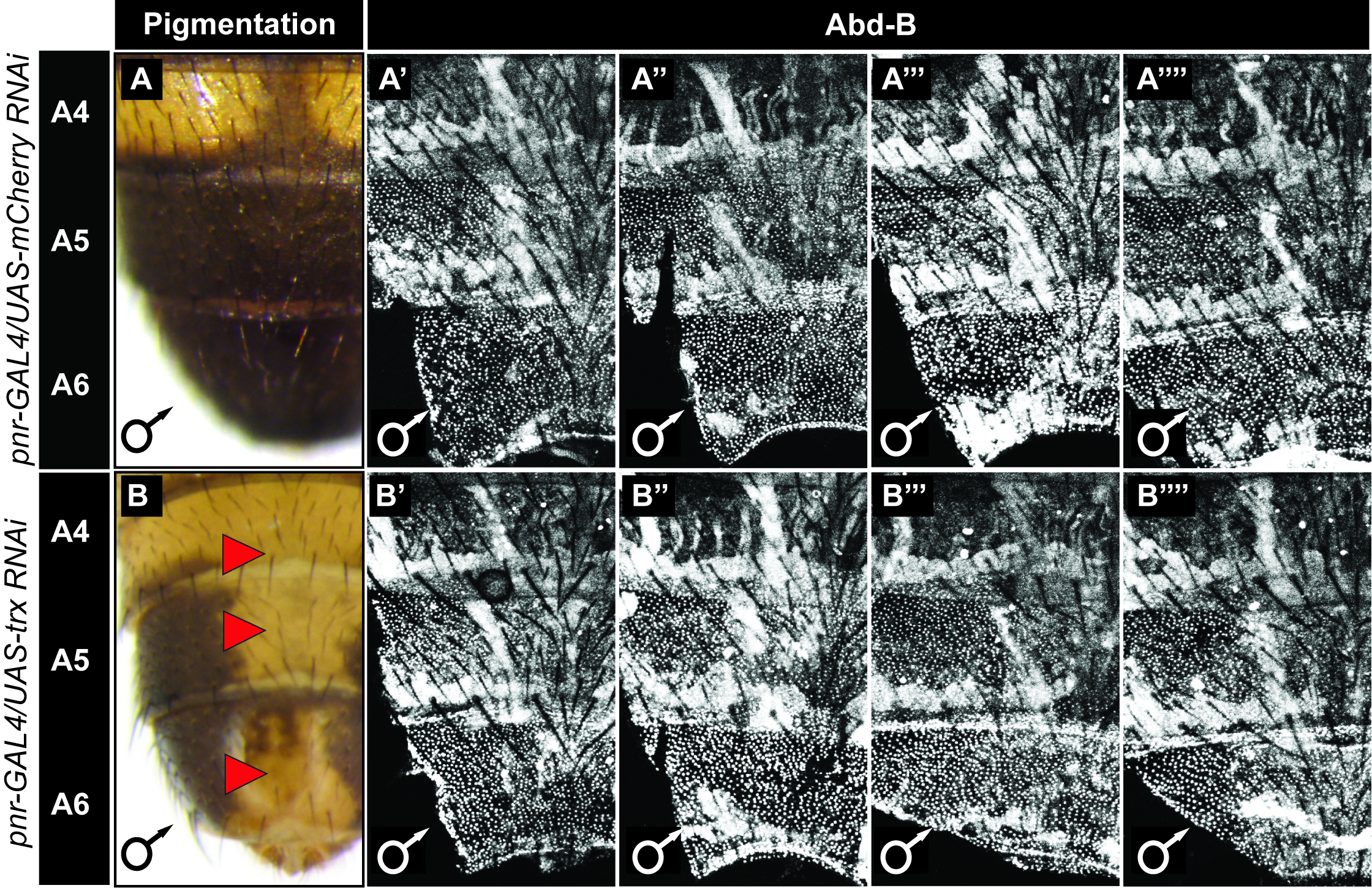

Supplement: S6 Fig — (A-A”“) Specimens for which RNAi targeted the negative control mCherry gene in the dorsal abdomen midline region. (B-B”“) Specimens for which RNAi targeted the trithorax gene in the dorsal abdomen midline region. (A) While mCherry RNAi had no effect on adult male tergite pigmentation, (B) a noticeable loss of pigmentation occurred following RNAi for trx. Neither (A’-A”“) mCherry RNAi nor (B’-B”“) trx RNAi had a noticeable effect on the A5 and A6 segment expression of Abd-B. Besides the expected Abd-B nuclear expression, these specimens show some non-specific expression in muscle tissue. The images in A and B are those used in Fig 6. (TIF) [file pgen.1010653.s006.tif]

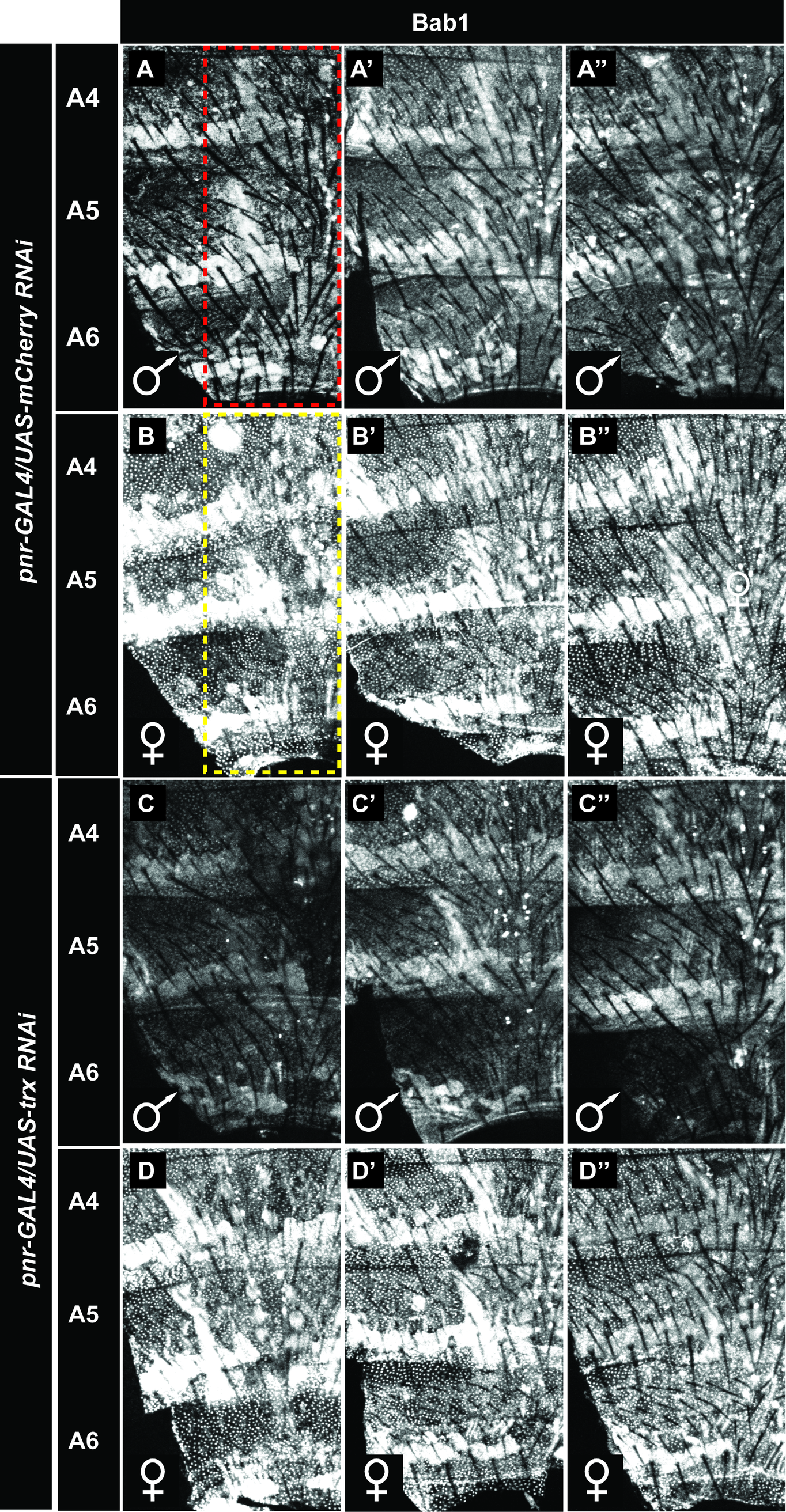

Supplement: S7 Fig — (A-A” and C-C”) Male and (B-B” and D-D”) female specimens. (A-A” and B-B”) Specimens for which RNAi targeted the negative control mCherry gene in the dorsal abdomen midline region. (C-C” and D-D”) Specimens for which RNAi targeted the trx gene in the dorsal abdomen midline region. Neither mCherry RNAi nor trx RNAi altered the dimorphic (little to no expression in males, and robust epidermal expression in females) pattern of Bab1 expression in the pupal abdominal epidermis tissue. Red and yellow dashed boxes respectively surround a representative region of the male and female midline region where RNAi occurred by pnr-GAL4 expression. (TIF) [file pgen.1010653.s007.tif]

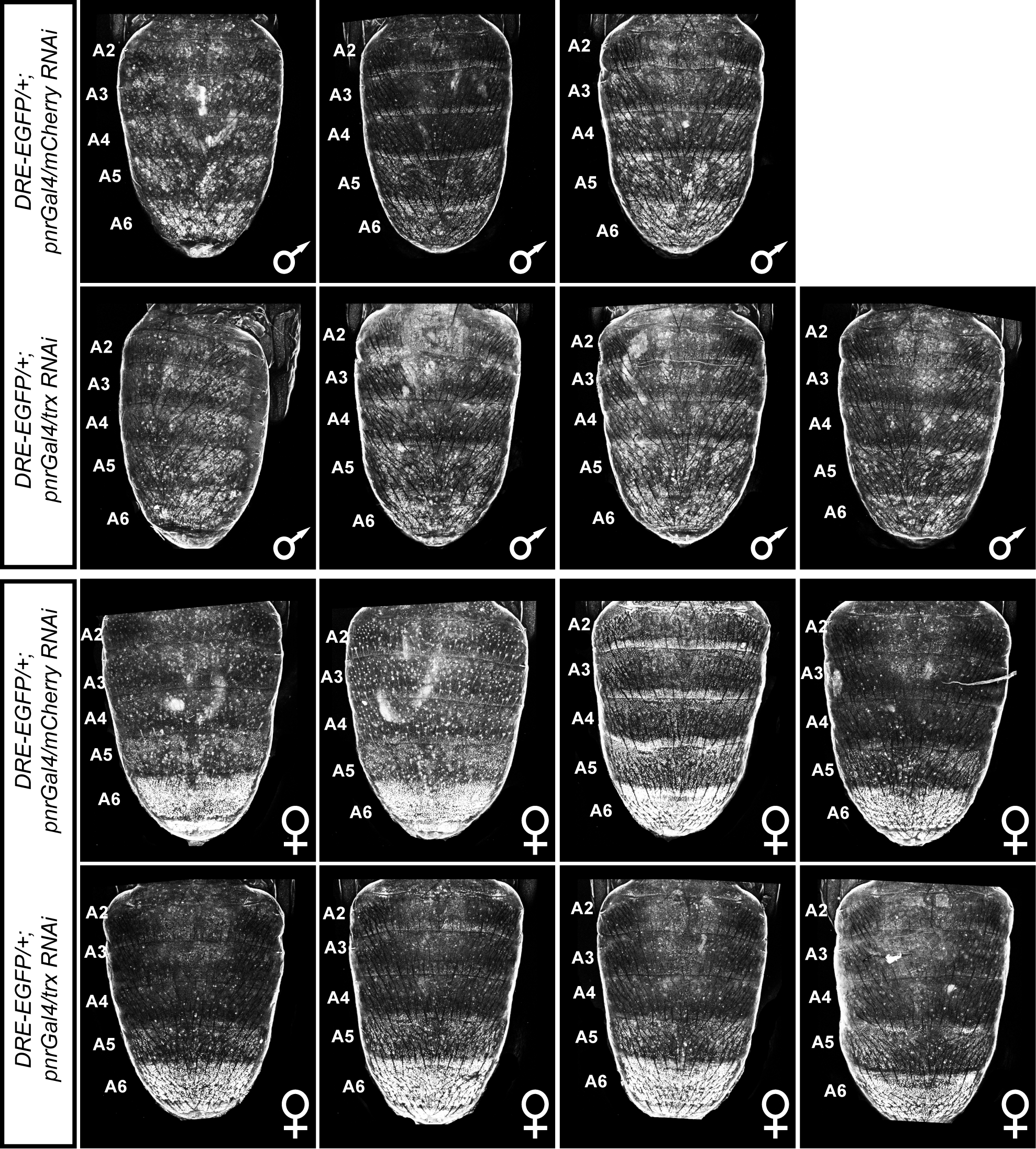

Supplement: S8 Fig — EGFP reporter transgene expression driven by the bab gene locus dimorphic element (DRE) CRE in genetic backgrounds where RNAi was performed in the midline region for the negative control mCherry gene and for trithorax. EGFP expression was recorded at the P13-14(i) stage. Neither RNAi for the negative control mCherry or trx caused a noticeable change in reporter expression in males or females. (TIF) [file pgen.1010653.s008.tif]

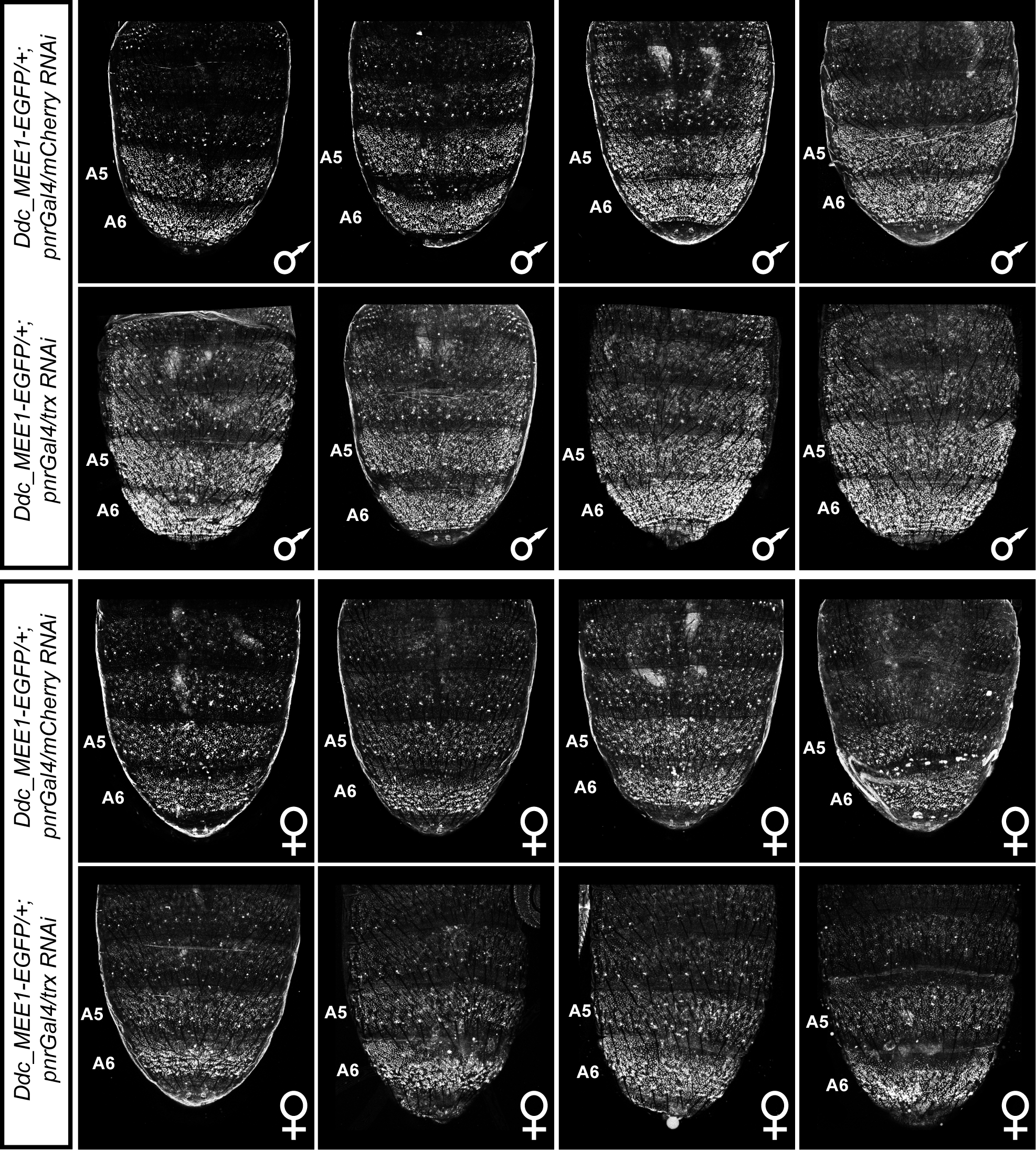

Supplement: S9 Fig — EGFP reporter transgene expression driven by the Ddc_MEE1 CRE in genetic backgrounds where RNAi was performed in the midline region for the negative control mCherry gene and for trithorax. EGFP expression was recorded at the P13-14(i) stage. Neither RNAi for the negative control mCherry or trx caused a noticeable change in reporter expression in males or females. (TIF) [file pgen.1010653.s009.tif]

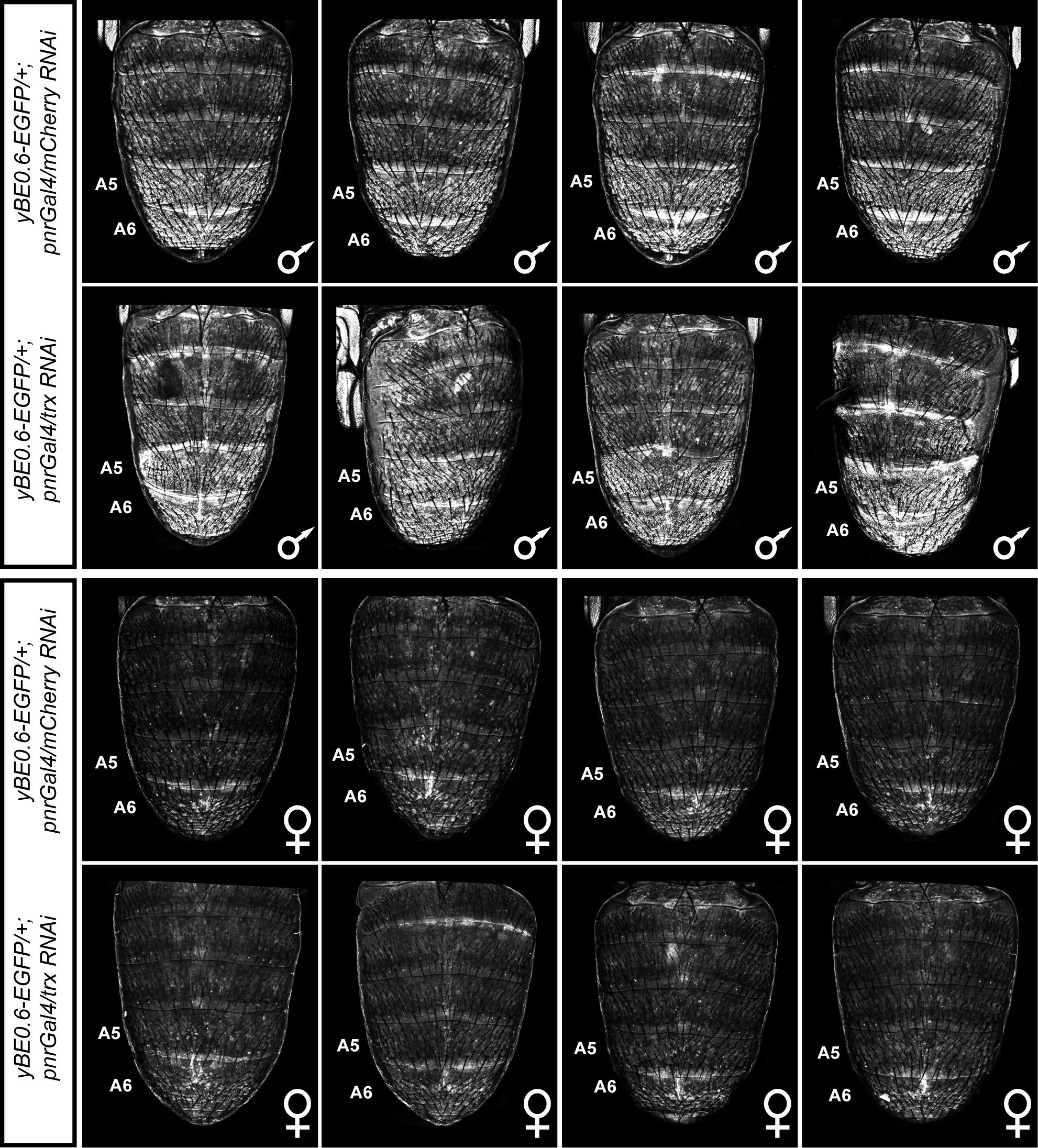

Supplement: S10 Fig — EGFP reporter transgene expression driven by the yBE0.6 CRE in genetic backgrounds where RNAi was performed in the midline region for the negative control mCherry gene and for trithorax. EGFP expression was recorded at the P13-14(i) stage. Neither RNAi for the negative control mCherry or trx caused a noticeable change in reporter expression in males or females. (TIF) [file pgen.1010653.s010.tif]

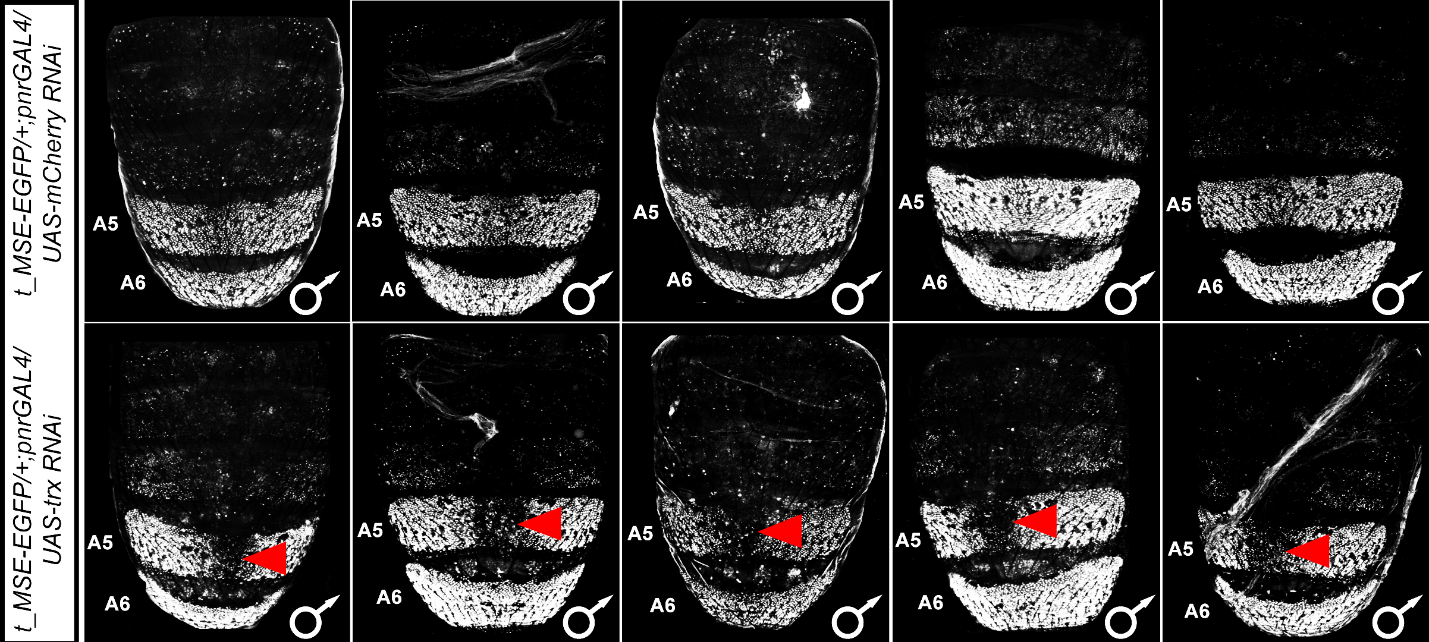

Supplement: S11 Fig — EGFP reporter transgene expression driven by CREs in genetic backgrounds where RNAi was performed in the midline region for the negative control mCherry gene and for trithorax. In all specimens, EGFP expression was driven by the t_MSE CRE and imaged at the P14-15(i) stage. Red arrowheads indicate segments where the genetic background alterations resulted in a reduced EGFP reporter expression. (TIF) [file pgen.1010653.s011.tif]

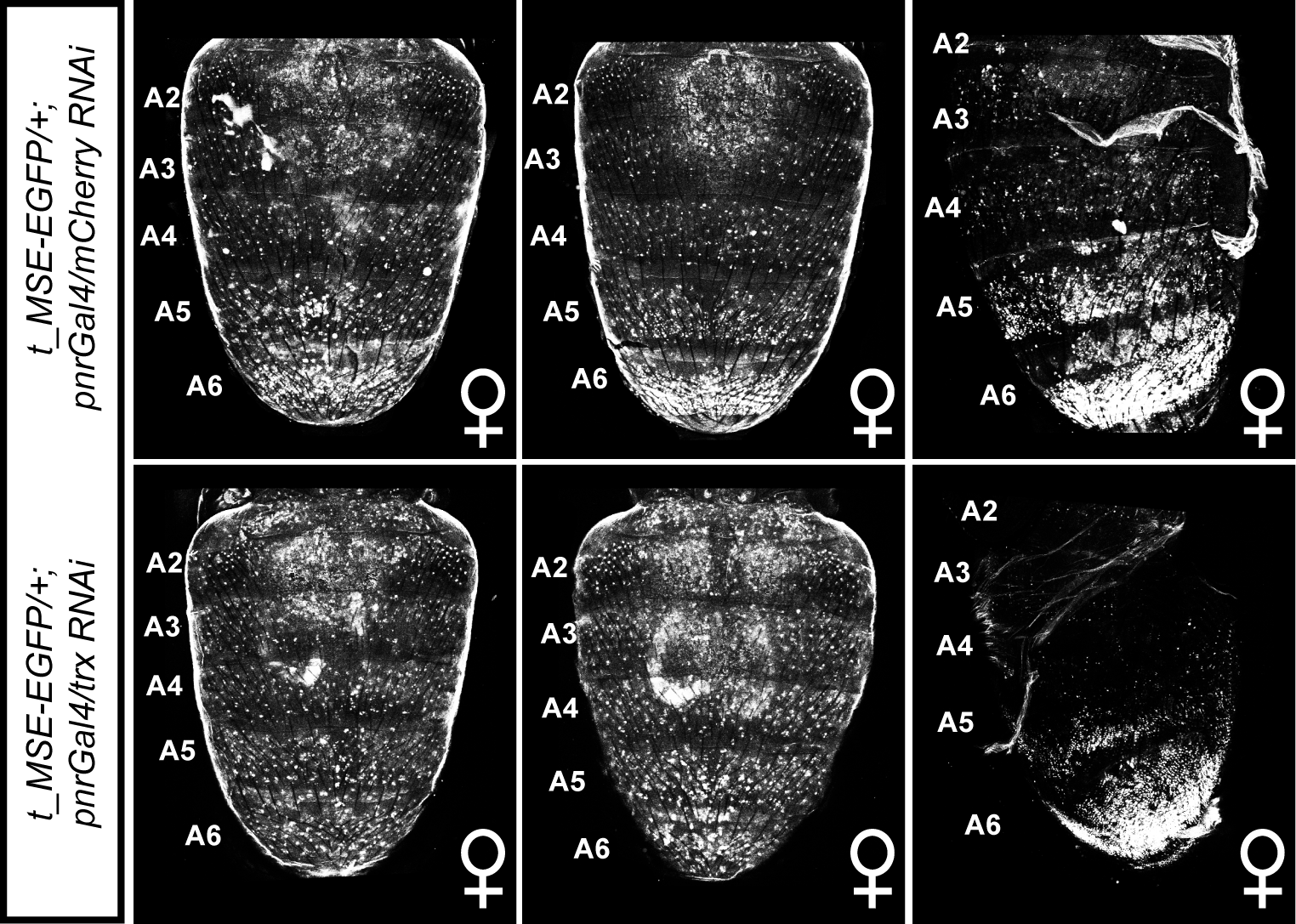

Supplement: S12 Fig — EGFP reporter transgene expression driven by CREs in genetic backgrounds where RNAi was performed in the midline region for the (top row) negative control mCherry gene and (bottom row) for trithorax. In all specimens, EGFP expression was driven by the t_MSE CRE and imaged within the P14-15(i) stage. Age matters for the onset and strength of t_MSE activity; younger specimens express EGFP at a low to modest level in the A6 epidermis, whereas slightly more advanced specimens show more robust EGFP expression in the A6 segment. Though we only analyzed a small number of specimens, the results lacked a compelling difference in the midline level of EGFP expression between control and trx RNAi specimens. (TIF) [file pgen.1010653.s012.tif]

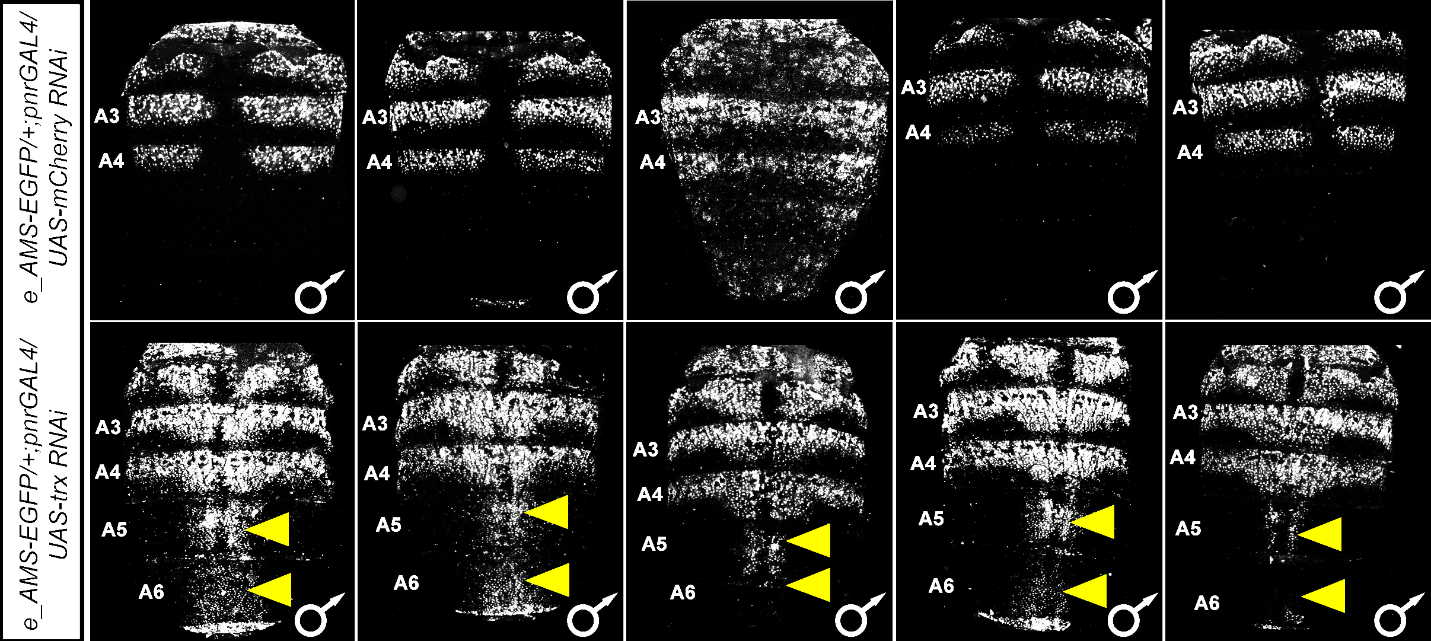

Supplement: S13 Fig — EGFP reporter transgene expression driven by CREs in genetic backgrounds where RNAi was performed in the midline region for the negative control mCherry gene and for trithorax. In all specimens, EGFP expression was driven by the e_AMS CREs and imaged at 2 hours after eclosion. Yellow arrowheads indicate segments where the genetic background alterations resulted in conspicuous ectopic EGFP reporter expression. (TIF) [file pgen.1010653.s013.tif]

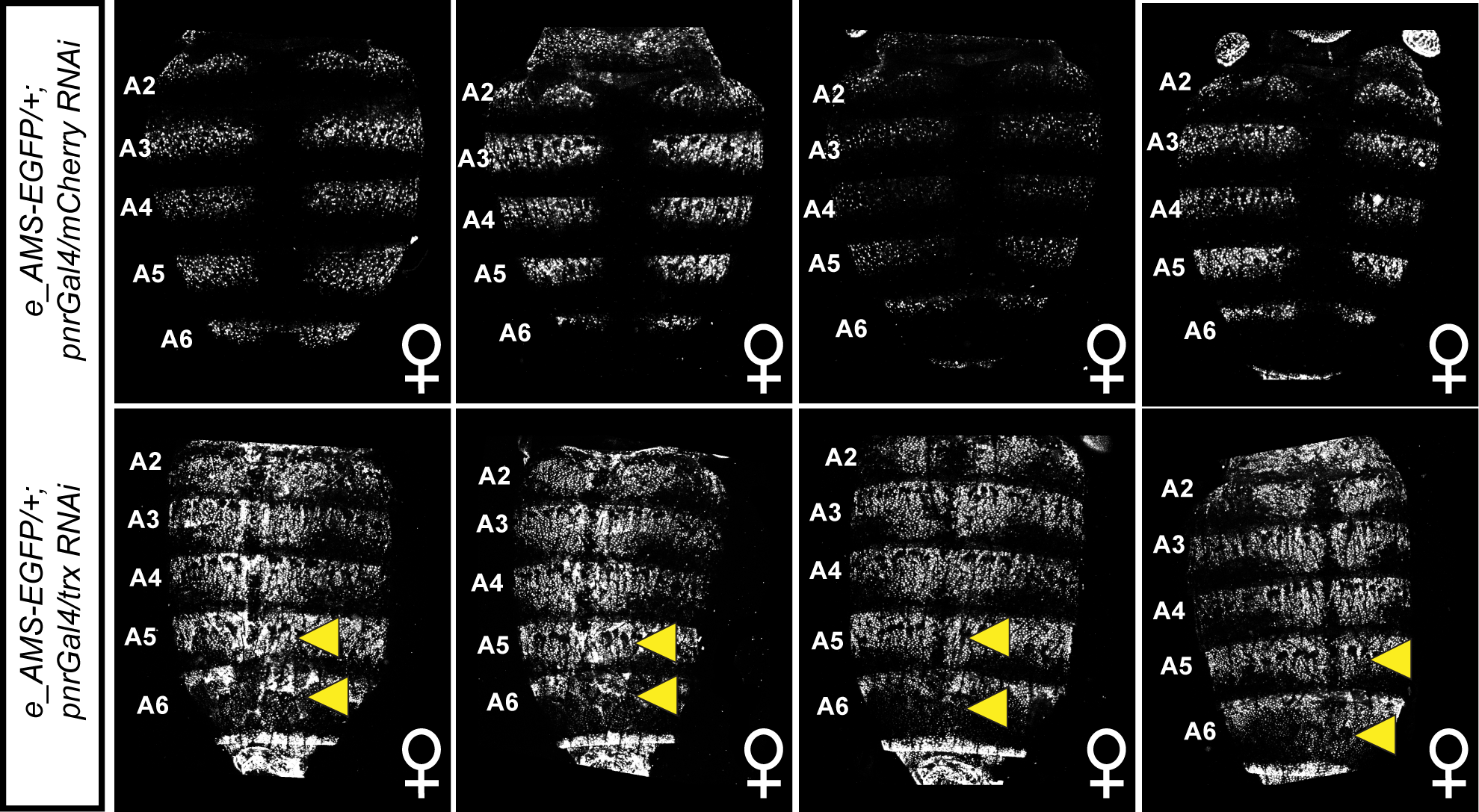

Supplement: S14 Fig — EGFP reporter transgene expression driven by CREs in genetic backgrounds where RNAi was performed in the midline region for the negative control mCherry gene and for trithorax. In all specimens, EGFP expression was driven by the e_AMS CREs and imaged at 2 hours after eclosion. Yellow arrowheads indicate the A5 and A6 segments where the genetic background alterations resulted in conspicuous ectopic EGFP reporter expression. (TIF) [file pgen.1010653.s014.tif]

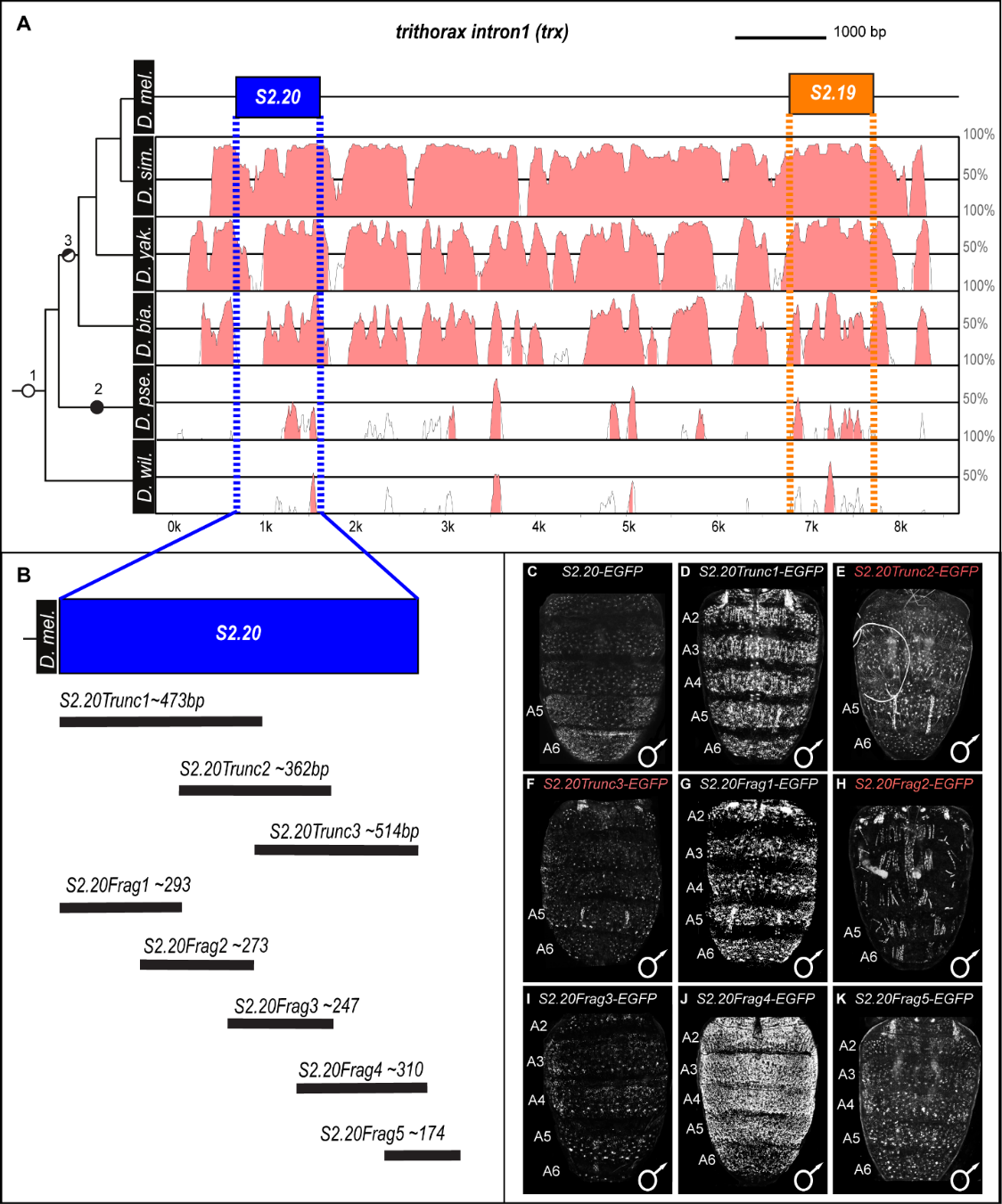

Supplement: S15 Fig — The trx first intron sequences were aligned with mVISTA to visualize conservation among species with ancestral or derived tergite pigmentation phenotypes. The node marked 1 indicates the last common ancestor (LCA) between D. willistoni and the other species, an ancestor suspected to have monomorphic non-melanic tergite pigmentation. The node marked 2 represents the LCA of the obscura species group for which a melanic monomorphic tergite pigmentation is the norm. The node marked 3 indicates the LCA of species that descend from an ancestor with the derived dimorphic tergite pigmentation. (B) The S2.20 sequence of D. melanogaster was truncated into 8 smaller fragments that were tested for enhancer activity on the EGFP reporter transgene in D. melanogaster pupae. (C-K) EGFP expression patterns in the abdomens of male pupae at ~80–88 hours after puparium formation (hAPF) or the P13-P14(i) developmental stage. Though expression is shown for males, expression in females was not noticeably different. (TIF) [file pgen.1010653.s015.tif]

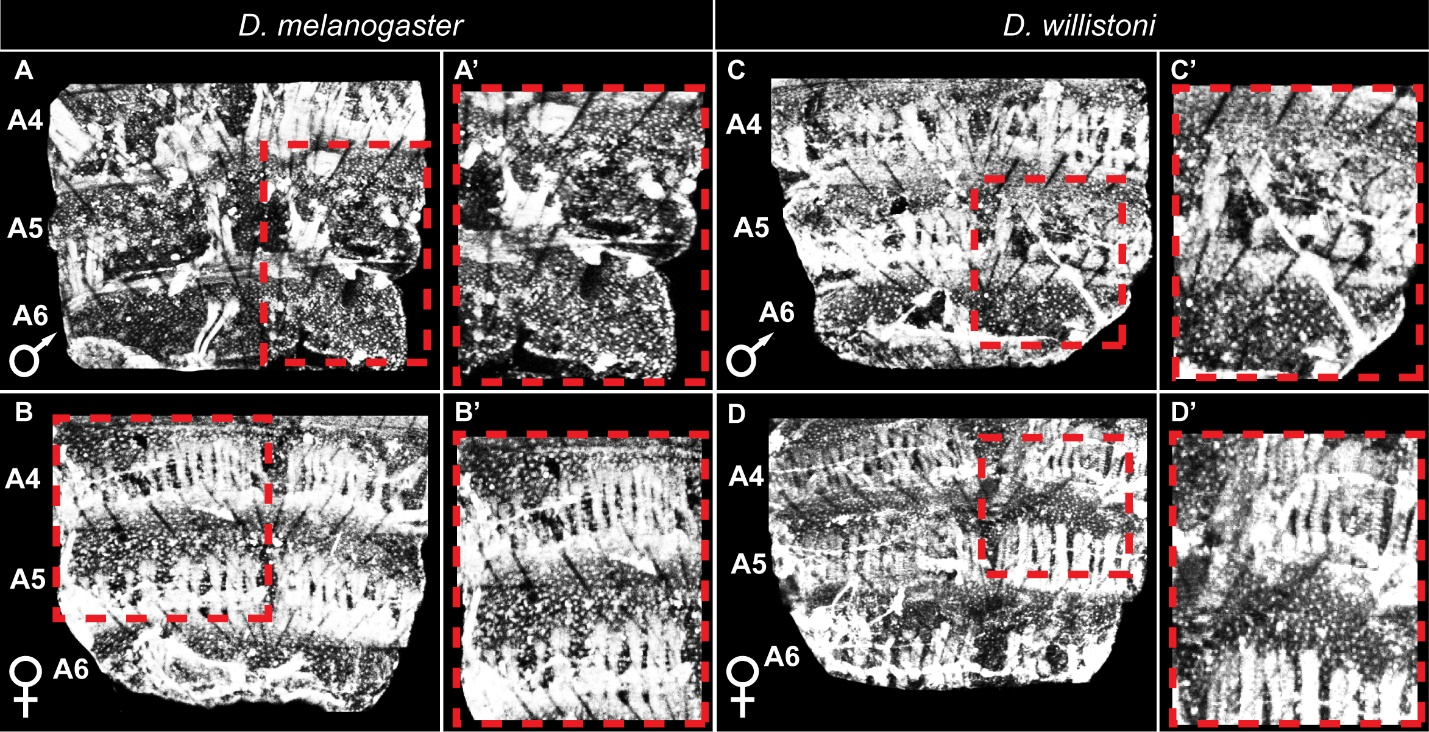

Supplement: S16 Fig — Trx protein expression in the abdominal epidermis of (A) male and (B) female D. melanogaster and (C) male and (D) female D. willistoni at the P14-15(i) developmental stage. Dashed red boxes in A-D indicate the zoomed in regions shown in A’-D’. (TIF) [file pgen.1010653.s016.tif]
