# Supplementary material for: A novel role for trithorax in the gene regulatory network for a rapidly evolving fruit fly pigmentation trait
Source: PLoS Genet. 2023 Feb 16;19(2):e1010653. doi: 10.1371/journal.pgen.1010653 (PMC9977049; doi:10.1371/journal.pgen.1010653)
Supplement: S1 Document — (DOCX) [file pgen.1010653.s017.docx]

*bric à brac* (*bab*) “dimorphic element” core region

GGTACCCTCGCTCTTTCTCTTTGCCATTTTAACTTTTATTACTCTTAATATAAAAAAGCTGGCTAGATGCGGGCCAGCTGTAAAAATGCACGCGGTCATAAAAAGTTGCAGGAGGCATGTTGCCAGTTGCCTGCAACCGGCAACATTCGCAGAACAGCAGCAACATCGTAAAATAACTTCTTGCTCTGCGGTCTGAGTTTGGCCGCAACAATGTTGCTGCATTTATTCGTATTATTATTACATTTTAATGAATAATTCTAATTATATGCAACTTGAATAAGCCCGCCGATGCCAATAAAAAGCGGCGTGGCAAAGTGGAGTGGACTGGGTTTGTGTGGCGCCCCTGCTAGTGGCACATAAAAATTGGCGCAAGTTAATTGTGGTAGTTATTTGCTGTTTTGCCATTTGGTCATTTTACAATTTTACCATTTCAGCCACAACTTTTCGCACTGCTCCCCCCCTTTCCCAGCACAACAATGTTGCGGCATTCTCGCACTTTACGAGGCGTTTTTTTTTTATATCACTTACTTTACTTAGTTGATTAAGGGCGTGGCCGATGGGCCAGATACATGCTTAGATTTGCTCCAGCAGTGGGCTGCATTTTACGACCCTCAAAACCCGATCCAAATGGAAAATATGAAAATACGGCTAATCCGCTTATGAGCACAA

*bric à brac* (*bab*) “anterior element” (ACEsub3 region)

GCCGGCAACACTAATACCCAGGCAAACAAACAAAGATGCAGATACAGATGCCGCGGCGGATTCAGATTCAGATGCAGATGCAGATGCAGATGCATTTTCCAAAGAATTTCTAAGCACATAAAAAGCAAAGCAAACGGTCAGTTGGGAAAATATTTAAAAAAGCATAAAAACAAAATATTTATTGAAAAGAGACGCTAACGAGCCAACGCCGCGGCGATCAGAGGCAAACAGACCGACAGACCGACAGACAGACGGCTTCAAAAATTGAGCACATGAGCAGGCTAATGCACATGCACATGTGTGCATAGCTGGCTATTTGATTAATGCACTCGAAAGCGACAATAAACACGGAACACGGAACATGGAACATGGCCAAGAACCGGCCAGAAAAGGAGTCGCACTGGGATCTCTTCTGCGCCCAGACAAACAAATATCGCATTCCGATTGATCCCGGACCGGGCAACTCCTCATCGAATGCATGTGACTCTGAATGGTGAATGGAGCTCCATCGCCAGAGATCTGGGCTCTTGAGTGAACGTCGTCGACGTCTATTGGATAAATAAATACAAATTTTTATTAAAGTGCGAATTCTGCGGTGAGCTCGGTGCTAATGCAGTCGCAGTCCAAATCGAACCGGTCGCTAAGCTAAGCTGACAAAAACTACCAGCTTACTTTAATAGTCGTAAATATTCAGATACAATGTTTGGAGTGAACTTATTGAAATATAATTATGAAGCAAAGCCGCATTAGGACTATAGATATCTTCATATGTATATTGCAAGCGCATCTTAAGATCAATATTTAATATTTTTTTTTTAATTTTCAATATCAGCATTTCAAAATTTAAAATATATTTTTATGCAATCATAATGATTCAATCTCAGAAAGAGAAAAATACATAAAAAATCCAAATAATGAATCTATCGATTTTAGCCCCAGATATTTTCTGTGCAGACCCATTCCGTCGTTGCCCTAATTGAAGCACACTTAGGGAACCACAACAAGTCACAAAATGC

*tan* Male Specific Element 2 or “t_MSE2”

TGAAATAATAATAAATAATCAGAATATAAATATATTATACGTTTTATAGATAGAATCAAGGCTTAGGATAATTGCACTAAGTAGTATACTTAAATTCCCATTGCCAAGTGAACCGGTTGGTATCCAAAGTTGAAGTCAATAACAAAAATGAGTGCATTTTACTCTTGCACCATTAGAATATTAGATTTTAGTGTTTAAATAAACTAATTTGAGAATTCAAGATCATAATATGTATACTAATTAGACAGTCTCTTTTTTTTATTACTTCAACTATTCAAATTTGCGTTTTTATTACTTTATAATTTTCAAGTGGTCTTGGTGCTTTCCAACTGCTAGGATTGAGTTGAAACA

*yellow* Body Element 0.6 region or “yBE0.6”

CTGTGGGTGCAATGATTTAGAATGCGGGCAAGGGATCAAGTTGAACCACTTCTAAGAAAAAATAGCATTGCATAAATGATATAGAGTCCAAAAACTACACAAATTCAATAGCAGTAATGGTTACATTAGCTTTGAAATTGTTTTTAGACATCCGAAGAAATAAGATTAAATTTAAACGGCATTCTTTAATTTGTATTTTAATATTTTGAGAGGTTTTCCTTATTTAAAGTGTAGATTATTGAGGATTAATGCAAACCACTTTATCTGCGGAGGTCGTAAAACGTATTTTTACCCATTTGCATGTTTATTATGCGTGTGGCTGGTTGTATTACTTTACTTAAGTTTTGCAATTTTTTCTTTAGCAAGCAGGTGCATTTGGGCCAAGAGATATATGCGATCGCTTTCGGTTCGAATTTTTAACATTTACTTGCGGCGATGGTCATTAGAGCATTACCCACTTAGGGCACCCCCAACATCCAGTTGATTTTCAGGGACCACAATATTTTAAATAACAGCTAGTGGAATTACCTAAAAGCGCTTTCGTCCCTTTTGAAATTTTATGTAACACTCAATTATATTTATGTATATGTATGCTCAAAATCACCTGCCAATAAC

*Dopa decarboxylase* Male Epidermis Element 1 or “DdcMEE1”

TTCTCAGTGTATGCGGAACTTCCCGCTCAAAAGGCTCAACCTAGCCCACTTCCCCTAGCACAATGCGAAAGTGAGTGAGAGCATTGGATTATTTGACGTCACAATTCCATGAGCGGTTCAAAAAGCACGTCATATGTGGTGCTCTATTAACCGGTTTCCAAGATGCGCGTAAAGCGTGCCATTCCACGGCTTAATCAATTTCTTGTCTTTCCTACGAATATAACTTTGTTTACATTTTTTTGCGTGATTTTTTCTTCGGGGAGTCCAAGAAAAACCCTGTTTCGAGTGACTCATAATTGGGGGATTCCTGACGAGATCGCTCTCTTTCCACAAATTCGAGTTGGGAAGCACGTGAGTAGAATTCAAAATGTTTTGCTTGCTGTTTTAAATATCACTAGGTTCTCAAACTAATTTCAAAAATAATCAAATTAAGTTCACAGAGCTGGCAAATAAAATGTAATAGCTTGCATGTATGTATATATATATATTTTTTTAAATTCTAAATAAATCCATGGAAAATAAAGCCTTTGATATCCAGTTACTGATTCAGCGCCCAATTAATGCATGTTCCAAAAAAGTGTCAAAAAACGTGCACAAATCAAACGAGAGCTGAATTTGTTTTTACGACAGCGGCTGCGATTCGAAGTTCAGCGGCTGCGGACTGCGATTGAACCGGTCCTGCGGAATTGGCAGCGCTGCTGGACGGGCTTTAAAAGCCATGGCCAAGAGCGGGCAGCGCTCAGTTAAGAGGAGAACGCCAAGCGCACAGCAATCAGCACCGAAATATCAGCATCGAAATATCAGCAAATAAATATTAGCTGTTCTAAACCAGAAGGGCAAACTGAACTTAGAGCAAAGATTTAGTTCGGAACGGAAGTAAAGCTCGGCAACAAG

*yellow-C* intron element

TTTGTCACTGTGCCAAGGTAAGTAACCAAATTCATAGACAAAAAAAAAAGAGACAACAA

ACTTACGGTAAGCTGTAGTTAGGTTTTCTTTTTTTTTCTTATTATTTACCTTTTTTGTTGGCTTGGTTTGTAATTAAATGTCAAGCTAGACTTGCAAAATCATCGAGCGTCAGGAGGGGAAACCGGTTTCCTCGCTGGCTAGTTAACTTTATCATTTTTTACCCCATTTGTGTATGTCTATTTTGCATTTTAAATCGTGGTTTCTAAATGCAAATCGTAACATGAGGTTAATTATAAATTAATAAGAGAAAACACATGATCGGAAAATTCTATGGAATATTATGGATCCCATAAAAGGCTAACTAGGTAACTAGTAAACAAAGTCTTTGTGACGTCACCTTTTTGGGATTTACCACCGATGCGCTTATTGGCCAAAAAGCCCCGAAAACCATTCTGCTTTTCGGGAGATATGAGTAATCAAAACAAAATTCGATTTAAAAACATTTCGAAAAATAAGATACGTAAAAAAAGTATATTTTGTTGATAATACAGCATAATTATTTAAGCTATAGTTGCCGTCAAAGTTTTAACTGATAGTGCTAAGATATAAATTAAAACTATAGCTTATATTAGACTATATCCAATCAGTCATAATAATTGCGCAGGTGAATGGTACTTTTTAATGTTATTAATTTGTTACTTGCCAGTGTCAGGTTTAAATGTAATTAGTAACTAAACAAGTCTGAAATGTAAATTTTTTAGTTAATTTATTTCTCCTCATTATTAAATGATCTACTTTCCCTTTAGATGGAAGGCTG

*ebony* activation element

TCTGGTAATTCAAAAACGCCTGTGCCCGTTCGAATCGGTTCTCAGGTGCTTTTTATTACTTTTTGATTAAGTAGATGCAATCAGTGCGGAAAGTTGATAGCGAGTATATCTTAATAATCCGATCTTTTCAATTAGTAAATTAACATAAGTCTGGTTTTGAGTGAAACTTGATAGACTTGAATAGTGATCAGCTGGTGTGGCTGCAACTTGTCACCATTAATATATGGTGTGGTAAATCATGAATGCATCTTTAATGGTAGTGTAATTAATCGCATAATTTCAATTTAACACATTTTTATTCTCGTAAGTCGTAGATTAAAAATTATGTAACAGATAGGATAGAGGATTTCAGTTCCTATAAAGTATAAGTAATCTTGGTCTTGTTTGTCCGTATGAGCATCCATATATCAGAAATATGGATTGTTTCAAACAACGTCCACACTTTTTAAAAAATGTTCCATTTTCTTTTCATTTTATAATTTATTATCATTCATTCATTTTATTATTATCCCAAGTTTTGTCAATCCATCAGTAAACAAGTCGGCTAGAGATGTTGATTAAGAAGAGCTTACATTTATAAATACAAAATACGAAATTAATATCGTAGCTGCCTTTTCCTTATAGGAATTTAATTATTTTTACGACTTACCAATTTTTTTTTTTATTTTTAAACGGCATACATATTCCAAAATCAATTCATAAGGGTAGAGGCTGTAAAAGTCATTGTACTAAGCTTTGCAAGTTCTATGTTTCTATGTTTCTAACGGATACTAATC

*ebony* male repressor element

AAATCTCCTGACTATTACACAGATTGTTATTACTATTAATAATTATTTCCATTTACAATGAAAAATCAGTTCTTAATTCTTGAACTCACAAAGTAATTGCTGAATTGGTCAGTCTGTCATATTTCTGCTATTAAAATCAAATTGATAAAAATGCAAATGGTAATGATGGCAATCGACAGCTCCAAAGTGTTAGCCCGCATCGCAATTTGGATTTACTGGCGTTGATTCTTGTACGAAACACTCA
