# Supplementary material for: A novel role for trithorax in the gene regulatory network for a rapidly evolving fruit fly pigmentation trait
Source: PLoS Genet. 2023 Feb 16;19(2):e1010653. doi: 10.1371/journal.pgen.1010653 (PMC9977049; doi:10.1371/journal.pgen.1010653)
Supplement: S2 Document — (DOCX) [file pgen.1010653.s018.docx]

***bric à brac* (*bab*) “dimorphic element” core region**

GGTACCCTCGCTCTTTCTCTTTGCCATTTTAACTTTTATTACTCTTAATATAAAAAAGCTGGCTAGATGCGGGCCAGCTGTAAAAATGCACGCGGTCATAAAAAGTTGCAGGAGGCATGTTGCCAGTTGCCTGCAACCGGCAACATTCGCAGAACAGCAGCAACATCGTAAAATAACTTCTTGCTCTGCGGTCTGAGTTTGGCCGCAACAATGTTGCTGCATTTATTCGTATTATTATTACATTTTAATGAATAATTCTAATTATATGCAACTTGAATAAGCCCGCCGATGCCAATAAAAAGCGGCGTGGCAAAGTGGAGTGGACTGGGTTTGTGTGGCGCCCCTGCTAGTGGCACATAAAAATTGGCGCAAGTTAATTGTGGTAGTTATTTGCTGTTTTGCCATTTGGTCATTTTACAATTTTACCATTTCAGCCACAACTTTTCGCACTGCTCCCCCCCTTTCCCAGCACAACAATGTTGCGGCATTCTCGCACTTTACGAGGCGTTTTTTTTTTATATCACTTACTTTACTTAGTTGATTAAGGGCGTGGCCGATGGGCCAGATACATGCTTAGATTTGCTCCAGCAGTGGGCTGCATTTTACGACCCTCAAAACCCGATCCAAATGGAAAATATGAAAATACGGCTAATCCGCTTATGAGCACAA

***bric à brac* (*bab*) “anterior element” (ACEsub3 region)**

GCCGGCAACACTAATACCCAGGCAAACAAACAAAGATGCAGATACAGATGCCGCGGCGGATTCAGATTCAGATGCAGATGCAGATGCAGATGCATTTTCCAAAGAATTTCTAAGCACATAAAAAGCAAAGCAAACGGTCAGTTGGGAAAATATTTAAAAAAGCATAAAAACAAAATATTTATTGAAAAGAGACGCTAACGAGCCAACGCCGCGGCGATCAGAGGCAAACAGACCGACAGACCGACAGACAGACGGCTTCAAAAATTGAGCACATGAGCAGGCTAATGCACATGCACATGTGTGCATAGCTGGCTATTTGATTAATGCACTCGAAAGCGACAATAAACACGGAACACGGAACATGGAACATGGCCAAGAACCGGCCAGAAAAGGAGTCGCACTGGGATCTCTTCTGCGCCCAGACAAACAAATATCGCATTCCGATTGATCCCGGACCGGGCAACTCCTCATCGAATGCATGTGACTCTGAATGGTGAATGGAGCTCCATCGCCAGAGATCTGGGCTCTTGAGTGAACGTCGTCGACGTCTATTGGATAAATAAATACAAATTTTTATTAAAGTGCGAATTCTGCGGTGAGCTCGGTGCTAATGCAGTCGCAGTCCAAATCGAACCGGTCGCTAAGCTAAGCTGACAAAAACTACCAGCTTACTTTAATAGTCGTAAATATTCAGATACAATGTTTGGAGTGAACTTATTGAAATATAATTATGAAGCAAAGCCGCATTAGGACTATAGATATCTTCATATGTATATTGCAAGCGCATCTTAAGATCAATATTTAATATTTTTTTTTTAATTTTCAATATCAGCATTTCAAAATTTAAAATATATTTTTATGCAATCATAATGATTCAATCTCAGAAAGAGAAAAATACATAAAAAATCCAAATAATGAATCTATCGATTTTAGCCCCAGATATTTTCTGTGCAGACCCATTCCGTCGTTGCCCTAATTGAAGCACACTTAGGGAACCACAACAAGTCACAAAATGC

***tan* Male Specific Element 2 or “t_MSE2”**

TGAAATAATAATAAATAATCAGAATATAAATATATTATACGTTTTATAGATAGAATCAAGGCTTAGGATAATTGCACTAAGTAGTATACTTAAATTCCCATTGCCAAGTGAACCGGTTGGTATCCAAAGTTGAAGTCAATAACAAAAATGAGTGCATTTTACTCTTGCACCATTAGAATATTAGATTTTAGTGTTTAAATAAACTAATTTGAGAATTCAAGATCATAATATGTATACTAATTAGACAGTCTCTTTTTTTTATTACTTCAACTATTCAAATTTGCGTTTTTATTACTTTATAATTTTCAAGTGGTCTTGGTGCTTTCCAACTGCTAGGATTGAGTTGAAACA

***yellow* Body Element 0.6 region or “yBE0.6”**

CTGTGGGTGCAATGATTTAGAATGCGGGCAAGGGATCAAGTTGAACCACTTCTAAGAAAAAATAGCATTGCATAAATGATATAGAGTCCAAAAACTACACAAATTCAATAGCAGTAATGGTTACATTAGCTTTGAAATTGTTTTTAGACATCCGAAGAAATAAGATTAAATTTAAACGGCATTCTTTAATTTGTATTTTAATATTTTGAGAGGTTTTCCTTATTTAAAGTGTAGATTATTGAGGATTAATGCAAACCACTTTATCTGCGGAGGTCGTAAAACGTATTTTTACCCATTTGCATGTTTATTATGCGTGTGGCTGGTTGTATTACTTTACTTAAGTTTTGCAATTTTTTCTTTAGCAAGCAGGTGCATTTGGGCCAAGAGATATATGCGATCGCTTTCGGTTCGAATTTTTAACATTTACTTGCGGCGATGGTCATTAGAGCATTACCCACTTAGGGCACCCCCAACATCCAGTTGATTTTCAGGGACCACAATATTTTAAATAACAGCTAGTGGAATTACCTAAAAGCGCTTTCGTCCCTTTTGAAATTTTATGTAACACTCAATTATATTTATGTATATGTATGCTCAAAATCACCTGCCAATAAC

***Dopa decarboxylase* (*Ddc*) Male Epidermis Element 1 or “DdcMEE1”**

TTCTCAGTGTATGCGGAACTTCCCGCTCAAAAGGCTCAACCTAGCCCACTTCCCCTAGCACAATGCGAAAGTGAGTGAGAGCATTGGATTATTTGACGTCACAATTCCATGAGCGGTTCAAAAAGCACGTCATATGTGGTGCTCTATTAACCGGTTTCCAAGATGCGCGTAAAGCGTGCCATTCCACGGCTTAATCAATTTCTTGTCTTTCCTACGAATATAACTTTGTTTACATTTTTTTGCGTGATTTTTTCTTCGGGGAGTCCAAGAAAAACCCTGTTTCGAGTGACTCATAATTGGGGGATTCCTGACGAGATCGCTCTCTTTCCACAAATTCGAGTTGGGAAGCACGTGAGTAGAATTCAAAATGTTTTGCTTGCTGTTTTAAATATCACTAGGTTCTCAAACTAATTTCAAAAATAATCAAATTAAGTTCACAGAGCTGGCAAATAAAATGTAATAGCTTGCATGTATGTATATATATATATTTTTTTAAATTCTAAATAAATCCATGGAAAATAAAGCCTTTGATATCCAGTTACTGATTCAGCGCCCAATTAATGCATGTTCCAAAAAAGTGTCAAAAAACGTGCACAAATCAAACGAGAGCTGAATTTGTTTTTACGACAGCGGCTGCGATTCGAAGTTCAGCGGCTGCGGACTGCGATTGAACCGGTCCTGCGGAATTGGCAGCGCTGCTGGACGGGCTTTAAAAGCCATGGCCAAGAGCGGGCAGCGCTCAGTTAAGAGGAGAACGCCAAGCGCACAGCAATCAGCACCGAAATATCAGCATCGAAATATCAGCAAATAAATATTAGCTGTTCTAAACCAGAAGGGCAAACTGAACTTAGAGCAAAGATTTAGTTCGGAACGGAAGTAAAGCTCGGCAACAAG

***ebony* (*e*) activation element**

TCTGGTAATTCAAAAACGCCTGTGCCCGTTCGAATCGGTTCTCAGGTGCTTTTTATTACTTTTTGATTAAGTAGATGCAATCAGTGCGGAAAGTTGATAGCGAGTATATCTTAATAATCCGATCTTTTCAATTAGTAAATTAACATAAGTCTGGTTTTGAGTGAAACTTGATAGACTTGAATAGTGATCAGCTGGTGTGGCTGCAACTTGTCACCATTAATATATGGTGTGGTAAATCATGAATGCATCTTTAATGGTAGTGTAATTAATCGCATAATTTCAATTTAACACATTTTTATTCTCGTAAGTCGTAGATTAAAAATTATGTAACAGATAGGATAGAGGATTTCAGTTCCTATAAAGTATAAGTAATCTTGGTCTTGTTTGTCCGTATGAGCATCCATATATCAGAAATATGGATTGTTTCAAACAACGTCCACACTTTTTAAAAAATGTTCCATTTTCTTTTCATTTTATAATTTATTATCATTCATTCATTTTATTATTATCCCAAGTTTTGTCAATCCATCAGTAAACAAGTCGGCTAGAGATGTTGATTAAGAAGAGCTTACATTTATAAATACAAAATACGAAATTAATATCGTAGCTGCCTTTTCCTTATAGGAATTTAATTATTTTTACGACTTACCAATTTTTTTTTTTATTTTTAAACGGCATACATATTCCAAAATCAATTCATAAGGGTAGAGGCTGTAAAAGTCATTGTACTAAGCTTTGCAAGTTCTATGTTTCTATGTTTCTAACGGATACTAATC

***S1.1 Histone deacetylase 4* (*HDAC4*) intron 1**

ACATACGAAGAGTGAGAGCGAGAGAGCGTGCGTTATTTATTTTATATTTTCGTTAATCATTTTTCGCATTTTGTGAAACAAGTTGGAAAAGCGACAATACAAAATACAGCAACAACAATAGCAGCAGTAAGCAGTTTGCACCACTTGGAAGAGGTATTTATGTTTGTTTGTTTGTCTTTGCCCCGTGTATTCTTATTAGCAGTCGTTGAGAGAGGGAGGAGGCACAGCATGTTCTTATTAGATCAGCCAGACTTCTTTTTCGTCTGTCTTGCTCCTCACTCGATTCGCCGTTATTTATGTAACAAGTGTGTGCGTGCTAAATGAAGTGCTCGCGCTCTCTTCTTCCCCCCTCTCTTCATACGGTTTCTGTTGCTCTCTCTCTCTCGCTCGCACTCTTTGCATTTGATGATTTGATTTTGTTCACTTAGTAAAGTTGCTGTAATGTGGCTGCCACCCAAAAAAATAAATAAAAAAAAAACAAGAACAAAATAATGAAAATGAAGCAAAAATAAATAATAAAAGCTCCACCGCAAAGCGCATTGAACTGGTTTATGTTGTGCTCGCTTCTTCTGCTCTCCTCATTCTTCCTTTACT

***S1.2 enhancer of yellow 3* (*e(y)3*) intron 4**

TCGTAGTCTAGGACATAACAGTTCATTCTCATAGAACGGTAGGAATAATTATACGAGTGGTACTTAACTTACTTTTACTTAGAATTGCCAATAACAGAATGAAACCCTGAAACCCTACGAAGTAAAAACTAACCATTTGTAAATACAGGGTAATTGCTCAACATGCATACATGCGTACATATGTACACATGTGCGCATAATTATTTATCCTTGCAATTTTTGGAAGCCATTGCTCTTTGCACATGTTTAAACGTGCAGCAGCGAAAACGGCGAGCGCAGAGTTGCCAGCTCTCAACGAAGTTCACATTCAAAGCCCCGCTGAGAGAGTGGGCAAGAGAGCAATCGAGAGTGAGAGCGAGTGCAGCAGCAGCAGGTGTGGTGAAGGTGGACTACTGTCAGCTCATTTACCCACACACATGCAAAGACACAAACACACACACGTGCGATTGGCGAGAGCCAAACAAAAACTTCAGCTGATTAAAATCGCAGGTGAAGCCAACAACAATCACAGCGGCTGCGCTGCTCAGTTCGCAGCTGCTTCCTTCGCCGCTCCCCCCCTTAACCGGCACGCCACACGCCCCCAATAGCTAATGCAGCAGCGGCTCGCTTGGCTTTATTGTTGTTGTTGG

***S1.3 Muc4B-CG43134*** **intergenic region**

ACTTCGCTATTTGCCTCTCGTCACGTCCCAGCTAAATAATTACCAATTTCAGCTTTTAAAACGGGCTAGGAATGACATGTAAATACATAATTAAAAATTGCGTGAGTATTTTTTTTTTGTTTTTTTTTATGCTTTTTTGAACACCAGCAATTAAATAATTCCCATTGCATTCGCCGCTGTTATCAGAGCTAATCGGATGTCGCATTGTCTGTTTACATTTCATTCGATTTATTCATAGTGGTTTTTTGAATCACTTTTTATTACTTTTACAATTAACTGGAATTAGTTAAGCGTTAGATATATTTATATATCTTTTTTTAATTAATAGCTGTTATACTACCTTTTACTAAAATGTTTTTGTATGTTCAGGAAATAACAAAGTGCTGGTAAATGCATAGTGTTCAGGATTAATTTGATTTTTTTGTTTTATGCATTTTTTTTGGGAGGAAAAAGTTTACAAGCCTTAATACACTAAACACTACTGTTTACTAAACTAAATTCACATAGCATAATTTTTCCAAAACTTCATTAATGAACGTATTTAAACGCATTGCATTCAAAATTGTTTGTTTTGTGTTTTACTTCGACCATCTGTTTTCGATGTTGTCCAAGATTACGTGCCCGGTTGCTAATCGAATGCAAGAGCAATGCATCCGGTTCGTCTGTTCGTAGTA

***S1.6 Sp1-CG1354* Intergenic region**

GGCATGATTTTATAGCACCGATTTTCCGATTGTATTATAGATAAGTAAAGTTCAACTGGTTTATAAAGAGATTCCAGCAGATCTGGCGAATCTGCAGAGGCAACTACACTCTGAGAAACCCGCCTTATCTCCTGCGGGCAAATGGCGCATAATTTCCATTTGGAACGGCCATTTTTGCAACAGCAATTACTTTGGTAGCCTGAACCTGTCTCGGGGAAGCCCCCTGCTCCCCCAAACAATTTTCAGCGAACGCATTGCATTCGTTGTAGTTTTTGTTGTGCGAAAAAAAAAGCGGCAATAAAAATTAAGTTGAAGTTGAATTTGAAATTCAGATGACGACGGCGACGGACGACGACGACTTGCCCAATTATTATTAATAATAGCAACAACAGCAGCAGCGGCAGCAACTTGAAGAAGCAGCAGCAGCAGCAGCAAGTTTCAGCAGGAAAAAAAATGCAGAGGAAAAAAGTTAATAGGACCGCATTCATTAAATCCATTTAATGCACAGAGAGGCAAATTATTGCGCTAAAAGAAAAGCAACAACACACTTGGAAAAAAAAGGCCAGCAGAAAAAACGAAATAATTGCAGTTGGCTGAACACCCCGAAATGGCCAAGAAGAAGCAGAAGCAGTACGAAACGGCGACGTTCGGTCGGCGGCCATTAAAATGGCCAACGAATTGGCCAAAAGTGGGTTTGGAAAGGGGCACAGGCAGGGGGCGATTGGAGTTGCCAAGTCCAACTGGCCATGTAAACTGGCTAAGCGGCCTATGCGAGCGAAAGAGACGAACAGAGGCGAATAGAGCGAGAACGAAAACCGGTAGCTCCACTTTAATGAGCGCCAAACGCAAAGAACCAAAAATTCCAGGCAAAAGGAGCAGCAGCCCCCCAAAACCATAGGGTACTCCAGGGGGCTATTCCTGAAGGTCTTTT

***S1.8* *Kruppel homolog 1* (*Kr-h1*) upstream region**

GCTGGATTAAATGCTGTCTGGGATCGGATGCCAGAACATTTTAGCACATATAAATGGTAAATATTAATTATAAATATAGATTACATATAAATTAAATATTACGAGTGAACTATTTGAACTATATTTTAACAATTAATAATCCAGCAGTTTCAATTTCTTAAGCATGAATATTAAGCTCTAATGTTTACTTTGCAATCGATTTAGGGGGCAGCTATTTATCTGCATTTTCGATCTGAATCTAATCAAATCGACGACCCTTGCACGGTGACTGGCAAATCAGCGGCTATCTGAAACGGCCATACTCTTTGTTAACAAATCTGATGTTCTGACCATTCCAAACGTCGACATTTTTTACGTGTGCGGCGATATCATAATAAAAATTTTATTGAGTAAGTTGAGCGGTGCCAGCAGAGGCAGAGGCAGCAACAACCATTGCAAAAAAAATACAAAAACTGCGAAGGCGGCGAACCACCTTTATTAACTTATTCTGTGCGTGTGTGTGTATAGCAGCTGCGGCAAATGCCGGTTCACACACACACACGCATATCGACTTTCGTTCTGCGCTGGCTGCGCAGCCGACGCCGCTGCCACCGCGAGCTTTGTTCTCGTCGAACTTTTCGGTTCTCGTATTTCTCG

***S1.9 twist* (*twi*) intron 1**

GGCTTTGTGGTCCTCTAATGTGTTAATAGGAACCGTACGCCGTACGCGATGCCGCGACTGCGGCGCTGGGGGAGATTGCTCAATTGTTTTTGCTTTTTAGAAAGGCCTCTGGTGTAGTAGGCTAATTTTTTTTGCTTGCTCGCGAGTATTGGATTTAATTTAATTTTTGTGCTTTTCATGCTTCGTTAACATATTTTGCTTATAAGGATTTCCCGCGCGCAGCAAAAAGAAATCACAACTGATCAGTGAACATGCCGCCAGGCAGCAAATGTGGCTGCTGCGGCTTCATTGTTGCTGCAGGTGCATGTTGCCCCAACAATGCCATAAATTGTGACATTGGTTTTTGCTCTCCTCCCCTCCCTCCCTCCTCGCACTCACCCCTGGATTCCCCCAAACTCCCGCTCCTCAAGAAGATATATGCACACACACACACACACAATCACATAGATACTCTGATCTGGTGACCAAGAGCAAAAACTTAAGCCTGGCTAATTCTTTTTTGTAGCCCAGACCGCTGCAGGGGCAAAAAGCTTAGAACGAGTTTTGGCATTTACGACTCACTCACTCTGGGCTCTGGGATCTGCACCACCCCCTCTATTGGAGCAGCCCCCCTCCCTCGCAAATCTGCGGCAGTGGCCAACTGCCACCGCTGCTTTTTTCTGTTCTCGTTGTTGTCGCTTGCCGCTTGCCGCATTATGTTGCTGCTTAATTGGTTATAAATTAATTAAACGTCGATTAATAATCCGGCTTCTTGTCAGTTAAAGCCGCCGTCCTCGCCGTCGATGGTTGGTTAGTTGTAGGTTCTGGGCTTGGGAACAAGAACGCTGCAGTCGTAG

***S1.10 klumpfuss* (*klu*) upstream region**

GTTTATTTTCCCTGCCCACAATCACACACACACACACTTAGAGATAGTGGTGTGGGTTGAGTATCTGCGTGTGCGTGTGTGTGTGAGTGCGCCACTATTCGTTCATTCACAAATTGTGTTGTTGCTGCCTTGGCATGCTTTTGCTTTGTTTTTTCCTTTTTTTTTTGGTTTTTTCTCAATTCAGTTTTGTTTTGTAAACACACACATACACACGCACACACCTGGATGGAGGCGCACACAGATACACTGACACCTCGGCAGCTGCGCCACACACACAACCGCACACACGGTGGGCAAAGAGAGAGAGAGCGCGCTCTCTCTCTCTCTCGTGCATCGGCATTTCAAGTTCAGATATCGTATCTGTGAAGTTGTTTTGCCGGCATTTCGATTCTCGCGAGTGCAGAGAGAGAGACCGAAACTGTATCTGTGTGCGAGCGAAACGTGTGTGTGGTATAGGTGAGAGAGTTTATCCGGCGATTTTATCCGGGCCTTCAATGCGGCTCAAAATCAGTCAACTGCGATTTTCTTATAATGCAAGTTGGCTTATATGCATGTTAGACAAAAATAACTTCATGGGTACAAATGAAAGTCTAAAATTAAACAAATATATATTTTAGATCATTTTGTAGCGCTTTAATTTTTTTGCGAAGTTAAACAAAAAAGAGTTCCAATTAAGAATATTATATTATTTTTAAAAGAAAATAAGTTCAAAGCCAAGAATTATTAAAAACTAGACTAATTAAATTTCAATCAAAAAGTATTGAATTTACATTTTTGCTTTAGAGAATGTTTGAGTTTTAAACTGTAACTAAAAACGCTGCTATTACTATTTGTAAAACATTTTAATAAATTATATATTTGAAATTGTTTTGTAATTGTTTCTAAGATTAGAAGGCCCTTAAAGTCGATAAAGTACTAAACGCAT

***S1.11 Ecdysone-induced protein 74EF* (*Eip74EF*) intron 2** GACCTTCAGCTAAGTCACTAGATTTTATAAAAAATATATAATTAAACTTAAGCAAGGGTCTGATTTAGAAAAGAAAGTAAGGATCTTAAATACAGTTCTCTATACATTGACTATACAAAATGGCATTAAGAATATGTAGAAACATTGTTTCAACTTCAAAACATTTGTACATCATTAGTTTACGACTAAATAAAGCACAAAAAGTGGGAACAAATAAGATATCTACTATTATCAATAACAAAAAAACAGATCTTTCGTTTATTATCTCAGCTGTTAAATATGACTGTTATTACATATTACATATAACTAAGCAAAATGAGAATAACATTATTATAAATGTTACCTTAAACAAATTCTTTAGTCTATACTAAGTGAAAATAAACTATCCGAACTGAAAGAAGCTAAGAGATACGAGACTTACTAAATAGGAGGAGTAAAGAGCCGAAAGAAATAATAACAACCCAATTACAAAACTCTAATAAATTCATCTGAATCCATAGCATTTTATAAAAGAAATTACATCTGCTATACAAGCAATAAATAAAGGAAAGCATTAAACATCTTTGCAGAAGCAGTATATCATGTAGATATTAGCATTAGAAGTCGAAAGAACAAAAACAATAGCAACTTTGTGTGCGGCACACGAGCAACAAACACACGCAACATGCAACATGCAGCAGCATCAGCATTATGAGCATTAGAAACGGCAGCAACATCAATGGCCGCAGTGCAACACATTCAACTTGCAGCACAGACATTACAACATTGCGCTGCCTGGCACCCGCGTCATCATCATTTCTCTTTCTCCCTACCTATCGCCACTTTTCT

***S1.13 GATAe–pnr* intergenic region**

TCTCTGATGGTTATCTTATGCTATTTTTTGATCATTTCGAGCTCTAAAAGATTGCATTTGAATTAAGTGTATAAACGACATCCTAAAGTAATGAAAATTCATAATCTTTTTCCGCCCTTTTGCATGAACAGCCGTCTTGAAAGGAGCTACTGCTACCGCATACGCTTTTTAATTAGCCCCGTCAGACGGACACATAAAGTATATCGGAATCGCAATTGTATTTATGCTTTCATAACTCGGCAATCCATAATAAGCGTACCACTAATAGCTGCCGCACCTGCAGCCAGCGGCACATCCTCTGCAGCAGCGAATTAAAACATTAGCGACCAACCTGTTTCATTTCCCATTTTTCCTGGCTAAAAGCGGGGAGCGGAGGGCTGGCGAGACCGTAAAATAATAATTCAATGTGACAGGCAACAAGATACGACAGCGGGCGCCATTTTTCAGATGCCACAGCGCCGAACTCCCCCTCGCCAGCGATCCCCTGGCACACTTACTATACAAGTTGCAGCGCACTGACATTATCGCCGGAATTATGACAGACGTACAACGGCGCAAGAAATGGGGGAATCACCGGTTGGGTGAAGGGGTTTTGGCAAGAGCGATCCGCTAATCTTCGGAGCCTTGCATGCATTTGAATTTCTATTAAGTTAATTGCAAATTGGCCAAGGCGAACGCGCATTTGTTCATGTCCAATAACAGTCGAGTAACG

***S1.17 headcase* (*hdc*) intron 2**

**GGCGCGCC**CACTATCGCTGTTTCATCTATCGCCCGCTCCATCGCACACTATTGGCGCTCTCTCGCTCCACCCGTCCCGCTCTCGCACGCTGACCGAGTGCATTGCACTCATTCCTCTGCTCTGCGCTCCACTGCGACGTCGGCAGCGCTGCTCGCATGGCAAATCAAATAGTGCAACAACAATGGCAGCAACAACAACACGGCGCAGCATTCGTCATCTGTTGTTTTTGTTGCCGCTGCTGCTGCTGCTGAATTTGACTAATTGCATTGAGTAGAGCGATGTTGTTGTTGTTGCTCCTCTAGCTGCTTTTGATGTTTTTCGCTGATTGTTGTTGCTTTTTATGTGCAACATGCCGGCACAATTTGTAATTAGTCGCTAGTTGCCTGACTTGTAATGTATTTAATGCCTAATGCTTTTCACATAAATTTGCATTGTTTTTCTCCTTTTTAATCTATTTATCTGCTTTTTATTCCCTTTTTTATTTCGCACAGCGGATGTTTAGCATCGTTTTTTTTTTCGTATGTGGGAGAAAGAGAGGCATTGAATGGAAAGATCTGGCACACCGAAAAAATTGTAAATTCGAATTCGATAAAATGTACCGAAAAACAAAAACACCGCTGGCCATTCGAATAGTTCCCACTGAGGGTGGGTGAATG**CCTGCAGG**
