# Supplementary material for: A novel role for trithorax in the gene regulatory network for a rapidly evolving fruit fly pigmentation trait
Source: PLoS Genet. 2023 Feb 16;19(2):e1010653. doi: 10.1371/journal.pgen.1010653 (PMC9977049; doi:10.1371/journal.pgen.1010653)
Supplement: S3 Document — (DOCX) [file pgen.1010653.s019.docx]

**Synthesized and subcloned sequence from *D. willistoni* and orthologous to the *D. melanogaster* *S2.20* *trithorax* CRE. Sequence is flanked by *Asc*I and *Sbf*I restriction sites that were used to subclone in the same sites of the S3AG reporter transgene vector.**

***Asc*I**

**ggcgcgcc**GGATAGGGCATTGCAAAGTCGTTTATGGTTTGTTTATCATAGAATTAGCCTACGGCCCATTGGCTGATGATGTGGATGTTGTGTGTGTGTGTGTTTCAATTTGTTTTTGCATTTGAAAGGCACAAATTTTTAATATGCGCAATTACTCATTTTACGCCTCAATGTTATTTGGCGCGTAGTTTCAAGCACACACACACACACACACAGGCAGGCGGCACACAACATCAACAACAACAACTACTCGAAGATTTGGAATTTGCCTAGTAGATACACAAATATATTTTGTAATTATTCATCAAGATCATCATCATCGTCGTCGTCGTCGTGAGTGAGGCGCGGCTGCTGCTTTATTGTATAATTTATTAAAAACTGTTCAACAATCAGCTCAATTTTTAGTTATACAAACGGGGCAACACATATAGACAAAAACACACACACAGCCATAGAGAGAGAAAGACATAGCACAGACAGGGATGAGAGAGAATAAGAGAGCAAATGAGCGCACAATTTGATTTAAAATCAGGCGATGACAACAAAAACAAAAACAATTGCCATTAAACAGTATTTATTGATGCTGTGTGCCCTTGTGTGTGTGTAAAATATTTTATTCAAGGACCCCGACAAAATGATACTCTTTATCAAATAGTAAAGAATTACCCAATCATTTCTATTACCATTTGACTACATGTTTATTGAAATTTAAGATATATGTGGGAAAAACTGAACAAGTAGCCGCCCATAATCGAAGCATATTAAAAATATTATTAAGTCTTTTTGCAATGATAGTATCAGGTGCACTCTTGCCTTTTACCCAATCATTTTCTCTAAAAAAAGACTACAAATTAAATCATGATCGAATTGACTTGATGTGCATTTAATTTATAACGTTGTTACACCTTTTGCCAAGCTCGTACATAAGATACTTATGTGAACATACCCTTTCTTTATACCAATAATTACTGAGTGCATTAAAATGGCATTTCAATATAACAGTATTTGTTGTAGATTTGTTGCTATTGCTCTTGCAGTGGCGGCCTTAATTTGCATTTCACAAAGGTCGGTCGCACCTCGCGGTGGGTCGATGCAAACGAGTGAATGTATCTCGCTACTGTTAACTACTACAAACATACAAAAATCAAATTCATATGCATATATACGGACATACATACATCAACAAATGGTTTTTATTAGTTCCTAATGGATACAACAATTTCTACCAGTTAATTAGTTGGATTAGTTTTGAAAATCTTATACGAATCGTGTGAC**cctgcagg**

***Sbf*I**

**Synthesized and subcloned sequence from *D. yakuba* and orthologous to the *D. melanogaster* *S2.19* *trithorax* CRE. Sequence is flanked by *Asc*I and *Sbf*I restriction sites that were used to subclone in the same sites of the S3AG reporter transgene vector.**

***Asc*I**

**ggcgcgcc**CTCATTGTCAAGGTGAAGAGATAGGTAATAAAAAAAACAATGAAAAAGTTTTTCTTAAATGAAAAATTAAAACAAACGTCAAAATACGAAATTTTTATGCTGTTGTTAATCCAAATAAGACAGATATTTTTGGATGAAAATATGTGTAATAGACTTGATGTTCAAAAGTTTACCAAAACGTTTAATTCGAATTAAATAAAAGAATTTCAAACGCTGTTAAGTATGCAACAGATCTTTAAAGGACATTTCACAATTTGGCCCAATTTAGCGGTAAAACAGCCTAGCTGCCAGCACTGGCTTACACCACCTTTACACGCTTTGCCACCACTAAGTGTGTGCAGTTAACGAAAAAACAAAAAAAAATTGAAGCAAAACAACTGCCGCGTTGTGGCTCCTTAAACTCATTCCGTTTTCTTTGGCACGTAGCACGCGCTCTCTCTCTCTGTCTCTCTCTCTAGCAGACTCCTTTTTAAGAAATGCTCTCACACTCCAATAACTCTCCCGCTCTTTTTCTCTCGGCAACTACCTTTCTCCCTCCCTCCCGCGGGCTAGTTGTCATCGACGTTTTTAAGTGGTTTTTCTACTTCAGTCTTGAACTTTTTTGTTTCTTGTTGCTGTTGTTTTCTTTGCAGGATTTTCTCGTTCGGCTTTTGTTGTTGCTGCTGCTGCCTTTGTTTTTTTCTCTCGGATATTTTTTTGTTTGTTGGAAATTGAAATTCTTACAGTTGTTGTTGTTGTTGCTGCTGCTGCTGCTGATGCCGTTGTGGGCCACTATTAAGTGGTGGGTTTTCCCTACTGCTGGCTCTTTTTACGACAGTTTTTCTCTTTGTTCTCGCTTTTATGGCCTGATGAAAATTCTTCTTTTTGCGCGTTTAGTTTTTCTGCGTTGGGAGTTTCTGCTCGACGAATTCACAGTTTTCCACATTGCTGTACTTTCGCGGCAACTTTTCTCATCTGTAACTGCCACTGTTGCTCTTGTTGTTGTTGCTGTTGCTGCTGATGCTGGTTTGTGTTGGCGGGCACATGGTGCTCATTACC**cctgcagg**

***Sbf*I**
