# Supplementary material for: A novel role for trithorax in the gene regulatory network for a rapidly evolving fruit fly pigmentation trait
Source: PLoS Genet. 2023 Feb 16;19(2):e1010653. doi: 10.1371/journal.pgen.1010653 (PMC9977049; doi:10.1371/journal.pgen.1010653)
Supplement: S4 Document — (DOCX) [file pgen.1010653.s020.docx]

**Alignment of the partial *trithorax* intron sequences for the species whose S2.19 and S2.20 orthologous regions were evaluated for CRE activity in transgenic *D. melanogaster*. Forward primers and their sequence are annotated with the DNA sequence indicated in white font with black background color. Reverse primers are annotated with the DNA sequence indicated with red font with black background when the actual primer is the reverse complement sequence. The synthesized *D. yakuba* S2.19 and *D. willistoni* S2.20 sequences are respectively indicated by white font color on blue background and white font on purple background.**

D.wil. -------------TTGTTTTCTCCATCTCCTCCATCATCATCAGTTAGTTGAGCAGAGTT

D.pse. GCCCCCCTTTCGGGCACGTTGCAGCAATGCACTGCTGACCACAAAGGGACCGGCGGTGGA

D.bia. ------------------------------------------------------------

D.yak. --------------------------------TGGAGGGTTTGCCGGGAAACTTGGAGCG

D.mel. ------------------------------------------------------------

D.sim. ------------------------------------------------------------

D.wil. TTGCGTAGCACTCTTTTGATGGCCATTGTCGTTGTTTTTGCTACCGTTTACACCGTTGCC

D.pse. GTGGGCTGTCGTTGTTGTCCTCTTTTCGCCATTGTGGCTG-------------------C

D.bia. ------------------------------------------------------------

D.yak. GCCCATAATAGTAGTGCGATTGCGTTTGCCTTTGCATTTG-------------------C

D.mel. --------------------------TGCCTTTGCATTTG-------------------C

D.sim. ----------------------------CCTTTGCATTTG-------------------C

D.wil. ATTTG-----------------TAGAACTGCCTCCTCCTCCTCCACTACCTC--------

D.pse. CACTGGCACTAGCACTGTCATCGTCTTCATCCTGCTCCTCCTCCTGGAGATCCTCAGAGT

D.bia. -----------------------------------TTTT---------------------

D.yak. ATTTG-------------------------CCTGCTTTT---------------------

D.mel. ATTTG-------------------------CCTGCCTTT---------------------

D.sim. ATTTG-------------------------CCTGCCTTT---------------------

*

D.wil. ------CTGCTGCATC---------------------------TCCCCCACTG-------

D.pse. TCTCAGCGGTTGCACTCTTGTGATGCGTACCTCCGCCTCCTCCTCCGCCATTCACCCCGC

D.bia. ------TGATTGGATT---------------------------TCCCCTATTC-------

D.yak. ------AGATTGGATT---------------------------TTCC----TC-------

D.mel. ------AGATTGGATT---------------------------TTCC----TC-------

D.sim. ------AGATTGAATT---------------------------TTCC----TC-------

** * * * *

D.wil. ------------------------------------------------------------

D.pse. TGCCATTGGTGGTGCCTCCTGTCGATCCATTGCTGCCACTGCCACCGCCACTGCTGCTGT

D.bia. ----------------------------------------------------------G-

D.yak. ----------------------------------------------------------GT

D.mel. ----------------------------------------------------------G-

D.sim. ----------------------------------------------------------GT

D.wil. -----GCGGCACCACCACCACCACCACCGCCAGTACTGCTTGTGGAGGCACCACCTGTGG

D.pse. TGTTTGTGGCTTCAGAGCTGCCACCGCCGCCTGTTCCACTGATGGAGGCACCGCCT---G

D.bia. TTTTTGT------------------------TGTCCGACTTAAG----------TT---G

D.yak. TTTTTTT------------------------CGTTCGATTTATA----------CT---G

D.mel. TTTTT---------------------------GTTCGATTTATA----------CT---G

D.sim. TTTTT---------------------------GTTCGATTTATA----------CT---G

** * * * *

D.wil. GCGCATTATCATCCTCATCGTTATCACAATTGTTGCCCTTCTCCCGCAATCCTGAATTGG

D.pse. GGGCATTGTCATCCTCGTCGTTATCACAATTGTTGCCCTTCTCCCGCGCAGCCGCCGATC

D.bia. GAA---------------------------------------------------------

D.yak. GGAC--------------------------------------------------------

D.mel. GGAC--------------------------------------------------------

D.sim. GGAC--------------------------------------------------------

*

D.wil. CGGCACTTTGCTCACTACTCTGTTGTTGTTG---------TTGCTGCTGCT------GTT

D.pse. CAGCGCTCTGTCCACTCTGCTGCTGCTGCTGCTGCTGCTGTTGCTGTTGCTCCGTTGTTG

D.bia. ---------------------------------------------------------TTT

D.yak. ----------------------------------------TTGCAGTTGAT------TTT

D.mel. ----------------------------------------TTGCAGTTTAT------TTT

D.sim. ----------------------------------------TTGCAGTTTGT------TTT

*

D.wil. CTCCAGCAGATGATGATGATGCTGCCGCAGCAGCTGCTGCATTAGCTCCAGCTTCATCCT

D.pse. CTTCGG------------------------------------TGGCAGCAGCATCTTCCT

D.bia. ATTTGA------------------------------------T------------TTCTT

D.yak. CTTTGA------------------------------------T------------TTCCT

D.mel. TTTT-A------------------------------------T------------TTCCA

D.sim. CTTTGA------------------------------------T------------TTCCT

* * **

D.wil. CCAATTGCAAGACACTTATTCGCTTACGATTAATCGACTTGGATGGTTTCCCCGGAAACT

D.pse. CCAATTGCAGGACACTTATCCGCTTACGATTGATCGACTTGGAGGGTTTCCCGGGAAACT

D.bia. CGATTTGT---------------------TTGA-------------TTTCTTG---GACT

D.yak. CGAATTGT---------------------TTGA-------------CTTCTCG---AACT

D.mel. CGAATTGT---------------------TTGA-------------TTTCTCG---AACT

D.sim. CGAATTGT---------------------TTGA-------------TTTCTCG---AACT

* * *** ** * *** ***

D.wil. TCGAACGGCCCATAATA-------------------------------------------

D.pse. TGGAGCGGCCCATAATAGAGTTGTTCTTCCGCTTGTTTGTTGCATTTGCCTTCGATTGCG

D.bia. TG----------------------------------------------------------

D.yak. TG----------------------------------------------------------

D.mel. TG----------------------------------------------------------

D.sim. TG----------------------------------------------------------

*

D.wil. -------------------------TATATA---------------------------CG

D.pse. GGGGGCACACGATAGGGGTATCACTTGGATATCGGAGCAGGTTGGCGCTTTCCTGGCTCC

D.bia. -------------------------TAGATA-----------------------------

D.yak. -------------------------TGGATA-----------------------------

D.mel. -------------------------TGGAAA-----------------------------

D.sim. -------------------------TGGATA-----------------------------

* * *

D.wil. CACTTGCTGAATGTCTTTTTAATTTTTGCATTTGCGTACCGTTTGCCGTTTGTCTTCGAG

D.pse. CACACACTG---------------------------------------------------

D.bia. -GCTCACTG---------------------------------------------------

D.yak. -ACTCACTG---------------------------------------------------

D.mel. -ACTCACTG---------------------------------------------------

D.sim. -ACTCACTG---------------------------------------------------

* ***

D.wil. TTTGCTGGTTTTCTTTTGTTTTTTGTGTTTTTTGTTTTTTG-GCTACAACATTGGCGGCA

D.pse. ---------------------------------------TATGAAGAGAGAGAGAC----

D.bia. ---------------------------------------TG-GATAAGAGAGAGGC-ACA

D.yak. ---------------------------------------TG-GATAAGAGAGAGGC-ACA

D.mel. ---------------------------------------TG-GATAAGAGAGAGGC-ACA

D.sim. ---------------------------------------TG-GATAAGAGAGAGGC-ACA

* * * * * *

D.wil. CTTTCCATGCAACAACAA-------CGACGACGGCTGCCACTGCTGCAACTGCAGGCGAA

D.pse. -GGTTCACGTGAGAGACACGACACCCAGCACAG--------------------------A

D.bia. AACTTCAAGTAAGAGAGA------CCGACTA----------------------------A

D.yak. AACTTCGGGTAAGAGAGA------CCGACTAAG--------------------------A

D.mel. AAGTTCGGGTAAGAGAGA------CCGACTAAG--------------------------A

D.sim. AAGTTCGGGTAAGAGAGA------CCGACTAAG--------------------------A

* * * * * * * * *

D.wil. AATAACATTGTTAACATGAGTCCAAGACAACAACAACAACAAAAAAAGACCGGTATTATA

D.pse. AATACCGGTATT--------------------GCAGCAGCAACG-------------ACA

D.bia. AAGACCGGTATTAGC----TAAAGGAAAACTGGC-AAAACAAAA-------------ACA

D.yak. AAGACCGGTATTAGCACG-AAGAAATCAACTAGCAAAAACAGAA-------------ACA

D.mel. AAGACCGGTATTAGCACGAAAAAAATCAACTAGCAAAAACAGAA-------------ACA

D.sim. AAGACCGGTATTAGCACGAAAAAAATCAACTAGCAAAAACAGAA-------------ACA

** * * * ** * * ** * *

D.wil. CCAGCAGCTACAAC----------------------AACAACAATGCCTGTAA-CCGAAC

D.pse. ACAACAAT-GCGGCCCCCGTCCCCCTTCCCCTGTAACCGAACCGAAC---CAAACCGAAC

D.bia. CCAAC----ACAGAGCTCGG------------GGAAAAAAACAATGCTTGTAAGCCGAAT

D.yak. CCAACAAC-ACAGCTCGGGGTA----------AAAAAAAAACAATACTTGTAAGCCAAAT

D.mel. CCAACAACAACACCGCTCGG------------GAAAAAAAACAATACTTGTAAGCCAAAT

D.sim. CCAACAACAACACCGCTCGGAA----------AAAAAAAAACAATACTTGTAAGCCAAAT

** * * *** * ** ** **

D.wil. CGCGC--TTGGAATGCGTCAATATTCCTCTC---CCCACACTTCCCTTCACCCCGTCTCT

D.pse. CGCGTCTTTGGAATGCGTCAATTTTCCCCCC------------TTTCGGTACGCGACG--

D.bia. CGCGCGTTTGGAATGCGTCAATTTTTCCCCCTGTTTTTTATTTTTGCGAATCTCGTTGTT

D.yak. CGCGCGTTTGGAATGCGTCAATTTTCCCCCT---------TTTTTACGAATCTCGTGGTT

D.mel. CGCGCGTTTGGAATGCGTCAATTTTCCCCCT---------TTTTTGCGAATCTCGTTGTT

D.sim. CGCGCGTTTGGAATGCGTCAATTTTCCCCCT---------TTTTTGCGAATCTCGTTGTT

**** *************** ** * * * **

D.wil. CCCACTAT---------ATCTATCTCTCTCTCACACAAAACCCGCCTTGAAGTTTTGTTG

D.pse. ---ACCGA-CCCGACTCGTTTCTGCT-CGTTCCTGTTTCG-----GCTCTAGCTCTCCTT

D.bia. GTTGCTGTGGGCAACTCGTTT-TTTTGCACTCCTGCCTCACGCCCCCCTCACTACCCACT

D.yak. GTTGCTGT-GGCAACTCGTTTATTTTGCACTCCTGCCGCACGCCCCCTTCACTTCCCACT

D.mel. GTTGCTGT-GGCAACTCGTTTATTTTGCACTCCTGCCGCACGCCCCCTTCACAACCCACT

D.sim. GTTGCTGT-GGCAACTCGTTTATTTTGCACTCCTGCCGCACGCCCCCTTCACAGCCCACT

* * * * * ** *

D.wil. CAGTTGTTGTTGTTGTTGTGGTTACCACTTACGATGCAATACGAGGCGACGCGTTTCGTT

D.pse. CGCCTTCTCCTTTTCCTCCTTCTTCTTCTT--------------------CTTCTTCTTT

D.bia. CACTCACTCCTTTTCGCACACACACACTCT--------------------TTTTCTCCTT

D.yak. CACTCACTCCTTTTGGCGCACACAC-------------------------TCTTTTCCTT

D.mel. CACTCACTCCTTTTGGCGCACACAC-------------------------TCTTCTCCTT

D.sim. CACTCACTCCTTTTGGCGCACACAC-------------------------TCTTTTCCTT

* * * ** * ** **

D.wil. T-------CAAATCGTTCCCGTAATTTTTT-----TGCGCGTTCCT------------CG

D.pse. TGCGTTCCCCACTTGCGTCTTGGTTTTTTTATTCGCGCGCGCTCCTCGTTCCGTTCCTCG

D.bia. GG------CTATTTATTTATTTATTTC-TT-TTCGCGCGCGTTTCTCGTTGCG-----CG

D.yak. GG------CTATTTATTTATTTATTTCTTT-TTCGCGCGCGTTTCTCGTTGTG-----CG

D.mel. GG------CTATTTATTTATTTATTTCTTT-TTCGCGCGCGTTTCTCGTTGCG-----CG

D.sim. GG------CTATTTATTTATTTATTTCTTT-TTCGCGCGCGTTTCGCGTTGCG-----CG

* * * ** ** ***** * * **

D.wil. CTCCTTGCTTTGCAAATTACAACTATTAAGCCGAAGATGACGTCGAAGTTGAAGCGCGCC

D.pse. CTCCTTGCTTTGCGAATTTCGA--ATAAAGCCGAAGGTGACGT--------CAAAGCGCG

D.bia. CTCCTTGCTTTGCGAATTACAACTACAAAGCCGAAGGTGACGT--------AGCCGCGCG

D.yak. CTCCTTGCTTTGCGAATTACAACTACAATGCCGAAGGTGACGT--------ATCCGCTCG

D.mel. CTCCTTGCTTTGCGAATTACAACTACAATGCCGAAGGTGACGT--------ATCCGCTCG

D.sim. CTCCTTGCTTTGCGAATTACAACTACAATGCCGAAGGTGACGT--------ATCCGCTCG

************* **** * * * * ******* ****** ** *

D.wil. AG---------AGACTCAACTCTG----AGCCC----------ACGACTTTGTTGACTCT

**D. pse. S2.19 Fwd**

D.pse. ACAACGCGTCAAGCCTCGGCTTTG-------TCTGA-----------GCTCTCTCTCTCT

D.bia. AC---------AGCCTCGGCTTTTGTGCCGCTCTTGCGCCCGAACGCGCTCAAACGCTCT

D.yak. AC---------AGCTTCGGCT----------TTTGA-------ACACGTTCGGTCGCTCT

D.mel. AC---------AGCCTCGGCTTTT----TGCTTTTG-------ACGCGCTCAATCGCTCT

D.sim. AC---------AGCCTCGGCTTTT----TGCTTTTG-------ACGCGCTCAATCG--CT

* ** ** ** * **

**D. wil. S2.19 Fwd**

D.wil. CTTGGACTCTCTCGTACGCT----------------------------------------

D.pse. TTCTGTTTTTT-TTCTCGCT----------------------------------------

D.bia. CTCAGTCGCTC-CTTACACTGGCCAATAAAAGGCT--ATTCTCGAATAA----AAAGGAC

D.yak. CTCAGTCGCTC-CGTACACT---CAAAAAAAAACT-GTTGTTCGAAAAAT---AAAGGAT

D.mel. CTCAGTCGGAG-CGTACGCT---CAAAAAAAAAATTGGTGATAATTTGAT---AAAGGAT

D.sim. CTCAGTCGCTC-CGTACACT---CAAGAAAATAGT---TGATAATTTGATTTAAAAACAA

* * * **

D.wil. -----------------CTCTTTG------------------------------------

D.pse. ---TGGTTACTCTCTCTCCCTCTCTGAGCCTGA---------------------------

D.bia. ATTTGGTTT--------TTCTTCTAGATTATGTTAAATCTTTGGGTCAAAAGGGCTTTAA

D.yak. CTATGATTTCGGCCG--TTTCTTGTGACCATATTTA-------------AGAAATTAAAA

D.mel. CTATGATTTTTTAAGATTTTTTTAAGATCATATTAAAGAT----ATTACAAGAAATATAA

D.sim. AAATGATTTTTAAAGATTTTTTTATAATTTTCTTAAGATC----A----AATTATTATAC

D.wil. ---------------------------------CTCCTGACG------------------

D.pse. ---------------------------------CTCTTGCGCGCTCCTTGG---------

D.bia. ATATTAACTTAAAGAGTAATTAAGACTTCTTTATTATTTAAAGTTTAATAAAGG---TTA

D.yak. AT-------------------------------TTATTAACCGTTTATTACATGAGTTTT

D.mel. AT-------------------------------TTATTAACGGTTT-TCAGGTG------

D.sim. AT-------------------------------TTATTGACGCTTT-T-AAATG------

* *

D.wil. --------------ACGACAACGAC-----------------------------------

D.pse. -----------------------ACTGCACTCTCAAG-----------------------

D.bia. TATTTTCGCTTTGAATTAGTGTTATTTTATATTCTTGTCCAACCGACTGGCACTACTCTA

D.yak. GTTATTAGAATTGTATTGAAACAACTGTATCCT---------------------------

D.mel. ----------ATTTATTAAAATAATTGTATCGT---------------------------

D.sim. ----------TTGTATTAAAATAATTGTACCCT---------------------------

*

D.wil. ------------------------------------------------------------

D.pse. --------GTATATTGCCGAAGGCGGGTAAGGCAGGCAGGTCTT-GGGCTGCACTCTCAC

D.bia. AGGCGTGAGTTTATTG--AAATTT----ATGGCCCATATTTTTTATGAGTGCATTCTCTC

D.yak. --------GGTTTTTT--GTACTCA---ATAGGCAATGTTTTTT-TGAGTGCACGCTGTC

D.mel. --------GTTCCTTT--TAACTTAAGGATAGCC--TGTTTTTT-TGAGTGCACACTCTC

D.sim. --------GTTTCTTT--TAATTTAGAGATAGAC--TATTTTTT-TGAGTGCGTACTCTC

D.wil. -------GACGACG----ACACCAACGAGGGGCGC--------------GCT--------

D.pse. GCG----GACCGCCCT-------ACCTTCG-CGGCTATATACCTTGGGGGCTGGAAGGAG

D.bia. CGGGCACCGCGGCTCTCTTCTCCGCCTTGGCCCGCTCTCT-CCT-----ACT--------

D.yak. GGG----AACGACTCTCTTCGCCACCTTGGCCCGCTCTTTCCCT-----GCT--------

D.mel. GGG----TACGACTCTC-TTGCCACCTTGGCCCGCTCTTTCCCT-----GCT--------

D.sim. GGA----TACGACTCTC-TCGCCACCTTGGCCCGCTCTCTCCCT-----GCT--------

* * * * ** **

D.wil. -------------------AAAAGGTGCAATTGCAGCTGCTTTTGTTGCCACTATTCC--

D.pse. AGCAGAGCAGAG--CGCGCTAAAGGTGCAATTGCAGCTGCCTTTGTT-TCGCTCTGGCGT

D.bia. -CCTGAGCAAGGAGCGCGCTAAAGGTGCAATTGCAGCTGCCTTTGTT-CCGCTCCTTC--

D.yak. -CCTGAGCAAGG--------AAAGGTGCAATTGCAGCTGCCTTTGTT-CCGCTCCTTC--

D.mel. -CCTGAGCAAGG--------AAAGGTGCAATTGCAGCTGCCTTTGTT-CCGCTCCTTC--

D.sim. -CCTGAGCAAGG--------AAAGGTGCAATTGCAGCTGCCTTTGTT-CCGCTCCTTC--

******************** ****** * ** *

D.wil. ------TGCTGCTC-----CGTCTTGCGTGACCACCGGCACAC-----------------

D.pse. TTACGTTGCCGCTG--GCACGTCTTGCGTGACCACCAGCACACACACAC-----------

D.bia. ------TGGCGCTGTCTCACGTCTTGCGTGACCACTAATACACTCACACCAAAGATAGTG

D.yak. ------TGCCGCTGTCGTACTTCTTGCGTGACCACTAATACACTCACAGT---GGCAGTG

D.mel. ------TGCCGCTGTCGCACTTCTTGCGTGACCACTAATACACTCACACCTAAAACAGTG

D.sim. ------TGCCGCTGTCGCACTTCTTGCGTGACCACTAATACACTCACACCTTAAACAGTG

** *** * ************** ****

D.wil. ------------------------------------------------------------

D.pse. ------------------------------------------------------------

**D. bia. S2.19 Fwd**

D.bia. ATGCCAGCATGGACTGCCTGTTTAAACTACAATGGGATTACCCACCTAATAATGATCTAT

D.yak. GTGGCAGAATTGGATTTTTATTTAAATT--AATGGGATAAGACATTTAAGACTATGC---

D.mel. TTGGCAGAATTGGTGTTTTATTTAAATT--AATGGGATTTGATATTTAATATTATACTAT

D.sim. TTGGCAGAATTGGATTTATATTTAAATT--AATGGGATTTGTTATTTAAAATTACACTAT

D.wil. ------------------------------------------------------------

D.pse. ------------------------------------------------------------

D.bia. TAAAAAAT--GCATTTTGAATTTTATTTTAAACATTAAGTAAAATGGCAACAAAA-----

D.yak. --------TGGAACAGTGCAACTCATTGTCAAGGTGAAGAGATAGGTAATAAAAA-----

D.mel. TATAAAATTATAACAATACCTGTCAGTATCAAAGTAATG---------------------

D.sim. TATAAAAGTATAAAAGTACCACTCAGTATCAAAATAATGGGAACGGGAAACGGGAAGTTT

D.wil. ------------------------------------------------------------

D.pse. ------------------------------------------------------------

D.bia. ---------------------------ATGATGCCCACTTAAGTAAAA-GACTCTCAGA-

D.yak. -AAACAATGAAAAAGTTTTT-------CTTAAATGAA--AAATTAAAACAAACGTCAAA-

D.mel. -----------------------ACTGCTAAGATCAATCAAATTCAAATAATCTTCAGAT

D.sim. TAATTAATTAAATAGTTTCTTAAATGGCTAAGATTGATCAAATTCAAACAATTTTCCGAT

D.wil. ------------------------------------------------------------

D.pse. ------------------------------------------------------------

D.bia. --AAGTACCGATTATTTCATAA----TAGAATAATTACAATGTATTAATTATTGAACAAT

D.yak. ATACGAAATTTTTATGCTGTTG---TTAATCCAAATAAGACA----------GATATTTT

**S2.19 Fwd**

D.mel. ATAAAAACAAATAATGTGATAAGGCTTAATACACGAAAAACACATGAAAAATGACATTTT

**D. sim. S2.19 Fwd**

D.sim. ATAAAAACAAATAATGTGATGAAACTTAATCCACGCAAAACACATGAAACAGGATATTTT

D.wil. ------------------------------------------------------------

D.pse. ------------------------------------------------------------

D.bia. TGTCAAAAATGGTATCAGAT---------------AAGTATGC------TTTCTTTTCAG

D.yak. TGGATGAAAATATGTGTAATAGACTTGATGTTCAAAAGTTTACCAAAACGTTTAATTCGA

D.mel. TAT-----------ACAAATCGAGGTTTTGTTCAGAAGTTTCCTTCTG-TTTTCTTTCGT

D.sim. TATAAAGAAATACAACAAATCGAGCTTTTGACCAGAAGTTTCCCAAAATTTTTCTTTCGT

D.wil. ------------------------------------------------------------

D.pse. ------------------------------ACACGCACACAGA-----------GAC---

D.bia. TTCCAACA------TTTCAAACCCTATTAAGTAGGCA-ACACGTATCCTAAAAGGACACG

D.yak. ATTAAATAAAAGAATTTCAAACGCTGTTAAGTATGCA-ACAGA--TCTTTAAAGGAC---

D.mel. ATTCAAAAAG--ACTTTAAAACGCGGTTAAGTATGCA-ATAGAT-TCTTTGAAGGAC---

D.sim. ATTCAATAAATTACTTTAAAACGCTGTTAAGTATGCA-ACAGAT-TCGTTGAA-CAC---

D.wil. ------------------------------------------------------------

D.pse. ------------------------GGGCCA------------------------------

D.bia. GAATTTCAAAACCCAAAACTACCTAGCTATTTCTGGCGGGAAATCGGCCTTGCTGTCGGC

D.yak. --ATTTCACAATTT----------GGCCCAATTTAGCGGTAAAACAGCCTAGCTGCCAGC

D.mel. --TTATCACATTTC----------GGCCAAATTTAGCGGGAAAACGCCATAGCTGCCAGC

D.sim. --TAATCACAATTC----------GGACCAATTTAGCGGGAAAACAGCATTGCTGCCAGC

D.wil. -----------ACCACCTTTTCACGC--TGCTACCACTAAATGT----AGTTTAAAATAA

D.pse. ----------CACCACCTTTC---------CTACCACTAAATAC---CCCTCAA------

D.bia. ACTGGCATACCTCCACCTTTACACGCTTTGCCACCACTAAATGTGTGCAGTTAACGAAAA

D.yak. ACTGGCTTACTACCACCTTTACACGCTTTGCCACCACTAAGTGTGTGCAGTTAACGAAAA

D.mel. ACTGGCTTACCACCACCTTTGCACGCTTTGCCACCACTAAGTGTGTGCAGTTTACGAAAA

D.sim. ACTAGCTTACCACCACCTTTACACGCTCTGCCACCACTAAGTGTGTGCAGTTAACGAAAA

******** * ******** * * *

D.wil. AAAAACAACAA-------CAATATAACTGCTGCA-----GCTCCTTA-------------

D.pse. -------------------AAAACAACTGCCCC--TGCTGCTCCTT--------------

D.bia. AACAAAAAAAAAGTGAAGCAAAACAACTGCTGCGTTGC-GCTCCTTAAACTCATTCC-TT

D.yak. AACAAAAAAAAATTGAAGCAAAACAACTGCCGCGTTGTGGCTCCTTAAACTCATTCCGTT

D.mel. AACAAAAAAAAATTGAAGCAAAACAACTGCTGCGTTGTGGCTCCTTAAACTCATTCCGTT

D.sim. AAC-AAAAAAAATTGAAGCAAAACAACTGCTGCGTTGTGGCTCCTTAAACTCATTCCGTT

** * ****** * *******

D.wil. ------------TAGCACGCG----CTCGCTTCGTCT---TCGTCCACAGATTCCTCTT-

D.pse. -----------GTAGCACGCG-----CGCCACGCTCTCTCACTCTCCCAGACTCCTTTTT

D.bia. TCCTTTGGCACGTAGCACGCG------CTCTCTCCCTCTCTCTCTCGCAGACTCCTTTTT

D.yak. TTCTTTGGCACGTAGCACGCGCTCTCTCTCTCTGTCTCTCTCTCTAGCAGACTCCTTTTT

D.mel. TTCTTTGGCACGTAGCACGCG----CTCTCTCTGTCTCTCTTTCTCGCAGACTCCTTTTT

D.sim. TTCTTTGGCACGTAGCACGCG----CTCTCTCTGTCTCTCTCTCTCGCAGACTCCTTTTT

********* * ** **** **** **

D.wil. ---------------ACTC-------TCTTTGGCTCTCTCTCTCTCTCTCTTTGTCTGTC

D.pse. AAGAAACTCTTGAACTCTC-------TCTTACGCTCTGCTCTGCTC--------------

D.bia. AAGAAACACTCGCTCACTC------------------TTTTTTTTC--------TGGATC

D.yak. AAGAAATGCTCTCACACTCCAATAACTCTCCCGCTCTTTTTCTCTC--------GGCAAC

D.mel. AAGAAATGCTCTCACACTCCAAACGCGCTCCCGCTCTCTCTCTCTCTCTCTCTTGGCAAC

D.sim. AAGAAATACTCTCACACTCCGAACGCGCTCCCGCTCTCTTTCTCTC--------GGCGAC

*** **

D.wil. GGTCTGTCTGTCACTCGTCCCGTAGACGTCGTTCGTTTGTACGTTCG-------------

D.pse. TGCTCTTCTCTCTCGC-TCTCTCTG-----TCCTGTCCGTCCGTTTGGTCTCTGTCACGC

D.bia. TACCTTTCTCCCACCC-TCCCGCGG-----GTTAGT------------------------

D.yak. TACCTTTCTCCCTCCC-TCCCGCGG-----GCTAGT------------------------

D.mel. TACCTTTCTCCCTCCC-TCCCGCGA-----GCTAGT------------------------

D.sim. TACCTTTCTCCCTCCC-TCCCGCGG-----GCTAGT------------------------

*** * * * ** * **

D.wil. -------------TTCAATCGACG-TTTTAAGTGGTTTTTCTACTTCAGACTTGAACT--

D.pse. TGTCCCATAGACGTTTCATCGACG-TTTTAAGTGGTTTTTCTACTTCAGACTTGAACT--

D.bia. -------------TGTCATCGACG-TTTTAAGTGGTTTTTCTACTTCAGACTTGAACTTT

D.yak. -------------TGTCATCGACGTTTTTAAGTGGTTTTTCTACTTCAGTCTTGAACTTT

D.mel. -------------TGTCATCGACGTTTTTAAGTGGTTTTTCTACTTCAGACTTGAACTTT

D.sim. -------------TGTCATCGACGTTTTTAAGTGGTTTTTCTACTTCAGACTTGAACTTT

* ******* ************************ ********

D.wil. -------------------------TTTTCTTCG-CAGGATTTTCTTGTTTCGC------

D.pse. -------------------------TTTTCTCTGCCAGGATTTTCTCGTTTTGCATTGTT

D.bia. TTCTTTTCCTCTCTTTTTTGTTTTCTTTTCTTTG-CAGGATTTTCTCGTTTGGC-----T

D.yak. TTTGTTTCTTGTTGCTGTTG-----TTTTCTTTG-CAGGATTTTCTCGTTCGGC-----T

D.mel. TTTGTTTCTTGTTGCTGTTG-----TTTTCTTTG-CAGGATTTTCTCGTTCGGC-----T

D.sim. TTTGTTTCTTGTTGCTGTTG-----TTTTCTTTG-CAGGATTTTCTCGTTCGGC-----T

****** * *********** *** **

D.wil. ATTGTTGT---AGCTCCTGCGCCTTGTTATTGTTGTTG------TTGTTGCTGCTGCTGC

D.pse. GTTGCTGCTGCCGCTGCTGC-TGCTGCTTTTGTTTCTCTTTTCTATTTTCTTTTTTTTT-

D.bia. TTTGTTGT---TGCTCCTGC-CTTTG-TTTTTTCTCTCGAATTTTTGTTGCTGTTGTTG-

D.yak. TTTGTTGTTGCTGCTGCTGC-CTTTGTTTTTTTCTCTCGGATATT--------TTTTTG-

D.mel. TTTGTTGT---TGCTGCTGC-CTTTGTTTTTTTCCCTGGGATA-T--------TTTTTG-

D.sim. TTTGTTGT---TGCTGCTGC-CTTTGTTTTTTTCTCTCGGATA-T--------TTTTTG-

*** ** *** **** ** * ** * * * *

D.wil. TTTTGCTAGAAATTTAAATTCTTACTTTTGTTGTTGTTGTTGTTGTAAGT----------

D.pse. -TTTGCTGGAAATTGAAATTCTTACAGTTGTTGTTGTTGCTGTT----------------

D.bia. -TTTGCTGGAAATTGAAATTCTTACAGTTGTTGTTGTTGTAGTTGTTGCT----------

D.yak. -TTTGTTGGAAATTGAAATTCTTACAGTTGTTGTTGTTGTTGCTGCTGCT------GCTG

D.mel. -TTTGCTGGAAATTGAAATTCTTACAGTTGTTGTTGTTGTTGCTGCTGCTGCGGATGGCG

D.sim. -TTTGCTGGAAATTGAAATTCTTACAGTTGTTGTTGCTGCTGCTGCTGCT---GATGGCG

**** * ****** ********** ********* ** * *

D.wil. ----------------CCACTACGTTTAAGTGGTGGGTTTTTCCC-----CTTTATGACT

D.pse. --------------GGCCACTA---TTAAGTGGTGGGTTTTTCCT-------TTATGACT

D.bia. -----GCTGCTGTGGGCCACTA---TTAAGTGGTGGGTTTTCCCTGCTGCTGCTGCCGCT

D.yak. CTGATGCCGTTGTGGGCCACTA---TTAAGTGGTGGGTTTTCCCT------------ACT

D.mel. TTGTTGCTGTTGTGGGCCACTA---TTAAGTGGTGGGTTTTCCCT------------ACT

D.sim. TTGTTGCTGTTGTGGGCCACTA---TTAAGTGGTGGGTTTTCCCT------------ACT

****** **************** ** **

D.wil. GC-----CTTTTTATGACGGTTTTTTCTTT-------------------CGCGCGTGTGT

D.pse. GC-----CTTTTTACGACAGTTTTTTTTTTGTTTTTCTGCTGCTGCTGCTG---------

D.bia. GCTGCCTCTTTTTACGACAGTTTTTCTCTT-------------------TG---------

D.yak. GCTGGCTCTTTTTACGACAGTTTTTCTCTT-------------------TG---------

D.mel. GCTGGCTCTTTTTACGACAGTTTTTCTCTT-------------------TG---------

D.sim. GCTGGCTCTTTTTACGACAGTTTTTCTCTT-------------------TG---------

** ******* *** ****** ** *

D.wil. GTGTGTGTGTATATATATTCCGTCTTATTTTTTTGTCTTTCCTTTCTTCTTCTTTATGGT

D.pse. ------------------------TTGCTCTGATTCTCTCTCTCGCTCGCTCTTTATGGC

D.bia. ------------------------TT-------------------CTCGCG-TTTATGGC

D.yak. ------------------------TT-------------------CTCGCT-TTTATGGC

D.mel. ------------------------TT-------------------CTCGCT-TTTATGGC

D.sim. ------------------------TT-------------------CTCGCT-TTTATGGC

** ** *******

D.wil. TTTACGGTGACGGTGACAAGAG-------TAGGAAAATTCTTTTTTTTGCGCGTTTAGTT

D.pse. CTCTAAGGCTGGGTGACAGGAGGAGAGTGAGAGAAAATTCTTCTTTTTGCGCGTTTAGTT

D.bia. CT---------------------------GATGAAAATTCTTCTTTTTGCGCGTTTAGTT

D.yak. CT---------------------------GATGAAAATTCTTCTTTTTGCGCGTTTAGTT

D.mel. CT---------------------------GATGAAAATTCTTCTTTTTGCGCGTTTAGTT

D.sim. CT---------------------------GATGAAAATTCTTCTTTTTGCGCGTTTAGTT

* ********** *****************

D.wil. TTTCTGCGTTTGGAGTTTTTGCTCGAG------GTATTTACAATTTTCCACATTGCTGTA

D.pse. TTTCTGCGTTCTCCGTTTTCGCTCAACTTCGTAGTATTTACAATTTTCCACATTGCTGTG

D.bia. TTTCTGCGTTGGGAGTTTCTGCTCGAC------GAATTCACAGTTTTCCACATTGCTGTA

D.yak. TTTCTGCGTTGGGAGTTTCTGCTCGAC------GAATTCACAGTTTTCCACATTGCTGTA

D.mel. TTTCTGCGTTGGGAGTTTCTGCTCGAC------GAATTCACAGTTTTCCACATTGCTGTA

D.sim. TTTCTGCGTTGGGAGTTTCTGCTCGAC------GAATTCACAGTTTTCCACATTGCTGTA

********** **** **** * * *** *** ****************

D.wil. ---------CTTTCGGAGAA--------CTTTTTTCCTCTTTCTCTCTCT---CTGTCTC

D.pse. TTGCTGTACTTTCCGCGAAAAACTTCGTCTTAACTTCTCTGCTGCCGCTGCTGCTGTTGC

D.bia. ---------CTTTCGCGGCAA-------CTTTTCTCATCTGTAACTGCCACCGCTGTTGC

D.yak. ---------CTTTCGCGGCAA-------CTTTTCTCATCTGTAACTGCCA---CTGTTGC

D.mel. ---------CTTTCGCGGCAA-------CTTTTCTCATCTGTAACTGCCA---CTGTTGC

D.sim. ---------CTTTCGCGGCAA-------CTTTTCTCATCTGTAACTGCCA---CTGTTGC

** ** * *** * *** * **** *

D.wil. T----------TAACTGGCGC----------------------AACCAGGCT--------

D.pse. TGCTGCTGCTGGCGCTGGCGC--------------TGGCTGTTGCTGTTGCTGTGGCAGA

D.bia. TGTTGTTGTTGTTGCTGTTGCTGTTCTTATATTGATTGTTGGTGTTGCTGCT--------

D.yak. TCTTGTTGTTGTTGCTGTTGC---------------------TGCTGATGCT--------

D.mel. TCTTGTTGTTCT---TGTTGC---------------------TGCTGATGCT--------

D.sim. TCTTG------T---TGTTGT---------------------TGCTGATGCT--------

* ** * ***

D.wil. -------------AGGCTACAT------CTTCATAATAATCATGATTATTATC-------

**D. wil. S2.19 Rvs**

D.pse. AGCCAGCGTTCTGGGTCTGCCCCTGCGGCCTCATCATGCTCATTATCACCATCATCCCCA

**D. pse. S2.19 Rvs**

D.bia. -------------GCTTTGTGTTGGCGGCCACATGGTGCTCATTATCATGACC-------

**D. bia. S2.19 Rvs**

D.yak. -------------GGTTTGTGTTGGCGGGCACATGGTGCTCATTACCATTACC-------

D.mel. -------------GGTTTGTGTTGGCGGCCACATGGTGCTCATTACCATTACC-------

**S2.19 Rvs**

D.sim. -------------GGTTTGTGTTGGCGGCCACATGGTGCTCATTACCATTACC-------

**D. sim. S2.19 Rvs**

* *** * **** * * * *

D.wil. --ATCATCATC--------AACATCGAAGGCATCTGAGCAG---CATTGGACTACATCAT

D.pse. TAATCATTATCGGGATCGGATCATCATCATCATCGTGGCATCGGCTCTGGTCTACATCAT

D.bia. --ATCGTTATC--------CCCATCATCATC-TCTCTATAT---CCTCGGTCTACATCAT

D.yak. --ATCATTATC--------CCCATCATCATC-TCAATACAT---CCTCGGTCTACATCAT

D.mel. --ATCATTATC--------CCCATCATCATC-TCAATACAT---CCTCGGTCTACATCAT

D.sim. --ATCATTATC--------CCCATCATCATC-TCAATACAT---CCTCGGTCTACATCAT

*** * *** **** * ** * * ** *********

D.wil. TGCCACACACACACAACAAACACTC--ACACACT----------CACTTACAATGCGATT

D.pse. TGCCATTCCTCCACAAAAACTATACCAACAAACC----------AACTCACA--------

D.bia. TGCCATCCTCCC---------ATTCCACCACTCTCTTCTTCAAGCACGCACA--------

D.yak. TGCCTTCTTCCC---------ATTCCACCCCACTGCTT------CACTCACA--------

D.mel. TGCCATCTTCCC---------ATTCCACCCCACTGCAT------CACTCACA--------

D.sim. TGCCATCTTCCC---------ATTCCACCCCACTGCTT------CACTCACA--------

**** * * * * * ** ***

D.wil. TACACTAGGCGGCAC-----AGGCACACACTTTGTTT-----------TTTTTTTG----

D.pse. CACACTGGCGCACAA-----AAGTCTGT-GTCTCTGTTGTTGGGCAGAGTTTTCTTGAAT

D.bia. CACACTGTTTAAAAAAAAAGAAGTCTGCCGTCTGCGT-----------GTGTCTTGAAAT

D.yak. CACACTGGCTCACAA-----ATGTCTGCAGTCTGCGT-----------GTGTCTTGAAAT

D.mel. CACACTGGCTCACAA-----AAGTCTGCAGTCTGC-------------GTGTCTTGAAAT

D.sim. CACACTGGCTCACAA-----AAGTCTGCAGTCTGC-------------GTGTCTTGAAAT

***** * * * * * * * *

D.wil. -CGCATCAAGTTTTTGTTAGCCTGTGTGCGAGCGCGAGG-----------------GGGG

D.pse. CTGCAAAAA--TGAT-------------TGAGAGGGGGTTTCGGG-----------TTGG

D.bia. CTGCAAAAAATTAAT-------------CGAGGGGGTGGCGGTGGGTGGTGGGCGTTGGG

D.yak. CTGCAAAAAATTAAT-------------CGAGGGGGTGG-----------------TGGG

D.mel. CTGCAAAAAATTAAT-------------CGAGGGGGTGG-----------------TAGG

D.sim. CTGCAAAAAATTAAT-------------CGAGGGGGTGG-----------------TGGG

*** ** * * *** * * * **

D.wil. AGGTCGGAGGAAATTGCGAATTTGTCTTGGCTAGAGAAGGCTCTTTGAATCTGGAAAAAG

D.pse. AGGTTGAG-----------------------------------------GGGGTTGGTAG

D.bia. CGGTTGGGTGGCCAACGAGG-------------------------TGGAGGAGGAGGCGG

D.yak. CGGTTGGGAGGCAATGGAA---------------------------------------GG

D.mel. TGGTCGGG----------A---------------------------------------GG

D.sim. CGGTCGGG----------A---------------------------------------GG

*** * *

D.wil. TTATTGAAAAGGGGGGAAAACAAA-AAATGAAGAAATTTATTAGTTCGCTTCGGTTGCAG

D.pse. GTGTTGGGGAGAGAGAGAAAA----CAAAGTAGAAATTTATTAGTTCGCTTC-GTCGCAG

D.bia. CAACGGGAGAGAGAAAAAAAA----CAAAGTAGAAATTTATTAGTTCGCTTC-GTCGCAG

D.yak. CAATGGGAGAGAGAGAAAAAAAAACAAAAGTAGAAATTTATTAGTTCGCTTC-GTCGCAG

D.mel. CAATGGGAGAGAGAAAAAAAAACA-AAAAGTAGAAATTTATTAGTTCGCTTC-GTCGCAG

D.sim. -GGTGGGAGAGAGAGAAAAAAAAACAAAAGTAGAAATTTATTAGTTCGCTTC-GTCGCAG

* ** * *** ** * ********************* ** ****

D.wil. TTG-GCAA----ACAAGTTGAAAGTTGACTACACATACACATACATGTGAGGTGGTGGTG

D.pse. TTGGGCAAACACACAAGTTGAAAGTTGACT-----------TTTGTTGGA----GAGTGT

D.bia. TTGAGCAA----ACAAGTTGAAAGTTGACT-----------T---TTCGA----GTG-TT

D.yak. TTGGGCAA----ACAAGTTGAAAGTTGACT-----------T---TTTGA----GTGTTT

D.mel. TTGGGCAA----ACAAGTTGAAAGTTGACT-----------T---TTTGA----GTGTTT

D.sim. TTGGGCAA----ACAAGTTGAAAGTTGACT-----------T---TTTGA----GTGTTT

*** **** ****************** * * ** * *

D.wil. TTGGTGAGTGTGTTTG-------GTGAATGA-------------GTGAATGTTGCTTTTG

D.pse. GGTGTGTGCGTGTGTGCGTGTGAGGGGGAGAGCGTGAATGGTGGGTGTGCGATGATTATG

D.bia. TGAGCGTGTGTGCGAATGTCTGTGTGGGTGA-----------CGATGTGCGATGATTATG

D.yak. TAAGCGTGTGT------------GTGGGTGA-----------CGATGTGCAATGATTATG

D.mel. TAAGCGTGTGTGTG---------GTGGGTGA-----------CGATGTGCGATGATTATG

D.sim. TAAGCGTGTGT------------GTGGGTGA-----------CGATGTGCGATGATTATG

* * * ** * * ** ** ** ** **

D.wil. TG----GGTGGGTGAGTGTTTGTCTCTCTGTGTGTGTGT------------CTGTGTGTG

D.pse. TGTGTGCGGATGTGCATGGTTGTGTTC----GTGTGAGTAGATTGCAAGGAGCTCAAATG

D.bia. CA----TGTGTGTGTGAATCA----------GTGAGTGTGCACAGAAAGAAATT----CA

D.yak. CA----TGTTTGTGTGTGGCAGTGACTGAGTGTGAGTGTGCACCGAAAGAAATTTTGGTG

D.mel. CATGTGTGTGTGTGTGTCGCAGTGACTGAGTGTGAGTGTGCACAGAAAGAACTTTCTATG

D.sim. CA----TGTGTGTGGGTCGCAGTGACTGAGTGTGAGTGTGCACAGAAAGAACTTTCTATG

* *** *** * **

D.wil. AG----------TGAATGGGTGAGAGTGGA------------------------------

D.pse. CGGCCTG-ACTGCTGCTGCTCCTGTGTGGCCCTCCCCTTACTACCACCTCCCCCACCTCC

D.bia. GTGCATT-TCTCAGAGAAGATAAATGCGGAGTGCTACAGAAGATTCTAAGGTTTTAACTA

D.yak. ATCTATTGTATTTTAATAC---ATAACGAA----TATA--------------TTTATTTC

D.mel. AT---CT-TATTTACATACTTAAAAACGGAACTCTATA--------------TTGACCTA

D.sim. ATATATT-TATTTACATACTTAAAAACGGAACTCTATA--------------TTGACCTA

*

D.wil. ------------------------------------------------------------

D.pse. CACCAACGAGCATG----AGTCAGTGTGGTAATT-----------------GTTTTCCGT

D.bia. CATAAGAGAGTGTGTAAAAGTATGCTTTAAAATTCTCACTTTCTCATCTTAAATTACTTT

D.yak. TAT---------------A-------TAATGATT------TTCTAAATTCTTATCACTAT

D.mel. TAT---------------ATTTATTTTATTTATTTATTTATTATAATGTATATTCACTAT

D.sim. T-T---------------A-TCAGCCTATTTATTTTGCTATTATAATTTATATTCACTAT

D.wil. -------------------------------------------TGT--------------

D.pse. TGGGATACTCTCGCCGGAAGGGTCATATTAGTTTAATGAGATGTCT-------------T

D.bia. AAAAAAACCTCCGAATTTAGCCCCAAA-TTTTATTTTATCACATATTTTTGTTTTTCAAA

D.yak. TAGTGGGCATGAGTAGAAGACCCCAAAAATATTTACAAATATGTAT--------------

D.mel. TAGTGGACACGAGTAGATACACCTAAG-------------GTGTAT--------------

D.sim. T-------ATAGGTAGATATACCTAAG-------------GTGTAT--------------

* *

D.wil. ----------GGAATGTCA-----------------------------------------

D.pse. TGCCACGAAAAGGAAGGAA----------CAAACTATGACCC---------CACTTCCCG

D.bia. CTGAACAAAAAGTATTTCAAGCATTTAATTTAATTATTAGATTCTCAATTTTAAATTTTC

D.yak. -------AAAAGTATGCTA---------------TAT-AGCTAC-------CATATTCTT

D.mel. -------AAAAGTATGCTA---------------TATAAGCTAC-------CACATTTT-

D.sim. -------AAAAGTATGCCA---------------TATAAGCTAC-------CACAATTTT

* * *

D.wil. -------------------------------------------------AGAGTT-----

D.pse. CAATCTTTT--------------------TTCAAACCAATCTTAA----GGAATTCAAGA

D.bia. AAATTGGTTGATTTTTTATTACCTTAACGTATACTTTAATTTAAAAGCTAAACTTAAGGA

D.yak. GGATAGTTTTATTCGCATTGCCCAATAAATTTGGACTAAATTTAA--CTAAAATT-----

D.mel. ----------------------------GTATATACTTAGTTAA-----AAAATT-----

D.sim. AAATAAATT---------------GTAAAAATATATTTAGTTAA-----AAAATT-----

* **

D.wil. ------------------------------------------------------------

D.pse. ATTCTGGTCTCATTTGGCTCTTCTGCTGA------------------------AAGACCT

D.bia. GTGACAAAATATTTATAATTTTATGATATATGTAGATCTTTTAATAGTAACACAAAACCT

D.yak. -----------------------------------------------------AAGTTCT

D.mel. -----------------------------------------------------AAA---T

D.sim. -----------------------------------------------------ATA---T

D.wil. ----------------------------------------------GCATGTG---ACGG

D.pse. ATCAGAATCAGAGTACTATTACGAT-----------TTCTGATCAT-CAGGTTCGATATT

D.bia. A--AAATTGTAAGTGCTCGTTGAACACATTTTTTGTTTCTGTGTAGGCATGTGCGA---G

D.yak. A--AACTTATAA----TAGTTCAAC-------TTTTTTCTGAGTGTGCATGTGTGA---G

D.mel. A--AA-----AA----TTCTTCCAC-------TTTTTTCTGAGTGTGCGTGTGTGAGCTG

D.sim. A--AA-----AA----TAGTTAAAC------TTTTTTTCTGAGTGTACATGTGTGAGCTG

* **

D.wil. TT-----------------AGACGGTTAGGAATGAGATTGAAGAAACACATTAAATTTCA

D.pse. TTAGATGAAATCCCAGTAGTCAAGGCTCTCTATAAGG-TT-CAGCACTTTCATGGTTCT-

D.bia. TT-----------------TAAGTGCTCGGAATGAGG-TGAAAATACTTTCGAGATTTTG

D.yak. CT-----------------TAAGTGCTCGGAATGAGG-TGAAAATACTTTCGAGATTTTG

D.mel. CT-----------------CAAGTGCTCGGAATGAGG-TGAAAATACTTTCGAGATTTTG

D.sim. CT-----------------CAAGTGCTCGGAATGAGG-TGAAAATACTTTCGAGATTTTG

* * * * ** ** * ** **

D.wil. AACTACTGGAAGAGA----------------------GAGAGTTCCTCTTCC-------C

D.pse. -GGTTTCAGATATCATTCCACACAATCCTGCACAATCATATGTGCTATTCCCACAGGAAC

D.bia. GGGTGGTGGTCGCCG-----------------------------CCTTTCCA-------C

D.yak. GGGTGGTGGCTACCA-----------------------------CCTTTCCC-------T

D.mel. GGGTGGTGGCTAACA-----------------------------CC--TCCC-------C

D.sim. GGGTGGTGGCTACCA-----------------------------CCTTTCCC-------G

* * * * *

D.wil. GTTCCTGTTGCCGCCTTCTTGCACCTTGACTACAGCTAACTACTATTGATTGGCTCCGAC

D.pse. CATTCACTCTACCGTTTA--------------AGGGTATCTAATGTT-------TC----

D.bia. TTCCCCATTTTTTCC---------------------CAACTCCTTTT-------TC-CAC

D.yak. GTCTCTCTCTTGCTCT--------------------CAACTCCTTTT-------TCCCAC

D.mel. CTCCCCCTCACCCTCTTT--------------CAGTGAACTCCTTTT-------TCGCAC

D.sim. CTCTCTCCCTCCCTCTTC--------------CACGCAACTCCTTTT-------TCCCAC

* * ** * ** **

D.wil. AGTTTTT----GCATGAGTCAGTGC-----AATAGTTCTCCTTTTTGTTTTGTTTTGGTT

D.pse. ATCATTCGCACGCCACGTTTATAGC-------TTGTTATTT----------ATTTTTTTA

D.bia. ATTTTCCGAAAGCATGACTCAGTGCAGCGTAATTGTTTTCC----------GTTTCGCCT

D.yak. ATTTTCCGAAAGCATGACTCAGTACGGCGTAATTGTTTTCC----------GTTTCACCT

D.mel. ATTTTCCGAGAGCATGACTCAGTGCGGCGTAATTGTTTTCC----------GTTTCGCCT

D.sim. ATTTTCCGAAAGCATGACTCAGTACGGCGTAATTGTTTTCC----------GTTTCGCCT

* * ** * * * * *** * ***

D.wil. TTGGTTTCCCA-----------------AAAGGTGTTTGTATTTACTTAGCCACCCGACA

D.pse. TGGGTTTCTACTCCTGTTAGCCGAGCCAAAAGGTGTTTC-------TT-GTCAGTGGTTG

D.bia. TTGGTTTCCACTC---------------AAAGGTGTTTG-------GCAGTCAG------

D.yak. CCGGTTTCCACTC---------------AAAGGTGTTTG-------GCAGTCAG------

D.mel. CCGGTTTCCACTC---------------AAAGGTGTTTG-------GCAGTCAG------

D.sim. CCGGTTTCCACTC---------------AAAGGTGTTTG-------GCAGTCAG------

****** ********** * **

D.wil. ACTGTA--ATAGTTAA-----------------CCCGCTCTAACCAGTTAGCTAGCTAGT

D.pse. ATTTAGTTTTAGTTTAGTTTGGTTTGGTTTGGTTTTAGTTTGGT----TTGCTTGTTAGT

D.bia. --------TTAGTTTA-----------------CCTGGTTTACC----TGCCTCGTTAGT

D.yak. --------TTAGTTTA-----------------CCTGGTTTACC----TGCCTCGTTAGT

D.mel. --------TTAGTTTA-----------------CCTGGTTTACC----TGCCTCGTTAGT

D.sim. --------TTAGTTTA-----------------CCTGGTTTACC----TGCCTCGTTAGT

***** * * * * ** * ****

D.wil. TAGTCAATCTTGTCAACTGTACT----------------------------CCAACTATG

D.pse. TAGTCAATCTTGACAACTGTACCCCACAGTGGGCAGGGGAGACGGCGGCGGGCAGC-AAG

D.bia. TAGTCAATCTTGTCAACTGTACTGCATACTGCTCC----------------CCAGC-AAG

D.yak. TAGTCAATCTTGTCAACTGTACTGCATACTGCTCC----------------CCAGC-AAG

D.mel. TAGTCAATCTTGTCAACTGTACTGCATACTGCTCC----------------CCAGC-AAG

D.sim. TAGTCAATCTTGTCAACTGTACTGCATACTGCTCC----------------CCAGC-AAG

************ ********* ** * * *

D.wil. TTTTCTTTATATG---TTTGTAT----------GTATGTATTTG----------------

D.pse. TCAATTT-AGTTACATTTTGTATCTGTATGCATGAACGAACCGAACACAGTGGTCGGGAG

D.bia. TCAATTTAAGTTGCT-TTCGTACATACAA----ATATGTAGTGGGCACAATGGGTTTAAC

D.yak. TCAATTTAAGTTGCA-CTCGTACTCAGCGATACGTACGTAGTTGGTACAATGGGCCAGAA

D.mel. TCAATTTAAGTTGCA-CTCGTACACAGAG----GTACGTAGTTGGCACAATGGGTCGGAA

D.sim. TCAATTTAAGTTGCA-CTCGTACACAGAG----GTACGTAGTTGGCACAATGGGACGGGA

* ** * * * *** * * *

D.wil. ------------------------------------------------------------

D.pse. CATACTGC----------------------------------------------------

D.bia. AAGTTGGCAAACAGGGTT-GATACAAATATAAATTAATCCACTCTGTATCTTATATACAA

D.yak. AATGCGGCAAAGAGGATTTAAAATAGTAATGAAAGCACCCACTTGGGGACTGGTAT----

D.mel. AATATGGCTAACAGGATT------------------ACCCACTTGGGAAGTAATAC----

D.sim. AATATGGCTAACAGGATT------------------ACCCACTTGGGAAGTAATAC----

D.wil. TGTA---------------------------------------CGTATTTAGTTAGTTGG

D.pse. TGTACT----------------------------------CCTCCCCCTCACTGA--GCC

D.bia. AATAGCTTAAAGTTTATATAATTAAAAAAATGTTATTTTACTTCGT-TGCAGTTTTTAAT

D.yak. CGTA-----ATTTTTAC------------------TATTATTTATTCCTTACATATCTCT

D.mel. CATACCTATACTTGTATGTTAT----ATTATTTTATATAATTTCATACTTATTTATCTCT

D.sim. CATACC---ACTTGTATGTTAT----ATTATTTTATATAATTTCATACTTATTTATCTCT

** *

D.wil. CAACACTAGCAAGT----------------------------------------------

D.pse. GGGGCCAAACAGGT----------------------------------------------

D.bia. GGATGTTAGCAAG-----------------------------AAGTAAATCGATGATCTT

D.yak. GGGGTATAGAAAGCTTTTAACAAGCGTC-----------------AAAAAGTTTGTTCTT

D.mel. GGGGATAAGAAAACTTTTCAGAAAGCCTGAAAAAGTTTTCAAAAAAAAAACTTCGTTCTT

D.sim. GGGGATTAGAAAACTTTTC------------------------AAAAAAACTTCGTTCTT

* *

D.wil. --------------------------------CAATGTAGTTTT----------------

D.pse. --------------------------------CAGCATGTGTGAGAGTGTGTG-------

D.bia. AA--AATTATTTCACGATTATTATGGCATTCGTAGCATACTTCTAGG-----AGCTCATA

D.yak. AGGCATTTAT-------------------------CATACTTTTGAACAGTTGAGACA--

D.mel. GGGCATTTATGCTCCTAT----------TTT-TAGCATACTTTTGAACATTTATCTTAAC

D.sim. GGGCATTTATGCTCCGAT----------TTTATAGCATACTTTTGGACATTTATCTTAAC

* *

D.wil. ----------------CCCACCACTCATACATACATA--CATACGTAGT-----------

D.pse. -----------------------------CACCACCCACTGTACGCGAG-----------

D.bia. TACTTCCT-------TTTTTTGAGAGATACATTTTTTAAAATATGAAATATTTACTTAAC

D.yak. ---------------GTCCTCTGGAGTT-CATTATTT--ACTATGAAGT-----------

D.mel. TGCCTCTTGACTTTTATCCTCTAGGGAT-CATTTGTC--TCTATGGAAT-----------

D.sim. TACCTCTTGACTTTTATCCACTAGAGAT-CATTTGTC--TCTATGGAAT-----------

** ** *

D.wil. ----------------------------------------ATATG---------------

D.pse. ---------------------TCGATGTCTCTGGACCTGGCTCTG---------------

D.bia. TAATGACTCAATAAAAACGCATTACTGCGTATGTTCAAATGTTTACCCCGGTTGAAATGT

D.yak. ---------------------TTTTTGTATAAGTTTCAGCATCTT---TGGCCAAATT--

D.mel. ---------------------TTAATGTATAAGTTATTGCATTTG---TGGCCAAATT--

D.sim. ---------------------TTAATGTATAAGTTATTGCATTTG---CGGCCAAATT--

* *

D.wil. ------------------------------------------------------------

D.pse. --------------------------GCTCTGGCTCTGACTCTGACTGTGTTGCTGTCTC

D.bia. GCAAAAAGAATTATACAAAAGCATGTGCTCTGTCACCCACTGTACCTTAACTTGGGTCTC

D.yak. ---AAAAAGTTTAAAACAAATTAAGTGCCTCGACACCCACTGTGCCTTAACTCGGGTCTC

D.mel. ---AAAAAGTTTATAATAAATTAAGTGCTTCGACACCCACTGTGCCTTCACTTTGGTCTC

D.sim. ---AAAAAGTTTATAATAAATTAAGTGCTTCGACACCCACTGTGCCTTCACTTGGGTCCC

D.wil. -------TATCTCAGTGTATTGACGATTGAGATTTTAAGCGTTGCTTGTTTGTCGATTTT

D.pse. CGGTTA-TGTGTC---TGTTTGCGATTTGTGATTTCATTTGTCGATT-TTCCTCTATTTT

D.bia. CTGCCACTGTCTCAGTTGTTTGCGATTTGTGATTTTATTTGTCGATT-TTCCTCGTTTTT

D.yak. CTGCCACTGTCTCAGTTGTTTGCGATTTGTGATTTTATTAGTCGATT-TTCCCCGTTTTT

D.mel. CTGCCACTGTCTCAGTTGTTTGCGATTTGTGATTTTATTAGTCGATT-TTCCTCGTTTTT

D.sim. CTGCCACTGTCTCAGTTGTTTGCGATTTGTGATTTTATTAGTCGATT-TTCCTCGTTTTT

* * ** *** *** ***** * ** * ** ** * ****

D.wil. CTTTCA----------------ATTTATCTTTTAAGC--CACGAACTATGTGGTGGTGGG

D.pse. TTATTTAATTTCCT----TTTAGTTGTTGCTTTTGTCGGGTTTTGTAGTGTCTTTATGGC

D.bia. ATTTTATATCGCCTTTGCTTTGGTTTTTGTTTCTACC--ATTGAGTTGTATCTTTATGGC

D.yak. ATTTTATATCGCCCCTGCTTTGGTTTGTGTTTCTACC--ATTGAGTTGTATCTTTATGGC

D.mel. ATTTTATATCGCCTCTGCTTTGGTTTGTGTTTCTACC--ATTGAGTTGTATCTTTATGGC

D.sim. ATTTTATATCGCCTCTGCTTTGGTTTGTGTTTCTACC--ATTGAGTTGTATCTTTATGGC

* * ** * ** * * * * ***

D.wil. AGAGGCGGAGGCGGGTGATAAAAGGTTGTTGGCATGATATAAGC----------------

D.pse. CC---GAACGGTTG---------GACTATTGGCATGATGTAAGC----------------

D.bia. CC---CAGCGG------------------TGGCATGATGTAAGCATGGGGTCTGAGAGAC

D.yak. CC---CATCGG------------------TGGCATGATGTAAGCATGGGATTCGAGATAC

D.mel. CC---CAGCGG------------------TGGCATGATGTAAGCATGGGATACGAGATAC

D.sim. CCCAGCAGCGG------------------TGGCATGATGTAAGCATGGGATACGAGATAC

** ********* *****

D.wil. ----------------ACCAAGTTAAGCTCAATTGAATAACACATT--------------

D.pse. ----------CCCGGAGCTAA-----GCTTAATTGGATAG--CACTCCTCCGTATATTGG

D.bia. AGCAAATGGGACTGGGACTAAACCGGGCTTAATTGGACAA--CACTCCTCCG--TATTTG

D.yak. AGCAAACGGGACTGGGACTAAACCGGGCTTAATTGGACAGAACACTCATCCG--TATTTG

D.mel. AGCGAACGGGACTGGGACTGAACCGGGCTTAATTGGACAAAACACTCATCCG--TATTTG

D.sim. AGCGAACGGGACTGGGACTAAACCAGGCTTAATTGGACAAATCACTCATCCG--TATTTG

* * *** ***** * * ** *

D.wil. --------TTACATTAATTGCTTAGACGGACGGCATTGTT----------ATGAATAATT

D.pse. CATAGCGATTACACTAATTGCC--GTTTGCCGGAGTTGGCTCGAATGTGTGCGCACGGAT

D.bia. CGCGGCGATTACACTAATTGCT--G-----CGGAGTTGCC----------GTGCATTGAA

D.yak. CACGGCGATTACACTAATTGCT--G-----CGAAGTTGCC----------GTGAATTGAA

D.mel. CACGACGATTACACTAATTGCT--G-----CGAAGTTGCC----------GTGAATTGAA

D.sim. CACGGCGATTACACTAATTGCT--G-----CGAAGTTGCC----------GTGAATTGAA

***** ******* * ** *** * *

D.wil. TAATTA--ATTGATTGTATAT------CAGAATTA-TTGCATTGATATTTATAAAGACTG

D.pse. TGAATTGGATTGAATATGCATATTTGAATGAATGATTTGCATATTCAATCGATAATCATT

D.bia. TAACTAATATCCAATATGCAT------ATGAGCCATTTGCATTTTAAGTCG---------

D.yak. TACCTAGCACCCAATATGCAT------ATGAATAATTTGCATTTTAAATCG---------

D.mel. TACCTAGCACTCAATATGCAT------ATGAATAATTTGCATTTTAAATCG---------

D.sim. TACCTAGCACTCAATATGCAT------ATGAATAATTTGCATTTGAAATCG---------

* * * * * * ** ** * ****** * *

D.wil. AAGTAGATAAGAAAGT-TGTAATGCCGACTCTGATATACCACATCATAAGACAAATTTTC

D.pse. CGATTCGCATAGCTGATTGAAGTGT---------------------AAAAATAAACACTT

D.bia. --------ATGGATGA-TGGAGTGT---------------------TAA---AAATACTT

D.yak. --------ATGGTTGA-TGGAGTGC---------------------TAA---AAATACCC

D.mel. --------ATGGATGA-TGGAGTGT---------------------TAA---AAATACTC

D.sim. --------ATGGATGA-TGAAGTGT---------------------TAA---AAATACTC

* * ** * ** ** ***

D.wil. AA---TGTATCGTGCTTAATCGT-------------------TTTTGTGACGC-------

D.pse. GATTCTAA--AGATCTTAATAGACTGCACACCACCCGCTTCCCCTAGTGATGCAATTGAT

D.bia. AA---TAA--AGATCTTAATCAG-------------------CCCCGTGATAATAGTGAT

D.yak. AA---TAA--AGATCTTAATCAG-------------------CCCCGTGAAGCAAGTGAT

D.mel. AA---TAA--AGATCTTAATCAT-------------------ACAAGTGATACAAGTGAT

D.sim. AA---TAAAGAGATCGTAATCAG-------------------CCCCTCGATGCAAGTGAT

* * * * **** **

D.wil. ------TTTTTTAATCCGATTTTTAGAAAATGTGGGCAATT----TAAAAT--TTTAATA

D.pse. T-ATGGCCTTTGG--------------------ATCAGATTTTCCTGCTATGTGGT----

D.bia. TGGTGGCTATTAAGTGAAGTGCCTTGAAAGGTTATTAGACTATATTGACATAAAATAAAG

D.yak. TTGTGGCTTTTTAATGCGATGCCTTGAAAGGTTATTAAACTATCATGACAT--AAT-AAA

D.mel. TTGTGGCTTTTTAATGTCATGCCTTGAAAGGTTATTAAACTATCATGACAT--AATAAAA

D.sim. TTGTGGCTTTTTAATGTCATGCCTTGAAAGGTTGTTAAACTATCATGACAT--AAT-AAA

** * * * ** *

D.wil. ATAAATTTTAGTGTGTAAG------------ATTTTATAAAAA-----------------

D.pse. -----------------------------------------------------------C

D.bia. GTTAATTCGAACCTATAATTTTAAAAATACCATATATTTATAAATAAAACTATTTCATTA

D.yak. ATATATGTGTGCTTGTAATG-----------GTTTATTTAAAA----------------A

D.mel. AAAAATGTTTGCCTGCAATG-----------GTGTATTTAAAA----------------T

D.sim. ATAAATGTTTGCCTGCAATG-----------GTGTATTAAAAA----------------C

D.wil. --TGTTTTAAATG-----------TGATATTCTTTA----TGGTGTTGT-----------

D.pse. TCTGGCTTCATTA-----------------------------------------------

D.bia. TTTGATCCAAATAATTTTATTCAAAAATGTTCAAGACATTTTATACAACAAGGTTCCATA

D.yak. CTTGATCTAGATA--ATTAC----AATTACTCTGAA----TTTTGCAAC-----------

D.mel. GCTGATCTAGTTACTCTTTC----AATTACTCGTAA----TTATGCGAC-----------

D.sim. GCTGATCTAGTTA--CTTTC----AATTAGTCGTAA----TTATGCGAC-----------

** *

D.wil. ----------------------------------------ATAGTATTCTG-------TC

D.pse. -----------------------------------------CAGTGCGCTT-------CA

D.bia. TTAATAAATACTGTACCTCAATCGAACCTGATACGCCCGCATAATCGACTTATTGATATT

D.yak. -------------TATGGCCCCACAAAGTTTCATAATCTTATTATATACTT-------TT

D.mel. -------------T---------------------------TAATTCACTT-------TT

D.sim. -------------T---------------------------TAATACACTA-------TT

* **

D.wil. ATTTTTACCAAAACACTACAGTCCTATCT-----------ATAGCAGTCATGGTTGACTT

D.pse. A-AA------GTATATGGCAG-TTGAAAGATT-----------------ACAGATTACCC

D.bia. A-AACAAACTGCATATGATTACTCAATCGATTCTCAACAATTTTCTAAAACAAATGAATT

D.yak. A-AATCACCTGAATCTAATTGTGCAATTAGTT--------CTATCTGGTACCAATTAATT

D.mel. A-AATCATCTACATTTTATAG-GCAATCGGTT--------TTACCTGGTACAAATGACTT

D.sim. A-AATCACCTACATATTATAG-GCAATCGGTT--------TTACTTGGTACAAATTAATT

* * * * * *

D.wil. TCCTA---------ATTAAATTTCTGCTTATAAAATGTCCCAGCAACATTCGATATATCC

D.pse. A--------AAGGGA----------ATCGTTAAGGGGGGACACTGG-AATTA-TATATCC

D.bia. ACCTTTGTTGTGCGATATATTTTCTGCCATTGGCAGATGCGAATGGGAGCTA-TTTTCCC

D.yak. GCCAATGTTATGCGATATATAATCTGTCATTGACTGATGCGACTGGGAATCA-TGTCCCC

D.mel. GCCGATGTTATGCGATATATTTTATGTCATTGACTGATGCGACTGGCAATCATTATCCCC

D.sim. GCCAATGTTATGCGATCTATTTTATGTCATTGACTGATGAGACTGACAATCA-TATTCCC

* * * * * * **

D.wil. T--TTTATTTTAAATAAAAGTCAATGAAATGAAATAAACTGGTG------TTTATAATGT

D.pse. ------------------ATTCA--AGACTTCATTTATTTAGT-------ACCATCTGTT

D.bia. TACTCGATGATTGATTACAATCG--GAAACC-------------AACGCAATTATAGATT

D.yak. TACTCTATGATTGATGAGAATCG--GGAACCTACTCAATCACCGGACGCAACTATAGATT

D.mel. CACTCCATGATTGATGAGAATCG--GGAACATACTCAATCAGTGGACGCAACCATTGATA

D.sim. CACTCTATGATTGATGACAATCG--GGAAACTACTCAATCAGTGGACGCAACCATAGATT

* ** * **

D.wil. GCTATTAG---------ATACAC-------------------------TATG--------

D.pse. GCC-TTACAACACCGAAACGCAC-----------TGTTTGTCTAAACGTCTGCTCAATTA

D.bia. TCTATTATTACAG----ACTTCCAAACATTTCTGTGTGTGTCTGA---TATAATCGTTCG

D.yak. GCTATTATAAGAC----ACACCC-----------TATGTCTCTGA---TATAATCGTTCC

D.mel. GCTATTATAAGAC----ACACCC-----------TTCCTCTCTGA---TATAATCGTTCC

D.sim. GCTATTATAAGAC----ACACCC-----------TTCGTCTCTGA---TATAATCGTTCC

* *** * * * *

D.wil. -------------------------------------------GATAATA----TGTACA

D.pse. TACACAATAAATAATTAGATATTTGCAACGACAACAGTAAAACTGCAATATTAGTATCTA

D.bia. TAATTGTGAAA------------TGCGAAAACAA---------TGCAGTATTAGTGACCA

D.yak. TAATCGCTTAA------------TACAAGAACAA---------TACCATATTAGTGGCCA

D.mel. TAATCGCTTAA------------TGCAAAAACAA---------TATAATATTAGTGACCA

D.sim. TAATCGCTTAA------------TGCAAAAACAA---------TACAATATTAGTGACCA

** * *

D.wil. ATTGTAACTAA-AAATAAAA-------TATT-------TTGCATGATATAACAATGCAAA

D.pse. TTTAATGGCGACACACAAAAGCTCAAGCACAAAGCAAATAACATGACATAACAAACTAAA

D.bia. TTTAATACCGA-ACACAAAA-------CACTAGGCAAATATCATGACATAACAAACTAAA

D.yak. TTTAATACCGA-GCACAAAA-------CACTAGGCAAATATCATGACATAACAAACTAAA

D.mel. TTTAATACCGA-GCACAAAA-------CACTAGGCAAATATCATGACATAACAAACTAAA

D.sim. TTTAATACCGA-GCACAAAA-------CACTAGGCAAATATCATGACATAACAAACTAAA

** * * **** * * ***** ******* ***

D.wil. GGTTAATTCTCTCAAGCTCAAAACAATTGTCAACAATTTAAAATACACTC----------

D.pse. GGTTAATTCTCTCAAGCTCAAAACAATTGTCAACAATTTAATACCCATTCACGGGGCAGG

D.bia. GGTTAATTCTCTCAAGCTCAAAACAATTGTCAACAATTTAATACCCATGCAGGGGGCAAA

D.yak. GGTTAATTCTCTCAAGCTCAAAACAATTGTCAACAATTTAATACCCATGCAGGGGGTGGG

D.mel. GGTTAATTCTCTCAAGCTCAAAACAATTGTCAACAATTTAATACCCATGCTGTGGGTAG-

D.sim. GGTTAATTCTCTCAAGCTCAAAACAATTGTCAACAATTTAATACCCATGCAGTGGGTAGA

***************************************** * ** *

D.wil. ------------------------GGGC--------------------------------

D.pse. CAGGCAGGCAGAGGCAGGCACTAATGGTTGACGTTCCGACTAGAAGATGCCCATCGTTCG

D.bia. GG----------GGGAGG------GGTTTTTGG-------------------------CG

D.yak. GG---TGGGGAGGGTGAA------GGGGT-AGG--------------------TAGTTCG

D.mel. ------------AGCGGG------GGGATGAGG--------------------GGGTTCA

D.sim. GGGTATGGTGACAGAGGG------GGGTTGAGG--------------------TAGTTCG

*

D.wil. -------------------------------------CCAACAAATA-------------

D.pse. GATGGGATGAATGTTTTGTTAGATGCTATCACATTTTCAGGAGCGTAAGGAAAGCGTGTC

D.bia. AGACGG-------------------------------CAGACAAGCAAGAAA-------C

D.yak. AGTCGG-------------------------------CAGACAAGCAAGAAA-------C

D.mel. AGTCGG-------------------------------CAGACAAGCAAGAAA-------C

D.sim. AGTCGG-------------------------------CAGACAAGCAAGAAA-------C

* *

D.wil. ----------------------------CACAAA--------------------------

D.pse. AATATTTGAAAACAAAGTTAAGTACGCACACAAAATGTGACTGATCTTTGAGTCCACCTA

D.bia. AAT-------------------------CACAAA--------------------------

D.yak. AAT-------------------------CAGAAA--------------------------

D.mel. AAT-------------------------CAGAAA--------------------------

D.sim. AAT-------------------------CAGAAA--------------------------

** ***

D.wil. ----------------------------------------------------------TA

D.pse. TGGATCCAGGTGTTCAGGATTTCTATGCCTGTTACAGACAATATACCCTATTGTTCTCAA

D.bia. ----------------------------------------------------------AA

D.yak. ----------------------------------------------------------AA

D.mel. ----------------------------------------------------------AA

D.sim. ----------------------------------------------------------AA

*

D.wil. CA-TGTACA-----------------------TACACACAAAATCTAT----------GA

D.pse. CGTTTACCAGGTACAAACACTCGCCAACAAATTGCACACAAATTGGGCC------TGAGA

D.bia. CACTCGCCA-------------GCCAACAAATTGCACACAAATTGTATCTGAGAACGAGA

D.yak. CACTGGCCA-------------GCCAACAAATTGCACACAAATTGTATC------TGAGA

D.mel. CACTGGCCA-------------GCCAACAAATTGCACACAAATTGTATC------CGAGA

D.sim. CACTGGCCA-------------ACCAACAAATTGCACACAAATTGTATC------CGAGA

* * ** * ******** * **

D.wil. CACACTGACTGACTGGCTGGCTGGCTGGTTGACTGACTTGAAAATGCTTAACGCCA----

D.pse. AACAAACACACACACACGCAC--------------GCACACAAAGGCACAGTGGCAGGCA

D.bia. AACCCACACAA--ATGCCGAG--------------G-----AAATGCGCA--GACA-GCA

D.yak. AACCCACACAA--ATGCCGAT--------------G-----AAATGCGCA--GACA-GCA

D.mel. AACACACACAA--ATGCCAAT--------------G-----AAATGCGCA--GACA-GCA

D.sim. CACACACACACATATGCCGAT--------------G-----AAATGCGCA--GACA-GCA

** ** * *** ** * * **

D.wil. -------GCCAACTAAAT--TGTGTTGACATCAATTAACTAAATCGTTTTAATTA---TA

D.pse. ACAGAATGCTTAGCACGCTGTGTGTTGACATCAATTAACTAAATCGCTTTAATTATAGTA

D.bia. GCAGAATGCTAACTACGCTGTGTGTTGACATCAATTAACTAAATCGCTTTAATTA---TG

D.yak. GCAGAATGCTAACTACGTTGTGTGTTGACATCAATTAACTAAATCGCTTTAATTA---TG

D.mel. GCAGAATGCTAACCACGTTGTGTGTTGACATCAATTAACTAAATCGCTTTAATTA---TG

D.sim. GCAGAATGCTAACTACGTTGTGTGTTGACATCAATTAACTAAATCGCTTTAATTA---TG

** * * ************************** ******** *

D.wil. TGACAC-----------ACAGCTGCTGCTGCCTGCCCGCCG--ATCGA----CCAAGCAT

D.pse. TGCCACCGGCA------GCAGTCCCAGCG----------CCAGATCAAA---CCAAGGCA

D.bia. TGCCACCAACCGAACGAACGGCAGCAA------------CAACATCAAAGGCCCAGGGAG

D.yak. TGCCACCAACTGGGCG-GCAGCAACAACATCAACCACATCAACATCAAAGTCACAAGGAG

D.mel. TGCCACCAACCGAGCG-GCAGCAACAACA----------CAACATCAAAGTTCCAAGGAG

D.sim. TGCCACCAACCGAGCG-GCAGCAACAACA----------CAACATCAAAGTTCCAAGGAG

** *** * * * * *** * ** *

D.wil. AT---------------AGCATGAAT----------------------------------

D.pse. AACCCCACCCAGGAGAGAGCGAGAGCGAGAGAGAGAGGCCGCCGCCCAGCCGCAAATACA

D.bia. AGCTC------------AGCATGAAT----------------------------------

D.yak. AATTT------------AGCGTGAAT----------------------------------

D.mel. AATTC------------AGCGTGAAT----------------------------------

D.sim. AATTC------------AGCGTGAAT----------------------------------

* *** **

D.wil. ATTGAAACTAATCAATAAAATCTGATCGAAAGATGACAAAATGCG-----------AATG

D.pse. ATCTGAACAAATCAATAAAAGGCCGCACAAAAATGACAAAACGCGAGCAGGTCCCAAAGA

D.bia. ATTTCAGCGAATCAATAAAATCCCACAGAATGATGACAAAACGCG-------TTGATACG

D.yak. AATTCACCGAATCGATAAAATCCCACAGAATGATGACAAAACGCG-------TTGATATA

D.mel. AATTCACCGAATCAATAAAATCCCACAGAATGATGACAAAACGCG-------TTGATATA

D.sim. AATTCACCGAATCAATAAAATCCCACAGAATGATGACAAAACGCG-------TTGATACA

* * * **** ****** ** ********* *** *

D.wil. ACCATCAAA---AGTAAA---AGACGTGAGAGGGGAGAAAAAGTATTTACTAGAT--CAG

D.pse. AGAGACGCGCAGAACAAACACAAAAACAAAAACAAAAAAAGAGTGAATTTT---------

D.bia. GACGACAAAATGAGCAAATTCAAATG-GAAAACTTTGCAGGGGTTGCTCCTTAATTACGG

D.yak. GACGACAAAATGAGCAAATTCAAATGTGAAGAGAGTACAGGGGTTGCTCCTTAATTACGG

D.mel. GACGGCAAAGTGAGCAAATCCCAATTCC--GAGTGTACAGGGGTTGCTCCTTAATTACGG

D.sim. GACGGCAAAGTGAGCAAATTCCCATTTG--GAGTGTACAGGGGTTGCTCCTTAATTACGG

* * *** * * ** * *

D.wil. TTAATATGTTTTTTGTTAGTTAAATTTTGAAATCAAATTATCA-----AGTAAACTTTAC

D.pse. --------------GATGGGAAAATGCAATT--TGAATGACACCG--------CCTATAC

D.bia. ATCATTTATTTACCGAGGGGAAGATGTTTGTACAGAAGGATAATGGGCGAGAACCTG--C

D.yak. ACCATTTGTTTAACAAGGGGATGATGTTTGTATCGAAGGATAATGGGCGGTAACCTGTAC

D.mel. ACCATTTGTTTACCAAGGGGAAGATGTTAGTATCGAAGGATAATGGGCGGTAACCTGTAC

D.sim. ACCATTTGTTTATCAAGGGGAAGATGTTAGTATCGAAGGATAATGGGCGGTAACCTGTAC

* ** ** * ** *

D.wil. CC----TGTAAGAT----------------TTTTTTCTTGAAAT----------------

D.pse. -----CTGTATGACGCTCAC------------TTTTCTAGA--TGTATCTGTATGGCACC

D.bia. AAGGGCTGCTCGATAATTACGGACTGT---TTGTCATTTGAAGAGCAGTTGATG------

D.yak. ACGGGGTGCTCGATAATTGCTGACCAG-----GTTTTTAGAATTTTAATTGAATTTTTTT

D.mel. ACGGGATGCTCGATAATTGCAGACCAATTTTTTTTTTTAGAATTTTAATTGAAGTTTTTT

D.sim. ACGGGGTGCTGGATAATTGCAGACCAG---TTTTTTTTAGAATTTTAATTGAAGTTTTTT

** ** * * **

D.wil. --AAACATAGGATAAAATACTGATAGGAATCGATCTGCTCGTTGATGCTTATAGCATTTT

D.pse. --GACTAGAGGGGCGTGGGCGT--------------------------------------

D.bia. --AAAGACAGGGGCG-TACCGTTT-CCAATTAATTGATACTTTCGTGTTTCAACCGTTTT

D.yak. T-AGGTACAGGGGCGTTTCCATTT-GAAGATAATTACTTCCCTTGCACATCCTTCATTTC

D.mel. TCAGGTACAGGGGCGTTTCCATTTGGAAAATAATTACTGCTCTTACACATCGTTCATTTA

D.sim. --GGGTACAGGGGCGTTTCCATTTCGAAAATAATTACTGCTCTTACACATCGTTCATTTA

* *** *

D.wil. G-------------------------GGTCCTTCGATTAAGGG--------AGAATCAGT

D.pse. ----------------------------------------GGGCTGACAGTACAGTTA--

D.bia. T------GTCTTTAAATTTTGATAATTATAGATGGGTTTTGGGGGTTTATTAAAATGGTA

D.yak. GATAAAGAGTTTAGACATTAAAAATTAATGGGTTGTTTTTAAGAAG-T-ATACAATTAAA

D.mel. G------------GAAATTTAAGGATGATGGTTTGTTCCAAGGGAGAT-CTGGATTTAAA

D.sim. G------------AAAATTTAAGAATGATGGTTTGTTTCAAGGCAGTT-TTAGAATTAAA

* * *

D.wil. TAACTTTCTAAAGGCGGC-------------------AGTTTAGAACTCCATTATTTAAA

D.pse. -----------------------------------TGAGTCTAGAAGAGT-----ACAAA

D.bia. TATTATTATAGAATGTATTTTAG------------CTAAGGTAAAAACTC-----TGAAA

D.yak. TATTTTTCTCTCATCTGTGTAAA--------ATTGTTTGTTCAAAAGCTC-----GGAAA

D.mel. TTTTTTTATCAGGTCTGCGTCAA---TTTATTTTTTTGGCTTATAAGCTC-----GCAAA

D.sim. TATTTTTATCTCATCTGCGTCAATTTTTTTTTTTTTTTGCTTATAAGCTC-----TCAAA

* ** ***

D.wil. ATAA-------------------------------TGGTTTG------------TTTGTG

D.pse. ACGG----------------------------TTCGGATATGAAATGCACAGTATTTTTA

D.bia. TTAAATTAAATTTCCCCAAGCGGAGAGTAACTGCCTAATTTGACG-----AATAATT-CG

D.yak. ATAA---AAGTTTCC-----------------AACTAGTTTGACGTG---AGTATTTACG

D.mel. ATAA---AAGTTTCC-----------------ATCTAATTTGACGTA---AGTGTTTACG

D.sim. ATAA---ATCTTTCC-----------------ATCTAATTTGACGTA---AGTGTTTACG

* ** **

D.wil. TTTGTTTAACTG--TTATTACGTCAA----------------------------------

D.pse. GATAT-------------TGAGATATTT-------------------TAGATT-------

D.bia. TTTAACCAACAAATTACCTGTGCTAATTGCCTAAATCCCATGCATAAAAGAACTCATTGG

D.yak. TTTATCCGACAAATTTACTGCGCTAATTGCCCGAATTTGATGGATAAAAGACC-------

D.mel. TTTATCCCACAAATTTACTGCGCTAATTGCCCGAATTGGATGG----AAGACC-------

D.sim. TTTATCCGACAAATTTACTGCGCTAATTGCTTAAATTTAATGG----AAGACC-------

* * * *

D.wil. -----GTTAAGT------------------------------------------------

D.pse. -----TTAAATT-----------------------TGCCCATATGAATCCTGTTTTAATC

D.bia. TGTTATTCCAACCCATATTTGCTGACCAACGAAGTTGTCAAAATCAAATTAATGGCTCTA

D.yak. -----CTGAAGC-------TTTTGGCCA-------TGTCCAGATCAAATAAGTGGCACTA

D.mel. -----TTCAAGC-------TTTTGGCCA-------TGTCCAGATCAAATTAGTGGTACTA

D.sim. -----TTCAAGC-------TTTAGACCA-------TGTCTAGATCAAATTAGTGGTACTA

* *

D.wil. ------------------------ATATCTAAG---------------------------

D.pse. AGCAAAAACGTAGAC---------TAATCTAA----------------------------

D.bia. ATAGAAATGGCAAATGTGCCAGA-TCATCTGAGCCACTTAAAAAAACTTTAAAGCATTCA

D.yak. AACGAAAATGCAAATACACTTGATTTATCTAAGTCATC--------ACTTAAAGAGCCGG

D.mel. AACGAAAATGCAAATACACTTGA-TTATCTAAGTTATT--------ACTT----------

D.sim. AACGAAAATGCAAATACACTTGA-TTATCAAAGTTATT--------ACTT----------

*** *

D.wil. ------------------------------------------------------------

D.pse. -----------------------CATGTAGTAGAAACAGTA-------------------

D.bia. AAGTCACCACCATAATCTAGGATTATATCAAGAGAACTCTGGTATCAAGTTACCAAGTAA

D.yak. ATTTCACCACT--------------TATCAAAGAAATGGGA-----AAAAAACCTTTTCA

D.mel. -------------------------------AGAGATTCTA-----AAATCACCTGATAA

D.sim. -------------------------------AGAAATTCTA-----AAAACACCTGATAA

D.wil. ----------------TTTGAAATGTTTATAGAAGAGTCGTAAAAT--------------

D.pse. --------------------------------------------ATTAAGACACAAATTG

D.bia. ATTACAGTCATTTTATTTGCAATGAATT---------TGATTAGCTTCAAACACCTCACA

D.yak. GTTTCTAAAAACT-ATTTTGAATTATTTGAACCCAATTTATTAAAGCGAATTATTAAATA

D.mel. ATACAAAACAATT--TTTTAAA--ATTTGAACACAAATTATGAAAGTAAAATATCAAATA

D.sim. ATACAAACCAATTTATTTTGAATTAGTTGAACAAAAATGATTAAAGTAAAATATAAAAAA

D.wil. ------------------------------------------------------------

D.pse. CA-------------------------------------------TCTCCCTCTAATTA-

D.bia. TA------------GAAGCCAGGGCTGCTCGACCATTTTCCCTGTTTTTCCACCAAATAG

D.yak. CA---------AATGAA--------------------------ATTTTCCCCACAAAAAC

D.mel. CA------CTTA--AAA--------------------------ATTTTCCCCCCAAATAC

D.sim. TAAAATATCCAAGTAAG--------------------------ATTTTCCCCCCAAATAC

D.wil. -----GGATTCAATAACATTTTAACCATTT-------------------------TATTA

D.pse. ---------------------TCTCTGATT-------ATCTGATA---TTGATTGTATGG

D.bia. AGCGCATATTGAGTTACAATTCATCTGATTGCTTGGAATTCGATACGTCTGCCTGTATCC

D.yak. TACA-GAATTAAATTGCTCTATATTTGATTGTTG--AATCTGAGACGTTTGACTGTATTT

D.mel. TTCA-GTATTGAATTGCACTTTATTCGATTGTTT--AATCTGAGACATTTGACTGTATTT

D.sim. TTCA-GTATTGAATTGCACTTTATTCGATTGTTT--AATCTGAGACGCTTGACTGTATTT

** ***

D.wil. C------------------AGGGTATT---------------------------------

D.pse. CTGT------ACTCTTTTAATGA------------ATTAATCCAATTGTAAGTGTCATAC

D.bia. CGAG------ACCCAATTAATGGCGTTCAAGT---GTCGGCTTCACTGTAATTCGAGTGC

D.yak. CGATTTCGACACCCAATTAATGATGTTCAAGTGTCGTCGGCTTCACTGTAATTCGAGTAC

D.mel. CGATATCGACACCCAATTAATGGTGTTCAAGTGTCGTCGGCTTCACTGTAATTCGAGTAC

D.sim. CGATTTCGACACCCAATTAATGGTGTTCAAGTGTCGTCGGCTTCACTGTAATTCGAGTAC

* * *

D.wil. -----TTAAA----AGTCACGCGAGATGCGTG------------GAGTACTATGATTAGG

D.pse. GGGC-CTAAACAAGAGTCACGCGAGA------------------------GCCGTTGGGG

D.bia. ACCATAAAAACCAGAGTCGCGCGGAATGCGTG----------AAGGGGAGGCTG-CGAGG

D.yak. AGCCATTAAACCAGAGCCGCGCGGAATGTGTA--------AAGACGGTAGGCTGTCAGAG

D.mel. AGCCATTAAACCAGAGTCGCGCGGAATTTGTGAAGGTGGGAAGGGGGAAGGCTGTCGAGG

D.sim. AGACATTAAACCAGAGTCGCGCGGAATTTGTG----------GGGGGAAGGCTGTCGGGG

*** ** * **** * * *

D.wil. GGTG----GAGAGAAGGAGT----------------------------------------

D.pse. AGAA----GGGGGGAGCAGAAGAG------------------------------------

D.bia. GGCGTGCCGGGGGCTGCGGCGGAG----------------------GGGGTGGGCCCACG

D.yak. GGGG----GTGGATGGGAGCGGAG---GTGAACGGAGAGCGAGGATGGGGGTGGGTAATA

D.mel. GGGG----GGGGGTTGTGGCAGAGGCTGTGGATGGAGGAGGAGGATGGGGGTGTTTAATA

D.sim. GGAG----GGGGGTTGTGGCAGGNNNNNNNG-----------------------------

* * * * *

D.wil. --------------------------ACGAAACTAGTTA---------ATACGACAAAT-

D.pse. -------GTCGAGAGATGACAGGC----------AACAATCTCC-----AGCGGCGAGCC

D.bia. TAAGGGGGCCAAGAGACAATCGGCCAAC-AAACGAACAATCTCCCGGCCAACGGCGAATA

D.yak. CCAAGGGGCCAAGAGACAATCGGCCAACAAAACGAACAATCTCCCGGCGAACAACGAATA

D.mel. CAAAGAGGCCAAGAGACAATCGGCCAACAAAACGAACAATCTCCCGGCAAACAACGAACA

D.sim. --------------------------CCTAAACGAACAATCTCCCGGCGAACAACGAACA

* * * * *

D.wil. TCGAAAC--------------CCAAACTGGCCAATTCAACAACACTCGGACCACAGCGAG

D.pse. CCGAGAG-------------------CTGGCCAATT-----------TGAACGCATC--G

D.bia. ACGAGGCACAGTAAAGTAAAACTTGCCTGGCCAATTCGA----AATCCGAATGCATC--G

D.yak. ACGAGGC-----AAAGTAAAACTGGCCTGGCCAATTCGA----AATTCGAATGCATC--G

D.mel. ACGAGGC-----AAAGTAAAACTGGCCTGGCCAATTCGA----AATTCGAATGCATC--G

D.sim. ACGAGGC-----AAAGTAAAACTGGCCTGGCCAATTCGA----AATTCGAATGCATC--G

*** ********** ** ** * *

D.wil. CCGGCTTCATC-------------------------------CGAAC---GACGGTCAAC

D.pse. TCATCGACATCGACAGCGA--------------------CATCG------AACCGGC--C

D.bia. TCATCGCCATCGCCATCGCCTCGCCATCATCATCATCACCCTCGAAATTGAATCGACAGC

D.yak. TCATCATCATCATCATCGTCT------------------CCTCGA-----AATCGACAGC

D.mel. TCATCATCATCATCACCGTCT------------------CCTCGA-----AATCGACAGC

D.sim. TCATCATCATCATCATCGTCT------------------CCTCGA-----AATCGACAGC

* * **** ** * * * *

D.wil. CGAATGACGACGACG---------ACAACAACATC---------------------GACG

D.pse. CGAACAGGCAGGCGGTGGCAGTAGGC-AGAGCAGACAGCCGAACAAACACGACGAGGCCG

D.bia. CGAACAAACACGAAG-----ATAAACGAATACTGTGAGCCGTAAATGAGGG--GGAGGGG

D.yak. CGAACAAACACGAAG-----ATAAACGAAAATACTAAACCGAATTGGAGGG--GGAAGAG

D.mel. CGAACGAACACGAAG-----ATAAACGAAAATACTAAACCAAATTTGACGGG-GGAGAAG

D.sim. CGAACGAACACGAAG-----ATAAACGAAAATACTAAACCAAATTTGCGGGGTGGGGAAG

**** * * * * * *

D.wil. AGGCCGCCG-----------------------------------------CACACACACC

D.pse. CCGACTCACACACTCGCAAAAG----CACACACAGAGAGACAGAGACCCACTCACACACC

D.bia. GTGGCGCGAGTCCCGGCAAAGA----GAGAGACGGGGAGAGAGAG-CCAACGCACACACC

D.yak. GCGGGATGA-----AGCAAAAT----------GAGAGAGAGAGAG-CCAACGCACACACC

D.mel. AGGGTATGG-----AGCAAAATGAGAGAGAGAGAGAGAGAGAGAG-CAAACGCACACACC

D.sim. AGGGGATGG-----ATCAAAAT----GAGAGAGAGAGAGAGAGAG-CAAACGCACACACC

* * ********

D.wil. TTTAATTTAATTGCCATCAATTATGGTAAAAAGATTAAGAAATCAAAACAAGCGCAAAAT

D.pse. TTTAATTTAATTGCCATCAATTATGGTAAAAAGATTAAGAAATCAAAACAAGCGCAAAAT

D.bia. TTTAATTTAATTGCCATCAATTATGGTAAAAAGATTAAGAAATCAAAACAAGCGCGAAAC

D.yak. TTTAATTTAATTGCCATCAATTATGGTAAAAAGATTAAGAAATCAAAACAA-CGCAAAAG

D.mel. TTTAATTTAATTGCCATCAATTATGGTAAAAAGATTAAGAAATCAAAGCAAGCGCAAAAG

D.sim. TTTAATTTAATTGCCATCAATTATGGTAAAAAGATTAAGAAATCAAA------GCAAAAG

*********************************************** ** ***

D.wil. GAATAAAAAAAAGAAATACATACATACATGAATAAGAAGAATGG-GCAA-CAAAATGAAA

D.pse. GTACAAAA-----------------ACATGAATAAGAAGAATGGCAAAAGCAAAATGAAA

D.bia. GTACAG-A-----------------ACTTGAATAAGAAGAATGG-GCCCCTAAAATGAAA

D.yak. GTAAAATA-----------------AGTTGAATAAGAAGAATGA-GCCA-CAAAATAAAA

D.mel. GTAAAATA-----------------ACTTGAATAAGAAGAATGG-GCCACCAAAATGAAA

D.sim. GTAAAATA-----------------ACTTGAATAAGAAGAATGG-GCCACCAAAATGAAA

* * * * * *************** ***** ***

D.wil. CAGAATAC----------------------------------------------------

D.pse. CGGAGTACACGCAG----------------------------------------------

D.bia. CGAAGCAAGGGCACTGAACGAAAATAAACACAGGCAGTTTCTATTGATTTTCTATCACAT

D.yak. CCGAATAAAGGCACTTAACGAAATTAGACG------------AGTTTTTTTCTA----AT

D.mel. CTGAATACGAGCACTTAACGAAATTAGATA------------AA--ATATTTTA----AT

D.sim. CCGAATACGAGCACTTAACGAAATTAGATA------------AA--ATACTCTA----AT

* * *

D.wil. -----------------------------------------------------AGACGAA

D.pse. -----------------------------------------------------GCACACA

D.bia. TAACTAATTAAAAACTGGTCAGTATCAGAATTTAACCAATCTAGTCTCCCCAAATATCAA

D.yak. TGTATA----------------CGCAT---GTTGAAGAGT-------------GTATCAA

D.mel. TGAATAAT-------------GTACATCGATTTGAAGAGT-------------ATATCAA

D.sim. TGAATAAT-------------GTACATCGATTTGAAGAGT-------------ATATCAA

* *

D.wil. A-----------------------------------------------------------

D.pse. A----------------------------------------------GCA----------

D.bia. ATAGCGATATAATCTATTAATCATATTAATTGTTCTTGTTTAACTAGGTAAATTTAGGAC

D.yak. A----------------------------------------------TTA----------

D.mel. A----------------------------------------------GTA----------

D.sim. A----------------------------------------------GTA----------

*

D.wil. ------------------------------------------------------------

D.pse. ------------------------------------------------------------

D.bia. TTATTAACTCAAAATTACTAAAGCTTTTCAAATTAAATTATAACTGATTGTCTTATAGAT

D.yak. ---------------TACAA---CTTTTTGAGCTCCAATATGATTGA-------------

D.mel. ---------------TAAGAATGCTTTTTGAGTTGCAATATGATTGA-------------

D.sim. ---------------TAAGAAAGCATTTTGAATTGCAATATGATTGA-------------

D.wil. ------------------------------------------------------------

D.pse. --------------------------AGCAG-----------------------------

D.bia. TTATTGTCCATTTTGTTGAAATAAACAAAGGTGCATCCTAAATTACTAAAAATAAACTAA

D.yak. ------------TTTTCAA------TAGCTG-----------------------------

D.mel. ------------TTGCTGAGATTGGCAGCAG-----------------------------

D.sim. ------------TTGCTGAGATT-GCAGCAG-----------------------------

D.wil. ------------------------------------------------------------

D.pse. ------------------------------------------------------------

D.bia. TAGTGTTTTGTCATATTAAGTGTGTTCTTGTTTAACCAGGTAAATTGATCTAAGCTGGTA

D.yak. ------------------------------------------------------------

D.mel. ------------------------------------------------------------

D.sim. ------------------------------------------------------------

D.wil. ------------------------------------------------------------

D.pse. ------------------------------------------------------------

D.bia. AAGCATCTTCATCTATTAATAACCATTGTCCAACTTTATATACCTATAAATGGATCCGTT

D.yak. --------------AATGCTAAAACTACTACAGCA--ATAGATATAC---TAGATCCAGT

D.mel. --------------ATTATAAACCTTTTCCCAACATTATTGACCTATAAATAGATCTAGT

D.sim. --------------AGTATAAACCTTTTCCCAATATTATTGACCTATAAAAAGATCTAGT

D.wil. --------------------------------------------------------GCCA

D.pse. ------------------------------------------------------------

D.bia. TTCAAAACAAATCTCGTA----TCTAATCTTTGGGATGACAGCATGTCGAAATAATGCCA

D.yak. TTCAAAACAAATCCCTTATTATTCAAATCATTTGGATGACAGCACATCGAAATAATGCCA

D.mel. TTAAAAACAAATCCCATA----TCAAATCAATCGGATGACAGCATGTCGAAATAATGCCA

D.sim. TTGAAAACAAATCTCATA----TCAAATTAATCGGATGACAGCATGTCGAAATAATGCCA

D.wil. --------------GATAAAGAGGGAGATCGGAGATGCAGAGAG----------------

D.pse. ------------------------------------GCGGGGAGAAAGAGAGAC------

D.bia. TTTAATTTCACAATGATA--------CGTCTGGGCTGCTAAGTGATATGAAAACTTGCCC

D.yak. TTTTATTTCACAATGATA--------CGTCTGGGCTGCAAAGTGATATGAAAACTTGCTC

D.mel. CTTTATTTCACAATGATAAGTATGTTCGTCTGAGCTGCGAAGTGATATGAAAACTTGCTC

D.sim. CTTTATTTCACAATGATAAGTATGTACGTCTGGGCTGCGAAGTGATATGAAAACTTGCTC

** * *

D.wil. --------------TGAGTGAGA-------------------------------------

D.pse. ----------------------ACTGACA-------------------------------

D.bia. GTAAATTGCTTAATTGATTAAAATTGACATCTAATTATACGTTTTTTGTTGTGTACGCTG

D.yak. GTAATTTGCTTAATTGATTAAAATTGACATCTAATTATACGTTTTTTGTTGTGTAC-CAG

D.mel. GTAAATTGCTTAATTGATTAAAATTGACATCTAATTATACGTTTTTTGTTGTGTAC-CAG

D.sim. GTAAAGTGCTTAATTGATTAAAATTGACATCTAATTATACGTTTTTTGTTGTGTAC-CAG

*

D.wil. ----------------------------------------------------GCAAGAGA

D.pse. --------GACAGACATAGCCCCCCC------------CTAACCAACACCCCACCAAAGA

D.bia. T--------GTGCCAGTAACCCTCTCTCTGTCGCACTGCATGCCAATA-ACCGTGCAAGA

D.yak. TACACCCCAGTACACATAACCCCATCTCGTTCGCAATACAAGCCAATA-ACCGCCAAAGA

D.mel. TAAACCCCAGTACACATAGCCCCGTCTCTGTCGCACTGCATGCCAATA-ACCGCCAAAGA

D.sim. TACACCCCAGTACACATAGCCCCGTCTCTGTCGCACTGCATGCCAATA-ACCGCCAAAGA

***

D.wil. GGCGAATCCCGGTACCCGAGTTGCAAATTGCGAGTAATTGAGAAGATTAAAGTCAAG---

D.pse. GGCG----CGCTCATACAACTTGCAAATTGCGAGTAATTGAGAAGATTAAAGTCAAGCAG

D.bia. GGCG----CGAGTACAAAACTTGCAAATTGCGAGTAATTGAGAAGATTAAAGTCAAG---

D.yak. GGCG----CGAGCACAAGACTTGCAAATTGCGAGTAATTGAGAAGATTAAAGTCA-----

D.mel. GGGG----CGAGCACAAGACTTGCAAATTGCGAGTAATTGAGAAGATTAAAGTCAAG---

D.sim. GGCG----CGAGCACAAGACTTGCAAATTGCGAGTAATTGAGAAGATTAAAGTCAAG---

** * * * * ***********************************

D.wil. -----CA------AGCACAAAG--CAGTAACAAAGGGAGG----GTGGCAGCCAAGAGAG

D.pse. CATGCCAGAGGAGGGCAGACAGCAGGGCAGCGCAAGAAACCC--GAGGAAG---ACAGAA

D.bia. -----CACACGAGAGCGGAAAG--AGATGGCAAATGAGGTGGGGGCGGGGG---GAGCAG

D.yak. ----------------AGACAG--AGATGGCAGTTGA-------GTGGGAG---GGAGAT

D.mel. -----CACACGAGAGCAGACAG--AGATGGCAAATGA-------GTGGGAG---GGAGAA

D.sim. -----CACACGAGAGCAGACAG--AGATGGCAAATGA-------GTGGGAG---GGAGAA

* ** * * * ** * *

D.wil. GGAGC------------------------GGAGGGTAGTGAGGGTAGACAGA------CA

D.pse. AGAGAC-----------------------AGCTGGAGGC------------------AGA

D.bia. AGGGAGTGG--------------------AGGTGGGGGCCGGACCAGAGAGACAGCAAGA

D.yak. GGAGAATGGTTGTTGGAGACAGGGGAAGGGGGAGGTGCCCCGGTTAAAGAGA----GGGA

D.mel. GGAGACTGGT-------------------GGGAGGGGGCCCGGTCAAAGAGA----GGGA

D.sim. GGAGACTGGT-------------------GGGAGGGGGCCCGGTCAAAGAGA----GGGA

* * * ** *

D.wil. GACAGGCCCAGGTAACTACGATTGAAAGCCGAATTGA-CTGCCGAACGATTAA-------

D.pse. GAGCAGACCAGGTAACGACGATTGAAAACCGAATCGA-CTGCCGAACGATTAAGAGCCAT

D.bia. GAGAGCGCCAGGTAA--------------CGAATCGACCTGGCCAGCGATTAAGAGCCAT

D.yak. GAGAGAGCCAGGTAA--------------CGAATCGA-CTGGCGAACGATTAA-AGCCAT

D.mel. GAGAGAGTCAGGTAA--------------CGAATCGA-CTGGCAAACGATTAA-AGCCAT

D.sim. GAGAGAGCCAGGTAA--------------CGAATCGA-CTGGCGAACGATTAA-AGCCAT

** ******* ***** ** *** * * *******

D.wil. AAAC-TGAGCACGACAATCACA---ACATTTGCACTCACAAATACACACAC-----ACAT

D.pse. AAACACGAGCACGACAATCACAGATACA---------------GCAGCAGCGGCCAGCAC

D.bia. AAACACGAGCACGACAATCACA---ACA---------------ACACTCAC-----CTAC

D.yak. AAACACGAGCACGACAATCACA---ACA-----------------ACTTAC-----ATAC

D.mel. AAACACGAGCACGACAATCACA---ACA-----------------ACTTAC-----ATAC

D.sim. AAACACGAGCACGACAATCACA---ACA-----------------ACTTAC-----ATAC

**** **************** *** * * *

D.wil. ACACATATACTCGCCCACTCATACATTCACCAAATAGACAACT-----------------

D.pse. CCCCTCCCACTCACAGACACACACAGGCACCCAATAGACAACTATCTGCATACACTCGCA

D.bia. AGCACC---------CACTCACACAGCCACCAAATAGACAACTATCTGCATGT---CACT

D.yak. CACACACAGCACCCACACACACACAACTAAAAAATAGACAACTATCTGCATGC---CACA

D.mel. ---------------AACACAAACAGCTACCAAATAGACAACTATCTGCATGC---CACA

D.sim. ---------------AACACAAACAGCTACCAAATAGACAACTATCTGCATGC---CACA

** ** *** * ***********

D.wil. ------------TATAAACATGACGGACAAGTTGGCCAGCGGGTAGAAACCACTATAGAT

D.pse. CTCACA-------ACAAACACGACTGACAAGTTGAGCAGCAAATATACATACATACATAT

D.bia. CACACACTTGTGCCCAAACTCGATTGACAAGTTGGG------------------------

D.yak. CACACACTGCAGCACAAACTCGATTGACAAGTT-GG------------------------

D.mel. CACACACTGCTGCACAAACTCGATTGACAAGTT-GG------------------------

D.sim. CACACACTGCTGCACAAACTCGATTGACAAGTT-GG------------------------

**** ** ********

D.wil. AAATGTATACAAAATCGAAGTCAACCACAACTAGCTTTGCGACAGCTACATACATAAAAT

D.pse. GTACGAAAAAAAAATCGAAGTTTGCCACAAACGAGAGCGCGACACAGCT--ACATAAAAT

D.bia. --------AAAAAATCGAAGTCAACCTCG-------------------C--TCATAAAAT

D.yak. --------AAAAAATCGAAGTCAACCTCG-------------------C--ACATAAAAT

D.mel. --------AAAAAATCGAAGTCAACCTCG-------------------C--ACATAAAAT

D.sim. --------GAAAAATCGAAGTCAACCTCG-------------------C--ACATAAAAT

*********** ** * ********

D.wil. ATGTAAATTTGTATAAAATTATGCAAAAAAACAAAAACAAACAACAAAAAGGCCAAAAAT

D.pse. ATGTAAATTCGTATGAAATTATGCAAAAATGC---------------------------C

D.bia. ATGTAAATTTGTATAAAATTATGCAAAAATGC---------------------------C

D.yak. ATGTAAATTTGTATGAAATTATGCAAAAATGT---------------------------C

D.mel. ATGTAAATTTGTATGAAATTATTCCAAAATGT---------------------------C

D.sim. ATGTAAATTTGTATGAAATTATGCAAAAATGT---------------------------C

********* **** ******* * ****

D.wil. TTAAATACATGTGCAA-GAAACA----AATAAGGCAGAGCGAGGGA-------GAGACAC

D.pse. TTAAATACATGTGCAACAATAAGAGACAAGGAGCGGAAC---------------------

D.bia. TCCACTACATATGCACTGAGACA----AAAGTTCGAAATTTATAAAGCCATTTAAATTAT

D.yak. TTAAATAAATGGGCACTGAAAAA----AATAATCG-------------------------

D.mel. GTTTATAAATGATCACTGAAAAA----AATGATCGTAAATGGGAGG------TAAATTAA

D.sim. TTAAATAAATGGTCACTGAAAAA----AATGATCGTAAGTGGGAGG------TAAATTAT

** ** ** * * **

D.wil. ATACA------------CACACAGA-------CAGACAGTTAGAGAGAGTATGAGAGCCC

D.pse. ---------------GCAAGAGAGA-------AAGCCAAT-----GG-------------

D.bia. ATGTGTCATTGTTGGGCAAAAACAATTTTTTTTATTAAAG-----CATTTCTTTAATATT

D.yak. ------------------ATACCGA-------TACTCAAT-----AAATTGTTTGAAACT

D.mel. ATGAA----TTTTAAGCCCCACAGT-------CACTCCAT-----AACTTTTCCTAC-TC

D.sim. ATGCA----TGTCAAGCACCACCGA-------CACTCAAT-----AAAATTTCCTACTTC

* *

D.wil. CAATAAAGGAGCAGCGTAGGAGA----------------------------GAGAGAGAA

D.pse. -----------------CCAAAA------------------------GCTGAAGAAGA--

D.bia. AATTTAAGCATTATAAACCAAAACAAATACATTCTTGCATAAAAACTTTTGAATAAAAGA

D.yak. CATTTATAATATGTAACATAAAA------------------------TTTAAAAATAATA

D.mel. CACTTATTAAATAT-------TC------------------------TTTGAA-------

D.sim. CATTCATGAAATAT-------TA------------------------TTTGAAAATAACA

*

D.wil. AGTCAA---------------------------------------------------GTT

D.pse. ---------------------------------------------------------AGA

D.bia. TATTGCTTATTTCAAACACTTTATGTATTGGAAATATTCAGAGTTATACTTGTACGTATT

D.yak. CTTCCC----------CATTTA-TG--------------------------------ATT

D.mel. -----------------------TT--------------------------------AAT

D.sim. TTTCGC----------CATTTTTCG--------------------------------AAT

D.wil. AAGCAGCTGAAGAAA--------------------------TTGATGGACAAAAAAAAA-

D.pse. GAAAGACGACACAAA--------------------------ACAAGCAACAAATG-----

D.bia. AAACCATTTTACAAACTTCTTATGTAATAATGCACTTCTATTCAATT-TTAATTGTTTAA

D.yak. AAGAAATTTAACCAA------AAGTAATAACAATAGTTTTTTTTATCAATAATTGTCAA-

D.mel. ----------AC--------------ATAA-------TTATTTGATCAATAATTGGCAA-

D.sim. ----------ATCAA------ATGAAATTA-------TCGTTTTATTAATAATTGACCA-

* * **

D.wil. -----------------------CAGCAGCAACAAATGT---------------------

D.pse. ------------------------------------------------------------

D.bia. AAAAGCCAACAAAATTGTATTTTCATTAAACCAACGAAATAAAAACTCTTGAGTTACTGT

D.yak. -----------------------CATAAATGCCATA------------------------

D.mel. -----------------------CATTAACCGCAAA------------------------

D.sim. -----------------------CATTAATCGCTTA------------------------

D.wil. ---------GAGCCACTCGGAATCAGAGAAAGAGA-----GCATAAGAAAGAAGATGTGC

D.pse. ---------AGCTTGGCGCTCACCTCAAAGAGAGATGGATGGCAAGAAAGA-------G-

D.bia. CAACTTGTTATCTCTTTCAGTGTAACAATGAGAGC-----GCTCAGAGAAA-------GC

D.yak. ---------ATTTTTCTCAGTACAACAATGAGAGA-----GCCCAGAGAAA-------GC

D.mel. ---------ATTTTTCTCAGTACAACAATGAGAGA-----GCCCAGAGAAA-------GC

D.sim. ---------A-TTTTCTCAGTACAACAATGAGAGA-----GCCCAGAGAAA-------GC

* **** * * * *

D.wil. AATAGTGGGGGTGGTAGTGGTGGGGGACAAACAAACTAACGAGTG---------------

D.pse. ------AGAAGC-----------AACGTCAAGAGAGCAGAGCGCAGCACATGGCTCCCCC

D.bia. AGCTGAAGGAGT-----------TGAATCAACAAATGAGAGAGC------------CCCC

D.yak. AACTGAAGAGGT-----------GGAATCAACAAATGAACGAGAG-----------CCCC

D.mel. AGCTGTAGAGGT-----------GGAATCAACAAATGAGCGTGAG-----------CCCC

D.sim. AGCTGTAGAGGT-----------GGAATCAACAAATGAGCGAGAG-----------CCCC

* * ** * * * * *

D.wil. ------TATATGTATGTATATGAGAGCCA--------GAGAGA--ATGCGGGGGTGTTGC

D.pse. AACCCCAACAGACA---ATAGGAGGGGAGCCATGAATGAGAGAGCATGCGGGGGTG----

D.bia. ATCGCCAAAAAACA---AAAAGAGAGCCG--------GAGAGCGCACGCGGGGGAG----

D.yak. ATTCCCCAAATCCA---ATGAGAGCGGCA--------GAGAGCGCACGCGGGGGAG----

D.mel. ATTCCCCAAAAACA---AAAAGAGCGGCA--------GAGAGCACACGCGGGGGAG----

D.sim. ATTCCCCAAAAACA---AAAAGAGCGGCA--------GAGAGCGCACGCGGGGGAG----

* * * * *** * ***** * ******* *

D.wil. TGTTGTGAGCGCGTATGAATGTGTGCGTGCGTG---------------TGTATTTTTGTG

D.pse. ---TGTGCGCTCACTCGTTCGTGTTTCTGCTTG---------------AGAGTG----TG

D.bia. ---TGAGAGTATGTATGTTTG---------------------------CGGGTT----TG

D.yak. ---TGTATGCTTGTATGCTTGTACACACGCACACTCACATACACACACTGTATT----CG

D.mel. ---TGTATGTTTGTATGCTTGTG--CACACAC--------ACATACACCGTATT----TG

D.sim. ---TGTATGTTTGTATGCTTGTGCACACACAC--------ACATACACCGTATT----TG

** * * * * * *

D.wil. AGTGGAAATCAAAACACGCAGC-AAAGGTGAAAC---AAAAATGTGCGCGCCAATACATG

D.pse. TGTGTAAATCAAAACACGCAGCAAAAGGTGAAACATGAAAAATGTGCGCGCCAATAAA-A

D.bia. CCTGTAAATCAAAACACGCAGCCAAAGGTGAAACATGAAAAATGTGCGCGCCAATAAACG

D.yak. CTTGTAAATCAAAACACGCAGCCAAAGGTGAAACATGAAAAATGTGCGCGCCAATAAATG

D.mel. CTTGTAAATCAAAACACGCAGCCAAAGGTGAAACATGAAAAATGTGCGCGCCAATAAATG

D.sim. CTTGTAAATCAAAACACGCAGCCAAAGGTGAAACATGAAAAATGTGCGCGCCAATAAATG

** ***************** *********** ******************* *

D.wil. T-----------------------------------------------------------

D.pse. TGCAAAAGGT------GTGAGAGAGC-------ATTGGTGCTGGTATTGCTGTG---GGC

D.bia. TGCGAAAGGTGTCGTGGTGTGGGTGCTCTCCGGTTTAGTATTGGGATGG-----------

D.yak. TGCGAAAGGTGTCGTGGTGTGGGTGCCACACGGATTGGTGTTGGGATGG--------GGA

D.mel. TGCGAAAGGTGTCGTGGTGTGGGTGCCGCATGGATTGGTGTTGGGATGGATGGATAGAGA

D.sim. TGCGAAAGGTGTCGTGGTGTGGGTGCCGCACGGATTGGTGTTGGGATGGATGGAT--AGA

*

D.wil. ------------------------------------------------------------

D.pse. CATTAAAATAGA-----GTATCTCTCTGGGCCATTAA---TGAGC----AGCATTAAGAG

D.bia. -----------ATATCTATATCTCTG-GGGCCATGAA---TGAGCATTGAAAATTGAGAG

D.yak. TGGATGGACGGATATCTGTATCTCTGTGGGCCATGAA---TGAGCATTGAATATTGAGAG

D.mel. TGGTCGGATGGATATCTGTATCTCTGTGGGCCATGAGCATTGGGCATTGAATATTGAGAG

D.sim. TGGTCGGATGGATATCTGTATCTCTGTGGGCCATGAGCATCGGGCATTGAATATTGAGAG

D.wil. -------------------------------------------------------AAGCA

D.pse. CTGAGCGTGAA---CGCAGTTTGATGATGATGATGAGATGTCCAATTTGCCACG-AAACA

D.bia. CCGACTTGGCAGCTCGCAATTTT-------------GATGTCCAATTTGCCACGAAAACA

D.yak. CCGACTTGGCAGCTCGCAATTTT-------------GATGTCCAATTTGCCACG-AAACA

D.mel. CCGACTTGGCAGCTCGCAATTTT-------------GATGTCCAATTTGCCACG-AAACA

D.sim. CCGACTTGGCAGCTCGCAATTTT-------------GATGTCCAATTTGCCACG-AAACA

** **

D.wil. ------------TG---TACATACAAATATATTTATGTTTGTGTACGTGTTGGAG-----

D.pse. CGAAACAGCAGCCGAACTAAATGCGTGAGAGTGTGTGTGCGTGTGAGTGTTGGGACATCT

D.bia. ------------TGAACTAAATACCCG------------CGCCTGTGTGTGTGGCCATCT

D.yak. ------------TGAACTAAATACTTGTAGGCGAATGTTTGTTTGCGTGTGTGAGCATCT

D.mel. ------------TGAACTAAATACTTGTACGCGAATGTTTGTTTGCGTGTGTGGGCATCT

D.sim. ------------TGAACTAAATACTTGTACGCGAATGTTTGTTTGCGTGTGTGGGCATCT

* ** ** * * * **** *

D.wil. ------GTGGCAGTTGTGGTGA-TGGTGATGG-TGGTGGTAA----TGATGATGTGTTGG

D.pse. -GTTTCGTCGTGGTATTAGTGG----TGATGTGCGGTGGTGGTAGGTAAGG---------

D.bia. GGTTTCGTGGTGGTGGTGGTGGTGGTTGATGGTTTATGGTAAAAGGTAACGAATAGCAAG

D.yak. GGTTTCGTCGTGTGGATGGTGATTG-TGGTGG-TGGTGGCAAAAGGTAACGAAGCACAAG

D.mel. GGTTTCGTGGTGTTTATGCTGTGTGTTGGTGG-TGGTGGTAAAAGGTAACGAAACACAAG

D.sim. GGTTTCGTGGTGTTTATGGTGTGTGTTGATGG-TGGTGGTAAAAGGTAACGAAACACAAG

** * * ** ** ** *** * * *

D.wil. ------------TAACCCAGTTGGCAGCTG---------------CACAC----------

D.pse. ------------TAACCCAGTTGGCAGCTGCACAGCCTCCTCCCTCTCCTTATTCACCAA

D.bia. GTAAAATAATATGAACCCAGTTGGCAGCTG--CAGCCTCTCGAAACGCACTGTAAACAAA

D.yak. GTAAAATAAATTCAACTCAGCTGGCAGCTG--CAGCCTCTCAAAACGCACTGTAAATAAA

D.mel. GTAAAATAAATTCAACTCAGCTGGCAGCTG--CAGCCTCTCAAAACGCACTGTAAATAAA

D.sim. GTAAAATAAATTCAACTCAGCTGACAGCTG--CAGCCTCTCAAAACGCACTGTAAATAAA

*** *** ** ****** * *

D.wil. ------------------------AGCCTCAACAAAAACTAATATTTTGCTAAGCTATCC

D.pse. CA----------------------AGAACTAACGAAAACTAATATTTTG----CCGAGG-

D.bia. GGTGTAAGCGGTATTTACACTGGTAACAACAAAGCAAACTATTATTTTAGCCACCGAAAC

D.yak. GGTGTAATTGCTGCT--GGCTGGTAGCAACAATGCAAACTAAAATTTCGGCCACCGAAGC

D.mel. GGTGTAAATACAGCT--AGCTGGTAGCAACAATGCAAACTAATAATTCGGCCACCGAAGC

D.sim. GGTGTAAATACAGCT--AGCTGGTAGCAACAATGCAAACTAATAATTCGGCCACCGAAGC

* ** ****** * ** * *

D.wil. AGAGAG-----------ACAATGGGAGAGAGAGAGAGAGAGAAAT---------------

D.pse. ------------------------------------------------------------

D.bia. AAACAGAAACCACTAGTACAGTGTTCCCTTGGTAGAGGGAATCTGGTTTTATATCCACAA

D.yak. AAATAGAAATCACACCTACAGTGATCGCTCGCTAAGCGCAGCGTTGT-------------

D.mel. AAATAGAAATCACACCTACAGTGATCGCTCGCTAAGCGAAGCATTGT-------------

D.sim. AAATAGAAATCACACCTACAGTGATCGCTCGCTAAACAAAGCGTTGT-------------

D.wil. -AGAGACC-----------------------TATTAACA---------------ATAAAA

D.pse. -GGTGTTC----------------------------------------------AAAGAG

D.bia. GAGTATCTTACAGATGTATCTTTTGAGATGTTATATAAAAGAGAGTTCCATCACAAAAAG

D.yak. -AATGTTT-------------------ATATTCTAGAAG-------------ATTAATAG

D.mel. -AGTGCTC-------------------ATGTTTTAGA-----------------AAATAG

D.sim. -AGTGCTC-------------------ATGTTGTAGAAA-------------ATAAATAG

* *

D.wil. ATTATTTTTGG----------------------CAATTTTGCGAGGTGTGTGCCTCA---

D.pse. TTGATGCTG---------------------------------------------------

D.bia. ACAAATTTAAAATTCAGGTATCTTATAATCGTTAAATAATAACA-ATATGTTGAATAGAA

D.yak. ATTATCTTGAA----------------------GAATCCCAATAGATACATCATTTAGGA

D.mel. ATTATCTTGGA----------------------GAAGAAGAACAAATAAAC-------AA

D.sim. ATCATCTTGGA----------------------GAAGAACAAGAAATAAACAGCTTAGAA

* *

D.wil. ---AGTTGTTC--------AGGTC--------AGAGAGAGAAATTACTGCTGCTTGTTCT

D.pse. -------CTGC-------------------------------------------------

D.bia. AAAAGATCTTTAATAAAAGAAAATGTAGCAACAAATAAAAAACAACTAACAATTTAATTT

D.yak. AGGTGTTTTTC--------AGATTTTA-----AAACAGGAT------------TTATCTA

D.mel. ATGACTTTTTT--------CGATTTTA-----CAATAGGAA------------TTATGTA

D.sim. AGGAGTTTTTT--------AGATTTTA-----CAATTGAAAAGGTTTAATTATTTATTTT

*

D.wil. TATTG------------------TTATTGTGCATTAAGT-------ACCGCTCATCAGAG

D.pse. ----------------------------------------------TGCGGCTGCTGCAG

D.bia. AAATTGATCGATTTTTTTAGGATTATAATAGTATTAATTATAATTATCAATCTTTCGAAA

D.yak. AA---------------------GAAAGAATTATTATTT-------TAAAGCCTTTGAAA

D.mel. AA---------------------CAAGGATATTTTAATT-------TAAACCCGTTGAAA

D.sim. AA---------------------T-----TATATTAATT-------TAAACCAGTTGAAA

*

D.wil. ----------CGTAAATGCATCGCATCGGCGACAAAG-----AAG--------TTTGTG-

D.pse. ------------------------------------------------------------

D.bia. GCACATTTTCCCTACTATGATCATACTATTCC--AAGAATTTCGATCAACCCTTTTGATA

D.yak. ----ATACACCTTCAAATGATCTAAAAAGTGCTTAAG-----AAA--------TC-----

D.mel. ----ATGTCCCATCAAATGATCTTA-----------------AAA--------TTTGTAT

D.sim. ----ATGTCCCATCAAATGATCTTAAGATTTGTAAAGAAATTAAA--------TTTGTAT

D.wil. -------------CAACGGCATTTGTAATTTGCATACTCTTCCTTAAAATGCAAGGCAGG

D.pse. -------------------------------------------------------CGGCG

D.bia. CTTTGAGTAATTTCCACCATATTTG-AATCTCTT-AAGATTCTG-----------CGACA

D.yak. -----------CCCGTCTAAGTTGA-AGCGT----AAGATTCTGGGGGGTGCGATCGACG

D.mel. TTGTGAGTAGACCCATCCGAATTCA-AATGTGCA-ATGATTCTGTGAGGCGTGATCGACG

D.sim. TTGTAAGTGAACCCGTCCGAATTCA-AATGTGCA-ACGATTCTGGGAGGTGTGATCGACG

D.wil. GTATTTGCGCTCAGCTGTAAGTGGAAAATGTGCATACAAATTA-TGTTGTT---AGGATA

**D. pse. S2.20 Fwd**

D.pse. A---------TCAGTGGAGCGTGAAAA--GTGCACCGGAATCAGCGTTAGCATACGGCCC

D.bia. AGATGCGAGTGCAGTGGGGCGTGAAAA---TGCATTCGGACGAGCATCACT---TCGTCC

D.yak. AGATGCGGATGCAGTAGAGCGTGAAAA---TGCATTTGGATGAGCATCGGT---TGGTCC

D.mel. AGATGCGGACGCAGTGGAGCGTGAAAA---TGCATTCGGATGAGCATCGGT---TGGCCC

D.sim. AGATGCGGACGCAGTGGAGCGTGAAAA---TGCATTCGGATGAGCATCGGT---TGGCCC

*** * *** *** **** * * * *

D.wil. GG---------GCA-TTGCAAAGTCGTTTATGGTTTGTTTATCATAGAATTAGCCTACGG

D.pse. A----------GTGGCCTGCTGTTCCTTC--------GAACTC---CCCTGATTTATTGA

**D. bia. S2.20 Fwd**

D.bia. CATTCCCGTTTGCATTCGGATTTTCATTCCCATTCAAGATCTC---GCATTAGCTTACGG

**D. yak. S2.20 Fwd**

D.yak. -----------GCA-TCACATTTTCATTCCCGTTCAAGATCTC---GCATTAGCTTACGG

**S2.20 Fwd**

D.mel. -----------GCA-TCGCATATTCATTCCCATTCAAGATCTC---GCATTAGCTTACGG

**D. sim. S2.20 Fwd**

D.sim. -----------GCA-TCGCATTTTCATTCCCATTCAAGATCTC---GCATTAGCTTACGG

* ** ** ** * * *

D.wil. CCCA-TTGGCTGATGATGTGGATGTTGTGTGTGTGTGTGTTTCAATTTGTTTTTGCATTT

D.pse. TTC------------------------------------------------------TTT

D.bia. CTCAGTTGGTCGA------------------------------------------CTTTT

D.yak. CTCAGTTGGTCGA------------------------------------------CTTTT

D.mel. CTCAGTTGGTCGA------------------------------------------CTTTT

D.sim. CTCAGTTGGTCGA------------------------------------------CTTTT

* ***

D.wil. GAAAGGCACA----AATTTTTAATATGCGCAATTACTC--------ATTTTACGCCTC-A

D.pse. GAAAGGCAAGGCACATTTTTTAATATGCGCAATTACTC--------ATTTTACGCCTCAA

D.bia. GAAAGGCGAA-------TTTTAATATGCGCAATTACTCATTTTGCGATTTTGCGCCTC-A

D.yak. GAAAGGCGAA-------TTTTAATATGCGCAATTACTCATTTGGTGATTTTGCGCCTC-A

D.mel. GAAAGGCGAA-------TTTTAATATGCGCAATTACTCATTTGGCGATTTTGCGCCTC-A

D.sim. GAAAGGCGAA-------TTTTAATATGCGCAATTACTCATTTGGCGATTTTGCGCCTC-A

******* ********************* ***** ****** *

D.wil. ATGTTATTTGGCGCGTAGTTTCAAGCACACACACACACACACACAGGCAGGCGGCACACA

D.pse. ATGTTATTTGGCGCGTAGTTTCTCACGAACTTAAGCTCACACACA--------------C

D.bia. ATGTTATTTGGCGCGTAGTTTCCCACACAC--CAACACATGCACAGCGT--------ACA

D.yak. ATGTTGTTTGGCGCGTAGTTTCCCACACAC--AAACACACACGCAGGCA--------TCG

D.mel. ATGTTGTTTGGCGCGTAGTTTCCCACACAC--AAACACACA------------------G

D.sim. ATGTTGTTTGGCGCGTAGTTTCCCACACAC--AAACACACA------------------G

***** **************** * ** * **

D.wil. ACATC-AACAACAACAACTACTCGAAGATTTGGAATTTGCCTAGTAGA------------

D.pse. ACAGT-AACACTCTC-----TCACAAGATTTGGAATTTGCCTTTTCGATAGCA----ACA

D.bia. CTAGCGGAGACTAGG-----C-----GATTTGGAATTTGCCTTTTTGATGGCGAAT-AAA

D.yak. ACATC-GACACTAGC-----C-----GATTTGGAATTTGCCTTTTTGATGGCAAAT-AAA

D.mel. GCATC-GACACTAGC-----C-----GATTTGGAATTTGCCTTTTTGATGGCAAAT-AAA

D.sim. GCATC-GACACTAGC-----C-----GATTTGGAATTTGCCTTTTTGATGGCAAATAAAA

* * * **************** * **

D.wil. -------------------------------------------------------TACAC

D.pse. AAAATGGCGTCAACAC---------CAGCCATACATATTTATATCTACAAA----AATAC

D.bia. AAAATGGCGTCAACAAATACCCAAGCAGCC-----------TATGTCTGAATCTGTACAT

D.yak. AAAATGGCGTCAACAAATACTCAAACAGCC-----------TATGTACAAA----TACAT

D.mel. AAAATGGCGTCAACAAATACTCAAACAGCC-----------AATGTACAAG----TACAT

D.sim. AAAATGGCGTCAACAAATACTCAAACAGCC-----------TATGTACAAA----TACAT

* *

D.wil. AAATATATTTTGTAATTATTCATCAAGATCATCATCATCG-TCGTCGTCGTCG--TGA--

D.pse. ACATTTATTTAGTAATTATTCAACAACAACCACAACAACAACAAGCAGCAGCCATCGATC

D.bia. ATATTTACTCTGTAATTATTCAGCGGCATCTA---------------GCAGCCATCGA--

D.yak. ATATTTATTTTGTAATTATTCAACAGCTTCTA-GGCAGCGAACAGCGGCGGCAATCGA--

D.mel. ATATTTATTTTGTAATTATTCAACAGCTTCTA-GGCAGCGAACGGCAGCGGCAATCGA--

D.sim. ATATTTATTTTGTAATTATTCAACAGCTTCTA-GGCAGCGAACGGCAGCGGCAATCGA--

* ** ** * *********** * * * * **

D.wil. --GTGAGGCG------CGGCTGCTGCT----------------TTATTGTATAATTTATT

D.pse. GTTTTAGCGG--------TCTGCCGCTGTGATCGTTTCTCATTTTATTGTATAATTTATT

D.bia. --TTTAG---------CTGCTGCCGCCGAGATCGTTTTTCATATTATTGTATAATTTATT

D.yak. --TTTAGCTGCTGCCTCTGCTGCTGCTGAGATCGTTTTTCATTTTATTGTATAATTTATT

D.mel. --TTTAGCTC------CTGCTGCTGCTGAGATCGTTTTTCATTTTATTGTATAATTTATT

D.sim. --TTTAGCTG------CTGCTGCTGCTGAGATCGTTTTTCATTTTATTGTATAATTTATT

* ** **** ** *****************

D.wil. AAAAACTGTTCAACAATCAGCTCAAT---------------------TTTTAGTTATACA

D.pse. AAAAGC--TTCAACGACAGCAGCAGC-TACAGCGGCAACAGATAAATTTTTAGTTATACA

D.bia. AAAAGT--TTCAACAGCGCCAGCAAC-CAAATAAACAACAA------ATTTAGTTATACA

D.yak. AAAAGT--TTCAACAGCGCCAGCAAC-AAAACAAACAACAA------TTTTAGTTATACA

D.mel. AAAAGT--TTCAACAGCGCCAGCAAC-AAAACAAACAGCAA------TTTTAGTTATACA

D.sim. AAAAGT--TTCAACAGCGCCAGCAACAAAAAAAAACAACTA------TTTTAGTTATACA

**** ****** ** ************

D.wil. AACGGGGCAACACATATAGACAAAAACACACACACAGCCATAGAGAGAGAAAGACATAGC

D.pse. AACACTTGGCC-CTGGTGTGC---------------GTAAGCGAGAGGGAGAGGG-----

D.bia. AACACTGTGGC-CATGTGAGC---------------GCCAGAGAGAGAGAGAGGGAG---

D.yak. AACACTTTGGC-CATGTGAGC---------------CCAACAGAGAGAGCGAGCGAGAGC

D.mel. AACACTTTGGC-CATGTGAGC---------------CCAATAGAGAGAGAGAGAGAGAGA

D.sim. AACACTTTGGC-CATGTGAGC---------------CCAACAGAGAG-------------

*** * * * * * *****

D.wil. ACAGACAGGGATGAGAGAGAATAAGAGAGCAAATGAGCGCACAATTTGA------TTTAA

D.pse. ---------------ACAAGTATCACAGGAGAATCTAAG----ATTTCAAGTACTTGTTA

D.bia. ---------------AGAGAGAGCGCGAGCGAGCGTTCG---AATTTGA------TTTAG

D.yak. ------------GGGAAAAACA--GAGAGCGAGAGAGTG----ATTTGA------TTTAG

D.mel. ------------GAAAGAGACGGTGAGAGCGAGAGAGTG----ATTTGA------TTTAG

D.sim. ---------------AGAGACAGCGAGAGCGAGAGAGCG----ATTTGA------TTTAG

* * * * * **** * * *

D.wil. AATCAGGCGATGACAACAAAAACAA--AAACAATTGC-CATTAAACAGTATTTATTGATG

D.pse. AACGATAC---AGCAACGCAA---------------------------------------

D.bia. AATGAGGC---GACAACAAAAGCA---------------ATTAAGTATTATTTATCGATG

D.yak. AATGAGGC---GACAACAAAAGCAATTAACTGGTCACACATTAAGTTTTATTTATCGGCG

D.mel. AATGAGGC---GACAACAAAAGCAATTAACTGGTCACACATTAAGTTTTATTTATCGGCG

D.sim. AATGAGGC---GACAACAAAAGCAATTAACTGGTCACACATTAAGTTTTATTTATCGGCG

** * * **** **

D.wil. CTG-------------------TGTGCCCTTGTGTGTGTGTAAAATATT-TTATTCAAGG

D.pse. CTGCATTCTTAGATGTCGC--------------------ATCAGAGATCACTAT------

D.bia. CTGCTTTATTATGGCCCGCTGGCGAGCTTGTGTGGGTGTGCCAGCGACCGTCGTTTACCG

D.yak. CTGCTTTATTATGGCTCGCCGCTGTGTTTGTGTGGGTGTGTTCGCGACTGTCATTTACCG

D.mel. ATGCTTTATTATAGCTCGCCGCTGTG--TGTGTGGGTGTGTTAGCGACTGTCATTTACCG

D.sim. CTGCTTTATTATGGCTCGCCGCTGTGTTTGTGTGGGTGTGTTAGCGACTGTCATTTACCG

** * *

D.wil. ACC---------------------------------------------------------

D.pse. ---GTCGAACAGATG---------------------AAATGGGAC---------------

D.bia. TTCGTTTAACGGGTGGTAAATAATGGAAAACGTCGTAGATGGAAATCTCGGCGGGCCATA

D.yak. TTCGTTTTACGGGTGGTAAATAATGGAAAACGTCGTAGATGGAAA---------------

D.mel. TTCGTTTTACGGGTGGTAAATAATGGAAAACGTCGTAGATGGAAA---------------

D.sim. TTCGTTTTACGGGTGGTAAATAATGGAAAACGTCGTAGATGGAAA---------------

D.wil. --CCGACAAAATGATACTCTTTATCAAA--------------------------------

D.pse. ----CATAATGTAGCC--------------------------------------------

D.bia. AGTCTACAGTGCAGCCTGGC-TGGCAAAGATCCAACCTTTTTATCGGACCTCCGATGTTT

D.yak. --CTTACAGTACAATCCCTC-CAACTAA--------------------------------

D.mel. --CTTGCAGTAAAATCCCCC-TGGCTAA--------------------------------

D.sim. --CTTACAGTATAATCCCCCTTAGCTAA--------------------------------

*

D.wil. --------------TA-----GTAAA---------------GAA----------------

D.pse. -----------------------------------------AAA----------------

D.bia. AGGTACCAGGATTTCA-----AAAAGCTGAATTTCAGATACGAA----------------

D.yak. --------------CAGTTATGAAAACCGAAGCCCCAGGGGAAAAAATTTGATGGGGGCT

D.mel. --------------CA-----GAAAAC--------------GAA----------------

D.sim. --------------CA-----GAAAACCAAACAGACAGGGGGAA----------------

**

D.wil. ------------TTACCCAATCATTTCTATTACCATTTG---------ACTACATGTTTA

D.pse. -----------------TAATTATATATC----------------------AACCATTCA

D.bia. -----------TAAACTCAATACTGAATTCCTTTAGTCATTTGCAGCTTCGAATCAATTG

D.yak. GACAATAATGCTGAACTTATTTTTGTGTCTCAATAGTAT-----------AAACTATTAA

D.mel. -----------------TAATATTGTTTCTTATAAGAAT-----------GAAATATTTG

D.sim. -----------------TAATATTGTATCTTATAAGAAA-----------GAAATATTTG

* * * * * *

D.wil. TTGAAATTTAAGAT---------------------ATATGTGGGAAAAACTGAACAAGTA

D.pse. AATGA--TTATTCT----------------------------------------------

D.bia. ATTGA--CTGGCAT---------------ACTTTTAGGCGCCTAACAATAAGTTCTTAAG

D.yak. ATGGA--TTACCATGTAATTAATCTAAAAATTAACAGAGGTCTTAAAATATAATTATA--

D.mel. ATGGA--TTAACTT--------------------CAGGAGTC-----ATATGATTATA--

D.sim. ATGGA--TTAACTT--------------------CAGGAGTC-----ATATAATCATATA

* * *

D.wil. GCCGCCCATAATCGAAG-----------------------------CATATTAAAA----

D.pse. -----------------TGCT---------------------------------------

D.bia. TATCTCAAGAGAAGGTATCCCAGAGATACTCTCAAAGATAATGTCGGATTTTGAAGG---

D.yak. -----TGAGTATG---GTTCT-------------------------TATTTTAAG-----

D.mel. -----TGATAATAGATGTACT-------------------------TATTTTAAG-----

D.sim. TAATCTGATAATAGATGTACT-------------------------CATTTTAAAATACA

D.wil. ---------------------ATATTATTAAGTCTTTTTGCAATGATAGTATCAGGTGCA

D.pse. -----GAAGCAATCGTTTTTGATATCTCGTTTCGCC------------------------

D.bia. -----CAAATGTCAGGTAATGATATCTTATATTATTTT-----TGTGCACTTTAGGTCTA

D.yak. -----CTAGGA----------ATACTT--ATTAGTTTGGAAACTGTTAGTATTGGACGAA

D.mel. -----CAAAAA----------ATATTTCGCCTCCTCTTTAAACTGTTAGTATTAGATTAA

D.sim. TTTCTTAAAAATAGTTTATATATATCTCGCCTTCTCTTAAATTTGTTAGTATTAGATCAA

***

D.wil. CTCTTG--------------------------CCTTTTACCCAATCATTTTCTCTAAAAA

D.pse. --------------------------------AGTACT-----------------TTAAT

D.bia. AGCTAAATATTTTCAAAGGGCTCTGAAGAAACAATGTAAAATTGTAAACTTAAACTTAAG

D.yak. AATTAA--------------------------AATATTAACTAAAGATTTCATAACTTAG

D.mel. AATAAA--------------------------AATATTAACTAATGAATTCGTTGTTAAG

D.sim. AATTAA--------------------------AATATTAACTAAGGAATTCATTGTTAAG

* *

D.wil. AAGACTACAAATTAAATCAT-------------GATCGA-ATTGACTTGATG----TGCA

D.pse. TGAAGCGACCACAAAAACAATTAGCTCGCCAGAG--CAGCACAGCAGCACTGGTCACATT

D.bia. TTAGATACTTATTAA-------------------------AATTCCTTAATATTAATAAA

D.yak. AAAAATGTTTATTAAATCAACATGTTTATCAGAAACCAATACTTGTTTACTAGTACTACA

D.mel. AAAGTTG-TTATTAAATCAACAGGTTTTCCAGAAACAAG-ACTTGCTTACTAGTACTACA

D.sim. AAAGTTG-TTATTAAATCAA---------CAGAAACCAG-ACTTGCTTACTAGTACTACA

* ** * *

D.wil. ------------------TTTAATTTATAACGTTGTTACACCTTT-TGC---------CA

D.pse. AACTGTTATTTATGGATGCTGTCTTGCGGGTGGGCT--TGGGCGTGTGCGAGAGTGAGTG

D.bia. TAATGACAAATAAAAATGAAAAACTAAGAACAGGCC--AACCAAT-TGT---------CA

D.yak. AAAA-AAAAAAAAA----AAGTGTCTAAAATTCTA---CGTCTGT-TGC---------TA

**D. yak. S2.20 Rvs**

D.mel. AAAT-AGAACAAAA----CTAAATCAAAAATGCTAC--CATCTGC-TGC---------TA

**S2.20 Rvs**

D.sim. AATTAAAAAAAAAA----CTAAATCAAAAATGCTAC--CATCTGC-TGC---------TA

**D. sim. S2.20 Rvs**

**

D.wil. AGCTCGTACA-TAAGATACTTATGTGAACATACCCTTTCTTTATACCAATAATTACTGAG

D.pse. GGCGCGACTAACGAG----TACCGTGG-----------------------AAACTCTGAT

D.bia. GC----CCTATCGAGGTCCCACTGCACTCATA------------------AATCGCAGCG

**D. bia. S2.20 Rvs**

D.yak. GTCGCACTCAACGAGGCCCCACTGTAGACATA------------------AATCGCAGCG

D.mel. GTCGCACTCAACGAGGTCCCACTGTAGACATA------------------AATCGCAGCG

D.sim. GTCGCACTCAACGAGACCCCACTGTAGACATA------------------AATCGCAGCG

* ** * ** * *

D.wil. TGCATTAAAATGGCATTTCAATATAACAGTATTTGTTGTAGATTTGTTGCTATTGCTCTT

D.pse. TTGGTCA-----------TAAAGTAACTGTATTTGTTGTACGAGTGGGAAGGCTGC----

**D. pse. S2.20 Rvs**

D.bia. TGGGCCA-----------TAAACTAACAGTATTTGTTG---------------TGC----

D.yak. TGGGCTA-----------TAAACTAACAGTATTTGTTG---------------TGC----

D.mel. TGGGCCA-----------TAAACTAACAGTATTTGTTG---------------TGC----

D.sim. TGGGCCA-----------TAAACTAACAGTATTTGTTG---------------TGC----

* * ** **** ********** ***

D.wil. GCAGTGGCGGCCTTAATTTGCATTTCACAAAGGTCGGTCGCACCTCGCGGTGGGTCGATG

D.pse. ---------TGCTCAATTTGCATT------------------------------------

D.bia. ---------AGCCTAATTTGCATT-----------------------------ATCGA-G

D.yak. ---------AGCCTAATTTGCATT-----------------------------ATCGA-G

D.mel. ---------AGCCTAATTTGCATT-----------------------------ATCGA-G

D.sim. ---------AGCCTAATTTGCATT-----------------------------ATCGA-G

* **********

D.wil. CAAACGAGTGAATG-TATCTCGCTACTGTTAACTACTACAAACATACAAAAATC--AAAT

D.pse. ------------------TCACCTTTGCATTCCAGCGGTAAA---TCAACAATCTAAAAT

D.bia. TGAATAATTGAACGACACTTG-CTGGCATTTCTAGCTA-AAA---ACAACAATCTAAAA-

D.yak. TGAATAATTGAACGACACTTTCCTGTTCTTTCTAACTA-AAA---ACAACAATCTAAAA-

D.mel. TGAATAATTGAACGACACTTTCCTATTCTTTCTAGCTA-AAA---ACAACAATCT-AAA-

D.sim. TGAATAATTGAACGACACTTTCCTGTTCTTTCTAGCTA-AAA---ACAACAATCT-AAA-

** * * *** *** **** ***

D.wil. TCATATGCATATAT-ACGGACATACATACATCAACAAATGGTTTTTATTAGTTCCTAATG

D.pse. CTACTCAC-----TAAAAGGTGTACGGGTGTTA------------------------GTA

D.bia. CTACTGAC-----T-AGAGGTCTACAGGGGTTA--GTGTGG---------GA---CTATG

D.yak. CTACTGAC-----T-AGAGGTCTACAAAGGTTAGTGTGTGG---------GGTCTTTATG

D.mel. CTACTGAC-----T-AGAGGTCTACAAAGGTTA----GTGG---------GGACTTTATG

D.sim. CTACTGAC-----T-AGAGGTCTACAAAGGTTA----GTGG---------GGTCTTGATG

* * * * * *** * * *

D.wil. GATACAACAATTTCTACCAGTTAATTAGTTGGATT--AGTT-TTGAAAATCTT---ATAC

D.pse. AATATGT---------------A------TGGATAATGGATAATGTATATGGATATGGAT

D.bia. AGTATGA---------------AGACGATGGGATA--TGGTGGTGTTAATGTGTGTTTAC

D.yak. AATATGA---------------AATTGACGGGATG--GGGT-ATGATAATGTG---ATGC

D.mel. AATATGA---------------AATCGACGGGATG--GGAT-ATGATAATGTG---ATGT

D.sim. AATATGA---------------AATCGACGGGATG--GGAT-ATGATAATGTG---ATGT

** * **** * * ** **

D.wil. GAATCGTGTGACAAAATGCAATATAAAAAAAATATGTATATGTATATGTATTAGAATGGA

D.pse. G----GA-TGGAAATGTGTGT-------------------GT----TAGAAGTCAAATCA

D.bia. G----GC-TGTAATAGTGTATGCAAGCTAAAGTATCTCCGGG----TGCATTCCAAATCA

D.yak. G----GTGTGCAATGGTGTAT---------AGTATCTCAGGG----TGCATTCCAAATCA

D.mel. G----GTGTGCAATGGTGTAC---------AGTATCTCCGGG----TGCATTCCAAATCA

D.sim. G----GTGTGCAATTGTGTAT---------AGTATCTCCGGG----TGCATTCCAAATCA

* * ** * ** * * ** *

D.wil. ATATTGAATTCAAAGGCTACTA--------------------TACTCGTGGAAATTGACT

D.pse. ATAT----C---------------------------------TACGTGCACA------AT

D.bia. ATAT----CTGGCATGCTTATA--------------------TATAT-------------

D.yak. ATAT----CTGCGTTGCTTATACTTATACATATGTATGCAGGTATGTGCGCAAAT---AT

D.mel. ATAT----CTGGCATGCTTATA--------------------TATGTGCGCAAAT---AT

D.sim. ATAT----CTGGCATGCTTATA--------------------TATGTGCGCAAAT---AT

**** **

D.wil. CGACTG------------------------ACTGACTGACTCATCGCTGGATCAGACAAA

D.pse. TCAGGG------------------------ACCGACTGACTCATCGGCCAGTC-------

D.bia. -CAGTGCAATATATAT------TCACG---GCAGACTGACTCATCGGC--AGC-------

D.yak. CCAGTG--ATATACATATATCATCACGGCAGCAGACTGACTCATCGTC--AGC-------

D.mel. CCATTG--ATATATAT------TCACG---GCAGACTGACTCATCGGC--AGC-------

D.sim. CCAGTG--ATATATAT------TCACG---GCAGACTGACTCATCGGC--AGC-------

* * * ************* *

D.wil. CAACAAATTGTCGACGCCTATATCTATGTATATCCATTTATCTATGTATCTACACAGGCT

D.pse. --------CGG-------TCGTATCACTCGGATCT-CTTAATTACCTTTC-GCTCAGGGA

D.bia. --------CGGCGACGCCTTTTATCGCGGCGATCT-TTTAATTACCTTTCTGCCGAGCGA

D.yak. --------CGGCGACGCCTTTTATCGCGGCGATCT-TTTAATTACCTTGCTGGCGAGCGT

D.mel. --------CGGCGACGCCTTTTATCGCGGCGATCT-TATAATTACCTTGCTGTCGAGCGT

D.sim. --------CGGCGACGCCTTTTATCGCGGCGATCT-TATAATTACCTTGCTGTCGAGCGT

* * *** ** ** * * **

D.wil. GTACTCAAAATTAA---TTAGATTT-ACATCAT------TTTG-----TTTAATTACCTT

D.pse. GGGCAGGGAGGCTGCATTTAATATATATCCAATATCCAATTTAGGCAGATTTCAAACTCT

D.bia. GTATCCAGATGCAG-ATTGCGGCTCGAATCCGTGCCCGCTTCAGGGGTTGTTAATACCAC

D.yak. GTATCCAGATGCAG-ATTGAGATTCGATACCGT------TTCAGGGGTTGTTGTTACCAT

D.mel. GTATCCAGATGCAG-ATTGAGATTCGATACCGT------TTCAGGGGTTGTTGTTACCAT

D.sim. GTATCCAGATGCAG-ATTGAGATTCGATACCGT------TTCAGGGGTTGTTGTTACCAT

* * * * * * ** * **

D.wil. TTGACTTTAAATATTTAATATTCATTTACAATAACAAAAAAAAAAAAGAAACTGAAACCA

D.pse. TT---------TTTTTTGTGTGCC---AAACAGGCAC-------GAAACAATC-------

D.bia. TGGGC-----ATCTCTAGTGC-----------------------GATATAATCCAAACTT

D.yak. TTGGC-----ATCCTTGGTGCACG---GAAATAAT------ACAAAAGTAATCCAGATTT

D.mel. TTGGC-----ATCCTTGGTGCACG---AATATAATACGAAAACGAAAATAATCCACACTT

D.sim. TTGGC-----ATCCTTGGTGCACG---AATATAATACGAAAACGAAAATAATCCACACTT

* * * * * **

D.wil. AGAGC------------ATTTGT---------ATTAGACACAAG----------------

D.pse. ------------------------ACAAAAACACCATACACAAA----------------

D.bia. GCCACTCGGCACGGTGTATTTTCAACAACAAAACGGCACAGAAACACAC-----------

D.yak. GGGAC------------AATTTC-ACAACAAAACGGCACAGAAACACACGCACTCCGAGC

D.mel. GGGAC------------AATTTC-ACAACAAAACGGGACAGAAACACAC---------GC

D.sim. GGGAC------------AATTTC-ACAACAAAACGGGACAGAAACACAC---------GC

* *** **

D.wil. ATTCACAATTTTTGCAAATGTATAATTTCTTTCTGGAATTTCCTTTGGTTTGCTC-TCTT

D.pse. ---TAATGTGACAG-GGACAGAGAGGACAGGGGGACG----------AATGGCGATTTTT

D.bia. --GCAAAACACTAGTAAACACAAAATACAGGGGTGGA----------GATTGCGA-TTTT

D.yak. ACTCAAAGCACTAGTAAACACAAAATACAGGGGTGGA----------GATTGCGA-TTTT

D.mel. ACTCAAAACACTAGTAAACACAAAATACAGGGGTGGA----------GATTGCGA-TTTT

D.sim. ACTCAAAACACTAGTAAACACAAAATACAGGGGTGGA----------GATTGCGA-TTTT

* * * * * * ** * **

D.wil. TGTTTTTGTTATTTTCATTCACTTATTCCTTCTTGCTTTTTGCTTTGACTTTCACATTCG

D.pse. CGTTT---TTATTTTCGTTCACTTAT---TTCTTGGTTTTTGCTTTGGCTTTCACATTCG

D.bia. CGTTT---TTATTTTCGTTCACTTAT---TTCTTGGTTTTTGCTTTGGCTCTCCCATTCG

D.yak. CGTTT---TTATTTTCGTTCACTTAT---TTCTTGGTTTTTGCTTTGGCTCTCACATTCG

D.mel. CGTTT---TTATTTTCGTTCACTTAT---TTCTTGGTTTTTGCTTTGGCTCTCACATTCG

D.sim. CGTTT---TTATTTTCGTTCACTTAT---TTCTTGGTTTTTGCTTTGGCTCTCACATTCG

**** ******** ********* ****** *********** ** ** ******

D.wil. CCGCGCGC-CACTCTCGC-ACTCT---CT--------CACAAAACAAAAACACAAAC---

D.pse. CCGCGCG-ACTCTCTCACAAAACAACACC----AACACACACAAAACACGAACACAAATG

D.bia. CCGCTCGCGCTCTCTCACTCCCTCGCTCCTTCTGTTGCACAAAACAAACGCACAAAAACA

D.yak. CCGCTCT-------------CTCGGCGCT--------CACAAAACAAACGCACAAGAACA

D.mel. CCGCTCTCTCACTCTGTC-GCTCTGCGCT--------CACAAAACAAACGCACAGGTACA

D.sim. CCGCTCTCTTTCGCTCTCTGCTCTGCGCT--------CACAAAACAAACGCACAGCTACA

**** * * **** ** * * ***

D.wil. -----------------------------TCGCTCTCTTTTATTTGTACGCTCTCTTCTC

D.pse. CACATATACAAAGACCG--ACTACTACTATTGCTCTCGTTCACGCTCGTGCTCTC-TTTC

D.bia. CGCACACACATTCACCGAAACGGC-----CCGCGCTCTCGCTATTGCG--CTCTC-TCTC

D.yak. CGCACACACGTACACCGCAATGGCCCGCTCCGCTCTCTTGCTATTTCGTTCTCTC-TCTC

D.mel. CGCACACACG--AACCGAAATGGC-----CCGCTCTCTTGCTATTTCGTTCTCTC-TCTC

D.sim. CGCACACACGTACACCGAAATGGC-----CCGCTCTCTTGCTATTCCGTTCTCTC-TCTC

** *** ***** * **

D.wil. GCGACCGCGCAT---GCACATACATATGTT---------------TTGTGTGTAAGTGTA

D.pse. GCGCCCGCGCGTTTCGCATATACACACACATGTATAGATTTGAGGCTGTGTGCGCGCGTG

D.bia. GCGCCCGCGCGTTCCGTATATACATAGATC------------------TGTCTGTAC---

D.yak. GCGCCCGCGCGTTTCGTATATACATATGTCGCAGCGTTTGTGTTCGCGTGTGTGTGCGGG

D.mel. GCGCCCGCGCGTTTCGTATATACATATGTTGCTACGTATGCGCGTGTGTGTGTGTGCGGG

D.sim. GCGCCCGCGCGTTTCGTATATACATATGTTGCTACGTTTGCGCGCGTGTGTGTGTGCGGG

*** ****** * * * ***** * ***

D.wil. TGTGTGTATGTATGTTTTTTTACGACGAT-GAAAAAAATGATGCCTGCTTCTTCTTC-AT

D.pse. TGTCTGCATGTATGTATTTTTACGACGATGGAAAA-----ATGCCTGCTTCTTCTTCTAT

D.bia. -GTATGTATGTACGGA-TTTTACGACGAT-GAAAA-----ATGCCTGCTTCTTCTTC-AC

D.yak. TGTCTGTATGTACGAG-TTTTACGACGAT-GAAAA-----ATGCCTGCTTCTTCTTC-AC

D.mel. TGTCTGTATGTATGGG-TTTTACGACGAT-GAAAA-----ATGCCTGCTTCTTCTTC-AC

D.sim. TGTCTGTATGTATGGG-TTTTACGACGAT-GAAAA-----ATGCCTGCTTCTTCTTC-AC

** ** ***** * ************ ***** ***************** *

D.wil. CCGAATCAGGTTTTCAACATTTCTTTTTCACATTTTTTCAGCATATT--CCAGTGGAATT

D.pse. ACAAA-------------------------------------------------------

D.bia. AAAAATCAGGTTTTCTGCTCTCGATTTTCACACTTTTTCGGCA-ATTGCCCGTCGCAAAT

D.yak. AGAAATCAGGTTTTCTGCTCTCGATTTTTACACTTTCTTGGCA-ACTGAACGCTACAAAT

D.mel. AGAAATCAGGTTTTCTGCTCTCGATTTTCACACTTTTTTAACA-ATTGAACGCCACAAAT

D.sim. AGAAATCAGGTTTTCTGCTCTCGATTTTCACACTTTTTTGGCA-ATTGAACGCCACAAAT

**

D.wil. TTAATGCAATTCAAACATTAAACAGTGAACATAACACTTATAAACACTTTTCACACAAAT

D.pse. ------------------------------------------------------------

D.bia. TCGATGCAATTCCACGGGCAGTCACTGCCCAGCACACTCGTACACACTTTTCACCACAGT

D.yak. TCGATGTAATTTCATTGGC-----------------------------------------

D.mel. TCGATGTAATTTCATTGGCAGACACTGCATACCATATTCACACACACTATTTAGCACAGT

D.sim. TCGATGTAATTTCATTGGCAGACACT----------------------------------

D.wil. TTAGCAATTATTTTCAATATTTTCCACGATTTTTCACAATTTTTTCGAGGACGAGTAGTT

D.pse. ------------------------------------------------------------

D.bia. TTATCAATTATTTTCCACATTTTCTGCCTTTTTTGAC-----------------------

D.yak. ------------------------------------------------------------

D.mel. TTATCAATTATTTTCCACATTTTCTGCGTTTTTTCGA-----------------------

D.sim. ------------------------------------------------------------

D.wil. TGTGTGTGTCTCTCTCACGAAATCGAACAAAAGAGAATGAAAAAAACACTATTTCCCGAC

D.pse. ------------------------------------------------------------

D.bia. -----------------------GAG----------------------------------

D.yak. ------------------------------------------------------------

D.mel. -----------------------CGAGCGAAATGGAAATAGCAAAGCAAAACGACCGGAC

D.sim. ------------------------------------------------------------

D.wil. ATTATGTCACTTTTCAACACTGTTAAACCTAGAACGGTGTTAAAAAAAACACTCATCACG

D.pse. ------------------------------------------------------------

D.bia. ------------------------------------------------------------

D.yak. ------------------------------------------------------------

D.mel. AGTGTGACCGCGCCGATAACTATAAAAAATACTGTGAGACTTACAAATAAATTCA-----

D.sim. ------------------------------------------------------------

D.wil. TTCGCGCGTTTTACTCACGATATCGAACTTGTTCGAATGAAAAAGCACTATTTCCCGACA

D.pse. ------------------------------------------------------------

D.bia. ------------------------------------------------------------

D.yak. ------------------------------------------------------------

D.mel. TGTGTGTGCAGACCTGGTCACACCGATATT------------------------------

D.sim. ------------------------------------------------------------

D.wil. TTATTTCACTTTTCAATACTATTAAAGCTGGGACAGTGTTAA

D.pse. ------------------------------------------

D.bia. ------------------------------------------

D.yak. ------------------------------------------

D.mel. ------------------------------------------

D.sim. ------------------------------------------
