# Supplementary material for: A novel role for trithorax in the gene regulatory network for a rapidly evolving fruit fly pigmentation trait
Source: PLoS Genet. 2023 Feb 16;19(2):e1010653. doi: 10.1371/journal.pgen.1010653 (PMC9977049; doi:10.1371/journal.pgen.1010653)
Supplement: S1 Table — (DOCX) [file pgen.1010653.s021.docx]

**S1 Table. 232 predicted abdominal pigmentation CREs in the *D. melanogaster* genome from the SCRMshaw analysis with the first training set of pigmentation GRN CREs.**

| **Coordinates (pCRE)** | **Score** | **Method** | **Proximal Gene 1** | **Proximal Gene 2** |
| --- | --- | --- | --- | --- |
| 2L:701750-702250 | 20.3197 | hexmcd | *ds* | *CR44988* |
| 2L:1618000-1618500 | 0.4083 | pac | *CR44603* | *RFeSP* |
| 2L:1995500-1996000 | 0.4604 | pac | *CG33543* | *Obp22a* |
| 2L:2940750-2941250 | 0.4102 | pac | *lilli* | *Rbp9* |
| 2L:3383750-3384250 | 11.1215 | imm | *CR45301* | *Sfp23F* |
| 2L:3557250-3557750 | 0.4196 | pac | *CR44309* | *CR44747* |
| 2L:4045000-4045500 | 15.609 | imm | *ed* | *CR44984* |
| 2L:4154750-4155250 | 12.8543 | imm | *CG2955* | *Or24a* |
| 2L:4891500-4892250 | 19.9427 | imm | *CR45716* | *CG15625,CG3036* |
| 2L:5500500-5501000 | 20.3808 | hexmcd | *CR45287* | *CG14020* |
| **2L:6082000-6082500**  **(S1.8)** | 0.4316 | pac | *Kr-h1* | *CR44773* |
| 2L:7530750-7531250 | 18.0842 | hexmcd | *RapGAP1* | *CR44082* |
| 2L:7828250-7828750 | 18.0999 | hexmcd | *mts* | *Rack1* |
| 2L:7901250-7901750 | 14.2673 | imm | *Snoo* | *Spn28Db* |
| 2L:7922000-7922500 | 10.1183 | imm | *Snoo* | *Spn28Db* |
| 2L:7961000-7961500 | 19.1462 | hexmcd | *Snoo* | *CG7231* |
| 2L:8376750-8377250 | 10.2217 | imm | *CG13390* | *CG17294* |
| 2L:10827750-10828250 | 0.4091 | pac | *Nos* | *CG17134* |
| 2L:11807250-11807750 | 0.4205 | pac | *crol* | *CycY* |
| 2L:12421500-12422000 | 10.3313 | imm | *vir-1* | *CG6405* |
| 2L:12655750-12656250 | 21.2455 | hexmcd,imm | *pdm2* | *Ref2* |
| 2L:12987500-12988000 | 10.2335 | imm | *Vha68-3* | *CG12404* |
| 2L:14623750-14624250 | 20.4245 | hexmcd,imm | *osp* | *Adhr* |
| 2L:14664250-14664750 | 0.4396 | pac | *osp* | *Adhr* |
| **2L:15035500-15036500**  **(S1.7)** | 34.5537 | hexmcd,imm | *yellow-c* | *CIAPIN1* |
| 2L:15306000-15306500 | 0.4119 | pac | *CG15262* | *CR43764* |
| 2L:15907500-15908000 | 17.7227 | hexmcd | *beat-Ib* | *CR44867* |
| 2L:16464000-16464500 | 18.8038 | hexmcd | *dac* | *Idgf3* |
| 2L:16486250-16486750 | 18.4393 | hexmcd | *dac* | *CG4580* |
| 2L:16808750-16809250 | 14.452 | imm | *CG13280* | *CG13272* |
| 2L:16996250-16996750 | 0.4091 | pac | *CR45356* | *beat-IIIb* |
| 2L:19120000-19121250 | 44.6616 | hexmcd,imm,pac | *Ddc* | *l(2)37Cc* |
| 2L:19413750-19414250 | 0.4247 | pac | *Pax* | *Lectin-galC1* |
| 2L:19571000-19571500 | 0.4326 | pac | *spi* | *mir-2b-2* |
| 2L:20268500-20269000 | 0.4139 | pac | *CG17570* | *CG17571* |
| 2R:977500-978000 | 19.425 | hexmcd | *CG40498* | *CR41257* |
| 2R:1645500-1646000 | 19.1782 | hexmcd | *CG41520* | *CR40068* |
| 2R:5821250-5821750 | 0.4136 | pac | *dpr12* | *Or42b* |
| 2R:5980500-5981000 | 10.1491 | imm | *mle* | *Src42A* |
| 2R:8232750-8233250 | 18.4211 | hexmcd | *dpn* | *CR44275* |
| 2R:8325500-8326000 | 0.4296 | pac | *pdm3* | *CR44450* |
| 2R:8326750-8327250 | 0.4176 | pac | *pdm3* | *CR44450* |
| 2R:10199750-10200250 | 0.4158 | pac | *CG34221* | *Hr3* |
| 2R:10554000-10554500 | 18.4918 | hexmcd | *psq* | *acal* |
| 2R:10564000-10564500 | 0.4078 | pac | *psq* | *acal* |
| 2R:10658250-10658750 | 0.4078 | pac | *CR44452* | *CG12934* |
| 2R:11416250-11416750 | 0.4141 | pac | *Drip* | *CG7763* |
| 2R:11420500-11421000 | 0.4439 | pac | *Drip* | *CG7763* |
| 2R:11626250-11626750 | 24.1638 | hexmcd | *CG9005* | *Egm* |
| 2R:12239500-12240000 | 20.8346 | hexmcd | *CG13170* | *CG43315* |
| 2R:14031250-14031750 | 19.6727 | hexmcd | *mam* | *CG18371* |
| 2R:15349250-15350000 | 25.0474 | hexmcd,imm,pac | *unc-5* | *Hr51* |
| 2R:15968500-15969000 | 17.5615 | hexmcd | *Strn-Mlck* | *CG8366* |
| 2R:17300750-17301250 | 0.4236 | pac | *mbl* | *CR43661* |
| 2R:17843000-17843750 | 14.6747 | imm | *grh,olf186-F* | *grh,olf186-F* |
| 2R:18331750-18332250 | 0.4126 | pac | *FBgn0010575* | *Tango8* |
| 2R:18373500-18374000 | 0.4162 | pac | *CR42736* | *CG43202* |
| 2R:19050000-19050750 | 0.4307 | pac | *CG15116* | *CG15109* |
| 2R:19078750-19079250 | 0.4126 | pac | *5-HT1A* | *Ir56a* |
| 2R:19381000-19381500 | 17.6215 | hexmcd,imm | *CG10073* | *CG10062* |
| 2R:20070750-20071250 | 0.4137 | pac | *CR44631* | *CR44632* |
| 2R:20484500-20485000 | 0.4156 | pac | *CG12484* | *CR44643* |
| 2R:21110750-21111250 | 0.4257 | pac | *ktub* | *CG9394* |
| 2R:21151750-21152250 | 0.4253 | pac | *CG30389* | *CG15657* |
| 2R:21542250-21542750 | 10.6849 | imm | *Egfr* | *CG30283* |
| 2R:21847000-21847500 | 10.2674 | imm | *tRNA:Gly-TCC-2-2* | *tRNA:Gly-TCC-2-1* |
| 2R:22136500-22137000 | 22.1335 | hexmcd | *a* | *CG11269* |
| 2R:22156500-22157000 | 18.5462 | hexmcd | *a* | *CG34206* |
| 2R:22159500-22160000 | 22.4371 | hexmcd | *a* | *CG34206* |
| 2R:22546250-22547000 | 19.6 | hexmcd,imm | *px* | *gas* |
| **2R:23005250-23005750**  **(S1.9)** | 10.0863 | imm | *CG42741* | *CG9896* |
| 2R:23242000-23242500 | 0.4141 | pac | *yip3* | *RpL22-like* |
| 2R:23508750-23509250 | 17.526 | hexmcd | *CR43794* | *CG3906* |
| 2R:24544750-24545250 | 0.4282 | pac | *ITP* | *CG4622* |
| 2R:25026000-25026500 | 0.4142 | pac | *uzip* | *Nplp1* |
| 3L:794250-794750 | 0.4126 | pac | *CG13898* | *CR44537* |
| 3L:1084500-1085500 | 50.7112 | hexmcd,imm,pac | *bab1* | *CG9205* |
| 3L:1097250-1098500 | 64.477 | hexmcd,imm,pac | *bab1* | *bab2* |
| 3L:1122250-1122750 | 0.4367 | pac | *bab2* | *bab1* |
| 3L:1165750-1166250 | 10.0663 | imm | *bab2* | *CG13912* |
| 3L:1586250-1587000 | 10.3359 | imm | *CG13917* | *CG12004* |
| 3L:3543000-3543500 | 18.3396 | hexmcd | *Eip63E* | *CR45820* |
| 3L:4672000-4672500 | 10.2105 | imm | *axo* | *CG32236* |
| 3L:4698250-4698750 | 0.4264 | pac | *RhoGEF64C* | *CG13713* |
| 3L:5185000-5185500 | 17.5309 | hexmcd | *shep* | *Srp54k* |
| 3L:5488250-5488750 | 0.4085 | pac | *CG4835* | *CR44520* |
| 3L:5502250-5502750 | 0.4298 | pac | *CR44519* | *CG34342* |
| 3L:6854000-6854500 | 0.4679 | pac | *CR45115* | *CR45416* |
| **3L:7833500-7834000**  **(S1.12)** | 0.4194 | pac | *Pdp1* | *CG32369* |
| 3L:9675500-9676000 | 17.9113 | hexmcd | *fry* | *CG16717* |
| 3L:9693250-9693750 | 0.432 | pac | *CG6767* | *CR45804* |
| 3L:9695500-9696000 | 11.7221 | imm | *CR45121* | *CG6767* |
| 3L:9865750-9866250 | 18.3144 | hexmcd | *CG8108* | *iPLA2-VIA* |
| 3L:10536000-10536500 | 11.4442 | imm | *Rbfox1* | *CG6527* |
| 3L:10737000-10737500 | 17.7884 | hexmcd | *CG43245* | *NijA* |
| 3L:10854750-10855250 | 11.532 | imm | *tna* | *CR45169* |
| **3L:11010000-11010750**  **(S1.10)** | 21.2007 | hexmcd | *klu* | *snoRNA:Me18S-G962* |
| 3L:11251500-11252000 | 0.4195 | pac | *CR44717* | *CR44716* |
| 3L:11758750-11759250 | 10.7663 | imm | *CG6024* | *Gr68a* |
| 3L:12348000-12348500 | 10.5606 | imm | *CrzR* | *CR45444* |
| 3L:12576250-12576750 | 18.2461 | hexmcd | *sowah* | *ara* |
| 3L:13170250-13170750 | 0.4176 | pac | *snky* | *CG33262* |
| 3L:13338250-13338750 | 10.7495 | imm | *CR44559* | *CG14113* |
| 3L:15477250-15477750 | 19.1438 | hexmcd | *CG7804* | *Ran-like* |
| 3L:15479750-15480250 | 0.4159 | pac | *Ran-like* | *CG7804* |
| 3L:16021750-16022250 | 19.2293 | hexmcd | *CR45998* | *CR43949* |
| 3L:16408750-16409250 | 0.4392 | pac | *fax* | *TMS1* |
| 3L:17056250-17056750 | 18.5231 | hexmcd | *CG7724* | *rogdi* |
| 3L:17590750-17591250 | 18.7975 | hexmcd | *Eip74EF* | *snoRNA:Me28S-A576* |
| **3L:17601000-17601500**  **(S1.11)** | 17.6943 | hexmcd | *Eip74EF* | *Vps60* |
| 3L:17920000-17920500 | 0.4133 | pac | *CG5290* | *CG14353* |
| 3L:18097000-18097500 | 0.4354 | pac | *CG13698* | *mRpS26* |
| 3L:18627750-18628250 | 0.4205 | pac | *MYPT-75D* | *bora* |
| 3L:20579000-20579500 | 0.4124 | pac | *knrl* | *Rcd2* |
| 3L:21008500-21009000 | 10.9635 | imm | *skd* | *scaRNA:PsiU6-40* |
| 3L:22910000-22910500 | 0.4262 | pac | *CR45661* | *CG12768* |
| 3L:27170500-27171000 | 9.92381 | imm | *CR43170* | *vtd* |
| 3R:6074000-6074500 | 0.4201 | pac | *Or83c* | *Gasp* |
| 3R:6101000-6101500 | 0.4219 | pac | *Gasp* | *CR44929* |
| 3R:6258750-6259250 | 10.6484 | imm | *Osi9* | *Osi8* |
| 3R:6285500-6286000 | 10.7783 | imm | *CG15597* | *CG15594* |
| 3R:8335250-8335750 | 18.8787 | hexmcd | *Poxm* | *COX7A* |
| 3R:8550750-8551250 | 11.9283 | imm | *Or85d* | *CR45574* |
| 3R:8947250-8947750 | 0.4167 | pac | *CG11964* | *CG11966* |
| 3R:9925750-9926250 | 0.4114 | pac | *Glut4EF* | *CR45029* |
| 3R:9981750-9982250 | 0.4141 | pac | *Glut4EF* | *Art4* |
| 3R:10216500-10217000 | 19.1911 | hexmcd,imm,pac | *Syn* | *Timp* |
| 3R:10586750-10587250 | 18.4839 | hexmcd | *hth* | *CR44018* |
| 3R:10964500-10965000 | 19.0637 | hexmcd | *CR45561* | *CG34114* |
| 3R:11154000-11154500 | 18.2122 | hexmcd | *Ugt86Di* | *Ugt86Dc* |
| 3R:11391000-11391500 | 0.4259 | pac | *pros* | *mRpL40* |
| 3R:13334250-13334750 | 0.4126 | pac | *PK2-R2* | *PK2-R1* |
| 3R:13395500-13396000 | 0.4167 | pac | *CCHa2* | *CG14374* |
| 3R:13453500-13454000 | 0.4129 | pac | *CG14372* | *CR17025* |
| 3R:13475000-13475500 | 20.7663 | hexmcd | *CR45589* | *CR45914* |
| 3R:13787000-13787500 | 19.1225 | hexmcd,imm | *CR45590* | *Dip-B* |
| 3R:14285000-14285500 | 17.9424 | hexmcd | *trx* | *CR45596* |
| 3R:14346750-14347250 | 12.0388 | imm | *NK7.1* | *HEATR2* |
| 3R:14370250-14370750 | 20.9533 | hexmcd | *NK7.1* | *snoRNA:Me18S-G1189* |
| 3R:14373500-14374250 | 23.6082 | hexmcd,imm | *NK7.1* | *CR44943,snoRNA:Me18S-G1189* |
| 3R:14438250-14438750 | 10.3849 | imm,pac | *FBgn0086901* | *CR44176* |
| 3R:14719000-14719500 | 18.5932 | hexmcd | *kibra* | *CG7530* |
| 3R:14739000-14739500 | 0.4229 | pac | *eff* | *jvl* |
| **3R:16020250-16020750**  **(S1.13)** | 0.4106 | pac | *GATAe* | *pnr* |
| 3R:16415000-16415500 | 10.7461 | imm | *CR45630* | *tRNA:Thr-AGT-1-3* |
| 3R:16536000-16536500 | 24.4376 | hexmcd,imm | *CG42342* | *CR46030* |
| 3R:16702500-16703000 | 20.433 | hexmcd,imm | *Ubx* | *bxd* |
| 3R:16703500-16704000 | 10.917 | imm | *Ubx* | *bxd* |
| **3R:16949500-16950000**  **(S1.14)** | 0.4219 | pac | *Abd-B* | *CR46267* |
| 3R:17210000-17210500 | 0.4128 | pac | *CG31262* | *Mur89F* |
| 3R:18033500-18034000 | 0.413 | pac | *CG7794* | *CG7785* |
| 3R:18122750-18123250 | 10.1762 | imm | *sr* | *tRNA:Gly-TCC-1-4* |
| 3R:18424750-18425250 | 17.6023 | hexmcd | *fru* | *CG31122* |
| 3R:18450000-18450500 | 0.4153 | pac | *fru* | *CG31122* |
| 3R:18485500-18486000 | 0.4151 | pac | *fru* | *CG31122* |
| 3R:18798250-18798750 | 22.4589 | hexmcd,imm | *CG18208* | *CG7720* |
| 3R:18923000-18923500 | 18.4526 | hexmcd | *Xrp1* | *Mpc1* |
| 3R:19250750-19251500 | 19.5526 | hexmcd | *CG5217* | *unc79* |
| 3R:20417500-20418000 | 0.4092 | pac | *Gfrl* | *eIF3g2* |
| 3R:20513750-20514250 | 0.4181 | pac | *MFS9* | *Or92a* |
| 3R:20695000-20695500 | 0.4153 | pac | *Oamb* | *CR43452* |
| 3R:21241000-21241750 | 19.9761 | hexmcd,imm | *CG5892* | *e* |
| 3R:21604500-21605000 | 0.4089 | pac | *InR* | *CR43653* |
| 3R:21837500-21838000 | 11.7095 | imm | *Gr93a* | *Gr93b* |
| 3R:21967000-21967500 | 11.8644 | imm | *Eip93F* | *CG6332* |
| 3R:21971750-21972250 | 17.6368 | hexmcd | *Eip93F* | *CG6332* |
| 3R:21978750-21979500 | 20.5415 | hexmcd | *Eip93F* | *CG6332* |
| **3R:22001250-22001750**  **(S1.15)** | 24.0551 | hexmcd,imm | *Eip93F* | *CG6332* |
| 3R:22200250-22200750 | 0.4114 | pac | *SKIP* | *CR45224* |
| **3R:23145000-23145500**  **(S1.16)** | 0.4233 | pac | *hh* | *unk* |
| 3R:25164500-25165000 | 19.0227 | hexmcd | *lobo* | *dan* |
| 3R:25760500-25761000 | 12.6241 | imm | *LpR1* | *CG17197* |
| 3R:26404750-26405250 | 0.4172 | pac | *ppk15* | *CG5432* |
| 3R:28398250-28398750 | 0.4236 | pac | *beat-VI* | *CR46106* |
| 3R:28449250-28449750 | 14.2668 | imm | *beat-VI* | *CR46106* |
| 3R:28681500-28682000 | 0.4091 | pac | *CG9990* | *Sid* |
| 3R:29565750-29566250 | 0.4094 | pac | *Dr* | *CG7567* |
| 3R:30100250-30100750 | 18.4766 | hexmcd,pac | *sima* | *CR46112* |
| **3R:30337750-30338250**  **(S1.17)** | 17.894 | hexmcd,imm | *hdc* | *CR46114* |
| **4:231750-232500**  **(S1.18)** | 12.8812 | imm | *CG1674* | *yellow-h* |
| X:355000-355750 | 33.6585 | hexmcd,imm | *CG32816* | *y* |
| X:1705500-1706000 | 12.1104 | imm | *CG3795* | *a6* |
| X:3047750-3048250 | 17.6446 | hexmcd | *kirre* | *CG4116* |
| X:3762000-3762500 | 19.1455 | hexmcd | *Tlk* | *mir-4962* |
| X:3780750-3781250 | 0.4134 | pac | *Tlk* | *mir-4962* |
| **X:4246250-4246750**  **(S1.3)** | 18.5699 | hexmcd,imm | *Muc4B* | *CG43134* |
| X:4695000-4695500 | 0.4079 | pac | *ctp* | *l(1)G0334* |
| X:4965750-4966250 | 11.0734 | imm | *CR44833* | *CG15468* |
| X:5087250-5087750 | 19.224 | hexmcd | *rg* | *CG32767* |
| X:5356750-5357250 | 0.4213 | pac | *SK* | *CanB* |
| X:5560250-5560750 | 11.3617 | imm | *Vsx2* | *Vsx1* |
| X:6013000-6013500 | 13.8725 | imm | *mab-21* | *CR44499* |
| **X:7213500-7214000**  **(S1.4)** | 19.2639 | hexmcd,imm | *CG9650* | *CR44357* |
| X:8100250-8100750 | 17.8762 | hexmcd | *CG2258* | *Gclc* |
| X:8473000-8473500 | 21.8486 | hexmcd,imm | *CG33223* | *Cp7Fa* |
| X:8785500-8786250 | 18.1839 | hexmcd | *Lim1* | *CR45537* |
| X:8887500-8888000 | 17.8491 | hexmcd | *Moe* | *CG1885* |
| X:9226500-9227250 | 24.7264 | hexmcd,imm | *CG15370* | *Gr8a* |
| **X:9389500-9390000**  **(S1.5)** | 0.423 | pac | *mgl* | *BCL7-like* |
| **X:9774000-9774750**  **(S1.6)** | 13.2676 | imm | *CG1354* | *CG32698* |
| X:10209500-10210250 | 11.1404 | imm | *CG15309* | *CR43899* |
| X:10386500-10387000 | 0.4472 | pac | *flw* | *Psf3* |
| X:11317750-11318250 | 19.079 | hexmcd | *sisA* | *Ir10a* |
| X:11374750-11375250 | 11.1135 | imm | *dlg1* | *Tim8* |
| X:11544250-11544750 | 0.4094 | pac | *Drak* | *mir-2492* |
| X:11756250-11756750 | 0.4112 | pac | *m* | *CG9360* |
| X:12462250-12462750 | 19.0815 | hexmcd,imm | *Rab40* | *CG42258* |
| X:13165250-13165750 | 0.4088 | pac | *Lgr4* | *CG12096* |
| **X:13284500-13285000** **(S1.1)** | 13.4075 | imm | *HDAC4* | *CG15743* |
| X:13909250-13909750 | 19.6475 | hexmcd,imm | *mamo* | *CG11068* |
| X:13965000-13965500 | 20.0356 | hexmcd | *mamo* | *CG11068* |
| X:14029250-14029750 | 10.6803 | imm | *tRNA:Ser-AGA-2-1* | *tRNA:Ser-AGA-3-1* |
| X:14072500-14073000 | 12.8555 | imm | *tRNA:Ser-AGA-1-1* | *Ste:CG33236* |
| X:14548750-14549250 | 18.5803 | hexmcd | *CR45521* | *CR44110* |
| X:14570500-14571250 | 19.2713 | hexmcd,pac | *CG14414* | *mRpS25* |
| X:14653750-14654250 | 11.7724 | imm | *NetA* | *CG5321* |
| X:14739000-14739500 | 9.92782 | imm | *NetB* | *CG15890* |
| X:15439000-15439500 | 19.9449 | hexmcd | *Cngl* | *Scamp* |
| X:16534500-16535000 | 19.2075 | hexmcd,imm | *FBgn0264255* | *CG9903* |
| X:16673250-16673750 | 19.1838 | hexmcd | *CG13012* | *r* |
| X:16816250-16816750 | 19.3412 | hexmcd | *CG4829* | *CR44129* |
| X:16980750-16981250 | 0.4307 | pac | *CG45002* | *Ubr1* |
| X:17994750-17995250 | 17.9966 | hexmcd | *Sh* | *CG12672* |
| X:18411500-18412000 | 0.4258 | pac | *CG32549* | *CG15056* |
| X:18541750-18542250 | 11.8303 | imm | *CG15047* | *CG15042* |
| **X:19631250-19631750**  **(S1.2)** | 0.4117 | pac | *e(y)3* | *CG14212* |
| X:19783250-19783750 | 11.621 | imm | *zld* | *CR44885* |
| X:19878500-19879000 | 10.6064 | imm | *Hers* | *amn* |
| X:21055000-21055500 | 0.4127 | pac | *bves* | *Stt3A* |
| X:21543750-21544250 | 17.5973 | hexmcd | *CR45082* | *Cyp6t1* |
| X:22799000-22799500 | 0.4088 | pac | *CR44997* | *CR45464* |
| Y:1155250-1155750 | 17.7624 | hexmcd | *CR40629* | *Su(Ste):CR45796* |

Note: Bolded font indicates sequences that were tested for enhancer activity in reporter transgene assays.
