# Supplementary material for: A novel role for trithorax in the gene regulatory network for a rapidly evolving fruit fly pigmentation trait
Source: PLoS Genet. 2023 Feb 16;19(2):e1010653. doi: 10.1371/journal.pgen.1010653 (PMC9977049; doi:10.1371/journal.pgen.1010653)
Supplement: S2 Table — (DOCX) [file pgen.1010653.s022.docx]

**S2 Table. Non-coding sequence controls with similar nucleotide contents to a *D. melanogaster* pigmentation GRN CRE.**

| **Coordinates** | **Upstream Gene** | **Downstream Gene** |
| --- | --- | --- |
| X:7749947-7751734 | *Tom40* | *Ir7a* |
| 2L:11922488-11924519 | *Pdc1c* | *Or33a* |
| 3L:27532906-27534457 | *Dbp80* | *eIF4B* |
