# Supplementary material for: A novel role for trithorax in the gene regulatory network for a rapidly evolving fruit fly pigmentation trait
Source: PLoS Genet. 2023 Feb 16;19(2):e1010653. doi: 10.1371/journal.pgen.1010653 (PMC9977049; doi:10.1371/journal.pgen.1010653)
Supplement: S3 Table — (DOCX) [file pgen.1010653.s023.docx]

**S3 Table.** Arbitrarily-selected transgenic lines that each possess a non-coding sequence-GAL4 transgene in the *att*P2 site. Males from these stocks were crossed to females of the w[1118]; P{w[+mC]=UAS-GFP.nls}14 stock (BDSC #4775).

| **BDSC#** | **gene** | **FBgn** | **symbol** |
| --- | --- | --- | --- |
| 39087 | *Ace* | FBgn0000024 | P{GMR54G07-GAL4}attP2 |
| 39374 | *CASK* | FBgn0013759 | P{GMR65G09-GAL4}attP2 |
| 40504 | *CG9932* | FBgn0262160 | P{GMR87H02-GAL4}attP2 |
| 41359 | *mamo* | FBgn0267033 | P{GMR75A04-GAL4}attP2 |
| 45013 | *pdm2* | FBgn0004394 | P{GMR11C12-GAL4}attP2 |
| 45241 | *cnc* | FBgn0262975 | P{GMR36G01-GAL4}attP2 |
| 45946 | *hh* | FBgn0004644 | P{GMR28D09-GAL4}attP2 |
| 45996 | *dpr5* | FBgn0037908 | P{GMR50E10-GAL4}attP2 |
| 47378 | *dpr18* | FBgn0030723 | P{GMR54G08-GAL4}attP2 |
| 47943 | *5-HT1B* | FBgn0263116 | P{GMR52F09-GAL4}attP2 |
| 49033 | *ana* | FBgn0011746 | P{GMR23E11-GAL4}attP2 |
| 49675 | *Ubx* | FBgn0003944 | P{GMR31D08-GAL4}attP2 |
| 50059 | *NK7.1* | FBgn0024321 | P{GMR39F11-GAL4}attP2 |
| 50279 | *E(z)* | FBgn0000629 | P{GMR46H03-GAL4}attP2 |
| 68384 | *none* | none | P{y[+t7.7] w[+mC]=GAL4.1Uw}attP2 |
