# Supplementary material for: A novel role for trithorax in the gene regulatory network for a rapidly evolving fruit fly pigmentation trait
Source: PLoS Genet. 2023 Feb 16;19(2):e1010653. doi: 10.1371/journal.pgen.1010653 (PMC9977049; doi:10.1371/journal.pgen.1010653)
Supplement: S4 Table — (DOCX) [file pgen.1010653.s024.docx]

**S4 Table. >700 predicted abdominal pigmentation CREs in the *D. melanogaster* genome from the SCRMshaw analysis with the second training set of CREs.**

| **Coordinates (pCRE)** | **Score** | **Score** | **Method** | **Proximal Gene 1** | **Proximal Gene 2** |
| --- | --- | --- | --- | --- | --- |
| 3R:18243700-18244200 | 17.1 | 17.1029 | imm | *14-3-3epsilon* | *CG7156* |
| 2R:20110880-20111690 | 124.3 | 19.1672 | imm | *18w* | *CR44633* |
| 2R:20109310-20109870 | 123.3 | 18.2403 | imm | *18w* | *CR44633* |
| 2R:22156520-22157180 | 282.8 | 21.5981 | imm | *a* | *CG34206* |
| 2R:22159470-22160020 | 109 | 19.1514 | imm | *a* | *CG34206* |
| 3R:16830110-16830620 | 32.8 | 16.9014 | imm | *abd-A* | *iab-8* |
| 2R:14419350-14420520 | 1574.7 | 35.9343 | imm | *Achl* | *Sin1* |
| X:15356260-15356760 | 16.2 | 16.2832 | imm | *acj6* | *Pp1-13C* |
| X:2036040-2036620 | 164.4 | 20.1372 | imm | *Actn* | *usp* |
| 2R:13953220-13953820 | 178.6 | 19.4006 | imm | *AGO1* | *CG33155* |
| 3R:7108270-7108770 | 16.7 | 16.7066 | imm | *Alh* | *CR45906* |
| X:11713970-11714620 | 48 | 16.0376 | imm | *Amun* | *CR45618* |
| 3L:16477050-16478110 | 538.3 | 27.247 | imm | *aos,CG33158* | *aos,CG33158* |
| X:9109950-9110480 | 69.3 | 18.1965 | imm | *AP-1gamma* | *Arfrp1* |
| 3L:12228390-12228930 | 97.8 | 21.2782 | imm | *app* | *CR43991* |
| 2R:23580430-23581230 | 641.4 | 29.9742 | imm | *apt* | *CG30184* |
| 3L:4638360-4638870 | 33.3 | 17.2414 | imm | *axo* | *CG13716* |
| 3L:4671790-4672480 | 415.9 | 23.3001 | imm | *axo* | *CG32236* |
| 3L:4663470-4664050 | 164.2 | 19.5778 | imm | *axo* | *CG32236* |
| X:17320340-17320870 | 72.9 | 19.037 | imm | *B-H2* | *CG5445* |
| 2L:13513120-13513620 | 16.5 | 16.5013 | imm | *B4* | *CG46308* |
| 3L:1097190-1098430 | 1732.9 | 42.5333 | imm | *bab1* | *bab2* |
| 3L:1108580-1109170 | 190.6 | 20.2203 | imm | *bab1* | *bab2* |
| 3L:1084550-1085620 | 1544.5 | 40.2125 | imm | *bab1* | *CG9205* |
| 3L:1064330-1064900 | 144.8 | 19.9018 | imm | *bab1* | *CG9205* |
| 3L:1122130-1122770 | 236.5 | 21.1417 | imm | *bab2* | *bab1* |
| 2L:2755140-2755800 | 374.8 | 26.3391 | imm | *Bacc* | *Pgk* |
| X:9152190-9152730 | 84.6 | 17.259 | imm | *Bap111* | *His3.3B* |
| 2L:3789440-3790220 | 552.5 | 23.9628 | imm | *bark* | *CR31958* |
| X:17170520-17171110 | 168.7 | 20.5442 | imm | *baz* | *xmas-2* |
| 3R:23569080-23569720 | 150.6 | 21.5991 | imm | *beat-IV* | *CG10164* |
| X:13994690-13995520 | 907 | 33.6103 | imm | *ben* | *CR44655* |
| X:4426550-4427470 | 1068.7 | 35.0731 | imm | *bi,CR32773* | *bi,CR32773* |
| X:4421820-4422730 | 1038.6 | 31.2588 | imm | *bi,CR32773* | *bi,CR32773* |
| X:4448780-4449320 | 71.7 | 19.2312 | imm | *bi,CR32773* | *bi,CR32773* |
| X:4423880-4424400 | 55.8 | 20.3807 | imm | *bi,CR32773* | *bi,CR32773* |
| 3L:5639880-5640390 | 37.7 | 19.4608 | imm | *Blimp-1* | *lin-28* |
| 3L:9102700-9103260 | 65.9 | 16.6809 | imm | *bol* | *CG13315* |
| 3L:9108020-9108560 | 49.7 | 16.8945 | imm | *bol* | *CG13315* |
| 3L:9124630-9125740 | 1019.7 | 31.1512 | imm | *bol* | *Dhpr* |
| 3L:9128580-9129140 | 109.8 | 19.8569 | imm | *bol* | *Dhpr* |
| 2L:12288320-12289190 | 861.6 | 26.5874 | imm | *bru1* | *CR45282* |
| 3L:13601050-13601570 | 50.6 | 17.1854 | imm | *bru3* | *CR45120* |
| 2L:8083370-8084190 | 269.7 | 19.4548 | imm | *Bsg* | *CR45257* |
| X:9693600-9694370 | 532.7 | 23.618 | imm | *btd* | *CR44016* |
| 2L:12507660-12508250 | 208.3 | 22.5361 | imm | *bun* | *CR43051* |
| 2L:12504710-12505350 | 70.1 | 18.289 | imm | *bun* | *CR43051* |
| 2L:12483260-12483900 | 308 | 22.875 | imm | *bun* | *t-cup* |
| X:21054870-21055570 | 176.3 | 19.4783 | imm | *bves* | *Stt3A* |
| X:6133310-6134120 | 725.4 | 27.2276 | imm | *Ca-alpha1T* | *Nep1* |
| X:6120170-6120930 | 564.8 | 27.3416 | imm | *Ca-alpha1T* | *Nep1* |
| 2R:12271180-12272030 | 440.8 | 22.8614 | imm | *Cam* | *CG42700* |
| X:16585480-16586020 | 78.2 | 21.6342 | imm | *CanA-14F* | *CG13014* |
| 2R:6062370-6062920 | 68 | 17.4187 | imm | *CCHa2-R* | *CG7856* |
| 2L:19479680-19480350 | 321.4 | 21.5578 | imm | *CG10195* | *CG13084* |
| 2R:20996870-20997410 | 88.3 | 17.9071 | imm | *CG10543* | *Prosalpha3* |
| X:12574590-12575400 | 541.9 | 25.1036 | imm | *CG11138* | *hwt* |
| X:12579800-12580560 | 478.9 | 24.959 | imm | *CG11138* | *LIMK1* |
| 2L:21899140-21899780 | 301.7 | 24.9043 | imm | *CG11629* | *CG1421,CR43762* |
| 3R:25237100-25237760 | 210.1 | 18.5255 | imm | *CG11852* | *CG10420* |
| X:8864250-8864770 | 50.8 | 17.3999 | imm | *CG12075* | *Moe* |
| 3L:10419200-10419860 | 120.1 | 18.0194 | imm | *CG12362* | *CR43990* |
| X:19719830-19720490 | 145.8 | 20.1646 | imm | *CG12531* | *CR44889* |
| X:7597240-7597760 | 52.7 | 18.4984 | imm | *CG12689* | *ct* |
| 2R:8060270-8061650 | 408.5 | 24.2025 | imm | *CG12769* | *CG17977* |
| X:16858930-16859510 | 161.9 | 19.7909 | imm | *CG13003* | *RSG7* |
| 3L:16528300-16528980 | 384.2 | 22.7205 | imm | *CG13033* | *st* |
| 2R:11364180-11365650 | 1224.1 | 30.6308 | imm | *CG13204,Gr47b* | *san,CG13204,Gr47b* |
| 2L:16808450-16809280 | 779.1 | 28.5521 | imm | *CG13280,CG13272* | *CG13280,CG13272* |
| X:9773680-9775060 | 2661.5 | 59.6054 | imm | *CG1354* | *CG32698* |
| 3L:1586160-1586970 | 490.2 | 21.7734 | imm | *CG13917* | *CG12004* |
| 2L:5511500-5512150 | 204.3 | 20.1609 | imm | *CG14020* | *CG7382* |
| 3L:18814000-18814520 | 52.7 | 18.4659 | imm | *CG14073* | *HipHop* |
| 3R:17666010-17666690 | 430.5 | 29.1708 | imm | *CG14322* | *CR45045* |
| 3R:17653720-17654220 | 16.9 | 16.9978 | imm | *CG14322* | *CR45223* |
| X:6854860-6856230 | 485.3 | 20.1044 | imm | *CG14434* | *CG4558* |
| X:6702470-6703160 | 309.1 | 19.3109 | imm | *CG14441* | *CG14440* |
| 3R:16591310-16591810 | 16.8 | 16.8815 | imm | *CG14892* | *CG10345* |
| X:18541400-18542340 | 1048.2 | 27.5705 | imm | *CG15047* | *CG15042* |
| X:18545730-18546430 | 142.4 | 19.1628 | imm | *CG15047* | *CG15042* |
| X:18534400-18534960 | 141.9 | 18.6129 | imm | *CG15047* | *CG15042* |
| X:18542600-18543170 | 126.4 | 20.0621 | imm | *CG15047* | *CG15042* |
| X:9064320-9065020 | 396.7 | 20.6372 | imm | *CG15365* | *CG10970* |
| 2L:3482940-3483620 | 163.1 | 19.8947 | imm | *CG15414* | *Thor* |
| 2L:4749060-4749580 | 50.7 | 17.159 | imm | *CG15630* | *CG42523* |
| 2R:16483510-16484180 | 289.3 | 26.2412 | imm | *CG15712* | *CG33458* |
| X:7300380-7301400 | 1101.3 | 26.8956 | imm | *CG1677,CR44357* | *CG1677,CR44357* |
| X:7307370-7308220 | 803.3 | 26.4499 | imm | *CG1677,CR44357* | *CG1677,CR44357* |
| 2L:12950390-12950970 | 142.5 | 19.3743 | imm | *CG16800* | *CR44183* |
| X:20436850-20437460 | 168.5 | 18.2965 | imm | *CG1695* | *mal* |
| 2L:2267180-2267850 | 215.8 | 19.5596 | imm | *CG17242* | *CG34049* |
| 2L:3000490-3001060 | 152.2 | 20.1915 | imm | *CG17265* | *CG3558* |
| 2R:13628030-13628530 | 33.8 | 17.9945 | imm | *CG17716* | *CG12464* |
| 3R:18265110-18265690 | 179.5 | 21.7745 | imm | *CG18599* | *Vps39* |
| 2R:9547850-9548520 | 68.4 | 17.9027 | imm | *CG1888* | *CR43651* |
| X:8100000-8100630 | 243.4 | 20.4057 | imm | *CG2258* | *Gclc* |
| X:12497850-12498410 | 131.7 | 20.3407 | imm | *CG2556* | *snoRNA:Me18S-G894* |
| X:2357580-2358080 | 17.1 | 17.1799 | imm | *CG2841* | *PsGEF* |
| X:2289220-2289830 | 203.5 | 20.9022 | imm | *CG2865* | *Raf* |
| X:2283150-2284120 | 1398.3 | 40.8403 | imm | *CG2865* | *Vml* |
| X:4779080-4779610 | 54.5 | 19.3162 | imm | *CG2871* | *CG15471* |
| 2R:21579860-21580390 | 76.2 | 20.0208 | imm | *CG30222* | *twz* |
| 2L:4891430-4892480 | 1840.8 | 42.1724 | imm | *CG3036,CR45716* | *CG15625,CG3036,CR45716* |
| 3R:29880170-29880820 | 322.6 | 23.5851 | imm | *CG31038* | *CG34133* |
| 3R:23965370-23965870 | 16.9 | 16.9212 | imm | *CG31140* | *AP-1sigma* |
| 3R:20104190-20104710 | 51.1 | 17.7952 | imm | *CG31206* | *CG10887* |
| 3L:19374550-19375050 | 17.6 | 17.6855 | imm | *CG32206* | *ms(3)76Ba* |
| 3L:3783930-3784520 | 157.6 | 18.4117 | imm | *CG32264* | *CG32266* |
| 3L:3771300-3771880 | 53 | 18.2739 | imm | *CG32264* | *ppk27* |
| 3L:7804510-7805110 | 67.3 | 17.4533 | imm | *CG32369* | *Pdp1* |
| X:16723390-16723970 | 101.9 | 18.1868 | imm | *CG32572* | *CG34325* |
| X:13143200-13143950 | 417.2 | 25.2183 | imm | *CG32645* | *MFS10* |
| X:12783940-12784470 | 67.9 | 17.5459 | imm | *CG32647* | *Pde9* |
| X:9992060-9992770 | 274.3 | 20.4384 | imm | *CG32694* | *CG15252* |
| X:4910450-4910950 | 16.5 | 16.5471 | imm | *CG32772* | *CG6927* |
| X:2789460-2789960 | 17.1 | 17.172 | imm | *CG32795* | *kirre* |
| X:355200-355700 | 16.5 | 16.5739 | imm | *CG32816* | *y* |
| 3R:27332560-27333190 | 266.5 | 21.4847 | imm | *CG3330* | *CG42534* |
| 2R:24299040-24299580 | 91.7 | 19.9281 | imm | *CG3394* | *mir-4985* |
| 3L:5952260-5952890 | 301.4 | 24.515 | imm | *CG33993* | *CG10479* |
| 3L:5955080-5955580 | 18.2 | 18.2489 | imm | *CG33993* | *CG10479* |
| X:6549370-6549990 | 119.3 | 17.4998 | imm | *CG34417* | *dx* |
| X:5935010-5935870 | 629.3 | 24.6402 | imm | *CG3726* | *CG12728* |
| X:3099210-3099750 | 92.9 | 21.2069 | imm | *CG4116* | *N* |
| X:13114400-13114930 | 70.3 | 19.5446 | imm | *CG42237* | *CG43313* |
| X:12471750-12472600 | 537 | 21.4752 | imm | *CG42258* | *Rab40* |
| 2L:14939840-14940430 | 145.4 | 20.3567 | imm | *CG42313* | *CG3491* |
| X:11001840-11002380 | 33.6 | 17.2527 | imm | *CG42339* | *C901* |
| X:18813960-18814770 | 487.4 | 24.285 | imm | *CG42450* | *CG7058* |
| 3L:14756950-14757880 | 900.8 | 21.3116 | imm | *CG42507* | *CG33260* |
| X:4865140-4865710 | 86.6 | 19.2661 | imm | *CG42594* | *CR43495* |
| 2R:23042770-23043520 | 357 | 21.4387 | imm | *CG42741* | *CG44252* |
| 2R:23006500-23007020 | 55.4 | 19.3534 | imm | *CG42741* | *CG9896* |
| X:5295000-5295820 | 327 | 24.7264 | imm | *CG42749* | *CG3323* |
| X:5280640-5281350 | 140.5 | 18.897 | imm | *CG42749* | *CG3323* |
| 2L:18234760-18235310 | 91.6 | 19.9344 | imm | *CG42750* | *CR43406* |
| 2L:13121700-13122270 | 149.9 | 20.7012 | imm | *CG43050* | *CR44198* |
| X:5862840-5863520 | 294.7 | 21.8233 | imm | *CG43137* | *CG15764* |
| 3L:10736610-10738080 | 1329 | 35.6568 | imm | *CG43245* | *NijA* |
| 3L:10739900-10740750 | 893.8 | 30.8776 | imm | *CG43245* | *NijA* |
| 3L:10739040-10739770 | 583.5 | 29.4006 | imm | *CG43245* | *NijA* |
| 3L:22500270-22500820 | 71.8 | 19.6963 | imm | *CG43312* | *CR45431* |
| X:10620210-10620710 | 16.6 | 16.638 | imm | *CG43347* | *Rab9Db* |
| 3R:23451750-23452370 | 176.5 | 19.9159 | imm | *CG4374* | *CG43694* |
| 3R:23472020-23472880 | 425.6 | 19.5027 | imm | *CG4374* | *Ir94f* |
| X:10344860-10345510 | 52.8 | 18.2756 | imm | *CG43740* | *snoRNA:U3:9B* |
| X:19006650-19007170 | 52.7 | 17.8441 | imm | *CG43759* | *Diedel3* |
| 3L:16683300-16684230 | 845.9 | 24.9166 | imm | *CG43954* | *CR45179* |
| 2L:2425540-2426370 | 537.9 | 23.2048 | imm | *CG44139* | *CG9886,CR44912* |
| 3R:23248600-23249300 | 189.1 | 17.8093 | imm | *CG4467* | *Rad60* |
| X:4937400-4938050 | 153 | 21.0613 | imm | *CG44774* | *Ptp4E* |
| 2R:7760810-7761340 | 74.4 | 20.6395 | imm | *CG45093* | *cn* |
| X:12278830-12279510 | 50.7 | 17.2744 | imm | *CG45603* | *CG32655* |
| 2L:13489550-13490170 | 279.5 | 25.4325 | imm | *CG46308* | *B4* |
| 3R:19889910-19890460 | 114 | 20.3128 | imm | *CG4733* | *ninaE* |
| 3R:19250560-19251740 | 996 | 33.577 | imm | *CG5217* | *unc79* |
| 2L:16422360-16423230 | 182.9 | 19.376 | imm | *CG5888* | *snoRNA:Me28S-A2958* |
| 2L:16521200-16521900 | 364.4 | 24.7559 | imm | *CG5953* | *mir-4943* |
| X:18315620-18316400 | 329.1 | 22.1092 | imm | *CG6023* | *CR45609* |
| 3R:19119650-19120260 | 32.9 | 16.8606 | imm | *CG6040* | *Cyp12a5* |
| 3R:27149790-27150400 | 216.5 | 24.1337 | imm | *CG6051* | *Men-b* |
| X:18347520-18348060 | 53.1 | 19.8207 | imm | *CG6123* | *CG6106* |
| 3R:21898630-21899150 | 34 | 17.4569 | imm | *CG6678* | *CG43844* |
| 3L:7591460-7592070 | 70.2 | 18.6114 | imm | *CG7716* | *Pura* |
| 3L:15507620-15508120 | 16.4 | 16.4467 | imm | *CG7841* | *Z600* |
| 2R:9012090-9012660 | 148.4 | 20.3965 | imm | *CG8172* | *CG13744* |
| X:17453180-17453860 | 271.1 | 23.3761 | imm | *CG8568* | *CG43658* |
| 2R:12079370-12079930 | 83.5 | 16.9734 | imm | *CG8888* | *CG13186* |
| 2R:11633130-11633630 | 17.1 | 17.1173 | imm | *CG9005* | *CG9003* |
| 2R:11626080-11627030 | 1044 | 31.9595 | imm | *CG9005* | *Egm* |
| 2L:5799690-5800230 | 93.6 | 20.2837 | imm | *CG9171* | *CG14005* |
| 2R:23004930-23006180 | 2489.9 | 58.4164 | imm | *CG9896,CG42741* | *CG9896,CG42741* |
| X:7696340-7697300 | 801.6 | 22.7717 | imm | *CHES-1-like* | *CR43287* |
| X:7702340-7702870 | 50.2 | 17.2048 | imm | *CHES-1-like* | *CR43287* |
| X:7692260-7692820 | 107.5 | 19.9566 | imm | *CHES-1-like* | *CR45665* |
| 2L:1669350-1669920 | 104.4 | 18.7153 | imm | *chinmo* | *CR45695* |
| 2L:1654120-1654700 | 103.4 | 18.2255 | imm | *chinmo* | *CR45695* |
| 2L:1650840-1651360 | 51.6 | 17.6159 | imm | *chinmo* | *CR45695* |
| 2L:1663240-1663750 | 36.9 | 18.6561 | imm | *chinmo* | *CR45695* |
| 3R:20292820-20293340 | 52.2 | 17.9372 | imm | *cic* | *CG4367* |
| 3R:20283970-20284520 | 34.7 | 17.4217 | imm | *cic* | *CG4367* |
| 3L:7344520-7345030 | 34.1 | 18.1945 | imm | *Cln7* | *CG14834* |
| 3L:5067070-5067600 | 86.5 | 18.3848 | imm | *Con* | *CR43884* |
| **3R:5087760-5088450 (S2.22)** | 421.9 | 27.3574 | imm | *corto* | *CG43131* |
| **3R:5086870-5087550**  **(S2.21)** | 264.2 | 19.3585 | imm | *corto* | *CG43131* |
| 3R:18007580-18008130 | 50.8 | 18.1772 | imm | *cpo* | *DNaseII* |
| 3R:17946850-17947440 | 90.8 | 19.9059 | imm | *cpo* | *Rim* |
| 3R:11281900-11282600 | 188.5 | 21.2438 | imm | *CR31386* | *lncRNA:TS25* |
| X:4602200-4602770 | 66.1 | 16.88 | imm | *CR32773* | *peb* |
| 3L:616400-616900 | 16.6 | 16.648 | imm | *CR42719* | *CG43337* |
| X:4003470-4003970 | 18.3 | 18.3141 | imm | *CR43298* | *CR45481* |
| X:11740370-11741280 | 944.1 | 27.859 | imm | *CR43385* | *CG15221* |
| X:9714100-9714810 | 67.7 | 17.4439 | imm | *CR43498* | *Sp1* |
| 3L:3876740-3877340 | 243.8 | 25.4205 | imm | *CR43917* | *CR45379* |
| X:12727670-12728380 | 299.2 | 21.8847 | imm | *CR43963,Smr* | *CR43963,Smr* |
| 2L:2530110-2530620 | 34.3 | 18.0714 | imm | *CR44112* | *CR46069* |
| 2R:12984500-12985270 | 407.8 | 22.9606 | imm | *CR44339* | *Psc* |
| 2R:15104770-15105560 | 611.3 | 28.8316 | imm | *CR44457* | *Rpb12* |
| 2R:15112690-15113240 | 108.2 | 19.5878 | imm | *CR44457* | *Rpb12* |
| 3L:2881320-2881840 | 55.3 | 19.1301 | imm | *CR44531* | *Tet* |
| 3L:10787660-10788450 | 711.2 | 30.9538 | imm | *CR44545* | *CR44544* |
| 2L:1629100-1629710 | 49.5 | 17.0144 | imm | *CR44603* | *RFeSP* |
| 2R:20494490-20495200 | 543.9 | 28.2024 | imm | *CR44643* | *Obp57c* |
| X:13991850-13992350 | 16.4 | 16.485 | imm | *CR44655* | *mamo* |
| 2R:20841760-20842280 | 53.2 | 19.3752 | imm | *CR44667* | *insc* |
| 2R:22678440-22679350 | 341.2 | 19.9919 | imm | *CR44761* | *RYBP* |
| X:5046760-5047520 | 551 | 27.6511 | imm | *CR44833* | *ovo* |
| X:5025550-5026120 | 87.1 | 18.9934 | imm | *CR44833* | *ovo* |
| X:5041940-5042450 | 33.9 | 17.0185 | imm | *CR44833* | *ovo* |
| 2L:18795870-18796390 | 52.5 | 18.1916 | imm | *CR44904* | *ham* |
| 2L:21846560-21847190 | 216 | 21.9666 | imm | *CR44917* | *tsh* |
| 3R:7037210-7037720 | 34.2 | 17.3272 | imm | *CR44933* | *Sodh-1* |
| 3R:10383170-10383850 | 67.8 | 17.9044 | imm | *CR44938* | *cwo* |
| X:21413800-21414330 | 72.8 | 19.192 | imm | *CR45003* | *DIP-beta* |
| 3R:18156770-18157270 | 16.9 | 16.9362 | imm | *CR45103* | *CR45104* |
| 3L:6308970-6309470 | 16.3 | 16.3548 | imm | *CR45113* | *CR45114* |
| **3L:6832930-6833560**  **(S2.17)** | 273.5 | 20.9583 | imm | *CR45115* | *vvl* |
| 3L:10852370-10852960 | 32.5 | 16.5297 | imm | *CR45169* | *tna* |
| 3L:10387940-10388840 | 767.4 | 24.8064 | imm | *CR45245* | *CR46006* |
| 3L:10933660-10934620 | 588.8 | 25.3502 | imm | *CR45248* | *CR45249* |
| 3L:10936420-10937120 | 434 | 25.2405 | imm | *CR45248* | *CR45249* |
| 3L:10937970-10938530 | 104.2 | 17.9245 | imm | *CR45248* | *CR45249* |
| 3L:10944400-10944950 | 84.6 | 17.1831 | imm | *CR45248* | *CR45249* |
| 3L:3866410-3867050 | 271.1 | 19.7807 | imm | *CR45379* | *Awh* |
| 3L:7218710-7219510 | 783.4 | 32.182 | imm | *CR45410* | *CG14826* |
| 3L:7225300-7225880 | 124.7 | 19.132 | imm | *CR45410* | *CG14826* |
| 3L:3246020-3246830 | 436.2 | 27.5672 | imm | *CR45424* | *CG12078,CG43389* |
| X:7118600-7119230 | 84.3 | 18.0256 | imm | *CR45532* | *fz4* |
| 3R:13787100-13787600 | 17.5 | 17.5978 | imm | *CR45590* | *Dip-B* |
| 3R:16218280-16219030 | 451.2 | 26.5302 | imm | *CR45641* | *CR45642* |
| 3R:16221430-16222030 | 104.9 | 19.5847 | imm | *CR45643* | *tara* |
| 3R:24768340-24768950 | 143.6 | 20.8966 | imm | *CR45650* | *CG13631* |
| X:5886700-5887420 | 295.2 | 19.4321 | imm | *CR45668* | *CG16721* |
| 3L:3710990-3711630 | 262.1 | 22.3603 | imm | *CR45675* | *CG43089* |
| 2L:17558300-17559480 | 1340.3 | 35.9001 | imm | *CR45919* | *CG7094* |
| 3L:10331970-10332630 | 270.9 | 19.6373 | imm | *CR46006* | *CR46004* |
| 3L:15535220-15535720 | 16.1 | 16.1054 | imm | *CrebA* | *CG43248* |
| X:18374260-18375230 | 1360.7 | 40.1308 | imm | *CrebB* | *por* |
| 2L:16267690-16268650 | 1219.8 | 32.4417 | imm | *crp* | *CG4935* |
| 2L:16284570-16285140 | 153.3 | 21.7296 | imm | *crp* | *pkaap* |
| X:7626640-7627630 | 1064.6 | 30.4758 | imm | *ct* | *CG12689* |
| X:7604340-7605090 | 497.5 | 22.9441 | imm | *ct* | *CG12689* |
| X:7607890-7609080 | 374.7 | 23.4428 | imm | *ct* | *CG12689* |
| X:7605680-7606230 | 105.7 | 18.5289 | imm | *ct* | *CG12689* |
| X:7615760-7616280 | 54.5 | 19.5507 | imm | *ct* | *CG12689* |
| X:7632720-7633490 | 592.9 | 26.5732 | imm | *ct* | *CG12689,CR45667* |
| X:7661620-7662160 | 85.6 | 17.7989 | imm | *ct* | *CR45667* |
| 3R:13011880-13012770 | 783.9 | 23.5166 | imm | *CtBP* | *CG8031* |
| X:4689450-4689980 | 69.8 | 17.9615 | imm | *ctp* | *Pp2C1* |
| 3L:15105620-15106240 | 257.4 | 22.1467 | imm | *CTPsyn* | *CG45071* |
| 3R:4798010-4798530 | 58 | 19.5511 | imm | *ctrip* | *Hus1-like* |
| 2R:21364370-21364870 | 16.7 | 16.7745 | imm | *cv-2* | *CG17974* |
| 3R:14468500-14469130 | 216.1 | 18.766 | imm | *cv-c* | *CR44177* |
| 3R:14390060-14390570 | 37.1 | 18.739 | imm | *cv-c* | *mir-2281* |
| 3R:29204890-29205430 | 87.4 | 18.1366 | imm | *Cyt-c1L* | *Mesh1* |
| X:1302990-1303600 | 107.8 | 19.9406 | imm | *DAAM* | *CR46248* |
| 2L:19120080-19121070 | 931 | 24.9308 | imm | *Ddc* | *l(2)37Cc* |
| 2R:16095200-16095840 | 312.9 | 25.5518 | imm | *Dg* | *mRpL34* |
| X:21418210-21418900 | 216.8 | 20.1293 | imm | *DIP-beta* | *CR45003* |
| 3R:28956980-28957480 | 17.3 | 17.3885 | imm | *DIP-gamma* | *CR45669* |
| X:16216470-16217760 | 749.9 | 24.8027 | imm | *disco* | *CR46211* |
| X:16206010-16206610 | 150.9 | 20.8379 | imm | *disco* | *CR46211* |
| X:16148620-16149470 | 524.3 | 19.5031 | imm | *disco-r* | *CR46211* |
| X:16150650-16151150 | 18.8 | 18.8685 | imm | *disco-r* | *CR46211* |
| 3R:19321580-19322530 | 1014.2 | 28.3797 | imm | *Dl* | *CR44175* |
| 2L:17449130-17450110 | 1319.9 | 33.1669 | imm | *dl* | *CR46204* |
| X:11370010-11370650 | 274.2 | 21.9881 | imm | *dlg1* | *Tim8* |
| 2L:19361950-19362480 | 71.1 | 19.1077 | imm | *dnt* | *CG13086* |
| 3R:29633240-29633890 | 317.4 | 22.4779 | imm | *Dop1R2* | *CG1907* |
| 2R:13223330-13223830 | 16.9 | 16.9748 | imm | *Dp* | *CR43918* |
| 3R:11515360-11515870 | 33.8 | 17.0761 | imm | *dpr5* | *dpr4* |
| X:14377100-14377910 | 251.9 | 19.899 | imm | *dpr8* | *betaNACtes3* |
| X:14375020-14375520 | 16.5 | 16.5459 | imm | *dpr8* | *betaNACtes3* |
| X:14324430-14324930 | 16.5 | 16.5435 | imm | *dpr8* | *betaNACtes6* |
| X:11519930-11520470 | 92.1 | 19.3803 | imm | *Drak* | *CR44556* |
| 2L:701520-702410 | 1092.5 | 32.8482 | imm | *ds* | *CR44988* |
| 3L:7199350-7200120 | 267.9 | 22.8912 | imm | *Dscam2* | *CR45410* |
| 3L:8266880-8267410 | 34 | 17.2353 | imm | *Dscam4* | *cert* |
| 3L:8242910-8243530 | 252.1 | 23.1716 | imm | *Dscam4* | *CG7213* |
| 2R:22270620-22271160 | 90.5 | 20.3104 | imm | *dve* | *CR43422* |
| X:11778630-11779330 | 481.2 | 25.9445 | imm | *dy* | *Karl* |
| X:19631060-19632020 | 1440.3 | 45.162 | imm | *e(y)3* | *CG14212* |
| 3R:13874870-13875470 | 205.5 | 20.3052 | imm | *E5* | *CR46239* |
| 3L:5610430-5611140 | 441 | 24.5371 | imm | *Eaf6* | *mthl2* |
| X:14957560-14958060 | 16.9 | 16.9136 | imm | *eag* | *CG9030* |
| **3L:10196740-10197660**  **(S2.16)** | 876.3 | 27.7726 | imm | *ect* | *CR44538* |
| 2L:4053680-4054450 | 674.5 | 36.9382 | imm | *ed* | *CR44984* |
| 2R:11892610-11893270 | 101.3 | 17.9943 | imm | *eEF1alpha1* | *snoRNA:Me28S-A1322* |
| 3L:3543300-3544000 | 418.2 | 27.9606 | imm | *Eip63E* | *CR45820* |
| 3L:17590410-17591290 | 1084.2 | 36.8499 | imm | *Eip74EF* | *snoRNA:Me28S-A576* |
| 3L:17563370-17564290 | 999.2 | 34.9818 | imm | *Eip74EF* | *snoRNA:Me28S-A576* |
| 3L:17577700-17578430 | 519.7 | 26.1094 | imm | *Eip74EF* | *snoRNA:Me28S-A576* |
| 3L:17566390-17566970 | 166.8 | 20.1001 | imm | *Eip74EF* | *snoRNA:Me28S-A576* |
| 3L:17576340-17576890 | 111.6 | 19.7301 | imm | *Eip74EF* | *snoRNA:Me28S-A576* |
| 3L:17569800-17570560 | 50.7 | 17.6974 | imm | *Eip74EF* | *snoRNA:Me28S-A576* |
| 3L:17600900-17601890 | 817.9 | 25.0794 | imm | *Eip74EF* | *Vps60* |
| 3L:17599880-17600740 | 725.8 | 22.4004 | imm | *Eip74EF* | *Vps60* |
| 3L:17611620-17612290 | 230.9 | 18.6674 | imm | *Eip74EF* | *Vps60* |
| 3L:17608870-17609380 | 33.2 | 17.0248 | imm | *Eip74EF* | *Vps60* |
| 3L:17964550-17965460 | 233.2 | 19.8778 | imm | *Eip75B* | *CR45921* |
| 3L:17968570-17969090 | 55 | 18.9399 | imm | *Eip75B* | *CR45921* |
| 3L:18029390-18029950 | 105.1 | 18.6332 | imm | *Eip75B* | *CR45922* |
| 3L:17995220-17995980 | 405.9 | 23.2229 | imm | *Eip75B* | *snoRNA:Me28S-A30* |
| 3L:21225680-21226580 | 512.1 | 25.8299 | imm | *Eip78C* | *CG43219* |
| 3L:21239760-21240430 | 369 | 23.4042 | imm | *Eip78C* | *CG43219* |
| 3R:21949020-21949960 | 1039.2 | 27.1402 | imm | *Eip93F* | *CG17843* |
| 3R:21935640-21936170 | 68.7 | 17.6718 | imm | *Eip93F* | *CG17843* |
| 3R:21951280-21951780 | 17.4 | 17.4862 | imm | *Eip93F* | *CG17843* |
| 3R:22001150-22001980 | 899.2 | 37.0611 | imm | *Eip93F* | *CG6332* |
| 3R:21978860-21979470 | 220.7 | 22.6658 | imm | *Eip93F* | *CG6332* |
| 3R:22000010-22000630 | 201.9 | 20.7849 | imm | *Eip93F* | *CG6332* |
| 3R:21973090-21973650 | 110.3 | 20.3435 | imm | *Eip93F* | *CG6332* |
| 2R:19142880-19143430 | 94 | 20.285 | imm | *ena* | *CR43399* |
| 2L:15339510-15340140 | 163.5 | 19.7956 | imm | *esg* | *nht* |
| 3L:11021940-11022760 | 376.5 | 23.3065 | imm | *Fad2* | *CG32079* |
| 3L:16408630-16409310 | 215.5 | 21.9985 | imm | *fax* | *TMS1* |
| 2R:21915220-21915850 | 146.9 | 19.8603 | imm | *Fili* | *CG43742* |
| X:10386420-10386950 | 34.6 | 17.7739 | imm | *flw* | *Psf3* |
| 3R:10108900-10109880 | 1264.1 | 36.246 | imm | *Fmr1* | *CG3940* |
| X:12924960-12926150 | 1193.7 | 32.8636 | imm | *fne* | *CR45626* |
| 3L:20942060-20942780 | 587.9 | 30.6564 | imm | *fng* | *CR45243* |
| 2L:3655620-3656530 | 469.3 | 23.451 | imm | *for* | *Drgx* |
| 3L:11119730-11120600 | 313.1 | 21.3896 | imm | *FoxK* | *mRpL2* |
| 3L:11118380-11119070 | 265.9 | 20.3043 | imm | *FoxK* | *mRpL2* |
| 3R:18584280-18584870 | 180.1 | 20.0222 | imm | *fray* | *qin* |
| X:18152080-18152590 | 35.1 | 17.7398 | imm | *Frq1* | *CG43229* |
| X:18158830-18159570 | 505.9 | 25.1596 | imm | *Frq1* | *Frq2* |
| 3R:18436700-18437580 | 947.2 | 28.9194 | imm | *fru* | *CG31122* |
| 3R:18480370-18481150 | 477.2 | 20.2579 | imm | *fru* | *CG31122* |
| 3R:18473650-18474510 | 388.1 | 25.0564 | imm | *fru* | *CG31122* |
| 3R:18424390-18425030 | 269.2 | 22.2278 | imm | *fru* | *CG31122* |
| 3R:18450110-18450660 | 105.4 | 18.1269 | imm | *fru* | *CG31122* |
| 3R:18546810-18547320 | 34.7 | 17.9977 | imm | *fru* | *CG7691* |
| 3R:18499870-18500370 | 16.3 | 16.302 | imm | *fru* | *CG7691* |
| 3L:9675310-9676060 | 557.1 | 24.0986 | imm | *fry* | *CG16717* |
| X:8058580-8059370 | 593 | 24.2343 | imm | *fs(1)h* | *mys* |
| 3L:18781450-18782250 | 560.9 | 25.7949 | imm | *ftz-f1,CR45939* | *ftz-f1,CR45939* |
| 3R:25467580-25468270 | 68.1 | 18.0779 | imm | *Fur1* | *Vps33B* |
| 2R:15674430-15674950 | 53.3 | 18.4229 | imm | *fus* | *CR45143* |
| 3R:16020050-16020990 | 1071.5 | 28.4754 | imm | *GATAe* | *pnr* |
| 2R:18571600-18572350 | 273.9 | 21.6301 | imm | *GEFmeso* | *Rgk2* |
| 3R:9934050-9934650 | 187.4 | 20.0915 | imm | *Glut4EF* | *Art4* |
| 3R:9949830-9950390 | 151.8 | 25.0642 | imm | *Glut4EF* | *Art4* |
| 3R:9874520-9875050 | 33.9 | 17.0983 | imm | *Glut4EF* | *CR45029* |
| X:15497570-15498180 | 176.8 | 19.5007 | imm | *Gmap,CR44888* | *Gmap,CR44888* |
| 3R:21837310-21838260 | 1209.5 | 32.7798 | imm | *Gr93a* | *Gr93b* |
| 2R:17813810-17814480 | 338.1 | 20.9955 | imm | *grh* | *CR45270* |
| 2R:17822120-17822690 | 164.9 | 22.7167 | imm | *grh* | *CR45270* |
| 2R:17837680-17838600 | 637.1 | 23.8475 | imm | *grh* | *olf186-F* |
| 2R:17823460-17824370 | 32.9 | 16.9094 | imm | *grh* | *olf186-F* |
| 3R:8173890-8174630 | 544.9 | 25.8719 | imm | *grn* | *CR43302* |
| 3R:8181340-8182020 | 402.8 | 25.7396 | imm | *grn* | *CR43302* |
| 3R:8165460-8166300 | 828.7 | 32.7572 | imm | *grn* | *tRNA:Arg-ACG-1-10* |
| 3R:26041870-26042420 | 85.8 | 17.5643 | imm | *gro* | *E(spl)m8-HLH* |
| X:11423680-11424180 | 17.1 | 17.1449 | imm | *Gs2* | *Gr10a* |
| X:17893170-17893700 | 72.3 | 19.7234 | imm | *GSS* | *CG6788* |
| **3L:8450420-8450970**  **(S2.18)** | 104.1 | 17.9123 | imm | *Gug* | *CG6983* |
| 2L:18782600-18783100 | 17.8 | 17.8267 | imm | *ham* | *CR43804* |
| X:13284170-13285200 | 1602.5 | 39.0102 | imm | *HDAC4* | *CG15743* |
| X:13278600-13279120 | 33.2 | 16.9067 | imm | *HDAC4* | *CG15743* |
| 3R:30337510-30338700 | 2464.2 | 60.2487 | imm | *hdc* | *CR46114* |
| 3R:30339260-30340180 | 894.5 | 29.0913 | imm | *hdc* | *CR46114* |
| X:19876920-19877670 | 473.8 | 22.0254 | imm | *Hers* | *amn* |
| 3R:23130680-23131450 | 569.5 | 25.2998 | imm | *hh* | *CG31457* |
| X:10255350-10256310 | 261.4 | 20.2271 | imm | *Hk* | *CR43959* |
| X:10247130-10247640 | 37.5 | 18.836 | imm | *Hk* | *CR43959* |
| 2R:21713630-21714340 | 263.7 | 22.9842 | imm | *HmgD* | *CG30403* |
| 2R:10231050-10231700 | 152.7 | 22.0379 | imm | *Hr3* | *CG46321* |
| 2R:10213750-10214440 | 301.5 | 20.5423 | imm | *Hr3,CG46321* | *Hr3,CG46321* |
| **X:1965990-1966710**  **(S2.2)** | 563.4 | 30.7516 | imm | *Hr4* | *CG3587* |
| **X:1947150-1947800**  **(S2.1)** | 200.5 | 19.9303 | imm | *Hr4* | *CG3587* |
| **X:1991560-1992580**  **(S2.5)** | 1325.5 | 32.803 | imm | *Hr4* | *PIG-K* |
| **X:1989990-1990810**  **(S2.4)** | 822 | 29.9344 | imm | *Hr4* | *PIG-K* |
| **X:1975750-1976540**  **(S2.3)** | 701.9 | 30.9389 | imm | *Hr4* | *PIG-K* |
| 3R:10616720-10617350 | 167.6 | 22.2956 | imm | *hth* | *CR44018* |
| 3R:10586900-10587700 | 102.2 | 18.3094 | imm | *hth* | *CR44018* |
| 3R:10552660-10553180 | 53.3 | 18.8862 | imm | *hth* | *mir-4944* |
| 3R:10535760-10536270 | 33.9 | 17.5333 | imm | *hth* | *mir-4944* |
| 3R:16847300-16848120 | 430.8 | 22.5094 | imm | *iab-8* | *iab-4* |
| 2L:3533900-3534440 | 70.2 | 18.3282 | imm | *IFT57* | *Spindly* |
| X:10815260-10815770 | 36.1 | 19.0644 | imm | *Imp* | *CR45624* |
| 3L:4233440-4234250 | 420.3 | 22.1867 | imm | *ImpL2* | *CR45738* |
| X:1506860-1507370 | 34.5 | 17.4021 | imm | *inc,Nmdar2* | *inc,Nmdar2* |
| 3R:21581630-21582340 | 495.5 | 26.2738 | imm | *InR* | *CR43653* |
| 3R:21605160-21605870 | 324.2 | 20.7534 | imm | *InR* | *CR44034* |
| 3R:21616030-21616550 | 63.3 | 22.6613 | imm | *InR* | *CR44034* |
| 3R:21618190-21618940 | 568.5 | 25.8943 | imm | *InR* | *E2f1* |
| 2R:11493560-11494060 | 18.2 | 18.2973 | imm | *inv* | *E(Pc)* |
| X:13322040-13322570 | 73.9 | 19.9271 | imm | *IP3K2* | *Jafrac1* |
| 3R:14775010-14775520 | 33.7 | 17.0935 | imm | *jvl* | *CG7362* |
| 3R:5231620-5232210 | 192.5 | 20.8722 | imm | *Kat60* | *Mms19* |
| 3R:29763970-29764950 | 1177.5 | 30.1985 | imm | *kay* | *CR44953* |
| 3R:29772400-29773230 | 457.3 | 19.8217 | imm | *kay* | *CR46110* |
| X:19353500-19354210 | 446.4 | 22.7791 | imm | *kek5* | *nAChRalpha7* |
| 3R:14713670-14714910 | 1613.5 | 39.0348 | imm | *kibra* | *CG7530* |
| 3R:14718930-14719910 | 1192.3 | 32.5949 | imm | *kibra* | *CG7530* |
| 3R:14722310-14723030 | 320.3 | 20.8223 | imm | *kibra* | *CG7530* |
| X:2822810-2823350 | 33.6 | 17.2903 | imm | *kirre* | *CG12498* |
| X:3047620-3048250 | 206.2 | 20.8325 | imm | *kirre* | *CG4116* |
| 2L:248400-248950 | 67.6 | 17.8883 | imm | *kis* | *CR44218* |
| 3L:11009900-11011120 | 2640.1 | 69.7238 | imm | *klu* | *snoRNA:Me18S-G962* |
| 3L:20695370-20695880 | 34.8 | 17.7941 | imm | *kni* | *cmpy* |
| 2L:6081830-6082940 | 1747 | 42.3724 | imm | *Kr-h1* | *CR44773* |
| 2L:13550350-13551220 | 769.7 | 27.2189 | imm | *kuz* | *B4* |
| 2R:9382940-9383460 | 49 | 16.6253 | imm | *l(2)03659* | *CG8800* |
| 2R:21194800-21195300 | 17.2 | 17.231 | imm | *Lapsyn* | *tud* |
| 2R:16159670-16160520 | 763.6 | 27.7467 | imm | *lbk* | *CG10734* |
| 3R:21433990-21434770 | 481.3 | 26.355 | imm | *lbl* | *CR44089* |
| 2L:2888580-2889470 | 958.8 | 28.895 | imm | *lilli* | *NTPase* |
| 2L:2886360-2887210 | 797.9 | 29.3647 | imm | *lilli* | *NTPase* |
| X:8785380-8786320 | 1010.6 | 30.4723 | imm | *Lim1* | *CR45537* |
| 2L:19083370-19083870 | 16.4 | 16.4074 | imm | *Lim3* | *CG10700* |
| X:12591380-12592250 | 750 | 24.5312 | imm | *LIMK1* | *CG1824* |
| 3L:16865260-16865780 | 51.4 | 17.3427 | imm | *Lmpt* | *CG9951* |
| 3L:12328330-12328910 | 147.9 | 20.3582 | imm | *Lmx1a* | *CG10418* |
| 3R:22626470-22627480 | 1286.2 | 35.1323 | imm | *loco* | *CR43475* |
| X:9578700-9579230 | 68 | 17.9452 | imm | *LPCAT* | *CG42395* |
| 2R:11081070-11081720 | 149 | 21.8478 | imm | *luna* | *CG43188* |
| 2R:11102180-11102680 | 18 | 18.073 | imm | *luna* | *CG43188* |
| X:9284180-9284770 | 126.1 | 20.3319 | imm | *lz* | *c11.1* |
| X:11753640-11754550 | 971.5 | 28.308 | imm | *m* | *CG9360* |
| X:6012870-6013720 | 768 | 26.3874 | imm | *mab-21* | *CR44499* |
| 2R:14031020-14032030 | 1481.8 | 38.4709 | imm | *mam* | *CG18371* |
| 2R:14026870-14027420 | 109.8 | 20.0935 | imm | *mam* | *CG18371* |
| 2R:14000640-14001240 | 265 | 28.7339 | imm | *mam* | *mir-4978* |
| 2R:13994510-13995150 | 132.4 | 19.8178 | imm | *mam* | *RN-tre* |
| 2R:13995620-13996240 | 120.2 | 17.6438 | imm | *mam* | *RN-tre* |
| X:13889190-13890110 | 1042.6 | 32.5324 | imm | *mamo* | *AMPdeam* |
| X:13893510-13894450 | 838.6 | 25.5019 | imm | *mamo* | *AMPdeam* |
| X:13894580-13895510 | 687.8 | 27.5374 | imm | *mamo* | *AMPdeam* |
| X:13873710-13874480 | 575.2 | 23.5817 | imm | *mamo* | *AMPdeam* |
| X:13885700-13886260 | 91 | 19.4482 | imm | *mamo* | *AMPdeam* |
| X:13879450-13879970 | 52.7 | 19.122 | imm | *mamo* | *AMPdeam* |
| X:13906200-13907160 | 1103.7 | 33.5706 | imm | *mamo* | *CG11068* |
| X:13909140-13909980 | 793 | 26.6927 | imm | *mamo* | *CG11068* |
| X:13903360-13904170 | 458.2 | 26.3634 | imm | *mamo* | *CG11068* |
| X:13920720-13921590 | 341.5 | 19.8135 | imm | *mamo* | *CG11068* |
| X:13907600-13908770 | 269.3 | 26.8676 | imm | *mamo* | *CG11068* |
| X:13923510-13924140 | 212.6 | 22.606 | imm | *mamo* | *CG11068* |
| X:13960800-13961330 | 53.2 | 19.0311 | imm | *mamo* | *CG11068* |
| X:13953200-13953710 | 32.9 | 16.5783 | imm | *mamo* | *CG11068* |
| X:13933910-13934410 | 18.5 | 18.5308 | imm | *mamo* | *CG11068* |
| X:13990230-13991030 | 580.5 | 21.6426 | imm | *mamo* | *CR44655* |
| X:13988650-13989250 | 214.5 | 20.9923 | imm | *mamo* | *CR44655* |
| X:13983000-13983500 | 17.6 | 17.6027 | imm | *mamo* | *CR44655* |
| 2R:17300760-17301260 | 16.2 | 16.2125 | imm | *mbl* | *CR43661* |
| 2R:9950020-9950620 | 67.3 | 17.4426 | imm | *Mef2* | *CG15863* |
| 2R:9955200-9955720 | 50.1 | 17.1108 | imm | *Mef2* | *CG15863* |
| 2R:19467870-19468670 | 246.4 | 24.2055 | imm | *mei-W68* | *CG7744* |
| X:13234120-13234740 | 267.4 | 26.1374 | imm | *mew* | *CG32639* |
| X:13219820-13220660 | 725.7 | 24.3895 | imm | *mew* | *REG* |
| **X:9495830-9496730**  **(S2.8)** | 1104.2 | 33.754 | imm | *mgl* | *CG32700* |
| **X:9467850-9468580**  **(S2.7)** | 501.1 | 25.5165 | imm | *mgl* | *CG44815* |
| **X:9428350-9429020**  **(S2.6)** | 159.6 | 19.6551 | imm | *mgl* | *CG44815* |
| 3L:12693270-12693770 | 16.4 | 16.4937 | imm | *mirr* | *CR44552* |
| **2L:6473220-6473810**  **(s2.9)** | 102.3 | 17.8899 | imm | *mmy* | *CG9536* |
| X:17878410-17879180 | 429.3 | 21.9347 | imm | *mnb* | *CG12985* |
| X:3694850-3695350 | 16.9 | 16.9742 | imm | *Mnt* | *CR45102* |
| 3L:11552820-11553690 | 957.3 | 28.3097 | imm | *Mob2* | *mir-4968* |
| 3L:11556360-11556890 | 68.9 | 18.3225 | imm | *Mob2* | *mir-4968* |
| 2L:14995600-14996110 | 34.5 | 17.3478 | imm | *mol* | *DCTN5-p25* |
| 2R:16221100-16221770 | 125.2 | 18.9326 | imm | *mrj* | *CR43898* |
| 2R:16206470-16207010 | 68.7 | 17.5341 | imm | *mrj* | *tRNA:Gln-CTG-1-1* |
| 3L:2578200-2578970 | 661.9 | 29.2493 | imm | *msn* | *RpL8* |
| X:4246240-4246900 | 348.3 | 23.342 | imm | *Muc4B* | *CG43134* |
| X:1611540-1612500 | 1142.1 | 33.3939 | imm | *Mur2B,br* | *Mur2B,br* |
| X:1603760-1604310 | 111.4 | 19.7093 | imm | *Mur2B,br* | *Mur2B,br* |
| X:1620920-1621430 | 33.8 | 17.2055 | imm | *Mur2B,br* | *Mur2B,br* |
| 3R:21060400-21061030 | 198.1 | 19.3464 | imm | *Mvl* | *Cortactin* |
| X:3381900-3382720 | 751.8 | 31.8727 | imm | *Myc* | *CG12535* |
| X:3161770-3162380 | 154.7 | 22.5615 | imm | *N* | *CG18508* |
| X:3154820-3155370 | 111.7 | 21.6146 | imm | *N* | *CG18508* |
| X:3143370-3143890 | 54 | 19.0709 | imm | *N* | *CG18508* |
| X:3122750-3123250 | 16.8 | 16.8206 | imm | *N* | *CG18508* |
| 3R:24450720-24451440 | 103.3 | 18.2047 | imm | *nAChRalpha1* | *CR45950* |
| X:8361510-8362100 | 105.6 | 18.3013 | imm | *nAChRalpha3* | *CG1387* |
| X:8337600-8338220 | 226.9 | 20.6761 | imm | *nAChRalpha3* | *CR43695* |
| X:8333650-8334170 | 52.6 | 18.1717 | imm | *nAChRalpha3* | *CR43695* |
| 2L:14095460-14096090 | 306.5 | 26.7727 | imm | *nAChRalpha5* | *CG46301* |
| X:14653620-14654370 | 477.4 | 20.112 | imm | *NetA* | *CG5321* |
| X:14730560-14731310 | 228.6 | 21.7075 | imm | *NetB* | *hog* |
| X:14734510-14735010 | 16.2 | 16.2733 | imm | *NetB* | *hog* |
| X:13440950-13441450 | 16.7 | 16.7619 | imm | *Neto* | *CG34324* |
| X:13627700-13628200 | 16.7 | 16.7834 | imm | *NFAT* | *CG2691* |
| 3R:14346750-14347420 | 419.2 | 26.3253 | imm | *NK7.1* | *HEATR2* |
| 3R:14370110-14370620 | 35.9 | 17.9642 | imm | *NK7.1* | *snoRNA:Me18S-G1189* |
| 3R:14372760-14373270 | 32.9 | 16.9225 | imm | *NK7.1* | *snoRNA:Me18S-G1189* |
| 2L:14490020-14490930 | 673.2 | 25.3824 | imm | *noc* | *CR44731* |
| X:9042090-9043070 | 1474.4 | 45.2895 | imm | *Nost* | *CG15365* |
| X:9049040-9049560 | 54.6 | 19.2962 | imm | *Nost* | *CG15365* |
| 3L:16022920-16023860 | 972.2 | 26.527 | imm | *Notum* | *CR45998* |
| 3L:13359560-13360340 | 553.4 | 25.8834 | imm | *Nplp2* | *CG17687* |
| X:8533530-8534380 | 892.5 | 32.6587 | imm | *Nrg* | *PIP82* |
| 2L:12586930-12587670 | 392 | 21.9128 | imm | *nub* | *CR44595* |
| 3L:17486930-17487530 | 181.7 | 20.5475 | imm | *Oatp74D* | *CG6333* |
| 3L:17500470-17501240 | 501.4 | 23.0999 | imm | *Oatp74D* | *edin* |
| 3L:17503960-17504520 | 93.3 | 20.3524 | imm | *Oatp74D* | *U4-U6-60K* |
| 2R:14439810-14440400 | 123.8 | 18.187 | imm | *Oaz* | *CR45277* |
| 2R:20499250-20499770 | 51.9 | 17.9881 | imm | *Obp57c* | *Obp57b* |
| 3R:29690720-29691550 | 67 | 17.8475 | imm | *Obp99c,dmrt99B* | *Obp99c,dmrt99B* |
| X:8650500-8651370 | 645.5 | 27.8433 | imm | *oc* | *CG12772* |
| X:17802210-17802780 | 151.1 | 19.7449 | imm | *OdsH* | *CG12986* |
| X:17789570-17790520 | 1054.8 | 34.5917 | imm | *OdsH* | *unc-4* |
| 2L:14623500-14624320 | 593.2 | 27.6744 | imm | *osp* | *Adhr* |
| 2L:14667130-14667770 | 33.1 | 16.7934 | imm | *osp* | *CG15282* |
| 2L:14674520-14675020 | 17.1 | 17.1629 | imm | *osp* | *CG15282* |
| 2R:18267720-18268320 | 143.9 | 18.6809 | imm | *Ote* | *pen-2* |
| X:19322510-19323070 | 71.1 | 18.8994 | imm | *out* | *nAChRalpha7* |
| X:16534230-16535100 | 735.9 | 24.6662 | imm | *para* | *CG9903* |
| X:13748060-13748580 | 53.4 | 18.4408 | imm | *Pdcd4* | *CR42861* |
| X:13747080-13747580 | 17 | 17.0917 | imm | *Pdcd4* | *CR42861* |
| X:12895400-12895900 | 17.4 | 17.433 | imm | *Pde9* | *CG32650* |
| 2L:12655600-12656330 | 493.7 | 23.1083 | imm | *pdm2* | *Ref2* |
| **2R:8326770-8327720**  **(S2.15)** | 968.1 | 30.5078 | imm | *pdm3* | *CR44450* |
| 3L:7856220-7856870 | 139.9 | 19.2866 | imm | *Pdp1* | *CG32365* |
| 3L:7821520-7822380 | 842.9 | 27.177 | imm | *Pdp1* | *CG32369* |
| 3L:7827870-7828530 | 328.5 | 21.0044 | imm | *Pdp1* | *CG32369* |
| X:11695320-11695880 | 101.7 | 17.2295 | imm | *PhKgamma* | *bif* |
| 3R:8792450-8793110 | 84.3 | 17.3796 | imm | *Pif1A* | *Ir85a* |
| X:6625200-6625770 | 159.6 | 23.5294 | imm | *pigs* | *CG14443* |
| X:6617150-6617650 | 16.1 | 16.1828 | imm | *pigs* | *CG14443* |
| 2R:10025220-10025720 | 17.4 | 17.453 | imm | *Pka-R2* | *CR44294* |
| 3R:26528770-26529420 | 300.3 | 23.9508 | imm | *plum* | *CR45226* |
| 3R:26534070-26534900 | 524.8 | 27.1925 | imm | *plum* | *scrib* |
| 3R:23334110-23334720 | 222.2 | 21.4695 | imm | *pnt* | *CR46090* |
| 3R:23318760-23319310 | 72.6 | 18.7259 | imm | *pnt* | *CR46090* |
| X:15245740-15246280 | 90.5 | 20.6895 | imm | *PPYR1* | *CG9101* |
| 3L:6907330-6907930 | 34.9 | 18.4555 | imm | *Prat2* | *CG45413* |
| 2R:18967080-18967620 | 92.4 | 19.5284 | imm | *prod* | *CG15107* |
| 3R:11334250-11334940 | 448 | 29.3111 | imm | *pros* | *CR46008* |
| 3R:9423920-9424580 | 211.4 | 25.3568 | imm | *ps* | *alpha-Man-IIa* |
| 2R:10566450-10567280 | 672.9 | 23.9063 | imm | *psq* | *acal* |
| 2R:10563970-10564770 | 529.2 | 21.4746 | imm | *psq* | *acal* |
| 2R:10553920-10554630 | 493.2 | 31.1384 | imm | *psq* | *acal* |
| 2R:10555060-10555740 | 370.6 | 22.935 | imm | *psq* | *acal* |
| 2R:10568590-10569260 | 226.4 | 21.3323 | imm | *psq* | *acal* |
| 2R:10582650-10583940 | 1198.7 | 29.6985 | imm | *psq* | *CG11883* |
| 3L:1349660-1350180 | 59 | 20.1217 | imm | *Ptp61F* | *312* |
| 3R:29381930-29382650 | 437.8 | 22.6877 | imm | *Ptp99A* | *CG45546* |
| 3R:29457710-29458540 | 919.3 | 33.6724 | imm | *Ptp99A* | *CR44022* |
| 3R:9220230-9220890 | 121.9 | 19.5284 | imm | *pum* | *CR45196* |
| 3R:9235940-9236830 | 673.4 | 20.9219 | imm | *pum* | *D1* |
| 3R:14624340-14624870 | 73.9 | 20.0372 | imm | *put* | *His4r* |
| 2R:22561260-22562030 | 597.2 | 27.7803 | imm | *px* | *CG11362* |
| 2R:22514300-22514940 | 123 | 18.6362 | imm | *px* | *CG4610* |
| 2R:22546070-22547060 | 1249.8 | 29.9464 | imm | *px* | *gas* |
| X:21010470-21011260 | 451.8 | 22.7367 | imm | *r-cup* | *CG1532* |
| X:2300210-2301370 | 799.2 | 26.937 | imm | *Raf* | *CG2865* |
| X:2305760-2306480 | 518.1 | 28.8081 | imm | *Raf* | *CG2865* |
| X:2301490-2302350 | 352.6 | 24.0089 | imm | *Raf* | *CG2865* |
| X:2294500-2295000 | 18.8 | 18.8817 | imm | *Raf* | *CG2865* |
| X:3718420-3719360 | 553 | 29.1335 | imm | *Rala* | *Tlk* |
| 2R:10429840-10430350 | 34.9 | 17.4993 | imm | *RanBPM* | *CG12896* |
| 2L:7576710-7577270 | 50.4 | 17.5334 | imm | *RapGAP1* | *Spn28B* |
| 3L:9853810-9854420 | 105.2 | 17.9832 | imm | *RasGAP1* | *CG10809* |
| 3L:10567960-10568510 | 49.7 | 17.4912 | imm | *Rbfox1* | *CG6527* |
| 3L:17149760-17150310 | 120.4 | 24.4552 | imm | *Rbp6* | *CR43433* |
| X:8974840-8975340 | 18 | 18.0038 | imm | *rdgA* | *CG10962* |
| X:9023080-9024050 | 1032.1 | 33.3033 | imm | *rdgA* | *Nost* |
| 3L:9188520-9189020 | 17.8 | 17.8277 | imm | *Rdl* | *Slc45-1* |
| 3R:13995750-13996330 | 89.1 | 19.4235 | imm | *rdx* | *CR43460* |
| 3R:13983400-13983900 | 18.1 | 18.1729 | imm | *rdx* | *CR43460* |
| 3R:14020480-14021030 | 34.5 | 17.6103 | imm | *rdx* | *Cyp6d5* |
| 3R:14017470-14017970 | 16.5 | 16.5696 | imm | *rdx* | *Cyp6d5* |
| 2L:12617800-12618640 | 205.6 | 22.0272 | imm | *Ref2,nub* | *Ref2,nub* |
| 3R:24582160-24582720 | 85.5 | 17.4197 | imm | *REPTOR* | *CG13625* |
| 3R:24572760-24573310 | 49.8 | 17.1145 | imm | *REPTOR* | *CR46094* |
| 2R:23637730-23638500 | 237.6 | 20.1122 | imm | *retn* | *CR45153* |
| 2R:23632060-23632600 | 54.5 | 19.9571 | imm | *retn* | *CR45153* |
| X:5087170-5087880 | 296.2 | 21.3256 | imm | *rg* | *CG32767* |
| X:5274210-5274750 | 87.1 | 18.7066 | imm | *rg* | *CG42749* |
| X:5165490-5166310 | 616.7 | 25.8326 | imm | *rg* | *CG5062* |
| X:5171900-5172430 | 73.9 | 19.3921 | imm | *rg* | *CG5062* |
| X:5224920-5225420 | 17.1 | 17.1701 | imm | *rg* | *CR44834* |
| X:5229520-5230020 | 16.1 | 16.152 | imm | *rg* | *CR44834* |
| X:19160680-19161270 | 182.1 | 19.9427 | imm | *RhoGAP18B* | *Inx5* |
| X:20509830-20510440 | 207.9 | 20.2558 | imm | *RhoGAP19D* | *CG1812* |
| 3L:15592550-15593690 | 817.7 | 29.7348 | imm | *RhoGAP71E* | *CG7656* |
| X:17651600-17652200 | 68.1 | 17.8728 | imm | *RhoGAPp190* | *IntS2* |
| 3L:4692200-4692750 | 111.3 | 20.738 | imm | *RhoGEF64C* | *CG15876* |
| 3L:4701430-4702010 | 50.1 | 16.8876 | imm | *RhoGEF64C* | *CR43879* |
| 3L:4729520-4730330 | 152.9 | 21.5357 | imm | *RhoGEF64C* | *CR45983* |
| 2R:19268750-19269350 | 159.6 | 19.1801 | imm | *rib* | *tbrd-2* |
| X:19259980-19260570 | 145.4 | 20.0406 | imm | *rictor* | *Vav* |
| 3R:17914920-17915430 | 34.2 | 17.1334 | imm | *Rim* | *CG43445* |
| 3R:7282080-7283030 | 207.6 | 21.1322 | imm | *rn* | *CR44333* |
| 3R:7309440-7310160 | 310.9 | 18.2235 | imm | *rn* | *CR44334* |
| 3R:7288390-7289050 | 374.7 | 27.1941 | imm | *rn* | *RacGAP84C* |
| 2L:1408390-1409270 | 141.4 | 19.0183 | imm | *robo2* | *CG43401* |
| X:16634810-16635310 | 17.7 | 17.772 | imm | *Rok* | *CR45001* |
| 2R:15135090-15135930 | 1026.3 | 38.3102 | imm | *Rpb12* | *CG8089* |
| X:14824800-14826020 | 1116.2 | 31.4972 | imm | *rut* | *CG14408* |
| 2R:20935050-20935560 | 37.2 | 18.9895 | imm | *Rx* | *Act57B* |
| 2L:11445520-11446440 | 750.7 | 24.4686 | imm | *salm* | *CG43355* |
| 2L:11440630-11441160 | 69.9 | 17.8958 | imm | *salm* | *CG43355* |
| 2L:11379100-11379600 | 16.3 | 16.3997 | imm | *salr* | *CR43681* |
| 2L:11044770-11045680 | 940.5 | 30.4356 | imm | *Samuel* | *CG18666* |
| 2L:11050330-11050830 | 16.8 | 16.8743 | imm | *Samuel* | *CG18666* |
| 2R:18878510-18879500 | 729.8 | 27.5077 | imm | *sano* | *CG43109* |
| **2R:18306580-18307470**  **(S2.12)** | 739 | 24.6131 | imm | *sbb* | *CG14505* |
| **2R:18333120-18333620**  **(S2.14)** | 16.1 | 16.1474 | imm | *sbb* | *Tango8* |
| **2R:18310870-18311370**  **(S2.13)** | 15.8 | 15.8523 | imm | *sbb* | *Tango8* |
| X:15811650-15812260 | 213.3 | 19.2011 | imm | *sd* | *CG8509* |
| X:8213710-8214230 | 34.6 | 18.0127 | imm | *sdt* | *CG2147* |
| X:12601120-12601850 | 152.3 | 17.891 | imm | *Sec16* | *CG1824* |
| 2R:17682680-17683310 | 199 | 20.3245 | imm | *Sema1b* | *HPS4* |
| 2R:16513930-16514490 | 121.7 | 18.3475 | imm | *Sema2a* | *knon* |
| 3R:27194490-27195050 | 113.9 | 20.6631 | imm | *Ser* | *CR34006* |
| 3R:27192260-27192760 | 19.3 | 19.388 | imm | *Ser* | *CR34006* |
| X:2634320-2634940 | 156.8 | 18.0328 | imm | *sgg* | *PI4KIIIalpha* |
| X:2642550-2643080 | 33.8 | 17.2344 | imm | *sgg* | *PI4KIIIalpha* |
| 3L:6964190-6964720 | 49.6 | 16.7943 | imm | *sgl* | *CG10064* |
| X:18008150-18009060 | 888.4 | 32.1023 | imm | *Sh* | *CG12672* |
| X:18000400-18001080 | 341.3 | 22.8588 | imm | *Sh* | *CG12672* |
| X:17994720-17995390 | 310.1 | 22.0895 | imm | *Sh* | *CG12672* |
| X:17997270-17997820 | 111.6 | 20.8843 | imm | *Sh* | *CG12672* |
| X:18054050-18054850 | 563.3 | 25.4319 | imm | *Sh* | *CG15373* |
| X:18050460-18051060 | 92.4 | 19.6905 | imm | *Sh* | *CG15373* |
| X:18052210-18052720 | 34.1 | 17.6414 | imm | *Sh* | *CG15373* |
| X:17959670-17960200 | 70 | 18.0144 | imm | *Sh* | *CG6847* |
| X:17963370-17963930 | 52.4 | 18.1232 | imm | *Sh* | *CG6847* |
| 3L:5248960-5249470 | 34.4 | 17.6803 | imm | *shep* | *CG4669* |
| 3L:5276840-5277720 | 696.6 | 25.5822 | imm | *shep* | *CR45741* |
| 3L:5285550-5286460 | 431.3 | 22.2649 | imm | *shep* | *CR45741* |
| 3L:5184680-5185620 | 947.3 | 26.4309 | imm | *shep* | *Srp54k* |
| 2R:11175240-11175800 | 108 | 18.413 | imm | *shn* | *CG13229* |
| 2R:14307080-14307660 | 156.9 | 20.6771 | imm | *Shroom* | *tej* |
| 2L:19027090-19028030 | 378.1 | 20.2818 | imm | *Sidpn* | *CR43700* |
| 3L:5729000-5729730 | 303.3 | 25.2003 | imm | *sif,CG46320* | *sif,CG46320* |
| 3L:5731010-5731570 | 112.6 | 19.7519 | imm | *sif,CG46320* | *sif,CG46320* |
| 2R:12586750-12587270 | 54 | 18.5545 | imm | *Sin3A* | *CR30055* |
| X:5363970-5364670 | 410.4 | 23.0609 | imm | *SK* | *CanB* |
| X:5339990-5340590 | 163.8 | 19.6035 | imm | *SK* | *CanB* |
| 3L:21026020-21026850 | 350.3 | 20.836 | imm | *skd* | *CG10585* |
| 3L:21008720-21009460 | 369 | 22.6401 | imm | *skd* | *scaRNA:PsiU6-40* |
| 3R:22157800-22158420 | 223.9 | 20.7816 | imm | *SKIP* | *CG42390* |
| 2R:19616760-19617280 | 51.2 | 17.5553 | imm | *sm* | *CR44474* |
| 2R:19630470-19631140 | 297.8 | 17.5826 | imm | *sm* | *CR44503* |
| 2L:4868640-4869160 | 53.8 | 19.9942 | imm | *smog* | *Cf2* |
| X:12699150-12699940 | 649.7 | 26.2853 | imm | *Smr* | *CG15725* |
| X:12696740-12697290 | 90.1 | 18.6382 | imm | *Smr* | *CG15725* |
| X:12716450-12717030 | 155.9 | 21.2337 | imm | *Smr* | *CR43963* |
| X:12737090-12737670 | 141.4 | 19.6963 | imm | *Smr* | *CR45623* |
| X:7978600-7979210 | 100.8 | 18.6131 | imm | *sn* | *sws* |
| 2L:7952170-7952670 | 32.6 | 16.9091 | imm | *Snoo* | *CG7231* |
| 2L:7974220-7974720 | 17.6 | 17.664 | imm | *Snoo* | *CG7231* |
| 2L:7953370-7953870 | 15.9 | 15.9564 | imm | *Snoo* | *CG7231* |
| 2R:10534800-10535310 | 35.4 | 18.5795 | imm | *snoRNA:lola-a* | *snoRNA:lola-b* |
| 2R:10538200-10538870 | 230.9 | 22.0951 | imm | *snoRNA:lola-d* | *snoRNA:lola-c* |
| X:17826940-17827610 | 313.2 | 20.7294 | imm | *Socs16D* | *CR44691* |
| X:9729100-9729850 | 484.1 | 23.2042 | imm | *Sp1* | *CR43498* |
| X:9739350-9739880 | 50.1 | 16.8787 | imm | *Sp1* | *CR43498* |
| X:9749460-9749960 | 16.6 | 16.6857 | imm | *Sp1* | *CR43498* |
| 2L:166450-167270 | 667.9 | 28.0507 | imm | *spen* | *CG33635* |
| 2R:16129950-16130470 | 33 | 16.8703 | imm | *spin* | *CG30095* |
| 2R:7144960-7145580 | 127.6 | 19.4137 | imm | *Spn43Aa* | *CG12828* |
| X:5417910-5418410 | 16.8 | 16.8699 | imm | *spoon* | *Usp16-45* |
| 3R:25883830-25884330 | 16.8 | 16.8362 | imm | *SppL* | *Tsp96F* |
| 3R:19165550-19166250 | 145.9 | 19.0185 | imm | *sqz* | *Nsun5,CG42359* |
| 3R:18107260-18107950 | 105.4 | 17.9427 | imm | *sr* | *tRNA:Gly-TCC-1-3* |
| 3R:18198340-18198930 | 193.9 | 21.5046 | imm | *Ssdp* | *CG7985* |
| X:14037550-14038560 | 1378.1 | 42.9175 | imm | *Ste:CG33247* | *Ste:CG33246* |
| X:10741760-10742370 | 200.6 | 19.9393 | imm | *stx* | *ras* |
| 2R:12996860-12997510 | 69.2 | 18.5105 | imm | *Su(z)2* | *CG33798* |
| X:7096810-7097780 | 1098 | 28.6828 | imm | *Sxl* | *CG4615* |
| 3R:20784010-20784700 | 371.6 | 21.8725 | imm | *Syp* | *Takl1* |
| 3R:20782550-20783140 | 191.9 | 20.6656 | imm | *Syp* | *Takl1* |
| 2L:9190010-9190580 | 113.3 | 20.2119 | imm | *tai* | *CG9586* |
| 3R:16240900-16241650 | 537.8 | 25.9178 | imm | *tara* | *CR45643* |
| 3R:16237090-16237900 | 387.9 | 22.5693 | imm | *tara* | *CR45643* |
| 3R:16231370-16232020 | 175.4 | 21.2609 | imm | *tara* | *CR45643* |
| 3R:16239450-16240000 | 66.9 | 17.0014 | imm | *tara* | *CR45643* |
| 3R:16228830-16229350 | 53.2 | 18.7634 | imm | *tara* | *CR45643* |
| X:15886220-15886770 | 87.5 | 18.7628 | imm | *tay* | *MSBP* |
| 3L:2879240-2880100 | 750.7 | 26.4969 | imm | *Tet* | *CR44531* |
| 3L:2823310-2824010 | 371 | 21.7829 | imm | *Tet* | *pgant6* |
| 3L:2820810-2821470 | 125 | 20.0035 | imm | *Tet* | *pgant6* |
| 3L:2805500-2806010 | 34.5 | 18.4563 | imm | *Tet* | *pgant6* |
| 3L:2808200-2808700 | 16.9 | 16.9603 | imm | *Tet* | *pgant6* |
| 3R:18261230-18261920 | 348.6 | 22.247 | imm | *Tgs1* | *Rpb4* |
| X:19127420-19127930 | 38.5 | 20.5358 | imm | *tgy* | *CG15882* |
| 2R:11789770-11790740 | 1153.3 | 28.6143 | imm | *ths* | *Ir48c* |
| 2L:5262140-5262870 | 497.1 | 23.9753 | imm | *tkv* | *Cyp4ac1* |
| 2L:5237150-5237860 | 259.7 | 20.3408 | imm | *tkv* | *tRNA:Asp-GTC-2-1* |
| X:3761940-3762770 | 672.3 | 23.1047 | imm | *Tlk* | *mir-4962* |
| X:3748840-3749520 | 176.5 | 22.2866 | imm | *Tlk* | *mir-4962* |
| X:3746620-3747460 | 575.7 | 24.478 | imm | *Tlk* | *Rala* |
| X:3740670-3741360 | 421.3 | 24.1912 | imm | *Tlk* | *Rala* |
| X:3733510-3734020 | 33.7 | 16.9286 | imm | *Tlk* | *Rala* |
| X:3734700-3735400 | 33.2 | 16.7909 | imm | *Tlk* | *Rala* |
| 3R:15291650-15292150 | 16.2 | 16.2556 | imm | *Tm1* | *CG45218* |
| 3L:10867990-10868710 | 34 | 17.4737 | imm | *tna* | *CG6418* |
| 3R:24720390-24721080 | 380.2 | 25.7431 | imm | *tok* | *CG13630* |
| X:12649130-12650410 | 828.6 | 27.5617 | imm | *Tomosyn* | *CkIalpha* |
| **2R:11604520-11605020**  **(S2.11)** | 16.6 | 16.6183 | imm | *tou* | *CR43907* |
| 2R:11603050-11603930 | 1105.2 | 39.2021 | imm | *tou* | *snoRNA:Psi28S-1180* |
| **2R:11596760-11597560**  **(S2.10)** | 693.6 | 26.0572 | imm | *tou* | *snoRNA:Psi28S-1180* |
| 3L:6942940-6943920 | 1357.7 | 38.2922 | imm | *tow* | *CR45412* |
| 3L:20399770-20400350 | 128 | 21.2808 | imm | *trbl* | *CR45677* |
| X:5679420-5679940 | 53.6 | 18.6808 | imm | *Tre1* | *Gr5a* |
| 3L:377840-378430 | 169.7 | 20.7203 | imm | *trh* | *CG13884* |
| 3L:1032040-1032690 | 318 | 26.5423 | imm | *trio* | *CG9205* |
| X:18552560-18553320 | 554.8 | 27.9797 | imm | *tRNA:Pro-CGG-2-1* | *CG15047* |
| X:14082580-14083320 | 165.1 | 19.669 | imm | *tRNA:Ser-AGA-1-1* | *CR46242* |
| X:14069120-14070050 | 648.9 | 31.4836 | imm | *tRNA:Ser-AGA-1-1* | *Ste:CG33236* |
| X:14073690-14074280 | 200 | 21.8065 | imm | *tRNA:Ser-AGA-1-1* | *Ste:CG33236* |
| X:14029150-14029900 | 345.4 | 25.9787 | imm | *tRNA:Ser-AGA-2-1* | *tRNA:Ser-AGA-3-1* |
| **3R:14278540-14279370**  **(S2.19)** | 811.3 | 30.6426 | imm | *trx* | *trx* |
| **3R:14284920-14285770**  **(S2.20)** | 738 | 25.4401 | imm | *trx* | *CR45596* |
| X:6074120-6074640 | 52 | 18.48 | imm | *Tsp5D* | *CG4666* |
| 3R:25880870-25881370 | 16.2 | 16.262 | imm | *Tsp96F* | *SppL* |
| 3R:26964120-26964770 | 184.3 | 18.1543 | imm | *Tsp97E* | *Gr97a* |
| 3R:31713210-31713720 | 33.5 | 17.1754 | imm | *ttk* | *CR34046* |
| 3R:31723550-31724180 | 320.4 | 26.956 | imm | *ttk* | *CstF50* |
| 3R:10133350-10134190 | 645.1 | 22.7703 | imm | *tws* | *TAF1B* |
| 2R:21577650-21578560 | 542.9 | 22.0932 | imm | *twz* | *CG30222* |
| 2R:21572850-21573380 | 67.8 | 17.2736 | imm | *twz* | *CG30222* |
| X:8091300-8091800 | 17.4 | 17.4776 | imm | *UbcE2H* | *CG2258* |
| 3R:16702510-16703310 | 529.8 | 23.4263 | imm | *Ubx* | *bxd* |
| 3R:16685460-16686710 | 697.6 | 26.57 | imm | *Ubx* | *modSP* |
| 3R:16675160-16675680 | 52.6 | 18.4167 | imm | *Ubx* | *modSP* |
| 2L:15273630-15274400 | 549.3 | 22.6761 | imm | *UK114* | *Cul3* |
| 2R:15349150-15350060 | 1242.9 | 40.9035 | imm | *unc-5* | *Hr51* |
| X:13601430-13602370 | 1081.9 | 32.3912 | imm | *up* | *CR44654* |
| 2L:510300-511050 | 294.2 | 22.4795 | imm | *ush* | *CR46259* |
| 2L:530950-531760 | 469.6 | 22.0813 | imm | *ush* | *lwr* |
| 2L:10276370-10277020 | 32.2 | 16.3766 | imm | *Utx* | *CG34043* |
| 2L:12987110-12987620 | 36.6 | 18.638 | imm | *Vha68-3* | *CG12404* |
| 3R:19225680-19226180 | 16.6 | 16.6741 | imm | *vib* | *CG11703* |
| 3L:5834080-5834980 | 465.3 | 24.3529 | imm | *vn* | *mad2* |
| 3L:5841340-5842050 | 316 | 23.9195 | imm | *vn* | *mad2* |
| X:5604300-5604880 | 95.2 | 20.55 | imm | *Vsx1* | *CG34435* |
| X:5533270-5533970 | 209.2 | 18.7171 | imm | *Vsx2* | *snoRNA:Psi28S-3342* |
| X:5560180-5560940 | 545.3 | 25.5622 | imm | *Vsx2* | *Vsx1* |
| X:5567910-5568520 | 242.1 | 25.0812 | imm | *Vsx2* | *Vsx1* |
| X:2157940-2158530 | 187.7 | 19.8501 | imm | *wapl* | *Cyp4d1* |
| 3R:29000700-29001260 | 67.8 | 17.5774 | imm | *wat* | *mir-4947* |
| 3L:19634870-19635620 | 100.9 | 17.0808 | imm | *wnd* | *CG8786* |
| 3R:30804100-30804640 | 92.7 | 19.1262 | imm | *wts* | *dj-1beta* |
| 3R:30764180-30764760 | 143.6 | 19.8689 | imm | *zfh1* | *CR45189* |
| X:19774050-19774660 | 255.8 | 23.3364 | imm | *zld* | *CG12702* |

Note: Bolded font indicates sequences that were tested for enhancer activity in reporter transgene assays.
