# Supplementary material for: A novel role for trithorax in the gene regulatory network for a rapidly evolving fruit fly pigmentation trait
Source: PLoS Genet. 2023 Feb 16;19(2):e1010653. doi: 10.1371/journal.pgen.1010653 (PMC9977049; doi:10.1371/journal.pgen.1010653)
Supplement: S5 Table — (DOCX) [file pgen.1010653.s025.docx]

**S5 Table. Iterative SCRMshaw improvement.**

| **SCRMshaw round** | **Training set size** | **Number of CREs validated in vivo** | **Predictions with enhancer activity** | **Predictions with enhancer activity in the pupal abdomen epidermis** | **Predictions with no activity** |
| --- | --- | --- | --- | --- | --- |
| 1 | 8 | 18 | 16 (89%) | 12 (67%) | 2 (11%) |
| 2 | 16 | 22 | 19 (86%) | 16 (73%) | 3 (14%) |
