# Supplementary material for: A novel role for trithorax in the gene regulatory network for a rapidly evolving fruit fly pigmentation trait
Source: PLoS Genet. 2023 Feb 16;19(2):e1010653. doi: 10.1371/journal.pgen.1010653 (PMC9977049; doi:10.1371/journal.pgen.1010653)
Supplement: S6 Table — (DOCX) [file pgen.1010653.s026.docx]

**S6 Table. Sequences targeted by gRNAs for CRISPR/Cas9 deletion of *trithorax* intron 1 CREs.**

| **Gene** | **CRE** | **target name** | **CRISPR target sequence (PAM)** |
| --- | --- | --- | --- |
| *trithorax* | S2.19 | S2.19 gRNA1 | TCATCATCTCAATACATCCT (CGG) |
| *trithorax* | S2.19 | S2.19 gRNA2 | AATTGCACCTTTCCTTGCTC (AGG) |
| *trithorax* | S2.20 | S2.20 gRNA1 | GCTGCTAGTCGCACTCAACG (AGG) |
| *trithorax* | S2.20 | S2.20 gRNA 2 | CAAGATCTCGCATTAGCTTA (CGG) |
