# Supplementary material for: A novel role for trithorax in the gene regulatory network for a rapidly evolving fruit fly pigmentation trait
Source: PLoS Genet. 2023 Feb 16;19(2):e1010653. doi: 10.1371/journal.pgen.1010653 (PMC9977049; doi:10.1371/journal.pgen.1010653)
Supplement: S7 Table — (DOCX) [file pgen.1010653.s027.docx]

**S7 Table. Primer pairs used to create CRE reporter transgenes to test 18 predicted CREs from the first dorsal pupal abdomen CRE training set**

| **Forward primer with introduced restriction enzyme site (lower case)** | **Reverse primer with introduced restriction enzyme site (lower case)** | **pCRE name** |
| --- | --- | --- |
| TTCCGggcgcgccACATACGAAGAGTGAGAGCGAG | TTGCCcctgcaggAGTAAAGGAAGAATGAGGAGAGC | S1.1 |
| TTCCGggcgcgccTCGTAGTCTAGGACATAACAG | TTGCCcctgcaggCCAACAACAACAATAAAGCC | S1.2 |
| TTCCGggcgcgccACTTCGCTATTTGCCTCTCGTC | TTGCCcctgcaggGTACTACGAACAGACGAACC | S1.3 |
| TTCCGggcgcgccACGGACATACGGACAAACTG | TTGCCcctgcaggATTCAACAAGGTATGGCATTC | S1.4 |
| TTCCGggcgcgccGTTGCAGATACGAGGGCTGG | TTGCCcctgcaggCTCATCAGATTCAAATTCTGAGC | S1.5 |
| TTCCGggcgcgccGGCAATGATTTTATAGCACCG | TTGCCcctgcaggGAAAAGACCTTCAGGAATAGCC | S1.6 |
| TTCCGggcgcgccCAGCAACACCAGCCTTCCATC | TTGCCcctgcaggCAGAATCTTTGTCACTGTGCC | S1.7 |
| TTCCGggcgcgccGCTGGATTAAATGCTGTCTGG | TTGCCcctgcaggCGAGAAATACGAGAACCGAAAAG | S1.8 |
| TTCCGggcgcgccGGCTTTGTGGTCCTCTAATGTG | TTGCCcctgcaggCTACGACTGCAGCGTTCTTGTTC | S1.9 |
| TTCCGggcgcgccGTTTATTTTCCCTGCCCACAATC | TTGCCcctgcaggATGCGTTTAGTACTTTATCGAC | S1.10 |
| TTCCGggcgcgccGACCTTCAGCTAAGTCACTAG | TTGCCcctgcaggAGAAAAGTGGCGATAGGTAGG | S1.11 |
| TTCCGggcgcgccGCACTGGGAAAATGATTAGC | TTGCCcctgcaggCCCATCGGATCTACTTACTG | S1.12 |
| TTCCGggcgcgccTCTCTGATGGTTATCTTATGC | TTGCCcctgcaggCGTTACTCGACTGTTATTGGAC | S1.13 |
| TTCCGggcgcgccCATTCTGGCACACAACCAGAC | TTGCCcctgcaggGTGCAATAAATCCAGAGACC | S1.14 |
| TTCCGggcgcgccCACAAGAACGCAAAAAGGATGG | TTGCCcctgcaggACCGAAAGTTATAGCCAACG | S1.15 |
| TTCCGggcgcgccGAATTAAACAGCCTGAACACAGC | TTGCCcctgcaggCAAAGGAAGCGCAGGTGACC | S1.16 |
| TTCCGggcgcgccCACTATCGCTGTTTCATCTATC | TTGCCcctgcaggCATTCACCCACCCTCAGTGG | S1.17 |
| TTCCGggcgcgccCGATGAGCTAATATCTAAAGACG | TTGCCcctgcaggGTGTGGTCGAAGAGAAATTAAC | S1.18 |

Note: Lower case letters indicate a sequence for an introduced restriction enzyme site. *Asc*I is ggcgcgcc and *Sbf*I is cctgcagg.
