# Supplementary material for: A novel role for trithorax in the gene regulatory network for a rapidly evolving fruit fly pigmentation trait
Source: PLoS Genet. 2023 Feb 16;19(2):e1010653. doi: 10.1371/journal.pgen.1010653 (PMC9977049; doi:10.1371/journal.pgen.1010653)
Supplement: S8 Table — (DOCX) [file pgen.1010653.s028.docx]

**S8 Table. Primer pairs used to create CRE reporter transgenes to test 22 predicted CREs from the second dorsal pupal abdomen CRE training set**

| **Forward primer with introduced restriction enzyme site (lower case)** | **Reverse primer with introduced restriction enzyme site (lower case)** | **pCRE name** |
| --- | --- | --- |
| TTGCCcctgcaggTCCGTTCAATAATGACGTCAATTCC | TTCCGggcgcgccCCCCGGAAAACAGTCCACCCCAC | S2.1 |
| TTGCCcctgcaggCCCCATGGAGATCTACTTCTATGC | TTCCGggcgcgccGGCAGTTTTTTTCATGCTCAGGTC | S2.2 |
| TTGCCcctgcaggCGGCCGAAAAGTCATTTCCACAC | TTCCGggcgcgccAGAGCGAAAGAGATGGACGGCTG | S2.3 |
| TTGCCcctgcaggAACAACAACAACAACCAGCTATGCC | TTCCGggcgcgccGCATCCCATCGATAAACGGACATAC | S2.4 |
| TTGCCcctgcaggCGTCCACACCAACTCCACGTAC | TTCCGggcgcgccCCATGAGCCGTGAGCCGTTAACC | S2.5 |
| TTCCGggcgcgccGGGGATTAAAATGGGTGGCAGC | TTGCCcctgcaggTTAAATAGCAATCGATGTGTGGAGC | S2.6 |
| TTCCGggcgcgccGGCAATGCAACAAATTCTGTAATGC | TTGCCcctgcaggGCAATGAAATGCAATGCAACGC | S2.7 |
| TTCCGgaattcGTGAGCGAGAGAGCAGCATCGTG | TTGCCcctgcaggCTTGTGACACGTGTTACTTCAAAG | S2.8 |
| TTCCGggcgcgccCGGTGCTCAGCCGGTTTATTCG | TTGCCcctgcaggATCAACAGCCGCACGCAGTGTG | S2.9 |
| TTGCCcctgcaggACAAAACGAACAGGAAACGAGCGTG | TTCCGggcgcgccCTTTGCCACCGCATCCCCGCATC | S2.10 |
| TTGCCcctgcaggGAATAAGGTGGCGGAATGGAC | TTCCGggcgcgccCTCCCAATCAGCAAACAACAACAGC | S2.11 |
| TTCCGggcgcgccGCATTAAAAGCGAATAACAAGGAC | TTGCCcctgcaggGAAAGAGGAAGAGAAGCGAAGTC | S2.12 |
| TTCCGggcgcgccGTTGTTTCGGCCAAAATTGCTCC | TTGCCcctgcaggCCCAGTAATTCACGATTTCCTGCTG | S2.13 |
| TTCCGggcgcgccGTATGCCATTTGAAGCGCAGTGAAC | TTGCCcctgcaggCGAAACGAAACGAATGAAACGAC | S2.14 |
| TTCCGggcgcgccGTTGTTGTGTGTTGAGAGAGGTTG | TTGCCcctgcaggCTGAGTAGTTTCGAAACGGATTC | S2.15 |
| TTCCGggcgcgccCCGTGTGAGAGTGCGAGTG | TTGCCcctgcaggCACCGCTGAGGAGCTTCGCAGAG | S2.16 |
| TTGCCcctgcaggGCACAAATGGCCAACTCCAACTG | TTCCGggcgcgccTTGCACAAGGAGCAGCAAGGAGG | S2.17 |
| TTGCCcctgcaggCAACAACACTTGGCAGCAGCGG | TTCCGggcgcgccGTTAGTCCGAGGCAGGAGCATCC | S2.18 |
| TTCCGggcgcgccGGCTTAATACACGAAAAACACATG | TTGCCcctgcaggGGTAATGAGCACCATGTGGCC | S2.19 |
| TTCCGggcgcgccCAAGATCTCGCATTAGCTTACG | TTGCCcctgcaggGTGCGACTAGCAGCAGATGGTAGC | S2.20 |
| TTGCCcctgcaggTAGACGCCTACGCCTGTGTG | TTCCGggcgcgccCATTCCCATTTTAACCAGGTGATGC | S2.21 |
| TTGCCcctgcaggACAACAAATACCCCAAGAACCTCTC | TTCCGggcgcgccCCATTAACTTGTATACATGCACC | S2.22 |

Note: Lower case letters indicate a sequence for an introduced restriction enzyme site. *Asc*I is ggcgcgcc, *Eco*RI is gaattc, and *Sbf*I is cctgcagg.
