# Supplementary material for: A novel role for trithorax in the gene regulatory network for a rapidly evolving fruit fly pigmentation trait
Source: PLoS Genet. 2023 Feb 16;19(2):e1010653. doi: 10.1371/journal.pgen.1010653 (PMC9977049; doi:10.1371/journal.pgen.1010653)
Supplement: S9 Table — (DOCX) [file pgen.1010653.s029.docx]

**S9 Table. Primer pairs used to create CRE reporter transgenes with sequences orthologous to the *D. melanogaster* S2.19 and S2.20 *trithorax* intron CREs**

| **Forward primer with introduced restriction enzyme site (lower case)** | **Reverse primer with introduced restriction enzyme site (lower case)** | ***Species***  **pCRE name** |
| --- | --- | --- |
| TTCCGggcgcgccCTTAATCCACGCAAAACACATGAAAC | TTGCCcctgcaggGGTAATGAGCACCATGTGGCC | *D. sim.*  S2.19 |
| TTCCGggcgcgccCAAGATCTCGCATTAGCTTACG | TTGCCcctgcaggGTGCGACTAGCAGCAGATGGTAGC | *D. sim.*  S2.20 |
| TTCCGggcgcgccCAAGATCTCGCATTAGCTTACG | TTGCCcctgcaggGAGTGCGACTAGCAACAGACGTAG | *D. yak.*  S2.20 |
| TTCCGggcgcgccGGACTGCCTGTTTAAACTACAATGG | TTGCCcctgcaggGATAATGAGCACCATGTGGCCG | *D. bia.*  S2.19 |
| TTCCGggcgcgccCAAGATCTCGCATTAGCTTACG | TTGCCcctgcaggAGTGCAGTGGGACCTCGATAGG | *D. bia.*  S2.20 |
| TTCCGggcgcgccCCTCGGCTTTGTCTGAGCTCTC | TTGCCcctgcaggGGTGATAATGAGCATGATGAGGCC | *D. pse.*  S2.19 |
| TTCCGggcgcgccCAGTGGAGCGTGAAAAGTGCACC | TTGCCcctgcaggGCAGCCTTCCCACTCGTACAAC | *D. pse.*  S2.20 |
| TTCCGggcgcgccCGTACGCTCTCTTTGCTCCTGACG | TTGCCcctgcaggCATGATTATTATGAAGATGTAGCCTAGCC | *D. wil.*  S2.19 |

Note: Lower case letters indicate a sequence for an introduced restriction enzyme site. *Asc*I is ggcgcgcc and *Sbf*I is cctgcagg.
