# Supplementary material for: A novel role for trithorax in the gene regulatory network for a rapidly evolving fruit fly pigmentation trait
Source: PLoS Genet. 2023 Feb 16;19(2):e1010653. doi: 10.1371/journal.pgen.1010653 (PMC9977049; doi:10.1371/journal.pgen.1010653)
Supplement: S10 Table — (DOCX) [file pgen.1010653.s030.docx]

**S10 Table. Primer pairs used to create CRE reporter transgenes with truncated versions of the *D. melanogaster* S2.20 trithorax CRE**

| **Forward primer with introduced restriction enzyme site (lower case)** | **Reverse primer with introduced restriction enzyme site (lower case)** | **Truncation name** |
| --- | --- | --- |
| TTCCGggcgcgccCAAGATCTCGCATTAGCTTACG | TTGCCcctgcaggCCTCATTCTAAATCAAATCACTCTCTCGC | trunc1 |
| TTCCGggcgcgccCCTGCTGCTGCTGAGATCGTTTTTC | TTGCCcctgcaggGCCAGGGGGATTTTACTGCAAGTTTCC | trunc2 |
| TTCCGggcgcgccGCGAGAGAGTGATTTGATTTAGAATGAGG | TTGCCcctgcaggGTGCGACTAGCAGCAGATGGTAGC | trunc3 |
| TTCCGggcgcgccCAAGATCTCGCATTAGCTTACG | TTGCCcctgcaggCCGCTGCCGTTCGCTGCCTAGAAGC | frag1 |
| TTCCGggcgcgccGGCGTCAACAAATACTCAAACAGCC | TTGCCcctgcaggCAAATCACTCTCTCGCTCTCACCG | frag2 |
| TTCCGggcgcgccGCCATGTGAGCCCAATAGAGAGAGAG | TTGCCcctgcaggCTGTTAGCCAGGGGGATTTTACTGC | frag3 |
| TTCCGggcgcgccGCGACTGTCATTTACCGTTCGTTTTACGG | TTGCCcctgcaggCTGGAAAACCTGTTGATTTAATAAC | frag4 |
| TTCCGggcgcgccCGCCTCCTCTTTAAACTGTTAG | TTGCCcctgcaggGTGCGACTAGCAGCAGATGGTAGC | frag5 |

Note: Lower case letters indicate a sequence for an introduced restriction enzyme site. *Asc*I is ggcgcgcc and *Sbf*I is cctgcagg.
