# Supplementary material for: A novel role for trithorax in the gene regulatory network for a rapidly evolving fruit fly pigmentation trait
Source: PLoS Genet. 2023 Feb 16;19(2):e1010653. doi: 10.1371/journal.pgen.1010653 (PMC9977049; doi:10.1371/journal.pgen.1010653)
Supplement: S11 Table — (DOCX) [file pgen.1010653.s031.docx]

**S11 Table. Primer pairs used to create CRE reporter transgenes to test non-coding sequences selected solely for the possession of similar nucleotide compositions to a specific training set CRE**

| **Forward primer with introduced restriction enzyme site (lower case)** | **Reverse primer with introduced restriction enzyme site (lower case)** | **Genomic location** |
| --- | --- | --- |
| TTCCGggcgcgccCTCTGTGCAGCTGTGTGGAT | TTGCCcctgcaggCAGTTTGGATAACAGAGTGC | X:7749947 |
| TTCCGggcgcgccCGCATAATTTCCAGAGCAAGCG | TTGCCcctgcaggGCACTTGCAGCATGAGAATATTAC | 2L:11922488 |
| TTCCGggcgcgccGAACGCACAAACTCTCTGAGC | TTGCCcctgcaggGACTCGACAATGAAGGATCGGAAT | 3L:27532906 |

Note: Lower case letters indicate a sequence for an introduced restriction enzyme site. *Asc*I is ggcgcgcc and *Sbf*I is cctgcagg.
